# Binuclear Acetoniminato Derivatives of Iron and Chromium Carbonyls: A Theoretical Study

**DOI:** 10.1021/acsomega.6c00171

**Published:** 2026-02-26

**Authors:** Haoyu Chen, Jinfeng Luo, Yongtao Liu, Huidong Li, Qunchao Fan, Zhixiang Fan, R. Bruce King, Henry F. Schaefer III

**Affiliations:** † School of Science, Key Laboratory of High Performance Scientific Computation, 12598 Xihua University, Chengdu 610039, China; ‡ Centre for Computational Quantum Chemistry, 1355 University of Georgia, Athens, Georgia 30602, United States

## Abstract

The structures and energetics of binuclear acetoniminato metal carbonyl derivatives of the types (Me_2_C=N)_2_Fe_2_(CO)_*n*_ and (Me_2_C=N)_2_Cr_2_(CO)_*n*_ have been investigated by density functional theory. The (Me_2_C=N)_2_Fe_2_(CO)_6_ structure, with bridging Me_2_C=N ligands using only their nitrogen atoms to bridge an Fe=Fe bond related to experimentally known species, as well as a related (Me_2_C=N)_2_Cr_2_(CO)_8_ structure, appear to be energetically preferred. Among carbonyl-richer systems, the iron system energetically prefers a {Me_2_C=NC­(O)}_2_Fe_2_(CO)_6_ structure with bridging Me_2_C=NC­(O) ligands, whereas the chromium system energetically prefers a structure with a 2,3-diazabutadiene ligand having each nitrogen atom bonded to a Cr­(CO)_5_ moiety. Preferred structures for systems with fewer CO groups include the tetracarbonyl (Me_2_C=N)_2_Fe_2_(CO)_4_ in which one of the bridging Me_2_C=N uses its C=N double bond to bond to an iron atom, as well as (Me_2_C=N)_2_Cr_2_(CO)_*n*_ (*n* = 7, 6) structures with chromium–chromium multiple bonds bridged by Me_2_C=N groups.

## Graphical abstract

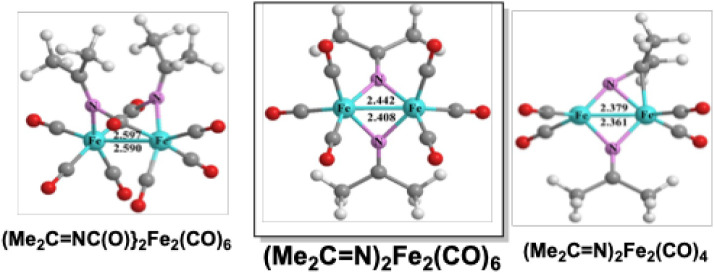

## 1 Introduction

A key development in the early history of the reaction chemistry of iron carbonyls was the discovery by Reihlen, Hieber and Spacu^1,2^ of the reactions between Fe_3_(CO)_12_ and various disulfides, mercaptans, and sulfides to give essentially air-stable products of stoichiometry RSFe­(CO)_3_, subsequently shown by molecular weight determinations to be the dimers (RS)_2_Fe_2_(CO)_6_ ^3^ At the time of their original discovery direct bonds between transition metals had not been recognized so that the nature of these products remained obscure. Eventually, however, the presence of a direct iron–iron bond was recognized and confirmed by X-ray crystallography on the ethylthio derivative^4^ (C_2_H_5_S)_2_Fe_2_(CO)_6_. The X-ray structure determination also showed that the alkylthio groups were bridging rather than terminal groups. The resulting structures can be regarded as butterfly structures where the iron–iron bond is the “body” of the butterfly and the organosulfur groups are the “wingtips” (Figure 1). The iron atoms in these butterfly structures have the favored 18-electron configuration by receiving six electrons from the three carbonyl groups, one electron from a two-center two-electron bond from one sulfur atom, two electrons from a dative S→Fe bond from the other sulfur atom, and one electron through the iron–iron bond. Stereoisomers of the (μRS)_2_Fe_2_(CO)_6_ structures are possible depending on the relative orientation of the alkyl group and the nonbonding lone pair on each sulfur atom. Such stereoisomers have been separated by column chromatography for the methylthio derivative (μCH_3_S)_2_Fe_2_(CO)_6_.^5,6^ Related butterfly species are known in which the sulfur atoms are connected by a direct bond such as (μ-S)_2_Fe_2_(CO)_6_ or by a carbon chain such as (μC_2_H_4_S_2_)­Fe_2_(CO)_6_.

**Figure 1:**
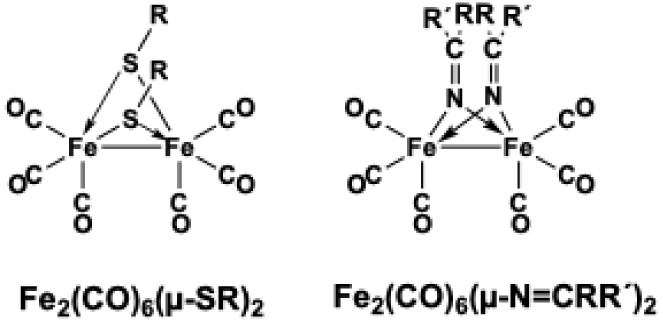
Butterfly structures of (μ-RS)_2_Fe_2_(CO)_6_ and (μ-ŔC=N)_2_Fe_2_(CO)_6_.

The butterfly structure originally found in (μ-RS)_2_Fe_2_(CO)_6_ derivatives is not limited to bridging organosulfur groups. The (μ-R_2_CN)_2_Fe_2_(R_2_CN)_4_ butterfly structure containing the ketamide ligands has attracted significant attention in recent years.^7^ Related butterfly (μ-X)_2_Fe_2_(CO)_6_ structures are also found where the bridging X groups have other donor atoms such as nitrogen or phosphorus. The electron counting in such (μ-R_2_E)_2_Fe_2_(CO)_6_ (E = N, P) structures is analogous to that given above for the (μ-RS)_2_Fe_2_(CO)_6_ derivatives likewise giving the iron atoms the favored 18-electron configuration. Furthermore, unsaturation can be added to the bridging group providing additional possibilities for electron donation from the bridging group to the iron carbonyl system. Thus, bridging ketiminato groups in structures of the type (μR_2_C=N)_2_Fe_2_(CO)_6_ provide carbon–nitrogen double bonds as a potential source of electrons for donation to the iron atoms in addition to those provided by the nitrogen lone pairs. In order to explore this possibility, we have used density functional theory to investigate the geometries and energetics of the structures of the type (Me_2_C=N)_2_Fe_2_(CO)_*n*_ (*n* = 8, 7, 6, 5, 4) and (Me_2_C=N)_2_Cr_2_(CO)_*n*_ (*n* = 10, 9, 8, 7, 6).

A number of compounds of the type (μ-RR′C=N)_2_Fe_2_(CO)_6_ are known experimentally with the most efficient syntheses involving cleavage of the N–N bond in the 2,3-diazabutadiene derivatives RR′=N–N=CRR′ with iron carbonyls.^8–10^ The required 2,3-diazabutadiene derivatives, also known as ketone azines, can readily be obtained from the corresponding ketones and hydrazine. The acetoniminato derivative (Me_2_C=N)_2_Fe_2_(CO)_6_ has also been isolated in minor quantities (3% yield) from the reaction of Na_2_Fe­(CO)_4_ with 2-bromo-2-nitrosopropane.^11^ In addition, (RRC=N)_2_Fe_2_(CO)_6_ derivatives have been isolated by the reactions of diazirines R′C­(N_2_) with Fe_2_(CO)_9_. A rather unstable intermediate of the type RR′C­{NFe­(CO)_4_}_2_ can be isolated from this reaction (Figure 2).^12^

**Figure 2:**
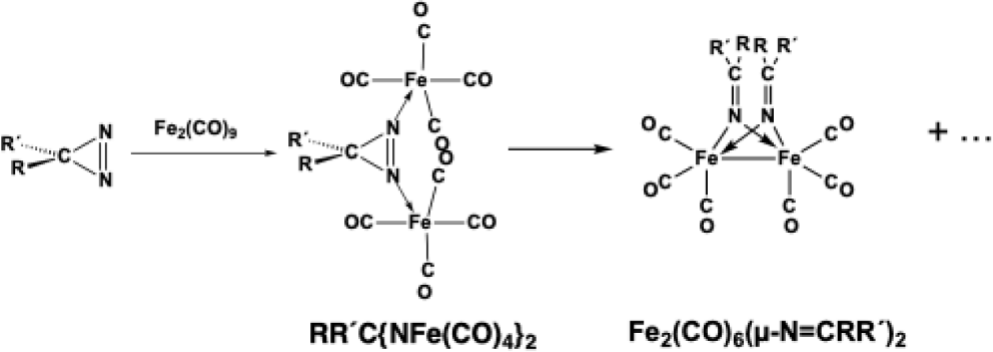
Formation of (μ-RR′C=N)_2_Fe_2_(CO)_6_ derivatives from diazirines and Fe_2_(CO)_9_.

Chromium carbonyl derivatives of the type (μ-RR′C=N)_2_Cr_2_(CO)_8_ having structures analogous to the (μ-RR′C=N)_2_Fe_2_(CO)_6_ derivatives but with one more terminal carbonyl group bonded to each metal atom also have the favored metal 18-electron configuration. Such species do not appear to have been reported. However, they are potentially accessible by reactions of the 2,3-diazabutadienes R′C=NN=CRR with a suitably chosen chromium carbonyl derivative.

## 2 Theoretical Methods

The *meta*-GGA DFT method M06-L^13,14^ as implemented in the Gaussian16 program^15^ was used for the computations. This method has been reported to give better overall performance for organometallic compounds than the first-generation functionals.^16^ The def2-TZVP basis sets^17,18^ were used for all the atoms. All geometries of the reported structures were fully optimized by using the M06-L/def2-TZVP method with the (120, 974) integration grid.

Wiberg Bond Indices (WBIs) for the metal–metal interactions in the (Me_2_C=N)_2_M_2_(CO)_*n*_ (M = Fe or Cr) compounds were determined using the MultiWFN software (Table S3 and S4).^19,20^ For these systems, the WBIs were found to range from 0.11 to 0.44 for M–M single bonds, and from 0.46 to 0.82 for M=M double bonds

The optimized structures in this paper are designated as ***M-nY-Z*** where ***M*** indicates the name of the transition metal atom, ***n*** indicates the number of CO groups, ***Y*** indicates the spin state as singlet (**S**) or triplet (**T**), and ***Z*** indicates the ranking of the structure on the relative energy.

## 3 Results and Discussion

### 3.1 Iron Carbonyl Derivatives

#### 3.1.1 (Me_2_C=N)_2_Fe_2_(CO)_6_ Structures

Only one low-energy structure **Fe-6S-1** is found for (Me_2_C=N)_2_Fe_2_(CO)_6_ (Figure 3). Structure **Fe-6S-1**, with *C*_2v_ symmetry and singlet spin state, lies at least ∼18 kcal/mol in energy below other predicted isomers. In **Fe-6S-1**, each iron atom bears three terminal carbonyl groups. An Fe–Fe bond of length 2.443 Å with a Wiberg Bond Index (WBI) of 0.44 is bridged by two equivalent Me_2_C=N groups through their nitrogen atoms. The critical point analysis suggests there is a direct bond between the two iron atoms. Two ring critical points were found nearby the bond critical points, indicating 3c-2e bonds within the two Fe–N–Fe rings. Interpreting this Fe–Fe bond as a single bond gives each iron atom in **Fe-6S-1** the favored 18-electron configuration. Although (Me_2_C=N)_2_Fe_2_(CO)_6_ has been obtained experimentally by several methods,^11,12^ its structure has not been determined by X-ray crystallography. However, the structure of the related *p*-tolyl derivative {(*p*-MeC_6_H_4_)_2_C=N}_2_Fe_2_(CO)_6_ was found by X-ray crystallography to have an Fe–Fe distance of 2.403 Å similar to the calculated Fe–Fe distance for **Fe-6S-1**. The predicted ν­(CO) frequencies of 2120, 2078, 2061, 2039, and 2029 cm^–1^ when scaled by a factor^21^ of 0.97 give 2056, 2016, 1999, 1978, and 1968 cm^–1^ which are comparable with the experimental observed values of 2077, 2029, 1982, and 1970 cm^–1^ in CH_2_Cl_2_ solution.^11^

**Figure 3:**
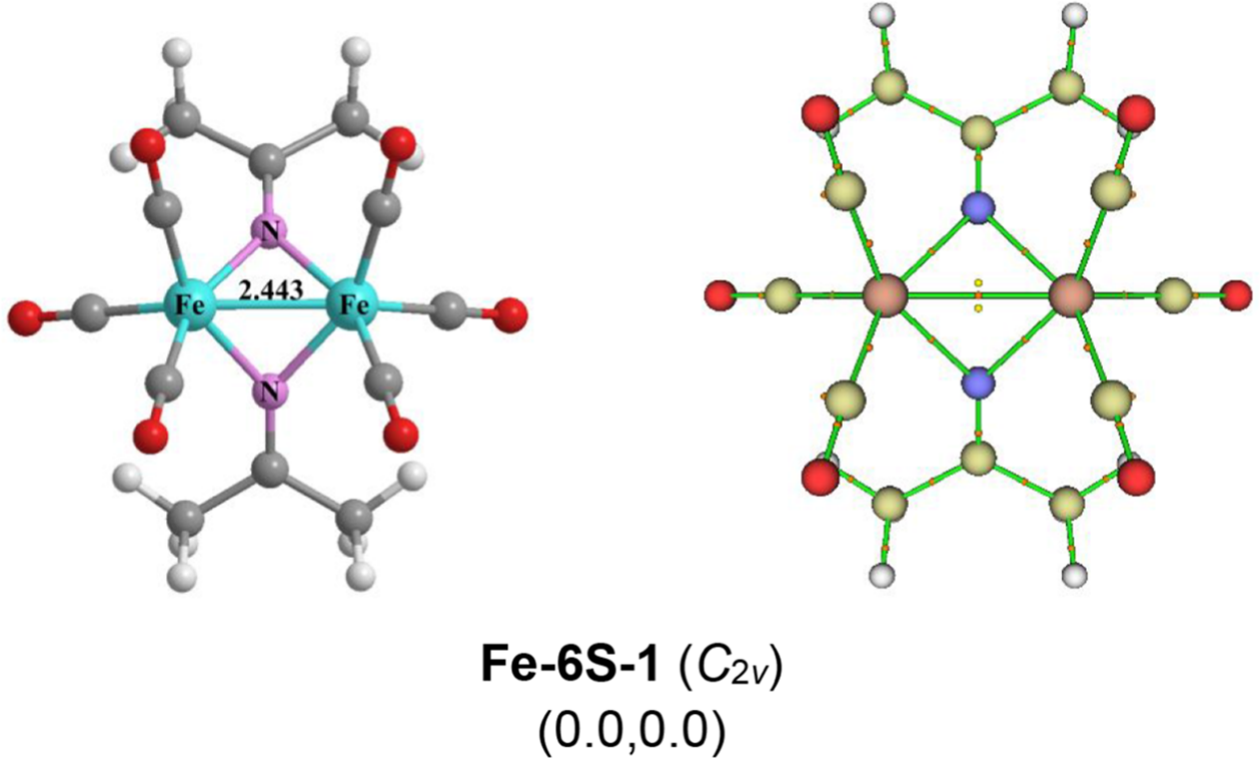
Optimized (Me_2_C=N)_2_Fe_2_(CO)_6_ structure and the critical point analysis by the AIM method within the MultiWFN software. In Figures 3–11, the numbers in parentheses are the relative energies (in kcal/mol) Δ*E* and relative Gibbs’ free energy Δ*G* at room temperature. Only the Δ*E* is reported in the text.

#### 3.1.2 (Me_2_C=N)_2_Fe_2_(CO)_5_ Structures

Three structures, namely the singlet **Fe-5S-1** and the triplets **Fe-5T-2** and **Fe-5T-3**, were found for (Me_2_C=N)_2_Fe_2_(CO)_5_ lying within ∼3 kcal/mol in energy (Figure 4). The singlet structure **Fe-5S-1** can be obtained by removing one carbonyl group from the hexacarbonyl **Fe-6S-1**. However, the Fe–Fe distance of 2.416 Å (M06-L) with a WBI of 0.46 is almost identical to that in the hexacarbonyl **Fe-6S-1**, suggesting that the Fe–Fe single bond is retained in **Fe-5S-1**. Thus, the iron atom in **Fe-5S-1** bearing two terminal CO groups has only a 16-electron configuration. The triplet structure **Fe-5T-2** having an Fe–Fe distance of 2.496 Å with a WBI of 0.38 appears to be a high-spin version of **Fe-5S-1**. The triplet structure **Fe-5T-3** has a similar coordination environment of each iron atom but with a longer Fe–Fe bond of 2.746 Å with a lower WBI of 0.15. In **Fe-5T-2** the FeC_2_N_2_ coordination environment of the iron atom bearing two CO groups is distorted tetrahedral whereas in **Fe-5T-3** the coordination environment of that iron atom is nearly tetragonal planar.

**Figure 4:**
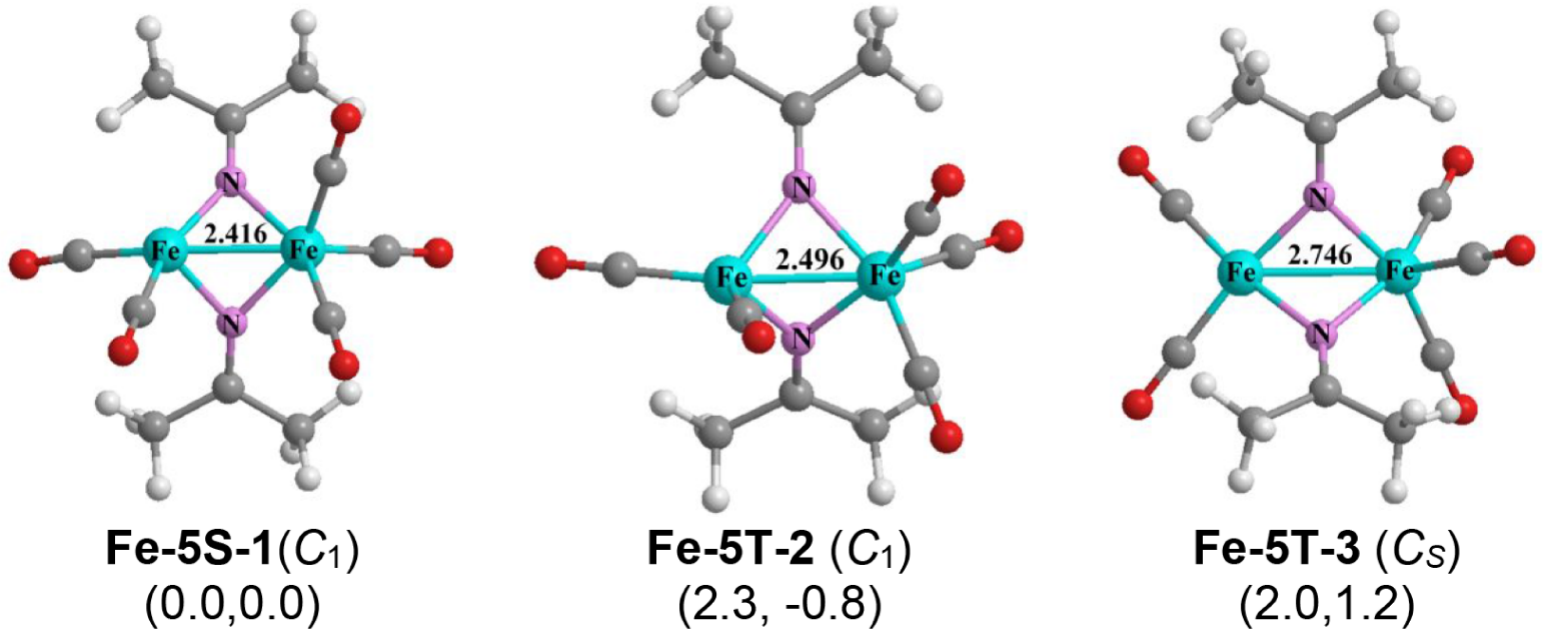
Optimized (Me_2_C=N)_2_Fe_2_(CO)_5_ structures.

#### 3.1.3 (Me_2_C=N)_2_Fe_2_(CO)_4_ Structures

Five low-energy structures were found for (Me_2_C=N)_2_Fe_2_(CO)_4_ (Figure 5). The lowest energy structure is the *C*_*s*_ singlet structure **Fe-4S-1** having an Fe—Fe distance of 2.381 Å with a WBI of 0.51, only 0.03 Å shorter than that in the (Me_2_C=N)_2_Fe_2_(CO)_*n*_ (*n* = 6, 5) structures discussed above (Figures 3 and 4), and thus can be also interpreted as a formal single bond. In **Fe-4S-1** each iron atom bears two CO groups and the Fe–Fe bond is bridged by two nonequivalent Me_2_C=N units. One of these Me_2_C=N groups bridges the Fe–Fe bond only through its nitrogen atom similar to the bridging Me_2_C=N units in all of the low-energy (Me_2_C=N)_2_Fe_2_(CO)_*n*_ (*n* = 6, 5) structures in Figures 3 and 4. However, the other bridging Me_2_C=N group is bent so that its carbon atom is brought close enough to one of its iron atoms for an Fe–C bond of length 2.116 Å. This provides two extra electrons for this iron atom from the π component of the C=N double bond. The combination of two electrons from coordination of the C=N double bond from one of the Me_2_C=N groups and the single Fe–Fe bond in **Fe-4S-1** gives the iron atom connecting to the carbon atom of one of the two C=N double bonds the favored 18-electron configuration, but the other iron atom acquires only a 16-electron configuration.

**Figure 5:**
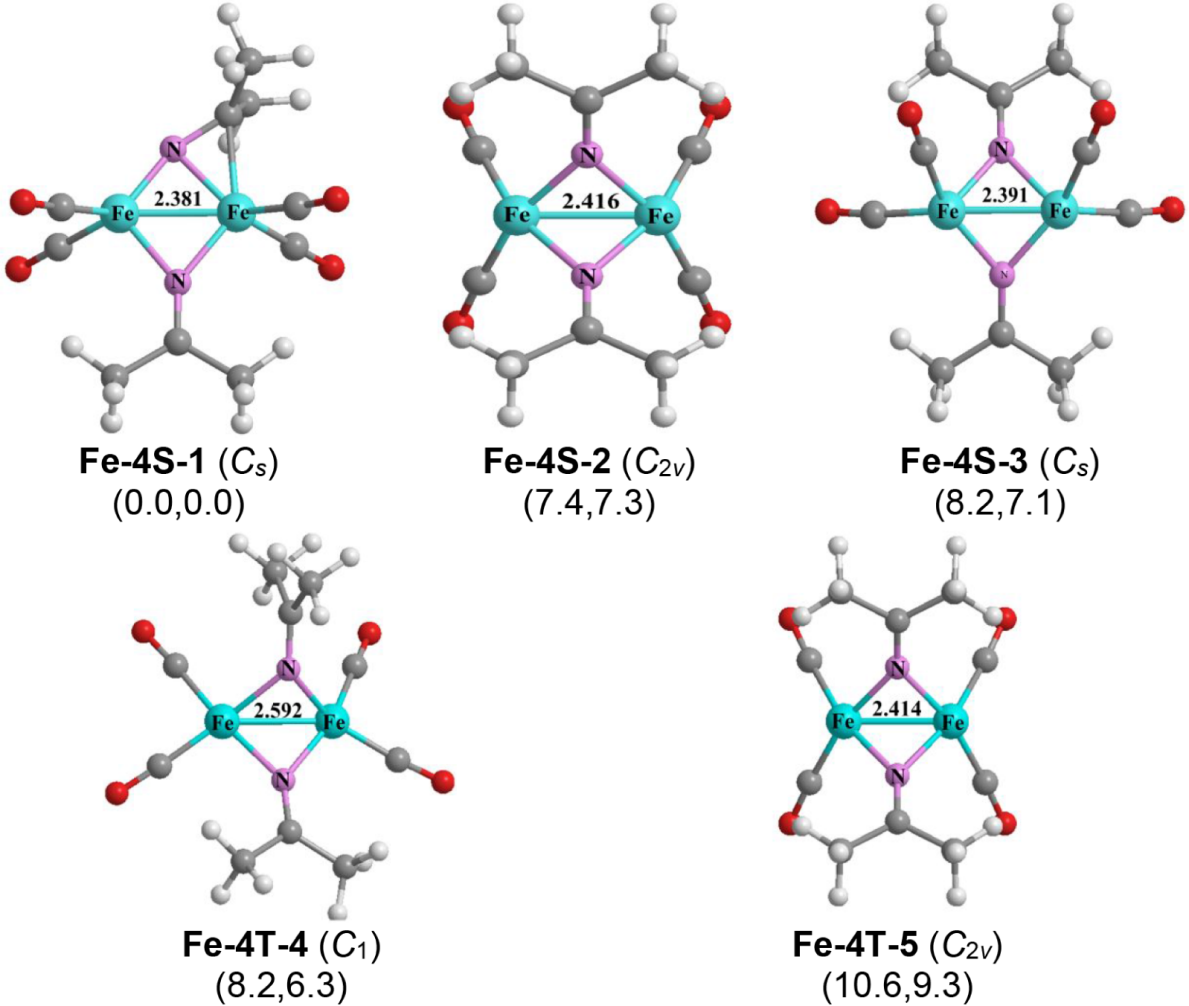
Optimized (Me_2_C=N)_2_Fe_2_(CO)_4_ structures.

The other four low-energy (Me_2_C=N)_2_Fe_2_(CO)_4_ structures, namely the singlets **Fe-4S-2** and **Fe-4S-3** as well as the triplets **Fe-4T-4** and **Fe-4T-5**, have similar coordination environments for the CO groups and the Me_2_C=N groups with energies within 10 kcal/mol of that of **Fe-4S-1** (Figure 5). Thus, in all four structures, each iron atom bears two terminal CO groups and the Me_2_C=N groups bridge the iron–iron bonds only through their nitrogen atoms similar to the low-energy the (Me_2_C=N)_2_Fe_2_(CO)_*n*_ (*n* = 6, 5) structures (Figures 3 and 4). The Fe–Fe bonds predicted for **Fe-4S-2**, **Fe-4S-3** and **Fe-4T-5** of lengths around ∼2.4 Å comparable to that in the singlet structure **Fe-4S-1**, correspond to formal single bonds. The longer iron–iron distance predicted for the triplet structure **Fe-4T-4** of 2.592 Å also corresponds to a single bond.

#### 3.1.4 Carbonyl-Rich (Me_2_C=N)_2_Fe_2_(CO)_*n*_ (*N* = 8, 7) Structures

Three low energy structures were predicted for (Me_2_C=N)_2_Fe_2_(CO)_8_ (Figure 6). The lowest energy structure is **Fe-8S-1** with *C*_2_ symmetry. This structure is related to the lowest energy and experimentally known (Me_2_C=N)_2_Fe_2_(CO)_6_ structure **Fe-6S-1** (Figure 3) by insertion of a CO group into one of the Fe–N bonds of each bridging Me_2_C=N ligand to form a bridging Me_2_C=N­(CO) ligand. The predicted ν­(CO) frequencies for the Me_2_C=N­(CO) ligands of 1741 cm^–1^ and 1744 cm^–1^ are significantly lower than those of terminal CO groups bonded to metal atoms. Such CO insertions do not affect the electron bookkeeping since a formally neutral bridging Me_2_C=NC­(O) ligand like the bridging Me_2_C=N ligands in **Fe-6S-1** formally donates three electrons to each iron atoms. Thus, each iron atom in **Fe-8S-1** like those in **Fe-6S-1** has the favored 18-electron configuration. The CO insertion into the Fe–N bonds in **Fe-6S-1** to give **Fe-8S-1** lengthens the single Fe–Fe bond indicated by the bond critical point to 2.610 Å with a WBI of 0.39. Each iron atom in **Fe-8S-1** acquires the favored 18-electron configuration.

**Figure 6:**
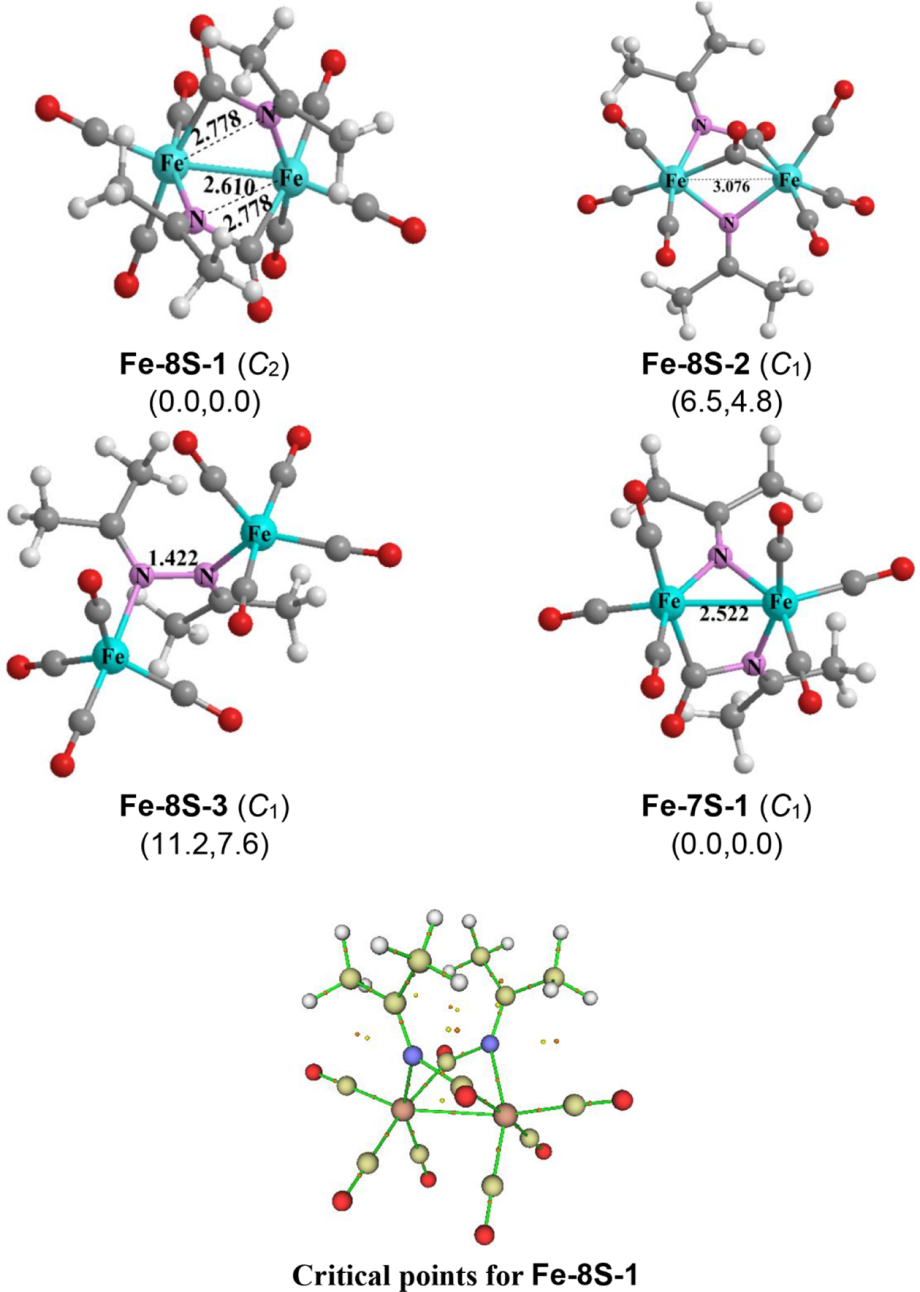
Optimized carbonyl-rich (Me_2_C=N)_2_Fe_2_(CO)*_n_* (*n* = 8, 7) structures and critical points analysis for structure **Fe-8S-1**.

The next highest energy structure for (Me_2_C=N)_2_Fe_2_(CO)_8_ is the singlet **Fe-8S-2**, lying 6.5 kcal/mol (M06-L) above **Fe-8S-1** (Figure 6). The iron atoms in **Fe-8S-2** are bridged by a CO group and a Me_2_C=N group, as well as a bridging Me_2_C=NC­(O) group with ν­(CO) frequencies of 1763 cm^–1^ similar to the bridging Me_2_C=NC­(O) groups in **Fe-8S-1**. The long iron–iron distance in **Fe-8S-2** of 3.076 Å corresponding to a low WBI of 0.11 suggests the absence of a formal iron–iron bond. However, since the bridging Me_2_C=N and Me_2_C=NC­(O) groups can each donate three electrons to the two iron atoms, then each iron atom obtains the favored 18-electron configuration without requiring any iron–iron bond.

The third (Me_2_C=N)_2_Fe_2_(CO)_8_ structure **Fe-8S-3,** lying 11.2 kcal/mol above **Fe-8S-1**, has four terminal CO groups bonded to each iron atom (Figure 6). In **Fe-8S-3** the two Me_2_C=N units are coupled to form a 2,3-diazabutadiene ligand that donates two electrons to each Fe­(CO)_4_ moiety to give each iron atom the favored 18-electron configuration. Structure **Fe-8S-3** represents an obvious intermediate in the synthesis of (Me_2_C=N)_2_Fe_2_(CO)_6_ **(Fe-6S-1**) from the 2,3-diazabutadiene Me_2_C=N–N=CMe_2_ and iron carbonyls (see Figure 2).^8–10^

Only one low energy structure, namely the singlet **Fe-7S-1**, was found for the heptacarbonyl (Me_2_C=N)_2_Fe_2_(CO)_7_ (Figure 6). Structure **Fe-7S-1** lies at least 24 kcal/mol below other predicted structures and thus appears to be highly favored. In **Fe-7S-1** the iron atoms are bridged by one Me_2_C=N group and one Me_2_C=N­(CO) group with a predicted ν­(CO) frequency of 1746 cm^–1^ for the latter. Each of these bridging groups, considered formally as neutral ligands, donates three electrons to the Fe_2_ unit. The predicted Fe–Fe distance of 2.522 Å in **Fe-7S-1** with a WBI of 0.41 corresponds to the formal single bond required to give each iron atom the favored 18-electron configuration.

### 3.2 Chromium Carbonyl Derivatives

#### 3.2.1 (Me_2_C=N)_2_Cr_2_(CO)_8_ Structures

Only one low-energy (Me_2_C=N)_2_Cr_2_(CO)_8_ structure was found, namely the singlet **Cr-8S-1** with *C*_2_ symmetry (Figure 7). The predicted Cr–Cr distance of 2.732 Å in **Cr-8S-1** with a WBI of 0.35 can be interpreted as a formal single bond, which is suggested by the HOMO–3 orbital. This Cr–Cr bond is bridged by two Me_2_C=N groups, forming the multicenter bond indicated by the ring critical point (Figure 7c), to give each chromium atom the favored 18-electron configuration. Structure **Cr-8S-1** is closely related to that of the iron complex (Me_2_C=N)_2_Fe_2_(CO)_6_ (**Fe-6S-1**) but with an additional CO group on each metal atom to compensate for the two fewer valence electrons of chromium relative to iron.

**Figure 7:**
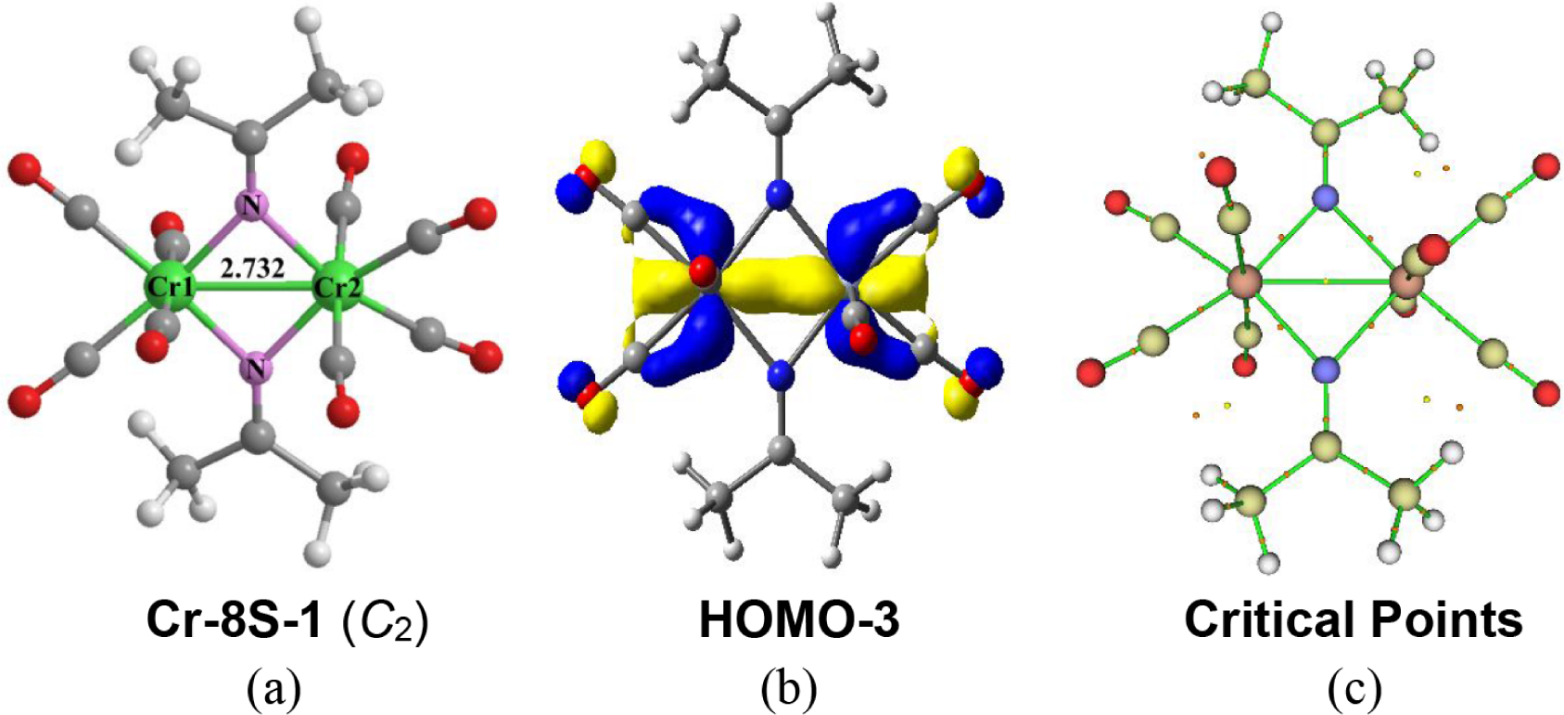
(a) The optimized (Me_2_C=N)_2_Cr_2_(CO)_8_ structure; (b) the selected bonding orbital and (c) critical point analysis.

#### 3.2.2 (Me_2_C=N)_2_Cr_2_(CO)_7_ Structures

Three low-energy structures were predicted for the heptacarbonyl (Me_2_C=N)_2_Cr_2_(CO)_7_ (Figure 8). The lowest energy structure **Cr-7S-1** could be obtained by removing one terminal CO group from one chromium atom in **Cr-8S-1** thereby shortening the Cr=Cr distance by ∼0.2 Å to 2.492 Å and increasing the WBI from 0.35 to 0.5. This Cr=Cr double bond can also be indicated by the frontier molecular orbitals. Thus, HOMO–4 and HOMO–3 correspond to the σ and π components, respectively, of the Cr=Cr double bond. The bridging Me_2_C=N groups in **Cr-8S-1** are not affected significantly by the loss of the CO group to give **Cr-7S-1.** Interpreting the Cr=Cr bond as a formal double bond in **Cr-7S-1** compensates for the loss of a CO group from **Cr-8S-1** so that each chromium atom retains the favored 18-electron configuration in **Cr-7S-1.** The triplet (Me_2_C=N)_2_Cr_2_(CO)_7_ structure **Cr-7T-2**, lying only 2.3 kcal/mol in energy above **Cr-7S-1** is very similar to **Cr-7S-1** except for the spin state. The predicted Cr=Cr distance of 2.503 Å with a WBI of 0.57 is similar to that of the singlet structure **Cr-7S-1** and thus can also correspond to a formal double bond. However, in order to account for the triplet spin state, the Cr=Cr double bond in **Cr-7T-2** must be of the σ + ^2^/_2_π type similar to the O=O bond in normal triplet dioxygen or the Fe=Fe bond in the organometallics (η^5^-R_5_C_5_)_2_Fe_2_(μ-CO)_3_ (R = H, Me)^22,23,24^ with an unpaired electron in each of the orthogonal π orbitals.

**Figure 8:**
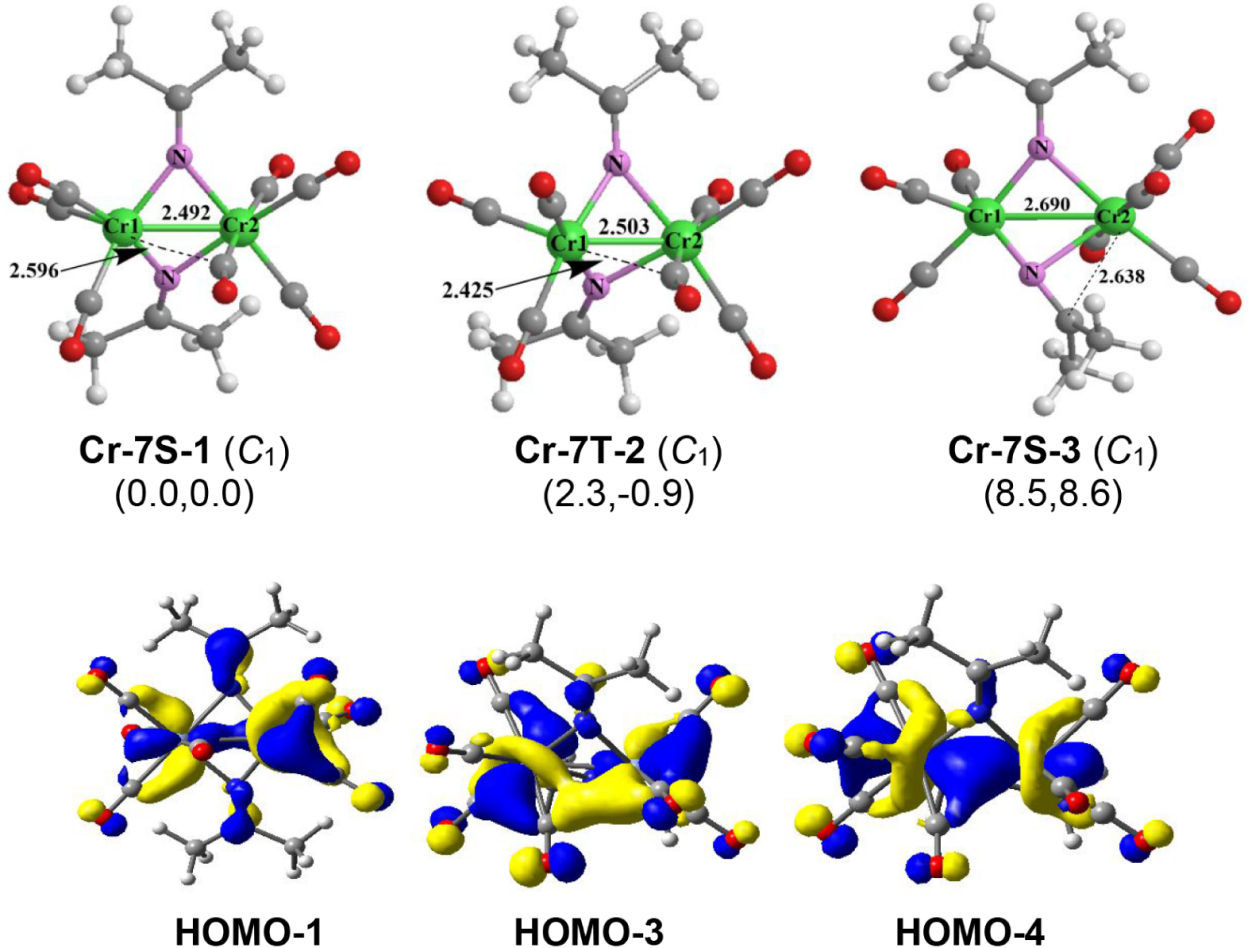
Optimized (Me_2_C=N)_2_Cr_2_(CO)_7_ structures and the selected frontier molecular orbitals for structure **Cr-7S-1**.

The third (Me_2_C=N)_2_Cr_2_(CO)_7_ structure **Cr-7S-3** is a singlet structure lying 8.5 kcal/mol in energy above **Cr-7S-1** (Figure 8). One of the bridging Me_2_C=N groups in **Cr-7S-3** is bent toward the Cr­(CO)_4_ chromium atom with a Cr–C distance of 2.638 Å implying coordination of the C=N double bond to that chromium atom. The Cr–Cr distance of 2.690 Å with a WBI of 0.32 in **Cr-7S-3** corresponds to a formal single bond. Placing a formal positive charge on the Cr­(CO)_4_ chromium atom and a balancing formal negative charge on the Cr­(CO)_3_ chromium atom gives each chromium atom in **Cr-7S-3** the favored 18-electron configuration.

#### 3.2.3 (Me_2_C=N)_2_Cr_2_(CO)_6_ Structures

Three low-energy structures were found for (Me_2_C=N)_2_Cr_2_(CO)_6_ (Figure 9). The lowest energy structure **Cr-6S-1** is a singlet with *C*_2_ symmetry with three terminal CO groups on each chromium atom and the two chromium atoms bridged by two Me_2_C=N ligands. The Cr–Cr distance of 2.593 Å with a WBI of 0.50 suggests a single bond formed by the d orbitals of the two chromium atoms, also the 3c-2e bonds were formed by the two Cr–N–Cr rings as indicted by the ring critical point, then each chromium obtains the 16-electron configuration (Figure 9).

**Figure 9:**
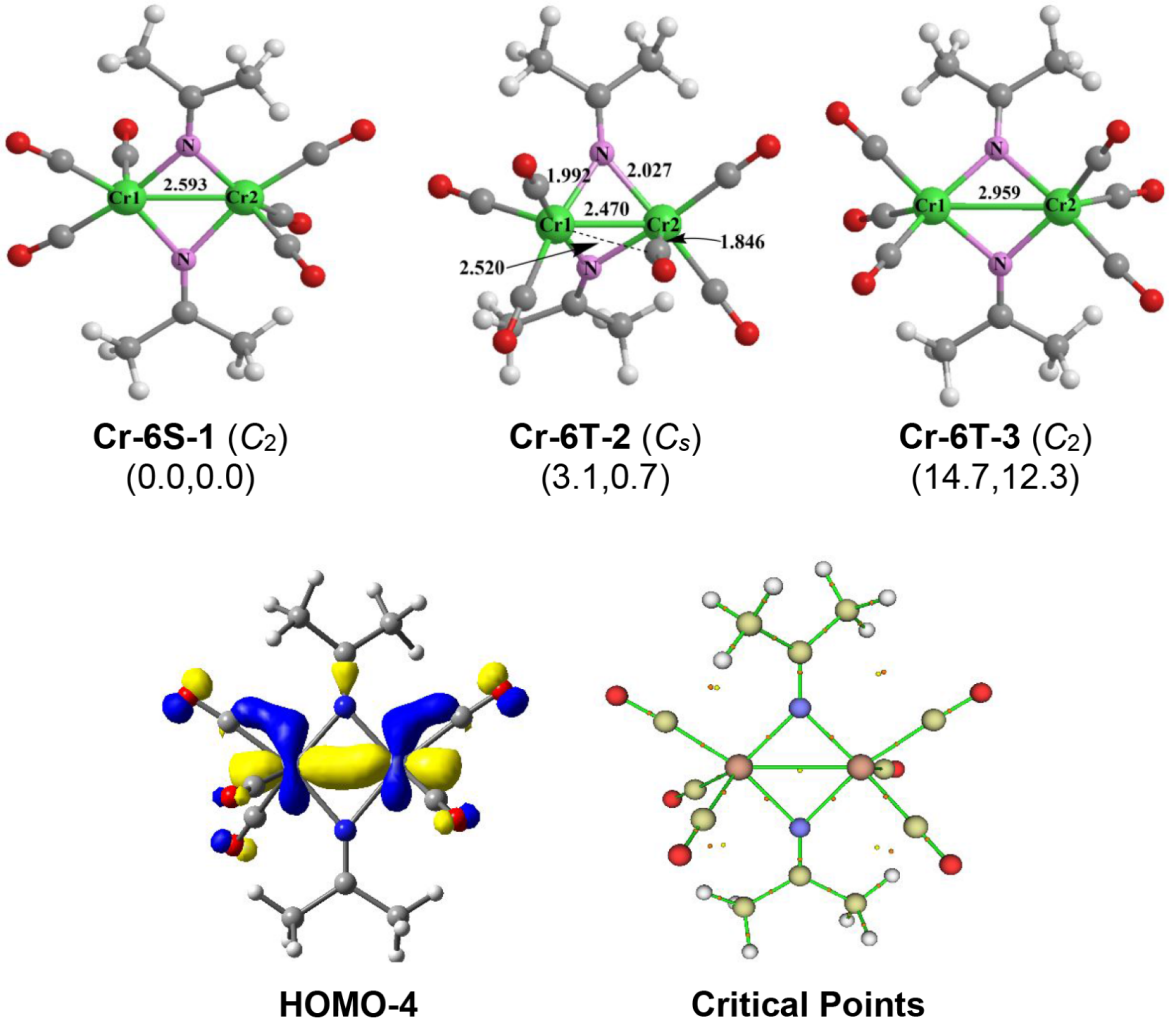
Optimized (Me_2_C=N)_2_Cr_2_(CO)_6_ structures, the selected molecular orbital, and the critical point analysis for structure **Cr-6S-1**.

The other two low-energy (Me_2_C=N)_2_Cr_2_(CO)_6_ structures are triplet spin state structures (Figure 9). Structure **Cr-6T-2**, lying 3.1 kcal/mol above **Cr-6S-1,** has a Cr=Cr distance of 2.470 Å with a WBI of 0.63 suggesting a Cr=Cr double bond. This Cr=Cr double bond bridged by the usual bridging Me_2_C=N group gives each chromium atom a 17-electron configuration consistent with a binuclear triplet. One of the CO groups in **Cr-6T-2** is a weakly semibridging CO group with a short Cr–C distance of 1.846 Å and a long Cr–C distance of 2.520 Å. This semibridging CO group has the effect of bending one of the Me_2_C=N bridges to make it unsymmetrical with a short Cr–N distance of 1.992 Å and a long Cr–N distance of 2.027 Å. The other (Me_2_C=N)_2_Cr_2_(CO)_6_ structure **Cr-6T-3**, lying 14.7 kcal/mol (M06-L) above **Cr-6S-1**, has a long Cr···Cr distance of 2.959 Å with a low WBI of 0.14 suggesting the lack of a formal chromium–chromium bond. The chromium–chromium distance is thus determined by the two bridging Me_2_C=N groups which are of the usual type. With this bonding interpretation, each chromium atom in **Cr-6T-3** has a 15-electron configuration consistent with a binuclear triplet.

#### 3.2.4 Carbonyl-Rich (Me_2_C=N)_2_Cr_2_(CO)_*n*_ (*N* = 10, 9) Structures

Only one low-energy (Me_2_C=N)_2_Cr_2_(CO)_10_ structure was found, namely the singlet **Cr-10S-1,** which was found to lie at least 17 kcal/mol below any of its isomers (Figure 10). In **Cr-10S-1** the two Me_2_C=N units couple to form a 2,3-diazabutadiene (acetone azine) Me_2_C=NN=CMe_2_ which then uses the lone pair on each of the two nitrogen atoms to coordinate to a Cr­(CO)_5_ moiety. The very long Cr···Cr distance of 4.499 Å with a near-zero WBI of 0.01 in **Cr-10S-1** clearly indicates the absence of any chromium–chromium bonding. Structure **Cr-10S-1** represents a likely intermediate in a synthesis of (Me_2_C=N)_2_Cr_2_(CO)_8_ **(Cr-8S-1**) from Me_2_C=NN=CMe_2_ and a suitably chosen chromium carbonyl derivative.

**Figure 10:**
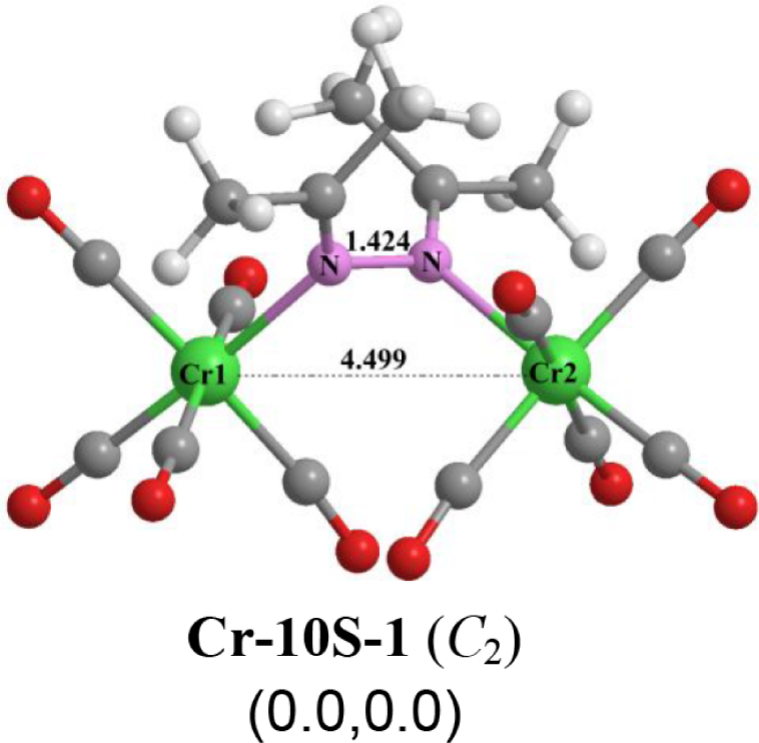
Optimized (Me_2_C=N)_2_Cr_2_(CO)_10_ structure.

The lowest energy (Me_2_C=N)_2_Cr_2_(CO)_9_ structure **Cr-9S-1** (Figure 11) can be obtained by removing one terminal carbonyl group from **Cr-10S-1** (Figure 10). In **Cr-9S-1**, the distance between the two chromium atoms at 4.427 Å (M06-L) is maintained upon loss of the CO group from **Cr-10S-1** suggesting absence of a direct metal–metal bond. Thus, the chromium atom losing the CO group in going from **Cr-10S-1** to **Cr-9S-1** goes from the favored 18-electron configuration to a 16-electron configuration.

**Figure 11:**
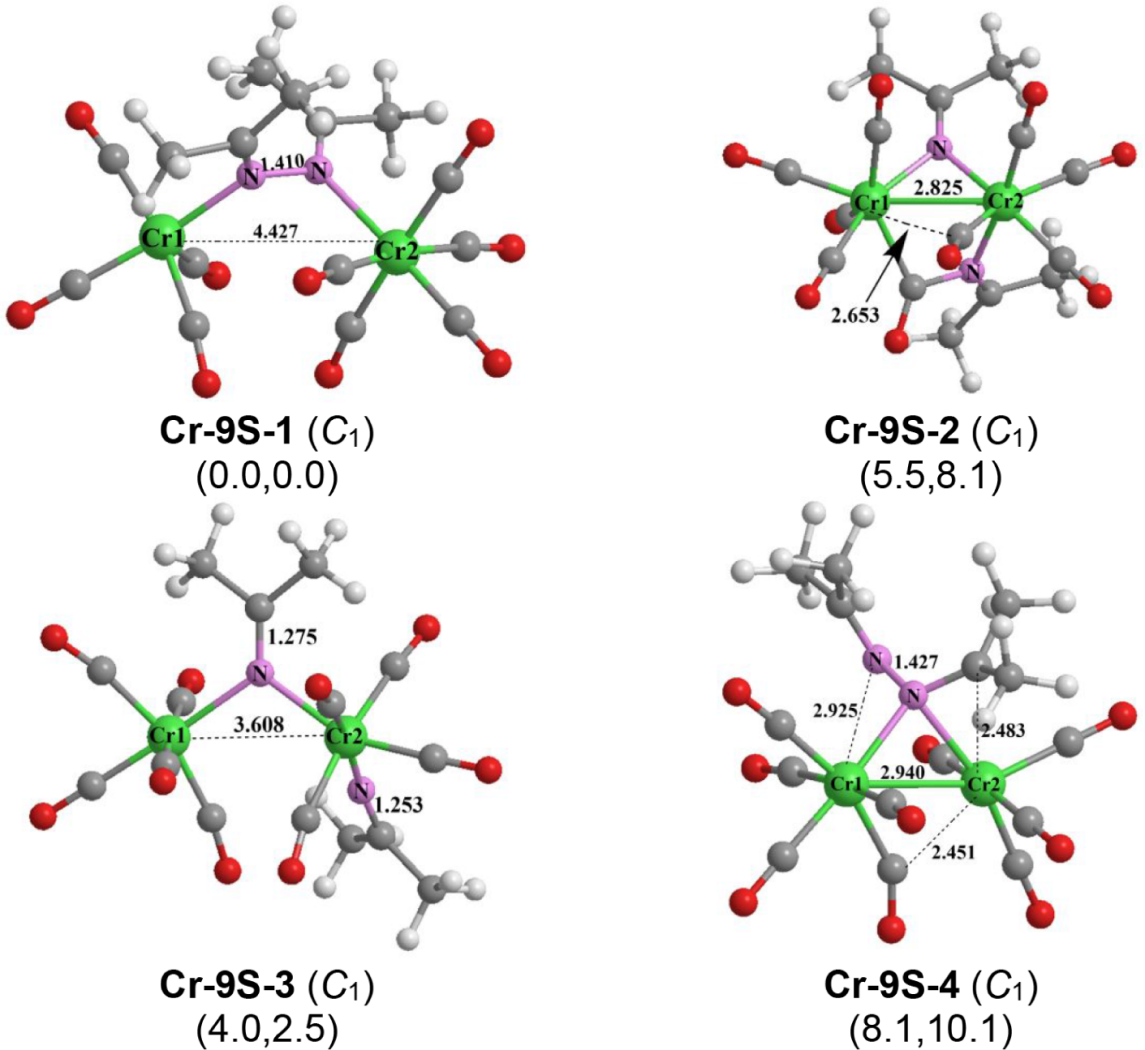
Optimized (Me_2_C=N)_2_Cr_2_(CO)_9_ structures.

The next (Me_2_C=N)_2_Cr_2_(CO)_9_ structure in terms of energy is the singlet **Cr-9S-2,** lying 5.5 kcal/mol above **Cr-9S-1**. Structure **Cr-9S-2** has a Cr–Cr distance of 2.825 Å with a WBI of 0.28 suggesting a formal single bond (Figure 11). Eight of the CO groups in **Cr-9S-2** are terminal CO groups whereas the ninth CO group inserts into a Cr–N bond to form a bridging Me_2_C=NC­(O) ligand with a relatively low ν­(CO) frequency of 1766 cm^–1^ similar to that found in the carbonyl-rich iron structures **Fe-8S-1**, **Fe-8S-2**, and **Fe-7S-1**. The combination of four terminal CO groups on each chromium atom, bridging Me_2_C=N and Me_2_C=NC­(O) groups, and a Cr–Cr single bond gives each chromium atom in **Cr-9S-2** the favored 18-electron configuration.

The singlet (Me_2_C=N)_2_Cr_2_(CO)_9_ structure **Cr-9S-3**, lying 4.0 kcal/mol above **Cr-9S-1**, has one chromium atom bonded to five terminal CO groups, and the other chromium atom bonded to four terminal CO groups and a terminal Me_2_C=N group (Figure 11). The pair of chromium atoms in **Cr-9S-3** is bridged by the other Me_2_C=N group. The long Cr···Cr distance of 3.608 Å with a low WBI of 0.06 indicates the lack of a chromium–chromium bond. The combination of bridging and terminal Me_2_C=N groups, each as three-electron donors, and the nine terminal CO groups gives each chromium atom in **Cr-9S-3** the favored 18-electron configuration, even in the absence of a chromium–chromium bond.

The fourth (Me_2_C=N)_2_Cr_2_(CO)_9_ structure **Cr-9S-4**, lying 8.1 kcal/mol in energy above **Cr-9S-1**, has the two Me_2_C=N units coupled to form a 2,3-diazabutadiene ligand similar to **Cr-9S-1** (Figure 11). However, one of the nitrogen atoms in the 2,3-diazabutadiene ligand in **Cr-9S-4** is not involved in the bonding to the chromium atoms. The Cr–Cr distance in **Cr-9S-4** of 2.940 Å with a WBI of 0.21 can be interpreted as a formal single bond. One chromium atom in **Cr-9S-4** bears five terminal CO groups whereas the other chromium atom bears only four terminal CO groups. The bridging 2,3-diazabutadiene ligand is bonded to the Cr­(CO)_5_ moiety only through one of its nitrogen atoms and to the Cr­(CO)_4_ moiety through its C=N double bond with a Cr–C distance of 2.483 Å. The 2,3-diazabutadiene ligand in **Cr-9S-4** is thus a four-electron donor, which, when combined with the nine terminal CO groups and the Cr–Cr single bond, gives each chromium atom the favored 18-electron configuration.

### 3.3 Thermochemistry

Tables 1 and 2 report the predicted energies for processes of the following two types considering the lowest energy singlet structures of the (Me_2_C=N)_2_M_2_(CO)_*n*_ (M = Fe or Cr) compounds:

**Table 1:** CO Dissociation Energies and Disproportionation Energies (in kcal/mol) for the (Me_2_C=N)_2_Fe_2_(CO)*_n_* (*N* = 8, 7, 6, 5, 4) Derivatives

	Δ*E*	Δ*G*
(Me_2_C=N)_2_Fe_2_(CO)_8_ (**Fe-8S-1**) → (Me_2_C=N)_2_Fe_2_(CO)_7_ (**Fe-7S-1**) + CO	2.0	–12.8
(Me_2_C=N)_2_Fe_2_(CO)_7_ (**Fe-7S-1**) → (Me_2_C=N)_2_Fe_2_(CO)_6_ (**Fe-6S-1**) + CO	–0.2	–13.9
(Me_2_C=N)_2_Fe_2_(CO)_6_ **(Fe-6S-1**) → (Me_2_C=N)_2_Fe_2_(CO)_5_ (**Fe-5S-1**) + CO	43.3	29.9
(Me_2_C=N)_2_Fe_2_(CO)_5_ (**Fe-5S-1**) → (Me_2_C=N)_2_Fe_2_(CO)_4_ (**Fe-4S-1**) + CO	36.1	24.1
2(Me_2_C=N)_2_Fe_2_(CO)_7_ **(Fe-7S-1)** → (Me_2_C=N)_2_Fe_2_(CO)_8_ (**Fe-8S-1**) + (Me_2_C=N)_2_Fe_2_(CO)_6_(**Fe-6S-1**)	–2.2	–1.0
2(Me_2_C=N)_2_Fe_2_(CO)_6_ (**Fe-6S-1**) → (Me_2_C=N)_2_Fe_2_(CO)_7_ (**Fe-7S-1**) + (Me_2_C=N)_2_Fe_2_(CO)_5_ (**Fe-5S-1**)	43.4	43.8
2(Me_2_C=N)_2_Fe_2_(CO)_5_ (**Fe-5S-1)** → (Me_2_C=N)_2_Fe_2_(CO)_6_ (**Fe-6S-1**) + (Me_2_C=N)_2_Fe_2_(CO)_4_ (**Fe-4S-1**)	–7.2	–5.8

**Table 2:** CO Dissociation Energies and Disproportionation Energies (in kcal/mol) for the (Me_2_C=N)_2_Cr_2_(CO)*_n_* (*N* = 10, 9, 8, 7, 6) Derivatives

	Δ*E*	Δ*G*
(Me_2_C=N)_2_Cr_2_(CO)_10_ (**Cr-10S-1**) → (Me_2_C=N)_2_Cr_2_(CO)_9_ (**Cr-9S-1**) + CO	33.0	18.7
(Me_2_C=N)_2_Cr_2_(CO)_9_ (**Cr**-**9S-1**) → (Me_2_C=N)_2_Cr_2_(CO)_8_ (**Cr-8S-1**) + CO	2.8	–9.5
(Me_2_C=N)_2_Cr_2_(CO)_8_ **(Cr-8S-1**) → (Me_2_C=N)_2_Cr_2_(CO)_7_ (**Cr-7S-1**) + CO	15.9	2.9
(Me_2_C=N)_2_Cr_2_(CO)_7_ **(Cr-7S-1**) → (Me_2_C=N)_2_Cr_2_(CO)_6_ **(Cr-6S-1**) + CO	33.3	20.3
2(Me_2_C=N)_2_Cr_2_(CO)_9_ (**Cr-9S-1**) → (Me_2_C=N)_2_Cr_2_(CO)_10_ (**Cr-10S-1**) + (Me_2_C=N)_2_Cr _2_(CO)_8_ (**Cr-8S-1**)	–30.2	–28.2
2(Me_2_C=N)_2_Cr_2_(CO)_8_ (**Cr-8S-1**) → (Me_2_C=N)_2_Cr_2_(CO)_9_ (**Cr-9S-1**) + (Me_2_C=N)_2_Cr_2_(CO)_7_ (**Cr-7S-1**)	13.1	12.4
2(Me_2_C=N)_2_Cr_2_(CO)_7_ (**Cr-7S-1**) → (Me_2_C=N)_2_Cr_2_(CO)_8_ (**Cr-8S-1**) + (Me_2_C=N)_2_Cr_2_(CO)_6_ (**Cr-6S-1**)	17.4	17.4

CO dissociation:

$${\left({\mathrm{Me}}_{2}C=N\right)}_{2}M_{2}{\left(\mathrm{CO}\right)}_{n}\to {\left({\mathrm{Me}}_{2}C=N\right)}_{2}M_{2}{\left(\mathrm{CO}\right)}_{n-1}+\left(\mathrm{CO}\right)$$

Disproportionation:

2(Me2C=N)2M2(CO)n→(Me2C=N)2M2(CO)n+1+(Me2C=N)2M2(CO)n−1

For the (Me_2_C=N)_2_Fe_2_(CO)_*n*_ (*n* = 6, 5) structures, the CO dissociation processes are seen to be endothermic by substantial amounts indicated by their positive Δ*G* values (Table 1). However, the CO dissociation processes for (Me_2_C=N)_2_Fe_2_(CO)_*n*_ (*n* = 8, 7) structures are exothermic. For the (Me_2_C=N)_2_Fe_2_(CO)_*n*_ structures, the CO dissociation energies and disproportionation energies for the experimentally known^11^ (Me_2_C=N)_2_Fe_2_(CO)_6_ structure **Fe-6S-1** were much more endothermic than those for the other (Me_2_C=N)_2_Fe_2_(CO)_*n*_ structures consistent with the stability of the hexacarbonyl and its experimental synthesis. Disproportionation of the carbonyl-rich (Me_2_C=N)_2_Fe_2_(CO)_7_ structure **Fe-7S-1** and the unsaturated (Me_2_C=N)_2_Fe_2_(CO)_5_ structure **Fe-5S-1** are clearly exothermic suggesting that these structures are not viable, also reflecting the stability of the known hexacarbonyl **Fe-6S-1** as an energy sink.

The CO dissociation processes for the (Me_2_C=N)_2_Cr_2_(CO)_*n*_ (*n* = 10, 8, 7) structures were predicted to be endothermic indicated by their positive Δ*G* values (Table 2). For (Me_2_C=N)_2_Cr_2_(CO)_9_ and (Me_2_C=N)_2_Fe_2_(CO)_8_, the CO dissociation processes were predicted to be exothermic by their negative Δ*G* values. Disproportionation of the (Me_2_C=N)_2_Cr_2_(CO)_9_ structure **Cr-9S-1** is the only such process among the chromium systems that is found to be exothermic and it is rather strongly exothermic at Δ*G* = −28.2 kcal/mol. However, the heptacarbonyl structure **Cr-7S-1** appears to be viable unlike the corresponding situation in the iron system.

## 4 Conclusion

This theoretical study reveals significant differences between the preferred structures for analogous (Me_2_C=N)_2_Fe_2_(CO)_*n*_ and (Me_2_C=N)_2_Cr_2_(CO)_*n*+2_ systems even though the extra carbonyl group for each chromium atom should compensate for the two fewer valence electrons of chromium relative to iron.

Analysis of the thermochemical data suggests that for the iron (Me_2_C=N)_2_Fe_2_(CO)_*n*_ systems the only viable structure is likely to be the hexacarbonyl. The preferred structure **Fe-6S-1** for the hexacarbonyl is of the type (Me_2_C=N)_2_Fe_2_(CO)_6_ in which an Fe–Fe single bond is bridged by two Me_2_C=N groups through only their nitrogen atoms. The preferred structure **Fe-4S-1** for the tetracarbonyl (Me_2_C=N)_2_Fe_2_(CO)_4_ has a formal Fe=Fe double bond bridged by two nonequivalent Me_2_C=N groups. One of these Me_2_C=N bridges bonds to the iron atoms solely through its nitrogen atoms whereas the other Me_2_C=N bridge uses its C=N double bond as well as its nitrogen atom to bridge the Fe=Fe bond. In all of these three preferred (Me_2_C=N)_2_Fe_2_(CO)_*n*_ structures the coordination number of each iron atom does not exceed six.

For the chromium systems (Me_2_C=N)_2_Cr_2_(CO)_*n*_ the decacarbonyl, octacarbonyl, heptacarbonyl, and hexacarbonyl all appear to be viable structures. The preferred structure **Cr-10S-1** for the carbonyl-rich decacarbonyl has an intact 2,3-diazabutadiene (acetone azine) Me_2_C=N–N=CMe_2_ ligand in which each nitrogen atom is coordinated to a Cr­(CO)_5_ moiety leading to an octahedral LCr­(CO)_5_ local environment. The preferred structure **Cr-8S-1** for the octacarbonyl (Me_2_C=N)_2_Cr_2_(CO)_8_ has a Cr–Cr bond bridged by two Me_2_C=N groups similar to the preferred structure **Fe-6S-1** for the iron derivative but with one more CO group per metal atom leading to heptacoordinate chromium counting the Cr–Cr bond. For the chromium systems the heptacarbonyl **Cr-7S-1** and hexacarbonyl **Cr-6S-1** both appear to be viable. These two structures retain the bridging Me_2_C=N groups of **Cr-8S-1** but have shorter chromium–chromium distances to compensate for carbonyl loss.

## Supplementary Material



## Notes

Figures S1 to S10: all optimized (Me_2_C=N)_2_Fe_2_(CO)_*n*_ (*n* = 8, 7, 6, 5, 4) and (Me_2_C=N)_2_Cr_2_(CO)_*n*_ (*n* = 10, 9, 8, 7, 6) structures; Tables S1 to S2: CO dissociation energies and disproportionation energies (in kcal/mol) for the (Me_2_C=N)_2_Fe_2_(CO)_*n*_ (*n* = 8, 7, 6, 5, 4) and (Me_2_C=N)_2_Cr_2_(CO)_*n*_ (*n* = 10, 9, 8, 7, 6) compounds; Tables S3 to S4: Wiberg bond indices (WBIs) and metal–metal bond distances (in Å) for the (Me_2_C=N)_2_Fe_2_(CO)_*n*_ (*n* = 8, 7, 6, 5, 4) and (Me_2_C=N)_2_Cr_2_(CO)_*n*_ (*n* = 10, 9, 8, 7, 6) structures predicted by the M06-L/DZP method; Tables S5 to S105: optimized coordinates of the (Me_2_C=N)_2_Fe_2_(CO)_*n*_ (*n* = 8, 7, 6, 5, 4) and (Me_2_C=N)_2_Cr_2_(CO)_*n*_ (*n* = 10, 9, 8, 7, 6) structures; Tables S106 to S206: harmonic vibrational frequencies (in cm^–1^) and infrared intensities (in parentheses, in km/mol) for (Me_2_C=N)_2_Fe_2_(CO)_*n*_ (*n* = 8, 7, 6, 5, 4) and (Me_2_C=N)_2_Cr_2_(CO)_*n*_ (*n* = 10, 9, 8, 7, 6) structures; complete Gaussian16 reference (reference 15) (PDF)
