# Supplementary material for: Binuclear Acetoniminato Derivatives of Iron and Chromium Carbonyls: A Theoretical Study
Source: ACS Omega. 2026 Feb 26;11(9):15538–47. doi: 10.1021/acsomega.6c00171 (PMC12980407; doi:10.1021/acsomega.6c00171)
Supplement: Supplementary file 1 [file ao6c00171_si_001.pdf]

Binuclear Acetoniminato Derivatives of Iron and Chromium Carbonyls: A Theoretical Study

Haoyu Chen ,[a] Jinfeng Luo , [a] Yongtao Liu, [a] Huidong Li, [a,b] *
Qunchao Fan,[a*] Zhixiang Fan, [a] R. Bruce King,[b] *
and Henry F. Schaefer, III[b]

[a] *School of Science, Key Laboratory of High Performance Scientific Computation, Xihua University, Chengdu, China 610039*
[b]*Centre for Computational Quantum Chemistry, University of Georgia, Athens, Georgia, USA 30602*
rbking@chem.uga.edu; huidongli@mail.xhu.edu.cn; fanqunchao@sina.com;

Supporting Information

.

Figures S1 to S10: All optimized $(Me_2C{=}N)_2Fe_2(CO)_n$ (n = 8, 7, 6, 5, 4) and $(Me_2C{=}N)_2Cr_2(CO)_n$ (n = 10, 9, 8, 7, 6) structures

Tables S1 to S2: CO Dissociation energies and disproportionation energies (in kcal/mol) for the $(Me_2C{=}N)_2Fe_2(CO)_n$ (n = 8, 7, 6, 5, 4) and $(Me_2C{=}N)_2Cr_2(CO)_n$ (n = 10, 9, 8, 7, 6) compounds

Tables S3 to S4: Wiberg bond indices (WBIs) and metal-metal bond distances (in Å) for the $(Me_2C{=}N)_2Fe_2(CO)_n$ (n = 8, 7, 6, 5, 4) and $(Me_2C{=}N)_2Cr_2(CO)_n$ (n = 10, 9, 8, 7, 6) structures predicted by the M06-L/DZP method

Tables S5 to S105: Optimized coordinates of the $(Me_2C{=}N)_2Fe_2(CO)_n$ (n = 8, 7, 6, 5, 4) and $(Me_2C{=}N)_2Cr_2(CO)_n$ (n = 10, 9, 8, 7, 6) structures

Tables S106 to S206: Harmonic vibrational frequencies (in cm^{-1}) and infrared intensities (in parentheses, in km/mol) for $(Me_2C{=}N)_2Fe_2(CO)_n$ (n = 8, 7, 6, 5, 4) and $(Me_2C{=}N)_2Cr_2(CO)_n$ (n = 10, 9, 8, 7, 6) structures

Complete Gaussian16 reference (Reference15).

Figure S1. Optimized $(Me_2C{=}N)_2Fe_2(CO)_8$ structures. In Figure S1 to S10, the numbers in the parentheses are the relative energies (in kcal/mol) calculated by M06-L and B3PW91-D3 methods.The numbers in red parentheses are the relative energies (in kcal/mol) calculated by M06-L/def2TZVP-deltE and M06-L/def2TZVP-deltG.

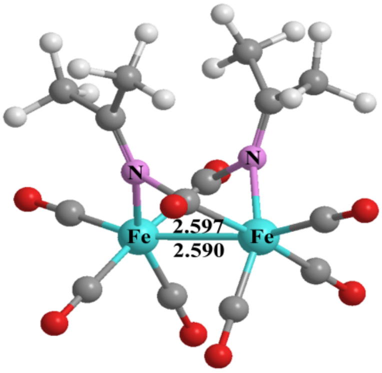<br>Fe-8S-1(C_2)<br>(0.0,0.0)<br>(0.0,0.0)	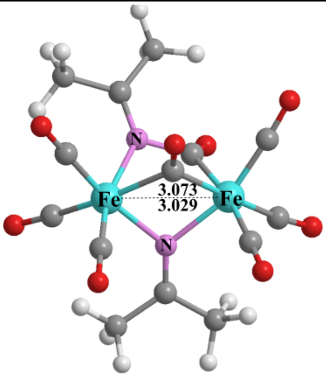<br>Fe-8S-2(C_1)<br>(6.9,10.4)<br>(6.5,4.8)	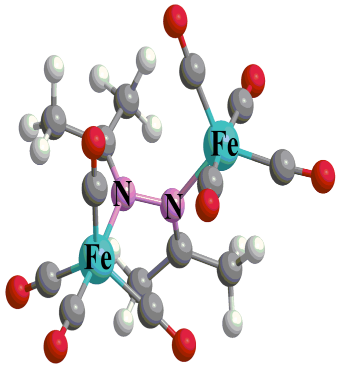<br>Fe-8S-3(C_1)<br>(14.9,12.5)<br>(11.2,7.6)
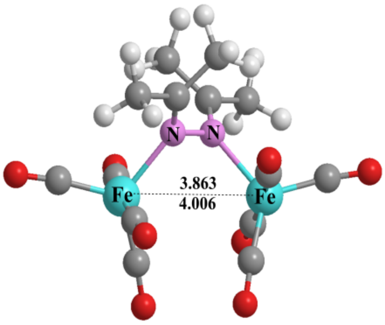<br>Fe-8S-4(C_2)<br>(19.7,17.8)	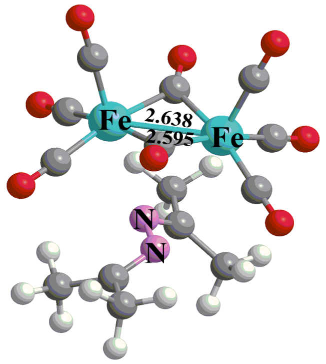<br>Fe-8T-5(C_1)<br>(23.6,32.0)	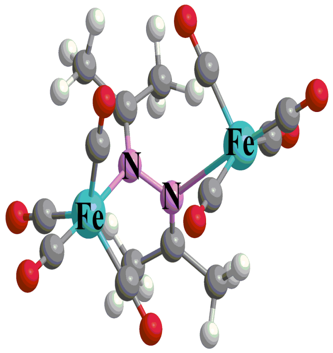<br>Fe-8T-6(C_1)<br>(27.0,37.2)
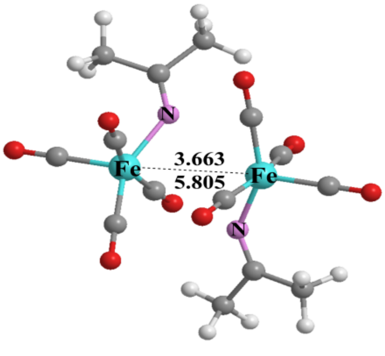<br>Fe-8T-7(C_1)<br>(36.8,47.9)	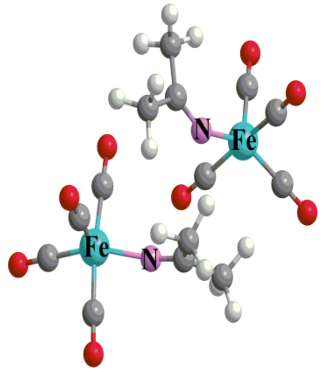<br>Fe-8T-8(C_1)<br>(39.1,47.2)	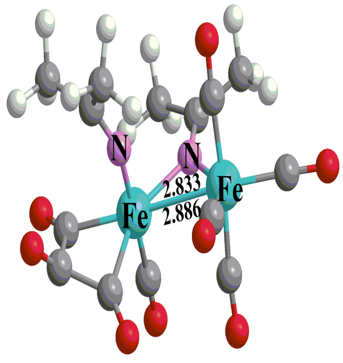<br>Fe-8T-9(C_1)<br>(60.4,66.3)

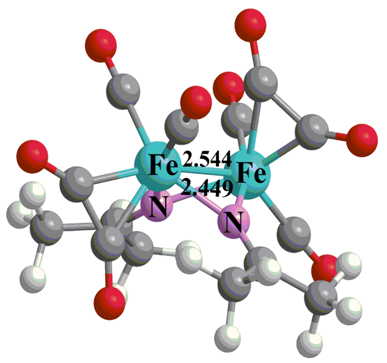

Fe-8S-10(C_1)
(79.9,52.8)

Figure S2. The optimized $(Me_2C{=}N)_2Cr_2(CO)_{10}$ structures.

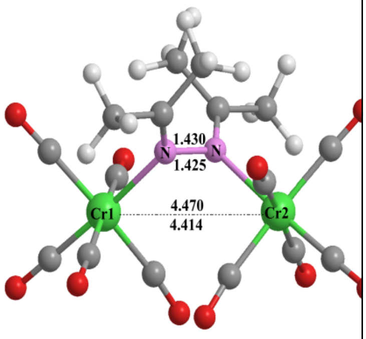

Cr-10S-1 (C_2)
(0.0,0.0)
(0.0,0.0)

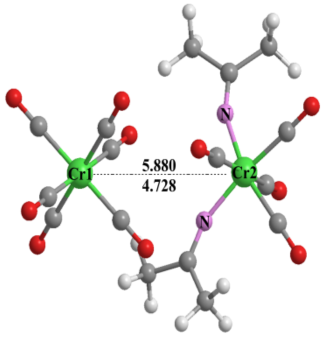

Cr-10
S-2 (C_1)
(18.3,17.2)

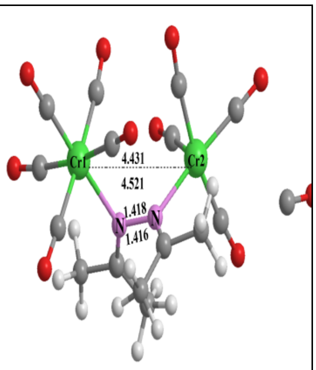

Cr-10S-3 (C_1)
(31.5,28.3)

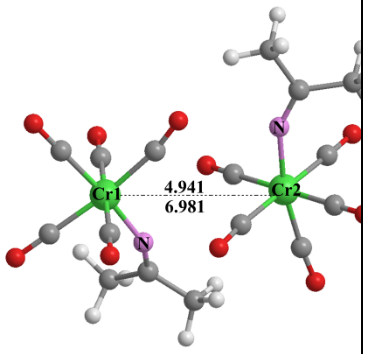

Cr-10S-4 (C_1)
(44.7,44.7)

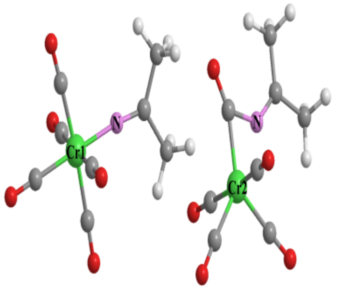

Cr-10S-5 (C_1)
(56.3,49.9)

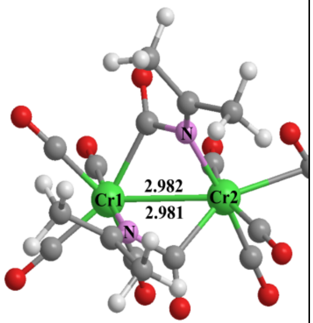

Cr-10S-6 (C_1)
(70.5,74.8)

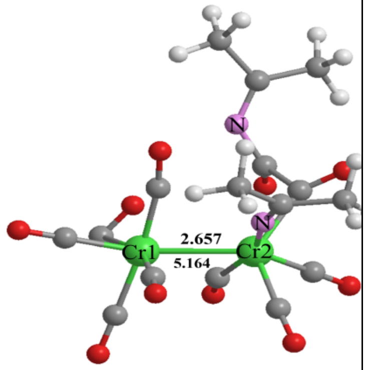

Cr-10S-7 (C_1)
(80.7,59.0)

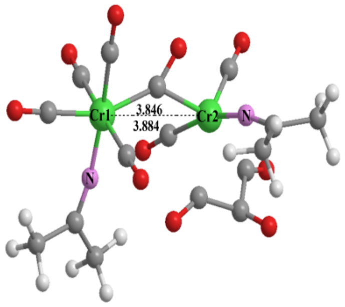

Cr-10S-8 (C_1)
(94.5,94.1)

Figure S3. The optimized $(Me_2C{=}N)_2Fe_2(CO)_7$ structures.

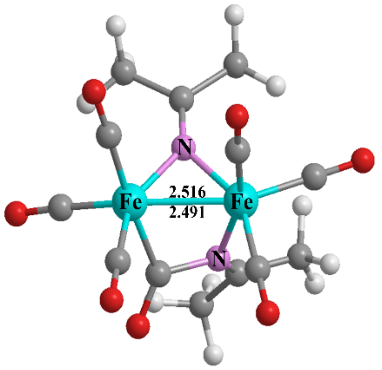

Fe-7S-1(C_1)
(0.0,0.0)
(0.0,0.0)

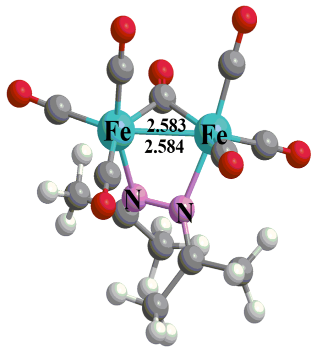

Fe-7S-2(C_1)
(24.6,25.4)

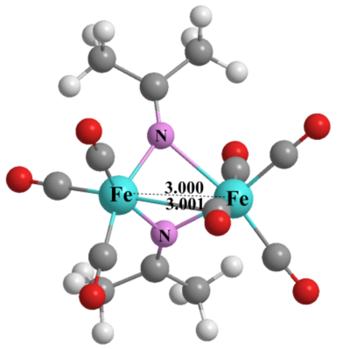

Fe-7T-3(C_1)
(28.6,29.9)

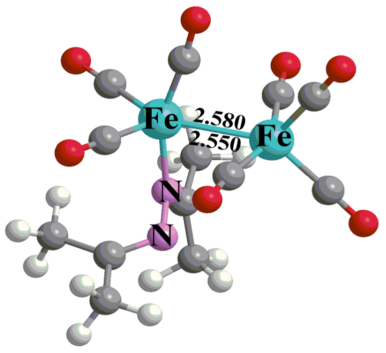

Fe-7T-4(C_1)
(31.9,30.3)

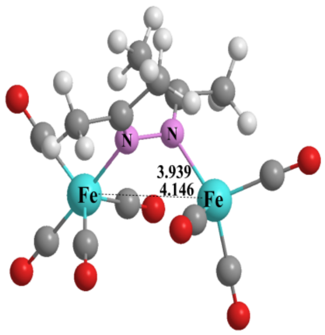

Fe-7T-5(C_1)
(33.3,30.0)

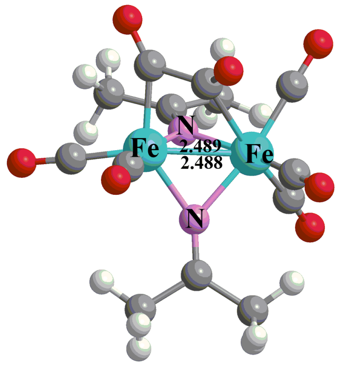

Fe-7S-6(C_1)
(34.9,39.0)

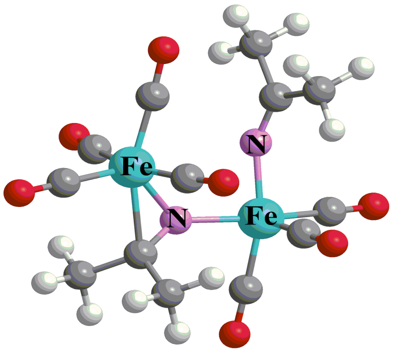

Fe-7S-7(C_1)
(44.3,47.4)

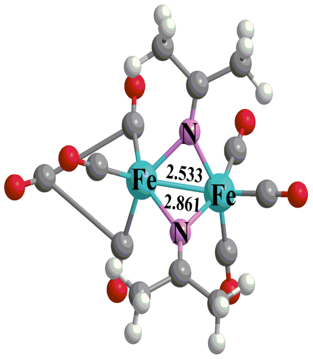

Fe-7T-8(C_1)
(44.7,26.4)

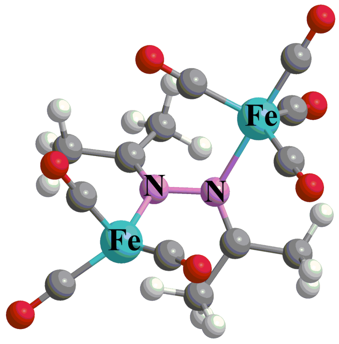

Fe-7S-9(C_1)
(55.5,52.1)

Figure S4. The optimized $(Me_2C{=}N)_2Cr_2(CO)_9$ structures.

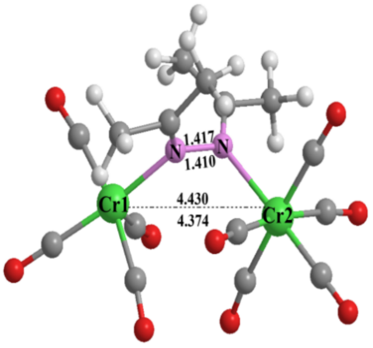

Cr-9S-1 (C_1)
(0.0,0.0)
(0.0,0.0)

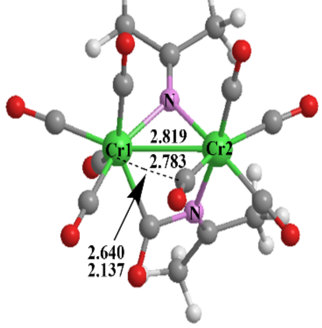

Cr-9S-2 (C_1)
(2.9,7.1)
(5.5,8.1)

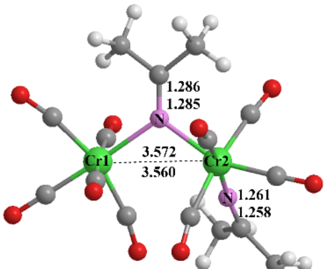

Cr-9S-3 (C_1)
(6.1,11.8)
(4.0,2.5)

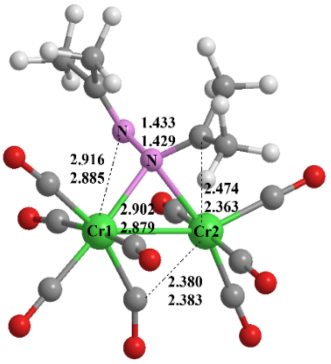

Cr-9S-4 (C_1)
(6.5,11.0)
(8.1,10.1)

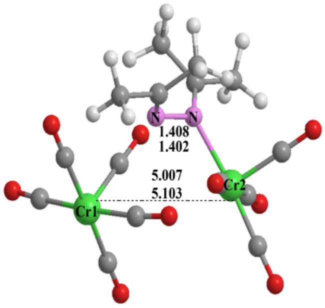

Cr-9S-5 (C_1)
(17.4,22.5)

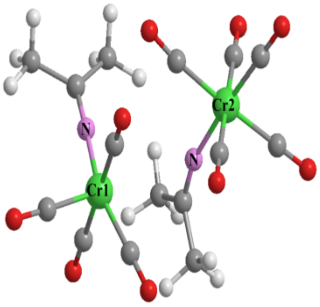

Cr-9T-6 (C_1)
(19.7,22.2)

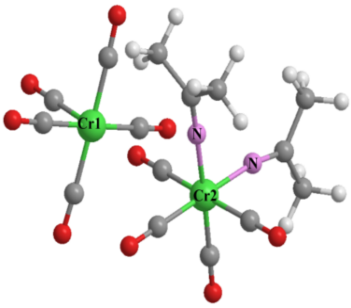

Cr-9T-7 (C_1)
(24.7,28.3)

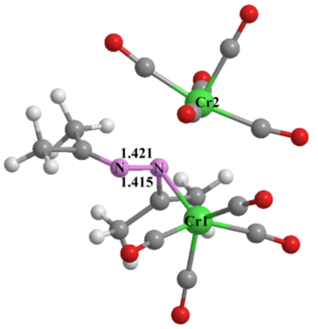

Cr-9T-8 (C_1)
(30.9,32.4)

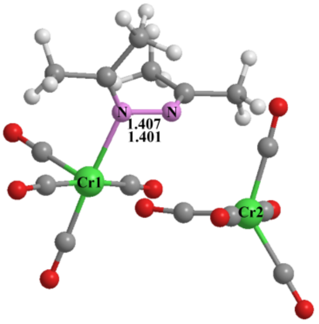

Cr-9T-9 (C_1)
(31.2,26.4)

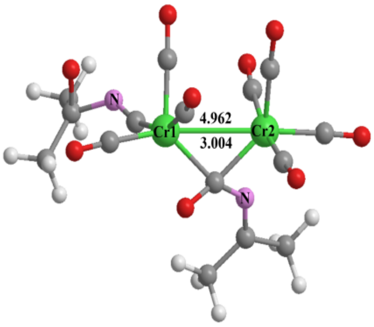

Cr-9S-10 (C_1)
(33.3,84.5)

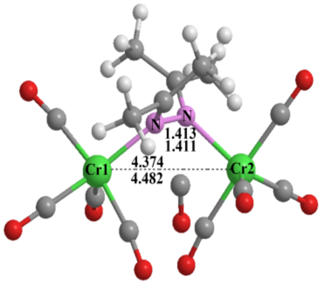

Cr-9T-11 (C_1)
(47.5,45.5)

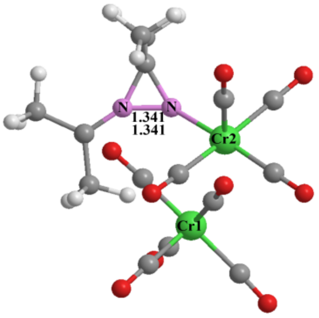

Cr-9S-12 (C_1)
(47.7,42.5)

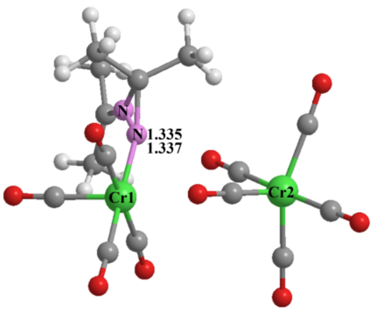<br>Cr-9T-13 (C_1)<br>(57.2,55.5)	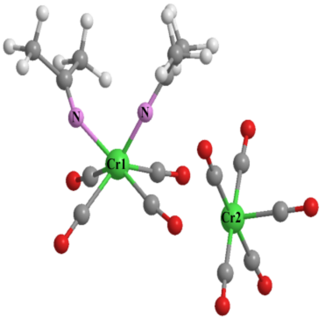<br>Cr-9S-14 (C_1)<br>(90.9,26.0)	

Figure S5. The optimized $(Me_2C{=}N)_2Fe_2(CO)_6$ structures.

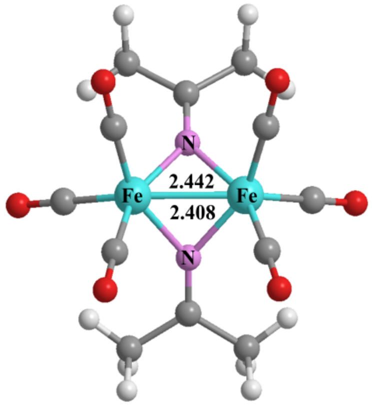

Fe-6S-1(C_{2v})
(0.0,0.0)
(0.0,0.0)

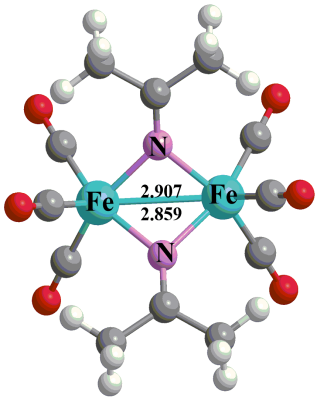

Fe-6T-2(C_{2v})
(23.9,18.0)

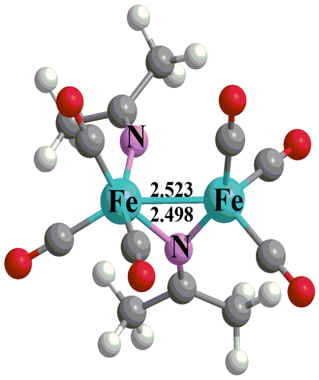

Fe-6T-3(C_1)
(37.4,35.3)

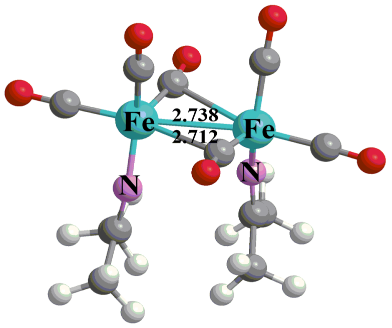

Fe-6S-4(C_1)
(44.4,48.4)

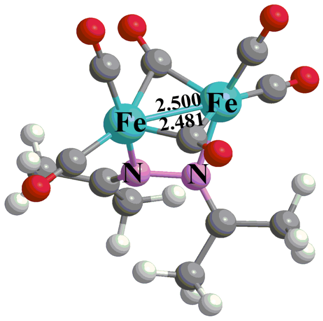

Fe-6S-5(C_2)
(51.4,53.5)

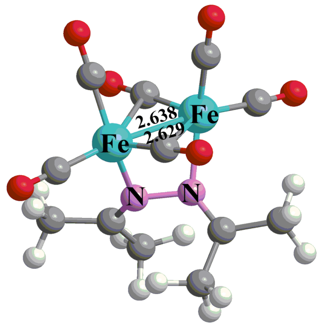

Fe-6T-6(C_2)
(52.6,49.1)

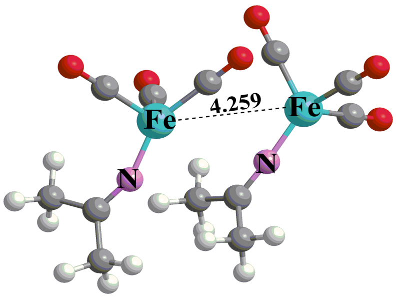

Fe-6T-7(C_1)
(59.4,64.4)

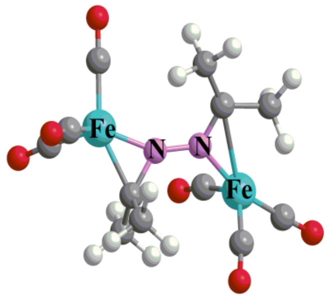

Fe-6S-8(C_1)
(81.0,74.1)

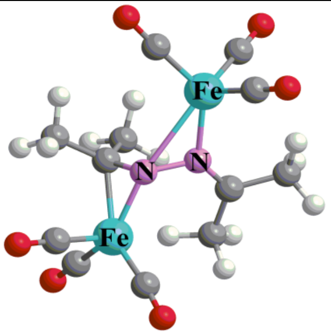

Fe-6T-9(C_1)
(83.6,79.3)

Figure S6. The optimized $(Me_2C{=}N)_2Cr_2(CO)_8$ structures.

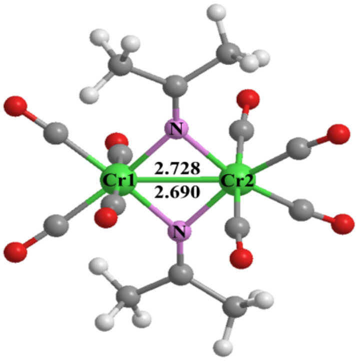

Cr-8S-1 (C_2)
(0.0,0.0)
(0.0,0.0)

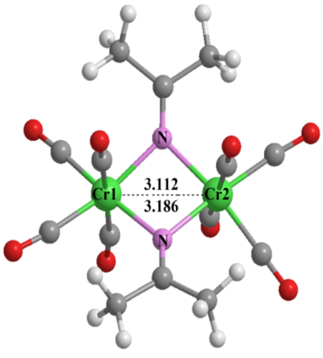

Cr-8T-2 (C_2)
(16.6,8.6)

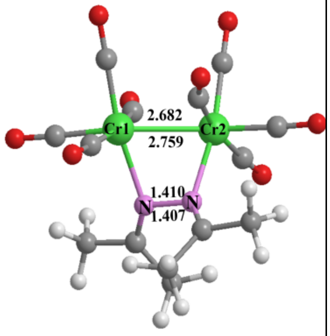

Cr-8S-3 (C_2)
(19.3,9.7)

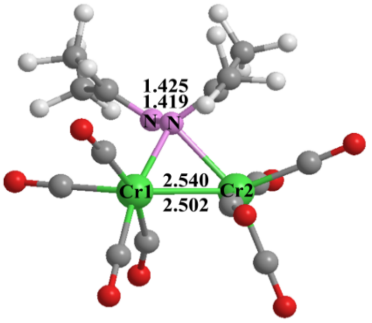

Cr-8S-4 (C_1)
(20.0,13.9)

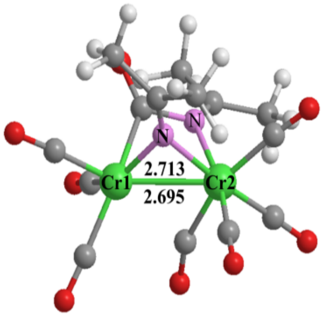

Cr-8S-5 (C_1)
(25.5,19.3)

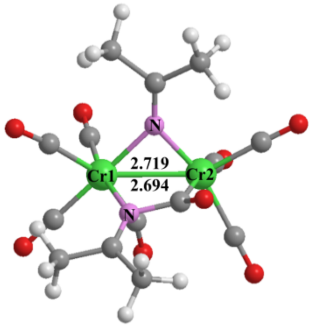

Cr-8T-6 (C_1)
(28.6,22.2)

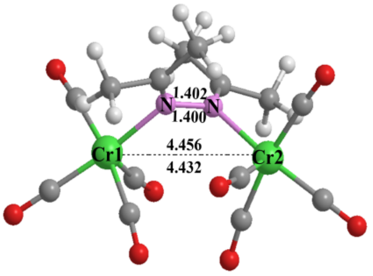

Cr-8S-7 (C_1)
(29.4,19.1)

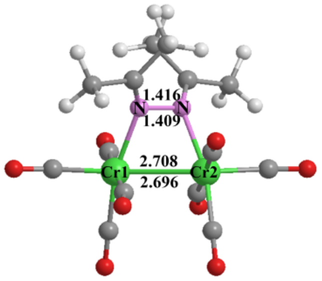

Cr-8T-8 (C_1)
(32.6,27.3)

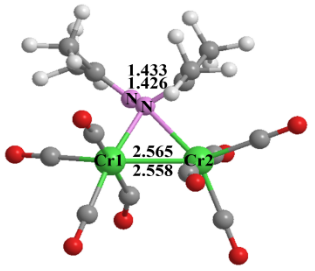

Cr-8T-9 (C_1)
(39.8,33.8)

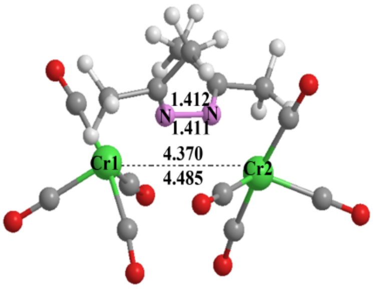

Cr-8T-10 (C_1)
(46.1,38.4)

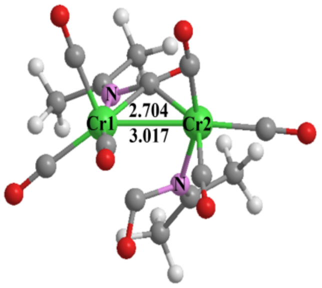

Cr-8T-11 (C_1)
(48.5,29.5)

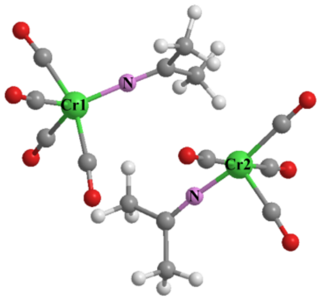

Cr-8S-12 (C_1)
(55.1,48.6)

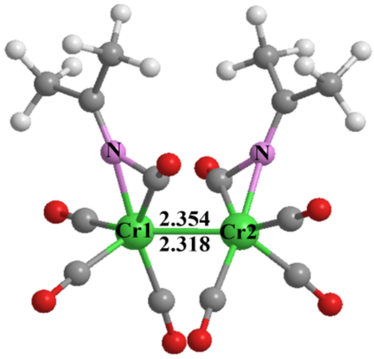<br>Cr-8S-13 (C_1)<br>(55.9,46.5)		

Figure S7. The optimized $(Me_2C{=}N)_2Fe_2(CO)_5$ structures.

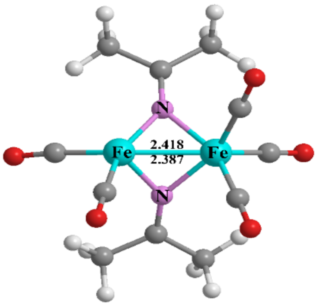<br>Fe-5S-1(C_1)<br>(0.0,0.0)<br>(0.0,0.0)	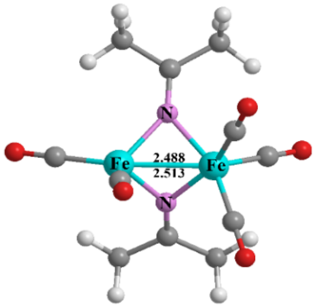<br>Fe-5T-2(C_1)<br>(2.0,-1.3)<br>(2.3,-0.8)	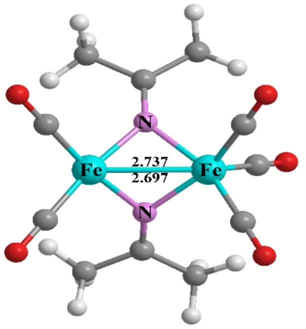<br>Fe-5T-3(C_S)<br>(3.2,-0.7)<br>(2.0,1.2)
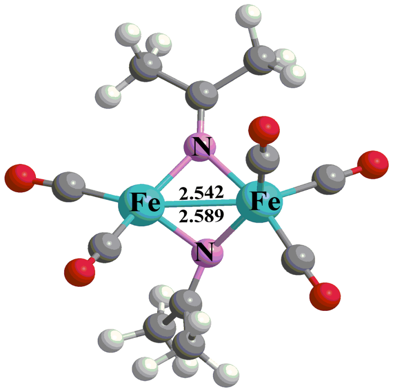<br>Fe-5T-4(C_1)<br>(19.4,17.8)	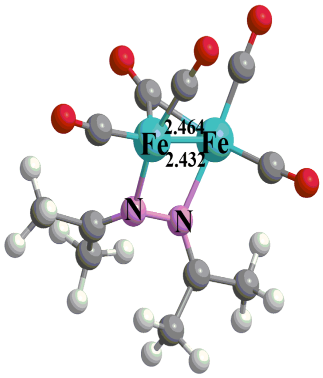<br>Fe-5S-5(C_1)<br>(35.4,57.8)	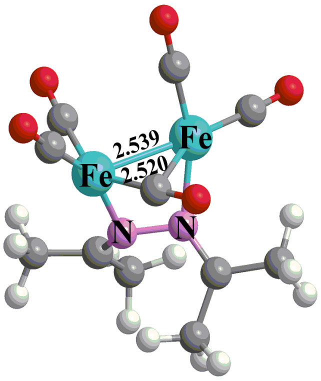<br>Fe-5T-6(C_1)<br>(44.8,36.4)
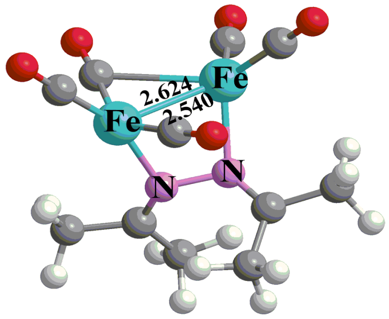<br>Fe-5T-7(C_1)<br>(55.6,66.8)	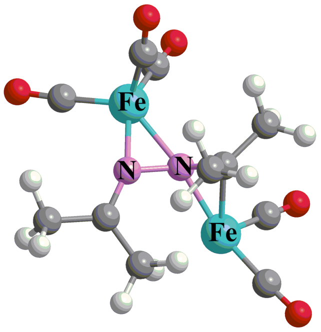<br>Fe-5S-8(C_1)<br>(67.6,65.9)	

Figure S8. The optimized $(Me_2C{=}N)_2Cr_2(CO)_7$ structures.

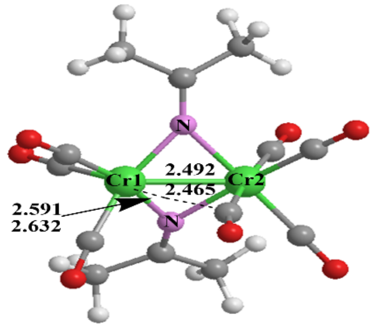

Cr-7S-1 (C_1)
(0.0,0.0)
(0.0,0.0)

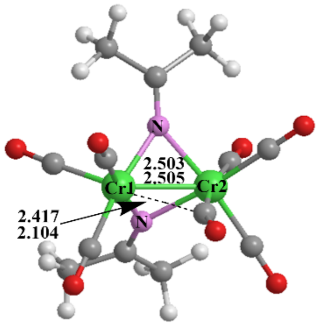

Cr-7T-2 (C_1)
(2.7,0.6)
(2.3,-0.9)

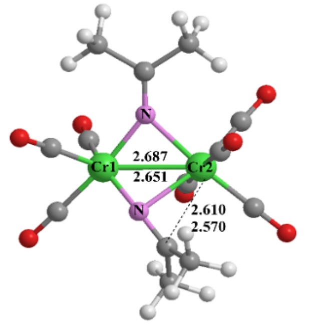

Cr-7S-3 (C_1)
(8.6,6.0)
(8.5,8.6)

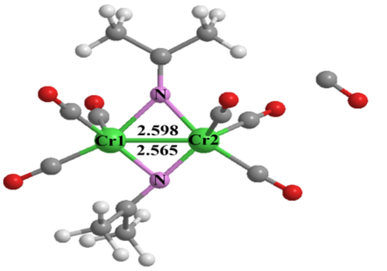

Cr-7S-4 (C_1)
(27.4,22.7)

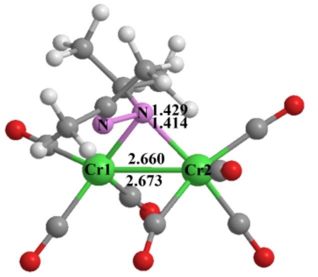

Cr-7S-5 (C_1)
(28.7,21.7)

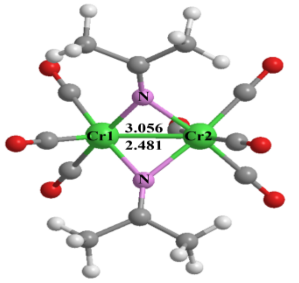

Cr-7T-6 (C_1)
(32.5,40.3)

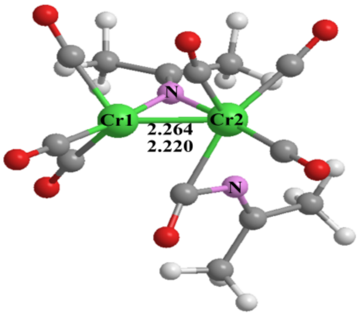

Cr-7S-7 (C_1)
(35.3,31.7)

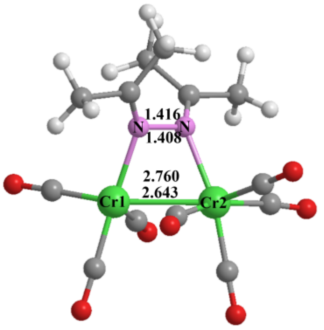

Cr-7S-8 (C_1)
(35.9,32.5)

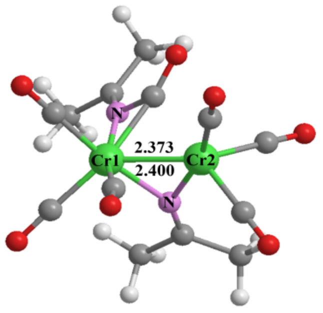

Cr-7T-9 (C_1)
(36.0,29.1)

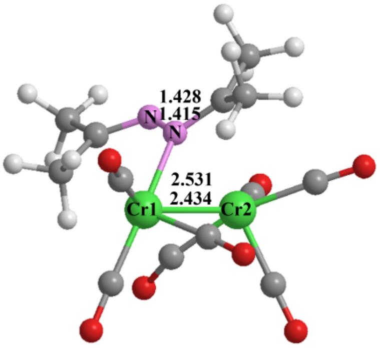

Cr-7S-10 (C_1)
(36.3,35.5)

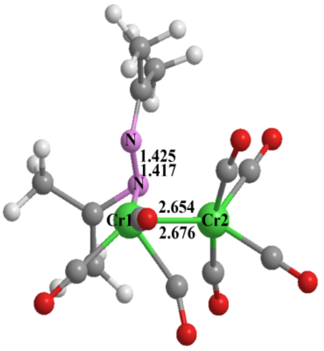

Cr-7T-11 (C_1)
(41.9,36.5)

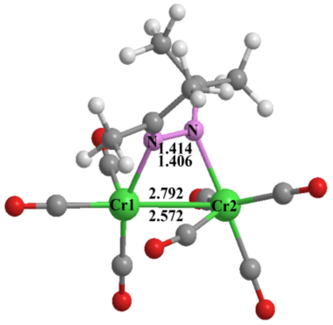

Cr-7T-12 (C_1)
(47.8,38.2)

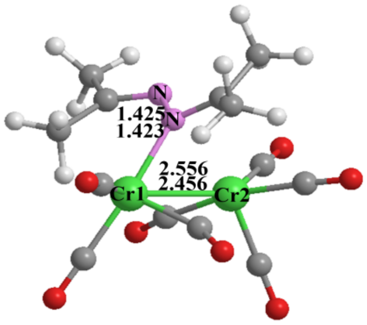<br>Cr-7T-13 (C_1)<br>(50.9,48.8)		

Figure S9. The optimized $(Me_2C{=}N)_2Fe_2(CO)_4$ structures.

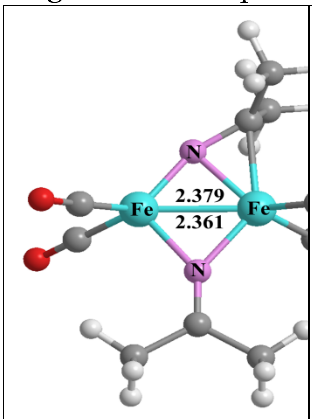 Fe-4S-1(C_s)<br>(0.0,0.0)<br>(0.0,0.0)	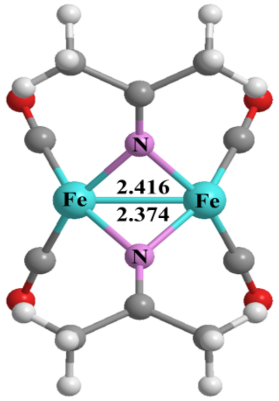 Fe-4S-2(C_{2v})<br>(7.6,7.0)<br>(7.4,7.3)	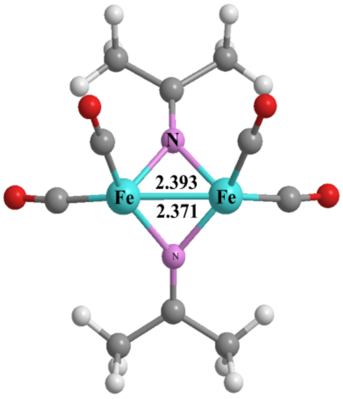 Fe-4S-3(C_s)<br>(7.8,4.8)<br>(8.2,7.1)
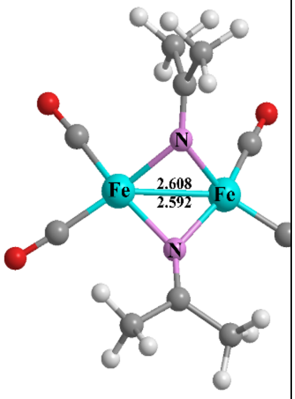 Fe-4T-4(C_1)<br>(8.6,3.0)<br>(8.2,6.3)	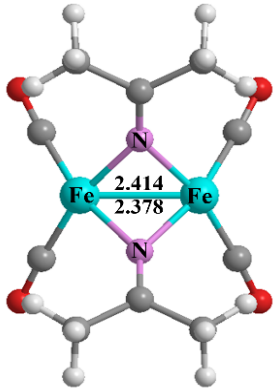 Fe-4T-5(C_{2v})<br>(10.6,6.2)<br>(10.6,9.3)	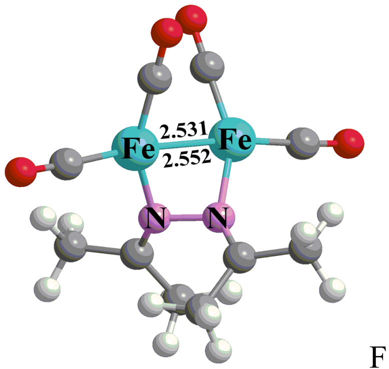 F<br>e-4T-6(C_1)<br>(38.9,32.7)
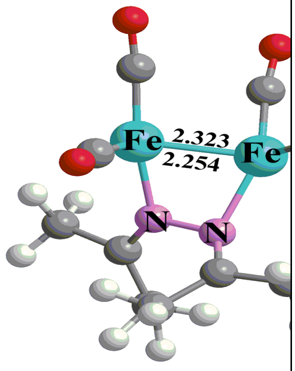 Fe-4S-7(C_1)<br>(68.4,70.0)	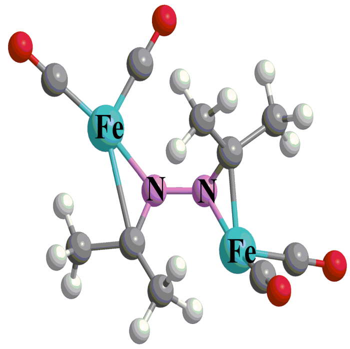 Fe<br>-4S-8(C_1)<br>(82.5,77.0)	

Figure S10. The optimized $(Me_2C{=}N)_2Cr_2(CO)_6$ structures.

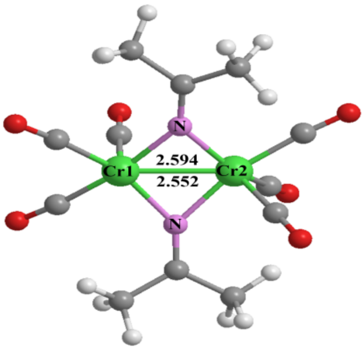

Cr-6S-1 (C_2)
(0.0,0.0)
(0.0,0.0)

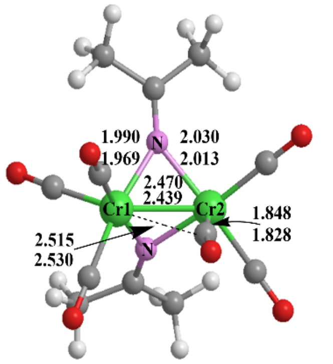

Cr-6T-2 (C_s)
(4.1,5.8)
(3.1,0.7)

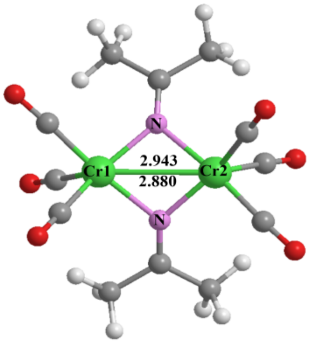

Cr-6T-3 (C_2)
(15.9,11.5)
(14.7,12.3)

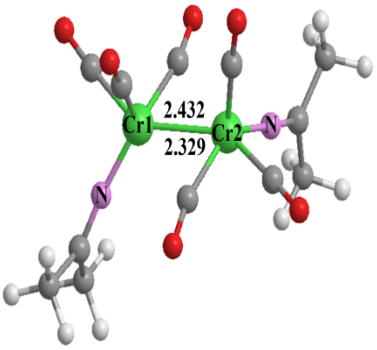

Cr-6S-4 (C_1)
(20.6,22.6)

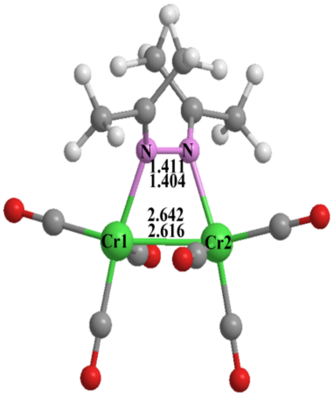

Cr-6S-5 (C_2)
(37.8,44.5)

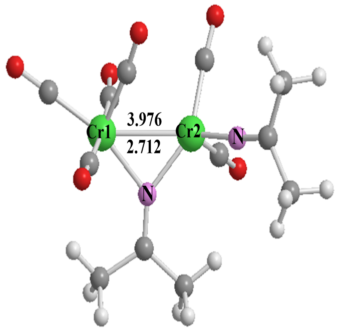

Cr-6S-6 (C_1)
(39.5,14.1)

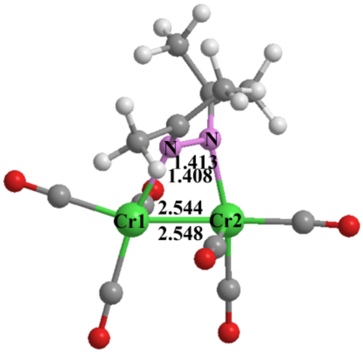

Cr-6T-7 (C_2)
(44.1,51.3)

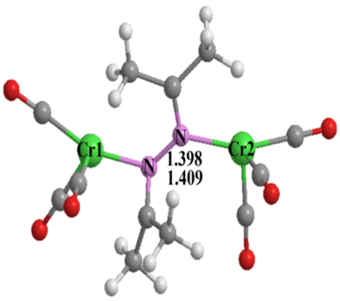

Cr-6S-8 (C_1)
(56.4,42.3)

Table S1. CO Dissociation energies and disproportionation energies (in kcal/mol) for the $(Me_2C=N)_2Fe_2(CO)_n$ (n=8,7,6,5,4) derivatives.

	M06-L	B3PW9-D3
$(Me_2C=N)_2Fe_2(CO)_8$(**Fe-8S-1**)→$(Me_2C=N)_2Fe_2(CO)_7$(**Fe-7S-1**) +(CO)	30.0	10.2
$(Me_2C=N)_2Fe_2(CO)_7$(**Fe-7S-1**)→$(Me_2C=N)_2Fe_2(CO)_6$(**Fe-6S-1**) + (CO)	19.4	9.0
$(Me_2C=N)_2Fe_2(CO)_6$(**Fe-6S-1**)→$(Me_2C=N)_2Fe_2(CO)_6$(**Fe-5S-1**) + (CO)	59.9	45.4
$(Me_2C=N)_2Fe_2(CO)_5$(**Fe-5S-1**)→$(Me_2C=N)_2Fe_2(CO)_4$(**Fe-4S-1**) + (CO)	53.3	41.9
2$(Me_2C=N)_2Fe_2(CO)_7$(**Fe-7S-1**)→$(Me_2C=N)_2Fe_2(CO)_8$(**Fe-8S-1**) +$(Me_2C=N)_2Fe_2(CO)_6$(**Fe-6S-1**)	-1.6	-1.2
2$(Me_2C=N)_2Fe_2(CO)_6$(**Fe-6S-1**)→$(Me_2C=N)_2Fe_2(CO)_7$(**Fe-7S-1**) +$(Me_2C=N)_2Fe_2(CO)_5$(**Fe-5S-1**)	40.5	36.5
2$(Me_2C=N)_2Fe_2(CO)_5$(**Fe-5S-1**)→$(Me_2C=N)_2Fe_2(CO)_6$(**Fe-6S-1**) + $(Me_2C=N)_2Fe_2(CO)_4$(**Fe-4S-1**)	-6.7	-3.6

Table S2. CO Dissociation energies and disproportionation energies (in kcal/mol) for the $(Me_2C=N)_2Cr_2(CO)_n$ (n=10,9,8,7,6) derivatives.

	M06-L	B3PW9-D3
$(Me_2C=N)_2Cr_2(CO)_{10}$(**Cr-10S-1**)→$(Me_2C=N)_2Cr_2(CO)_9$(**Cr-9S-1**) + (CO)	49.2	35.6
$(Me_2C=N)_2Cr_2(CO)_9$(**Cr-9S-1**)→$(Me_2C=N)_2Cr_2(CO)_8$(**Cr-8S-1**)+ (CO)	19.1	16.8
$(Me_2C=N)_2Cr_2(CO)_8$(**Cr-8S-1**)→$(Me_2C=N)_2Cr_2(CO)_7$(**Cr-7S-1**)+ (CO)	33.3	18.7
$(Me_2C=N)_2Cr_2(CO)_7$(**Cr-7S-1**)→$(Me_2C=N)_2Cr_2(CO)_6$(**Cr-6S-1**)+ (CO)	49.8	26.5
2$(Me_2C=N)_2Cr_2(CO)_9$(**Cr-9S-1**)→$(Me_2C=N)_2Cr_2(CO)_{10}$(**Cr-10S-1**)+ $(Me_2C=N)_2Cr_2(CO)_8$(**Cr-8S-1**)	-30.2	18.8
2$(Me_2C=N)_2Cr_2(CO)_8$(**Cr-8S-1**)→$(Me_2C=N)_2Cr_2(CO)_{10}$(**Cr-10S-1**)+ $(Me_2C=N)_2Cr_2(CO)_6$(**Cr-6S-1**)	14.8	7.2
2$(Me_2C=N)_2Cr_2(CO)_7$(**Cr-7S-1**)→$(Me_2C=N)_2Cr_2(CO)_8$(**Cr-8S-1**) + $(Me_2C=N)_2Cr_2(CO)_6$(**Cr-6S-1**)	16.5	21.4

Table S3. Wiberg bond indices (WBIs) and the Fe-Fe bond distances (in Å) for the $(Me_2C{=}N)_2Fe_2(CO)_n$ structures (n=8,7,6,5,4) predicted by the M06-L method.

	Wiberg bond index	Fe-Fe distance(Å)
$(Me_2C{=}N)_2Fe_2(CO)_8$**(Fe-8S-1)**	0.39	2.597
$(Me_2C{=}N)_2Fe_2(CO)_8$**(Fe-8S-2)**	0.11	3.073
$(Me_2C{=}N)_2Fe_2(CO)_8$**(Fe-8S-3)**	0.01	4.212
$(Me_2C{=}N)_2Fe_2(CO)_8$**(Fe-8S-4)**	0.02	3.863
$(Me_2C{=}N)_2Fe_2(CO)_7$**(Fe-7S-1)**	0.41	2.516
$(Me_2C{=}N)_2Fe_2(CO)_6$**(Fe-6S-1)**	0.44	2.442
$(Me_2C{=}N)_2Fe_2(CO)_5$**(Fe-5S-1)**	0.46	2.418
$(Me_2C{=}N)_2Fe_2(CO)_5$**(Fe-5T-2)**	0.38	2.488
$(Me_2C{=}N)_2Fe_2(CO)_5$**(Fe-5T-3)**	0.15	2.737
$(Me_2C{=}N)_2Fe_2(CO)_5$**(Fe-5T-4)**	0.35	2.542
$(Me_2C{=}N)_2Fe_2(CO)_4$**(Fe-4S-1)**	0.51	2.379
$(Me_2C{=}N)_2Fe_2(CO)_4$**(Fe-4S-2)**	0.82	2.416
$(Me_2C{=}N)_2Fe_2(CO)_4$**(Fe-4S-3)**	0.53	2.393
$(Me_2C{=}N)_2Fe_2(CO)_4$**(Fe-4T-4)**	0.20	2.608

Table S4. Wiberg bond indices (WBIs) and the Cr-Cr bond distances (in Å) for the $(Me_2C{=}N)_2Cr_2(CO)_n$ structures (n=10,9,8,7,6) predicted by the M06-L method.

	Wiberg bond index	Cr-Cr distance(Å)
$(Me_2C{=}N)_2Cr_2(CO)_{10}$**(Cr-10S-1)**	0.01	4.470
$(Me_2C{=}N)_2Cr_2(CO)_{10}$**(Cr-10S-2)**	0.00	5.880
$(Me_2C{=}N)_2Cr_2(CO)_9$**(Cr-9S-1)**	0.01	4.430
$(Me_2C{=}N)_2Cr_2(CO)_9$**(Cr-9S-2)**	0.28	2.819
$(Me_2C{=}N)_2Cr_2(CO)_9$**(Cr-9S-3)**	0.06	3.572
$(Me_2C{=}N)_2Cr_2(CO)_9$**(Cr-9S-4)**	0.21	2.902
$(Me_2C{=}N)_2Cr_2(CO)_9$**(Cr-9S-5)**	0.05	5.007
$(Me_2C{=}N)_2Cr_2(CO)_9$**(Cr-9T-6)**	0.01	4.477
$(Me_2C{=}N)_2Cr_2(CO)_8$**(Cr-8S-1)**	0.35	2.728
$(Me_2C{=}N)_2Cr_2(CO)_8$**(Cr-8T-2)**	0.12	3.112
$(Me_2C{=}N)_2Cr_2(CO)_8$**(Cr-8S-3)**	0.35	2.682
$(Me_2C{=}N)_2Cr_2(CO)_7$**(Cr-7S-1)**	0.53	2.492
$(Me_2C{=}N)_2Cr_2(CO)_7$**(Cr-7T-2)**	0.57	2.503
$(Me_2C{=}N)_2Cr_2(CO)_7$**(Cr-7S-3)**	0.32	2.687
$(Me_2C{=}N)_2Cr_2(CO)_6$**(Cr-6S-1)**	0.50	2.594
$(Me_2C{=}N)_2Cr_2(CO)_6$**(Cr-6T-2)**	0.63	2.470
$(Me_2C{=}N)_2Cr_2(CO)_6$**(Cr-6T-3)**	0.14	2.943

Table S5. Optimized coordinates for the $(Me_2C{=}N)_2Fe_2(CO)_8$ structure **8S-1.**

	M06-L				B3PW91-D3		
	x	y	z		x	y	z
C	-1.417697	1.791107	-1.566429	C	-1.385401	1.820196	-1.561710
C	0.617926	2.943082	-0.164160	C	0.632003	2.914566	-0.153734
C	-1.095435	-1.119656	-2.057923	C	-1.095656	-1.142288	-2.039062
C	-0.617926	-2.943082	-0.164160	C	-0.632003	-2.914566	-0.153734
O	-2.337478	2.123712	-2.183715	O	-2.283628	2.190021	-2.180285
O	1.039056	3.995909	0.084942	O	1.068483	3.955329	0.093799
O	-1.775179	-1.027274	-2.993396	O	-1.774979	-1.066317	-2.970146
O	-1.039056	-3.995909	0.084942	O	-1.068483	-3.955329	0.093799
C	2.125494	-0.352956	3.157400	C	2.062713	-0.327582	3.159742
C	0.617926	-2.361898	2.686249	C	0.658207	-2.401637	2.640146
H	1.020104	-3.242953	2.175983	H	0.975172	-3.240008	2.010699
H	0.761142	-2.491365	3.761132	H	0.928919	-2.613101	3.678256
H	2.960578	-0.981902	3.484317	H	2.938128	-0.911136	3.473630
H	1.536503	-0.136740	4.057301	H	1.443317	-0.181039	4.054807
C	1.095435	1.119656	-2.057923	C	1.095656	1.142288	-2.039062
C	-1.611996	-0.472667	0.374852	C	-1.582179	-0.489729	0.367459
O	1.775179	1.027274	-2.993396	O	1.774979	1.066317	-2.970146
O	-2.794291	-0.748636	0.428587	O	-2.759218	-0.786668	0.446630
H	2.535753	0.564779	2.739280	H	2.412226	0.629697	2.773657
H	-0.455585	-2.336781	2.468984	H	-0.433511	-2.334586	2.562614
C	-0.617926	2.361898	2.686249	C	-0.658207	2.401637	2.640146
H	0.455585	2.336781	2.468984	H	0.433511	2.334586	2.562614
H	-1.020104	3.242953	2.175983	H	-0.975172	3.240008	2.010699
H	-0.761142	2.491365	3.761132	H	-0.928919	2.613101	3.678256
C	-2.125494	0.352956	3.157400	C	-2.062713	0.327582	3.159742
H	-1.536503	0.136740	4.057301	H	-1.443317	0.181039	4.054807
H	-2.960578	0.981902	3.484317	H	-2.938128	0.911136	3.473630
H	-2.535753	-0.564779	2.739280	H	-2.412226	-0.629697	2.773657
Fe	-0.075244	-1.296486	-0.607709	Fe	-0.075673	-1.292921	-0.600921
Fe	0.075244	1.296486	-0.607709	Fe	0.075673	1.292921	-0.600921
C	-1.287228	1.118786	2.184515	C	-1.282871	1.123998	2.153912
C	1.287228	-1.118786	2.184515	C	1.282871	-1.123998	2.153912
N	-1.097075	0.755763	0.962153	N	-1.095656	0.758080	0.935685
N	1.097075	-0.755763	0.962153	N	1.095656	-0.758080	0.935685
O	2.794291	0.748636	0.428587	O	2.759218	0.786668	0.446630
C	1.611996	0.472667	0.374852	C	1.582179	0.489729	0.367459
C	1.417697	-1.791107	-1.566429	C	1.385401	-1.820196	-1.561710
O	2.337478	-2.123712	-2.183715	O	2.283628	-2.190021	-2.180285

Table S6. Optimized coordinates for the $(Me_2C{=}N)_2Fe_2(CO)_8$ structure **8S-2.**

	M06-L				B3PW91-D3		
	x	y	z		x	y	z
C	2.968691	0.564411	-0.910350	C	2.928921	0.646664	-0.879469
C	2.013227	1.896069	1.120573	C	1.878579	1.952993	1.092119
C	-2.438742	-1.179436	-1.194340	C	-2.361429	-1.221109	-1.197847
C	-1.110752	-2.141766	1.134692	C	-1.049931	-2.148467	1.125081
O	3.878829	0.785173	-1.589007	O	3.839445	0.893633	-1.539000
O	2.231795	2.898456	1.660353	O	2.024979	2.975147	1.607624
O	-3.237824	-1.533050	-1.956005	O	-3.124343	-1.548756	-1.998184
O	-0.938667	-3.133311	1.713137	O	-0.860364	-3.121397	1.717617
C	-1.311575	3.691114	-1.098563	C	-1.384407	3.623766	-1.118255
C	-3.265829	2.182882	-0.591502	C	-3.320322	2.092719	-0.570246
H	-3.539226	2.517676	0.415789	H	-3.581345	2.431844	0.440937
H	-3.784903	2.843563	-1.292014	H	-3.851466	2.741840	-1.275764
H	-1.969522	4.419320	-0.615291	H	-2.051385	4.347496	-0.636345
H	-1.366515	3.861062	-2.178307	H	-1.449220	3.770604	-2.203287
C	2.400978	-0.815678	1.397121	C	2.393990	-0.703025	1.419222
C	-2.680269	-0.140818	1.253262	C	-2.634822	-0.206884	1.257018
O	2.889757	-1.600767	2.093466	O	2.907233	-1.438597	2.142866
O	-3.514787	0.136936	2.008561	O	-3.460398	0.042197	2.022519
H	-0.277542	3.864693	-0.801118	H	-0.351088	3.816486	-0.824817
H	-3.623316	1.161425	-0.725733	H	-3.665240	1.065419	-0.695710
C	2.290977	-2.232741	-1.800310	C	2.377098	-2.175699	-1.760149
H	2.498760	-1.491155	-2.578370	H	2.565981	-1.445704	-2.556060
H	3.005648	-2.065193	-0.988821	H	3.070216	-1.966257	-0.939500
H	2.473530	-3.222907	-2.225080	H	2.593970	-3.172248	-2.157398
C	-0.034214	-3.217965	-1.742014	C	0.068295	-3.211662	-1.750259
H	0.393051	-4.174942	-1.422038	H	0.495518	-4.158205	-1.394399
H	-1.040511	-3.136829	-1.332688	H	-0.955178	-3.136285	-1.383557
H	-0.096385	-3.255361	-2.835798	H	0.054714	-3.257306	-2.847339
Fe	-1.321252	-0.644807	0.200142	Fe	-1.282572	-0.667930	0.191651
Fe	1.574424	0.380697	0.281475	Fe	1.536025	0.414700	0.280376
C	0.869106	-2.095948	-1.309867	C	0.940192	-2.067526	-1.292227
C	-1.789662	2.304028	-0.788012	C	-1.841588	2.228154	-0.782464
N	0.490623	-1.110570	-0.587061	N	0.527461	-1.097013	-0.571574
N	-1.017845	1.270638	-0.670273	N	-1.053600	1.212799	-0.660711
O	-0.216475	0.627956	2.651633	O	-0.221256	0.573464	2.638157
C	-0.016079	0.258561	1.538053	C	-0.021612	0.233752	1.511251
C	0.421440	1.427672	-0.973710	C	0.377261	1.375813	-0.984042
O	0.723778	1.996448	-2.001069	O	0.659247	1.901665	-2.040586

Table S7. Optimized coordinates for the $(Me_2C{=}N)_2Fe_2(CO)_8$ structure **8S-3.**

	M06-L				B3PW91-D3		
	x	y	z		x	y	z
C	-3.213989	1.100854	-0.274086	C	-3.210385	1.067331	-0.258423
C	-1.368527	-1.057300	-1.544241	C	-1.386994	-1.065483	-1.522956
C	3.256014	0.878586	0.123860	C	3.231049	0.883537	0.168773
C	1.996902	-0.370218	1.983686	C	1.957179	-0.374299	1.966389
O	-3.879943	2.049221	-0.413515	O	-3.909851	1.984249	-0.402437
O	-0.923640	-1.531647	-2.511518	O	-0.980864	-1.556293	-2.492378
O	4.023766	1.749013	0.051876	O	4.016273	1.734213	0.137241
O	2.003982	-0.328300	3.150600	O	1.971263	-0.353886	3.128487
C	1.581847	1.062486	-2.806999	C	1.576489	0.999889	-2.836017
C	-0.601764	2.246326	-2.211024	C	-0.612569	2.185442	-2.243512
H	-0.345348	2.807394	-3.110497	H	-0.414437	2.633253	-3.219737
H	-1.493564	1.640779	-2.420155	H	-1.533181	1.589162	-2.297574
H	2.220427	1.944795	-2.934195	H	2.157864	1.909740	-3.036466
H	1.125970	0.856988	-3.780938	H	1.100068	0.702519	-3.778515
C	-3.516121	-1.425154	-0.246194	C	-3.474304	-1.423353	-0.182163
C	3.223988	-1.444225	-0.743014	C	3.218839	-1.395360	-0.711957
O	-4.425405	-2.137647	-0.359144	O	-4.376819	-2.141638	-0.262850
O	4.031512	-2.097548	-1.275728	O	4.038006	-2.036949	-1.232134
H	2.201221	0.217185	-2.501450	H	2.244873	0.208625	-2.493699
H	-0.882955	2.941554	-1.414919	H	-0.795348	2.978047	-1.510341
C	-1.425512	2.158870	2.145069	C	-1.456319	2.228068	2.071074
H	-2.219735	1.414804	2.112878	H	-2.178402	1.415422	2.149122
H	-0.946675	2.142302	3.130971	H	-0.980773	2.402412	3.043766
H	-1.864240	3.155666	2.024229	H	-1.991818	3.147155	1.797957
C	0.817567	2.814728	1.175694	C	0.811081	2.837131	1.120542
H	1.571082	2.341456	1.817906	H	1.532430	2.379625	1.810597
H	1.285202	2.991976	0.205472	H	1.312193	2.966507	0.159921
H	0.555475	3.770636	1.633860	H	0.534234	3.814377	1.527027
C	-0.387341	1.933937	1.102784	C	-0.398022	1.953402	1.051676
C	0.517206	1.342732	-1.807992	C	0.517606	1.291450	-1.827110
N	0.581480	0.766611	-0.646046	N	0.584163	0.746614	-0.653948
N	-0.532431	0.992571	0.218427	N	-0.531919	0.989733	0.196078
Fe	2.067131	-0.474837	0.204686	Fe	2.039932	-0.449359	0.201582
Fe	-2.133344	-0.331688	-0.066951	Fe	-2.101150	-0.322705	-0.049740
C	-1.748859	-1.058531	1.552839	C	-1.689875	-1.049248	1.544057
O	-1.490152	-1.507127	2.594700	O	-1.422561	-1.509823	2.572568
O	0.539645	-3.031711	0.311534	O	0.577201	-3.018839	0.264272
C	1.057456	-1.997122	0.259390	C	1.072401	-1.977822	0.226789

Table S8. Optimized coordinates for the $(Me_2C{=}N)_2Fe_2(CO)_8$ structure **8S-4.**

	M06-L				B3PW91-D3		
	x	y	z		x	y	z
C	3.655277	-0.029354	0.077558	C	3.639076	0.020734	0.088327
C	1.782955	0.027068	1.917337	C	1.848348	-0.318055	1.861838
C	-3.661160	-0.056129	-0.057530	C	-3.665594	-0.208042	0.092723
C	-1.813211	0.068743	-1.914491	C	-2.141691	0.508565	-1.767090
O	4.811130	0.130456	0.051330	O	4.785412	0.215092	0.114775
O	1.642967	0.296029	3.040637	O	1.785858	-0.290832	3.017778
O	-4.818588	0.088423	-0.014978	O	-4.817631	-0.171676	0.251797
O	-1.690620	0.365739	-3.032712	O	-2.278051	1.043592	-2.785270
C	-1.805357	1.936969	2.303703	C	-1.797963	1.459084	2.601057
C	0.450883	2.927733	1.691292	C	0.475120	2.520086	2.157458
H	0.132905	3.925424	1.363659	H	0.161328	3.561872	2.004560
H	0.605609	2.987152	2.771871	H	0.630069	2.381510	3.232260
H	-2.053598	2.959218	2.603634	H	-1.975408	2.389488	3.151127
H	-1.554282	1.388603	3.220088	H	-1.579422	0.674638	3.338343
C	1.461855	-2.078302	0.725899	C	1.456534	-2.123229	0.303856
C	-1.444853	-2.059746	-0.774147	C	-1.325991	-1.724852	-1.222869
O	1.236042	-3.156045	1.107714	O	1.231212	-3.248968	0.474660
O	-1.206869	-3.125279	-1.182072	O	-1.013816	-2.616568	-1.897205
H	-2.673978	1.460963	1.844391	H	-2.693140	1.167390	2.047270
H	1.399231	2.690225	1.206083	H	1.418597	2.356479	1.632758
C	1.800922	1.976456	-2.275272	C	1.793688	2.332489	-1.893844
H	2.671026	1.497138	-1.822209	H	2.693030	1.887305	-1.461525
H	1.552275	1.439747	-3.199205	H	1.608242	1.854591	-2.865041
H	2.046211	3.003394	-2.561182	H	1.951535	3.400697	-2.076104
C	-0.454720	2.957381	-1.646086	C	-0.480856	3.156055	-1.106970
H	-0.611935	3.033245	-2.725289	H	-0.666271	3.387725	-2.161112
H	-1.402413	2.714760	-1.162288	H	-1.415475	2.844230	-0.638287
H	-0.133821	3.949432	-1.304459	H	-0.135250	4.082247	-0.627558
C	0.625742	1.963052	-1.360978	C	0.608383	2.127843	-1.004936
C	-0.630513	1.939502	1.388982	C	-0.623692	1.610392	1.688664
N	-0.596858	1.098050	0.399329	N	-0.602974	0.961957	0.567388
N	0.591582	1.104393	-0.386222	N	0.577588	1.105993	-0.209182
Fe	-1.923634	-0.450215	-0.187243	Fe	-1.938971	-0.394613	-0.233560
Fe	1.921598	-0.449171	0.179796	Fe	1.930468	-0.437734	0.076564
O	1.738667	-1.485458	-2.603511	O	1.974982	-1.021313	-2.819864
C	1.838446	-1.083034	-1.520433	C	1.963607	-0.784573	-1.690005
C	-1.813936	-1.114620	1.499714	C	-1.749949	-1.444038	1.216861
O	-1.692896	-1.530949	2.575279	O	-1.617111	-2.111521	2.149246

Table S9. Optimized coordinates for the $(Me_2C{=}N)_2Fe_2(CO)_8$ structure **8T-5.**

	M06-L				B3PW91-D3		
	x	y	z		x	y	z
C	1.580020	2.258272	0.701049	C	-0.211442	-2.509000	0.806561
C	1.593774	-0.282810	1.133158	C	-1.336614	-0.278938	1.417522
C	-1.168816	-2.293392	-0.553209	C	-0.211587	2.509008	-0.806616
C	0.387247	-0.552350	-1.561655	C	-1.336793	0.278926	-1.417441
O	1.855398	3.135787	1.408412	O	0.202618	-3.342066	1.489061
O	2.297290	-0.283619	2.082036	O	-1.693082	-0.561778	2.503909
O	-2.028159	-2.864813	-1.081879	O	0.202376	3.342080	-1.489168
O	0.142957	-0.786198	-2.692862	O	-1.693396	0.561758	-2.503785
C	-0.834198	1.336216	3.033612	C	2.786413	-0.490360	2.932295
C	-1.839111	3.009558	1.355238	C	3.627651	-1.921768	0.963453
H	-2.908131	3.164996	1.539945	H	4.692675	-1.673274	0.856377
H	-1.287086	3.737865	1.954444	H	3.553627	-2.745250	1.681819
H	-1.504110	1.663866	3.836207	H	3.793798	-0.466379	3.368999
H	0.096014	1.904701	3.156307	H	2.249455	-1.316660	3.417717
C	2.861772	0.708075	-1.182289	C	-2.616900	-1.945822	-0.388023
C	1.532135	-2.715283	-0.214052	C	-2.616887	1.945788	0.388266
O	3.917114	0.611179	-1.648638	O	-3.672197	-2.405304	-0.457218
O	2.312759	-3.528976	-0.477391	O	-3.672184	2.405249	0.457593
H	-0.619186	0.273889	3.164888	H	2.275776	0.449083	3.157534
H	-1.674844	3.191546	0.287449	H	3.261283	-2.242093	-0.016868
C	-3.535148	0.467888	-2.200135	C	2.786080	0.490395	-2.932426
H	-2.865385	1.160926	-2.712343	H	2.275409	-0.449041	-3.157615
H	-3.503567	-0.499802	-2.715941	H	2.249077	1.316705	-3.417783
H	-4.565906	0.830982	-2.277841	H	3.793417	0.466408	-3.369243
C	-3.986698	-0.563815	0.111944	C	3.627555	1.921783	-0.963669
H	-4.476417	-1.357987	-0.457213	H	3.553512	2.745251	-1.682049
H	-3.391494	-0.995474	0.922509	H	3.261255	2.242139	0.016668
H	-4.774077	0.043434	0.573803	H	4.692577	1.673261	-0.856648
C	-3.133226	0.291007	-0.773515	C	2.862719	0.719293	-1.450220
C	-1.443245	1.607484	1.699004	C	2.862881	-0.719267	1.450081
N	-1.610315	0.608201	0.894792	N	2.285895	0.134850	0.677081
N	-2.065774	0.901729	-0.380298	N	2.285814	-0.134821	-0.677156
Fe	0.256999	-1.468116	0.232241	Fe	-0.928401	1.263045	0.297215
Fe	1.191648	0.903310	-0.447738	Fe	-0.928414	-1.263050	-0.297185
O	0.017204	2.692456	-2.531856	O	0.083400	-2.550457	-2.768240
C	0.453097	1.994392	-1.718663	C	-0.287175	-2.029478	-1.809269
C	-0.113554	-2.073118	1.917317	C	-0.286984	2.029478	1.809222
O	-0.337355	-2.495346	2.972480	O	0.083698	2.550462	2.768150

Table S10. Optimized coordinates for the $(Me_2C=N)_2Fe_2(CO)_8$ structure **8T-6.**

	M06-L				B3PW91-D3		
	x	y	z		x	y	z
C	3.486123	-0.570228	0.383407	C	-3.830184	-0.977558	0.314769
C	1.539271	0.535699	-1.766690	C	-1.800947	-0.217461	-1.888460
C	-3.235881	-1.313402	-0.291327	C	2.815380	1.083349	-1.488446
C	-2.096633	0.518209	1.560400	C	2.891191	0.486644	1.257886
O	4.321836	-1.334180	0.667287	O	-4.922867	-0.952775	0.711971
O	1.191688	0.538607	-2.880194	O	-1.597011	0.207991	-2.948775
O	-3.702647	-2.376098	-0.282697	O	2.415252	1.920922	-2.171042
O	-1.834944	0.561538	2.692798	O	2.493690	0.913240	2.256849
C	-0.696711	-2.418905	-2.460353	C	-1.577224	3.674552	-1.364363
C	1.551403	-2.882159	-1.322312	C	-3.617496	2.474096	-0.369444
H	1.462553	-3.860037	-1.801368	H	-4.074976	3.467034	-0.433207
H	2.280294	-2.296102	-1.897183	H	-4.008887	1.873426	-1.201568
H	-1.086192	-3.442059	-2.419167	H	-1.853228	4.654808	-0.953987
H	-0.164863	-2.315364	-3.412316	H	-2.014624	3.611531	-2.369570
C	3.320598	1.819614	-0.488644	C	-2.523831	-2.515712	-1.156357
C	-4.108782	1.358872	-0.150679	C	5.230156	0.452478	-0.185783
O	4.091225	2.643832	-0.763549	O	-2.771479	-3.505645	-1.703746
O	-5.107176	1.946610	-0.088000	O	6.308836	0.856658	-0.092296
H	-1.532413	-1.714069	-2.443598	H	-0.489795	3.603602	-1.441539
H	1.960197	-2.998411	-0.315788	H	-3.926461	1.986859	0.558591
C	1.417404	-1.032328	2.880937	C	-2.005795	-0.255800	3.014988
H	2.214332	-0.340438	2.610115	H	-2.394990	-1.134158	2.499266
H	0.766234	-0.552894	3.621908	H	-1.114169	-0.541695	3.589740
H	1.846355	-1.917058	3.363765	H	-2.747160	0.107683	3.738654
C	-0.465269	-2.469901	1.934957	C	-1.171240	2.136966	2.675122
H	-0.698596	-2.558766	2.997361	H	-0.920554	1.994983	3.729958
H	-1.373588	-2.223035	1.377178	H	-0.297348	2.511437	2.130656
H	-0.132112	-3.451940	1.577802	H	-1.948240	2.910080	2.608488
C	0.597741	-1.441596	1.709428	C	-1.648913	0.846203	2.069116
C	0.241140	-2.161309	-1.328347	C	-2.122328	2.574253	-0.500326
N	-0.123227	-1.279001	-0.461519	N	-1.260717	1.779576	0.030712
N	0.800849	-0.923943	0.534318	N	-1.733643	0.696556	0.783461
Fe	-2.518866	0.410698	-0.223821	Fe	3.518779	-0.180543	-0.321410
Fe	2.149925	0.560709	-0.059179	Fe	-2.151135	-1.012852	-0.310407
C	1.341956	1.694186	1.104777	C	-0.741738	-1.813819	0.459213
O	0.811314	2.420502	1.844457	O	0.182980	-2.330030	0.937027
O	-0.931456	2.747976	-1.353699	O	4.016310	-3.025499	0.534255
C	-1.460994	1.803900	-0.949143	C	3.800721	-1.953049	0.175185

Table S11. Optimized coordinates for the $(Me_2C{=}N)_2Fe_2(CO)_8$ structure **8T-7.**

	M06-L				B3PW91-D3		
	x	y	z		x	y	z
C	3.324344	1.538569	0.171776	C	4.418271	-0.487737	1.430776
C	0.710702	2.280375	0.848607	C	1.804408	-1.654452	1.291594
C	-1.170636	-1.328528	-1.574708	C	-2.147847	0.913542	-1.497943
C	-0.710694	-2.280363	0.848621	C	-1.803363	-1.648945	-1.298055
O	4.397840	1.977762	0.178995	O	5.412479	-0.515065	2.019718
O	0.109076	3.172163	1.280858	O	1.127700	-2.439164	1.804036
O	-0.929188	-1.580955	-2.673684	O	-1.722570	1.787956	-2.113887
O	-0.109060	-3.172144	1.280875	O	-1.126175	-2.431183	-1.813634
C	-2.289790	3.154306	-1.287285	C	-2.678473	2.334358	2.769791
C	-4.092722	1.502384	-0.527394	C	-4.824285	2.143707	1.366920
H	-4.248887	1.084392	0.472578	H	-5.270201	1.612702	0.522206
H	-4.705880	2.402003	-0.630549	H	-5.437763	1.978329	2.262568
H	-2.596397	3.920093	-0.564947	H	-3.223614	2.158708	3.706755
H	-2.841926	3.351270	-2.213471	H	-2.634209	3.421175	2.619063
C	1.709081	0.021834	1.809390	C	3.405638	-1.643559	-0.751378
C	-3.324348	-1.538558	0.171798	C	-4.418898	-0.485326	-1.430893
O	1.812536	-0.530792	2.820163	O	3.773456	-2.380290	-1.554946
O	-4.397845	-1.977748	0.179015	O	-5.413685	-0.512170	-2.018879
H	-1.220230	3.258334	-1.479109	H	-1.663158	1.941929	2.863608
H	-4.453789	0.760866	-1.248419	H	-4.836772	3.222680	1.162898
C	4.092711	-1.502395	-0.527394	C	4.822577	2.150790	-1.358427
H	4.705860	-2.402023	-0.630528	H	5.437424	1.988678	-2.253735
H	4.453787	-0.760897	-1.248436	H	4.833215	3.229237	-1.151546
H	4.248880	-1.084382	0.472568	H	5.268209	1.618207	-0.514557
C	2.289774	-3.154312	-1.287277	C	2.678041	2.342372	-2.763131
H	2.596400	-3.920098	-0.564946	H	3.224626	2.170634	-3.699979
H	1.220212	-3.258345	-1.479086	H	1.663528	1.948469	-2.859429
H	2.841896	-3.351268	-2.213474	H	2.631687	3.428628	-2.609028
Fe	-1.620006	-0.854200	0.173233	Fe	-2.859374	-0.431346	-0.497896
Fe	1.620003	0.854208	0.173219	Fe	2.859649	-0.432986	0.496325
C	2.620979	-1.779441	-0.758432	C	3.401133	1.677582	-1.604784
C	-2.620991	1.779438	-0.758433	C	-3.401830	1.671966	1.610234
N	1.687913	-0.944692	-0.540379	N	2.825722	0.791977	-0.913659
N	-1.687922	0.944696	-0.540368	N	-2.825814	0.789376	0.915766
O	-1.812484	0.530797	2.820183	O	-3.768314	-2.386204	1.548349
C	-1.709054	-0.021826	1.809406	C	-3.402356	-1.646502	0.746662
C	1.170622	1.328511	-1.574725	C	2.145371	0.907875	1.499798
O	0.929169	1.580899	-2.673709	O	1.718382	1.779838	2.118027

Table S12. Optimized coordinates for the $(Me_2C{=}N)_2Fe_2(CO)_8$ structure **8T-8.**

	M06-L				B3PW91-D3		
	x	y	z		x	y	z
C	1.564291	0.420642	1.567534	C	-3.997375	0.520877	-1.664831
C	3.933662	-0.833626	1.189479	C	-5.585849	0.082817	0.429593
C	-4.165060	-0.506876	1.572149	C	5.935219	-0.721409	-0.255657
C	-3.388195	-0.978351	-1.164204	C	4.043672	-0.085155	1.809538
O	0.797633	0.506907	2.425334	O	-4.111862	0.542556	-2.809612
O	4.651531	-1.502064	1.808248	O	-6.704447	-0.143276	0.608470
O	-4.927791	-0.838826	2.381663	O	6.997531	-1.145836	-0.425054
O	-3.651418	-1.622754	-2.093905	O	3.885109	-0.098344	2.954316
C	0.149770	2.861872	-0.493307	C	1.062159	1.437839	-2.382626
C	-0.820940	1.396220	-2.337708	C	0.817606	1.361553	0.175308
H	-0.907524	2.289057	-2.966649	H	0.565445	2.429347	0.224273
H	0.133060	0.911911	-2.583066	H	-0.128911	0.809355	0.101518
H	-0.134785	3.803297	-0.976624	H	0.815589	2.507322	-2.416529
H	1.155678	2.606704	-0.850531	H	0.124267	0.884434	-2.522569
C	3.467986	1.902070	0.576056	C	-3.680484	2.213979	0.282087
C	-1.740342	-1.170810	0.946347	C	3.532481	-1.699191	-0.245817
O	3.863452	2.972029	0.790772	O	-3.586203	3.363085	0.368269
O	-0.965200	-1.941142	1.325802	O	3.021220	-2.718996	-0.402373
H	0.174342	3.010747	0.587900	H	1.749784	1.199936	-3.197973
H	-1.641082	0.711053	-2.569879	H	1.336624	1.072814	1.092916
C	1.856004	-3.167202	-0.431608	C	-3.855457	-3.279381	-0.183128
H	2.049933	-3.887353	-1.233959	H	-3.677306	-4.003399	0.622975
H	2.798163	-2.905312	0.053750	H	-4.775207	-2.727535	0.025752
H	1.211413	-3.670173	0.298544	H	-3.981211	-3.854586	-1.109965
C	-0.044306	-2.222695	-1.843645	C	-1.338369	-2.991169	-0.605495
H	-0.705093	-2.957257	-1.367966	H	-1.392555	-3.552732	-1.547468
H	-0.606466	-1.306858	-2.040310	H	-0.546063	-2.242558	-0.678288
H	0.274942	-2.655249	-2.798428	H	-1.084204	-3.709481	0.184998
Fe	-2.984350	0.018087	0.304980	Fe	4.283351	-0.062314	0.008482
Fe	2.820183	0.248594	0.221203	Fe	-3.820773	0.425504	0.145567
C	1.155477	-1.942916	-0.968595	C	-2.674478	-2.334295	-0.308484
C	-0.819617	1.769697	-0.875598	C	1.681448	1.086139	-1.041314
N	1.499677	-0.748691	-0.721491	N	-2.760253	-1.080649	-0.179847
N	-1.559495	1.232164	-0.002568	N	2.843685	0.597314	-0.982329
O	4.385758	0.002115	-2.315646	O	-3.079549	0.018428	2.995389
C	3.784467	0.130729	-1.337222	C	-3.363319	0.199305	1.895318
C	-4.055802	1.429494	-0.170911	C	4.906044	1.648959	-0.019848
O	-4.701450	2.323974	-0.514485	O	5.263567	2.742667	-0.034098

Table S13. Optimized coordinates for the $(Me_2C{=}N)_2Fe_2(CO)_8$ structure **8T-9.**

	M06-L				B3PW91-D3		
	x	y	z		x	y	z
C	1.761216	-0.581621	-1.729871	C	1.987574	-0.191727	-1.729353
C	2.842985	-0.973963	0.332076	C	2.871037	-0.937988	0.322694
C	-1.605721	-1.280919	-1.443341	C	-1.610210	-1.022531	-1.646442
C	-1.707379	1.757971	0.602396	C	-1.796023	1.553868	0.936448
O	1.387565	-0.648979	-2.859699	O	1.723527	-0.039854	-2.882194
O	3.523619	-1.407735	1.212443	O	3.459739	-1.520561	1.182792
O	-1.643435	-2.220540	-2.116519	O	-1.652980	-1.819952	-2.474815
O	-1.747622	2.669388	1.314130	O	-1.891014	2.320656	1.789153
C	1.305577	3.581109	0.113023	C	1.006339	3.636021	0.579008
C	2.849677	2.197552	1.621680	C	2.608834	2.181131	1.950599
H	3.772932	2.392568	1.065772	H	3.516550	2.629085	1.527144
H	2.748176	2.978717	2.382215	H	2.338609	2.762474	2.841767
H	2.182487	4.037143	-0.358233	H	1.860660	4.243596	0.254079
H	0.985272	4.249001	0.920058	H	0.573528	4.127028	1.460128
C	0.722078	-2.439105	-0.159445	C	0.877003	-2.269869	-0.631214
C	-1.369796	1.249931	-1.922450	C	-1.681039	1.551969	-1.655523
O	0.669433	-3.589102	-0.305232	O	0.874468	-3.373116	-0.976967
O	-1.119583	1.909935	-2.840814	O	-1.623658	2.377783	-2.457162
H	0.506966	3.500473	-0.626300	H	0.266017	3.609442	-0.223673
H	2.937003	1.224455	2.109434	H	2.825322	1.151494	2.243725
C	-0.064965	-1.452178	3.252805	C	0.060944	-2.122234	2.820655
H	-0.345323	-2.458763	3.582716	H	-0.224359	-3.165016	3.011468
H	0.918643	-1.500437	2.774848	H	1.026306	-2.102898	2.307825
H	0.013553	-0.835860	4.155101	H	0.178205	-1.638952	3.799836
C	-2.473590	-0.686489	2.915872	C	-2.396477	-1.500985	2.635908
H	-2.441206	0.128663	3.648349	H	-2.373849	-1.152489	3.676440
H	-3.207059	-0.434708	2.147304	H	-3.124022	-0.910567	2.076616
H	-2.806089	-1.580110	3.453918	H	-2.732356	-2.546320	2.658198
Fe	-1.743041	0.263575	-0.448096	Fe	-1.775547	0.284803	-0.369723
Fe	0.878644	-0.652640	0.111045	Fe	0.961871	-0.571423	-0.047144
C	-1.109639	-0.894202	2.322588	C	-1.017623	-1.418598	2.026546
C	1.680539	2.230268	0.665894	C	1.484716	2.241175	0.931501
N	-0.825529	-0.608530	1.113163	N	-0.753482	-0.811008	0.936497
N	1.059195	1.182804	0.330388	N	0.981680	1.209416	0.403449
C	-3.546673	0.114577	-0.376263	C	-3.568068	-0.014152	-0.269809
O	-4.705459	0.049189	-0.399939	O	-4.712010	-0.169541	-0.262496
C	3.105395	-0.330324	-1.038109	C	3.324340	-0.130729	-0.932718
O	3.789864	0.667718	-1.196603	O	4.136865	0.765121	-0.943867

Table S14. Optimized coordinates for the $(Me_2C{=}N)_2Fe_2(CO)_8$ structure **8S-10.**

	M06-L				B3PW91-D3		
	x	y	z		x	y	z
C	-1.424038	-1.031900	-1.599280	C	1.019556	0.751244	-1.518360
C	-2.235039	0.370539	1.271575	C	-0.839917	2.520500	0.683414
C	1.090779	0.373904	-2.057190	C	4.551548	-0.384540	-0.841306
C	2.682331	-0.348692	1.113640	C	-1.488218	-2.562364	0.339149
O	-1.606775	-1.855117	-2.384270	O	1.541838	0.765889	-2.549905
O	-2.118405	0.495030	2.463861	O	-1.644909	2.899339	1.501093
O	0.929860	-0.028074	-3.178738	O	5.662574	-0.513327	-1.069520
O	3.586404	-0.354109	1.841452	O	-2.234414	-3.378815	0.674460
C	0.521427	-3.163207	1.949890	C	-3.521586	-0.694056	-1.621893
C	-1.956128	-2.764637	1.510403	C	-3.083180	1.805042	-1.461258
H	-2.015549	-3.853796	1.427300	H	-3.836886	1.799793	-2.255613
H	-2.315656	-2.498135	2.510780	H	-3.552447	2.206699	-0.554158
H	0.609038	-4.099702	1.386762	H	-3.647108	-0.669404	-2.712621
H	0.251272	-3.431848	2.976344	H	-4.513290	-0.522661	-1.183359
C	-1.466870	1.663429	-1.355317	C	1.703724	1.502944	0.868709
C	1.889424	-1.809827	-0.936347	C	-0.232581	-1.854452	-1.908304
O	-1.703830	2.582834	-2.012500	O	2.681495	1.773893	1.418561
O	2.166162	-2.825079	-1.421217	O	-0.204983	-2.211964	-3.003877
H	1.492908	-2.663968	1.947921	H	-3.167410	-1.684177	-1.331082
H	-2.631335	-2.322107	0.773565	H	-2.275625	2.481205	-1.752776
C	-0.693486	3.213239	1.568971	C	-0.092320	0.797905	3.604792
H	-0.459755	4.190241	1.131792	H	0.891712	0.716593	4.086612
H	-1.638756	2.860033	1.153925	H	-0.161367	1.747212	3.073550
H	-0.812678	3.364310	2.646647	H	-0.845147	0.780674	4.402172
C	1.768335	2.647966	1.887317	C	-0.599529	-1.682350	3.371876
H	1.844915	2.239326	2.901645	H	-1.644942	-1.684175	3.708878
H	2.593722	2.260743	1.288848	H	-0.438204	-2.543733	2.721257
H	1.862544	3.734273	1.963032	H	0.026440	-1.788300	4.266112
Fe	1.367143	-0.300662	-0.094283	Fe	-0.322419	-1.334351	-0.185486
Fe	-1.114702	0.219284	-0.299039	Fe	0.180193	1.051033	0.046532
C	0.437771	2.266166	1.296952	C	-0.293361	-0.375569	2.677076
C	-0.538898	-2.292717	1.333502	C	-2.575035	0.403005	-1.196887
N	0.301185	1.194517	0.621503	N	-0.206801	-0.271480	1.411748
N	-0.217812	-1.224329	0.718976	N	-1.449592	0.175361	-0.649523
C	2.064464	1.053354	-1.215227	C	1.192515	-2.214002	0.245495
O	2.718973	2.070465	-1.240218	O	2.156163	-2.772965	0.540750
C	-2.991698	0.119617	0.126131	C	-0.140837	2.833897	-0.531024
O	-4.074638	0.009265	-0.397828	O	0.148763	3.702469	-1.319143

Table S15. Optimized coordinates for the $(Me_2C{=}N)_2Cr_2(CO)_{10}$ structure **10S-1.**

	M06-L				B3PW91-D3		
	x	y	z		x	y	z
C	-0.517374	3.447190	0.979411	C	-0.498428	3.427413	0.959070
C	1.828629	2.323951	0.330544	C	1.803898	2.322257	0.302064
C	0.517374	-3.447190	0.979411	C	0.498428	-3.427413	0.959070
C	-1.828629	-2.323951	0.330544	C	-1.803898	-2.322257	0.302064
O	-0.808260	4.226296	1.792965	O	-0.766000	4.223153	1.758680
O	2.938611	2.418064	0.672647	O	2.912457	2.435850	0.627466
O	0.808260	-4.226296	1.792965	O	0.766000	-4.223153	1.758680
O	-2.938611	-2.418064	0.672647	O	-2.912457	-2.435850	0.627466
C	2.638389	-1.274474	1.790837	C	2.658224	-1.258381	1.792307
C	1.498373	0.695206	2.882346	C	1.506343	0.702620	2.881260
H	1.250516	0.220739	3.840447	H	1.261869	0.218435	3.836779
H	2.506252	1.103421	2.984892	H	2.517714	1.108568	2.972395
H	2.725535	-1.804537	2.745792	H	2.812284	-1.695152	2.786662
H	3.552373	-0.681563	1.674514	H	3.545370	-0.653276	1.563382
C	0.872016	1.176434	-1.764458	C	0.858200	1.173253	-1.742906
C	-0.872016	-1.176434	-1.764458	C	-0.858200	-1.173253	-1.742906
O	1.452584	0.680634	-2.638685	O	1.450121	0.693423	-2.612906
O	-1.452584	-0.680634	-2.638685	O	-1.450121	-0.693423	-2.612906
H	2.584153	-2.004808	0.986133	H	2.580304	-2.057609	1.058600
H	0.798991	1.515386	2.711494	H	0.806366	1.522354	2.716529
C	-2.638389	1.274474	1.790837	C	-2.658224	1.258381	1.792307
H	-2.584153	2.004808	0.986133	H	-2.580304	2.057609	1.058600
H	-3.552373	0.681563	1.674514	H	-3.545370	0.653276	1.563382
H	-2.725535	1.804537	2.745792	H	-2.812284	1.695152	2.786662
C	-1.498373	-0.695206	2.882346	C	-1.506343	-0.702620	2.881260
H	-2.506252	-1.103421	2.984892	H	-2.517714	-1.108568	2.972395
H	-0.798991	-1.515386	2.711494	H	-0.806366	-1.522354	2.716529
H	-1.250516	-0.220739	3.840447	H	-1.261869	-0.218435	3.836779
C	-1.466914	0.354718	1.814313	C	-1.468093	0.352680	1.809562
C	1.466914	-0.354718	1.814313	C	1.468093	-0.352680	1.809562
N	0.502299	-0.508721	0.958474	N	0.498428	-0.509262	0.965127
N	-0.502299	0.508721	0.958474	N	-0.498428	0.509262	0.965127
O	-2.638389	2.249869	-1.866513	O	-2.648808	2.167247	-1.805946
C	-1.640215	2.202309	-1.279861	C	-1.642445	2.144047	-1.240337
C	1.640215	-2.202309	-1.279861	C	1.642445	-2.144047	-1.240337
O	2.638389	-2.249869	-1.866513	O	2.648808	-2.167247	-1.805946
Cr	-0.067489	-2.233809	-0.376097	Cr	-0.054445	-2.206433	-0.371642
Cr	0.067489	2.233809	-0.376097	Cr	0.054445	2.206433	-0.371642
C	0.494302	3.720816	-1.406962	C	0.440329	3.676459	-1.417101
C	-0.494302	-3.720816	-1.406962	C	-0.440329	-3.676459	-1.417101
O	-0.754994	-4.658332	-2.048325	O	-0.672387	-4.607730	-2.071587

O	0.754994	4.658332	-2.048325	O	0.672387	4.607730	-2.071587

Table S16. Optimized coordinates for the $(Me_2C{=}N)_2Cr_2(CO)_{10}$ structure **10S-2.**

	M06-L				B3PW91-D3		
	x	y	z		x	y	z
C	-1.619796	-0.931294	0.741762	C	-1.278066	-0.197626	1.348316
C	-2.309148	1.264215	-0.734286	C	-1.764475	1.447811	-0.883662
C	1.139373	-0.053392	-1.372004	C	1.300090	0.397063	-1.783993
C	3.961109	0.560324	1.180741	C	2.866747	-0.247004	1.646033
O	-0.659424	-1.332075	1.244228	O	-0.633019	-0.294806	2.296352
O	-1.754453	2.200315	-1.130190	O	-1.359508	2.390493	-1.411167
O	0.222156	-0.269091	-2.046533	O	0.811952	0.623062	-2.802739
O	4.775385	0.665253	1.995110	O	3.252774	-0.482389	2.704097
C	2.208226	-3.590203	1.614455	C	-0.032333	-3.508697	0.857809
C	4.305589	-3.176410	0.208290	C	2.479169	-3.681101	0.413412
H	4.764489	-3.861044	0.928264	H	2.411807	-4.489317	1.150690
H	4.141089	-3.741159	-0.716508	H	2.559780	-4.143111	-0.579220
H	2.784305	-3.837229	2.513588	H	0.067829	-3.936858	1.863586
H	2.027958	-4.532446	1.083647	H	-0.222632	-4.338208	0.163940
C	-3.660533	0.660814	1.550343	C	-3.545314	1.098180	1.043985
C	3.203933	1.949721	-0.940494	C	3.576714	1.314044	-0.276937
O	-3.910850	1.225963	2.529943	O	-4.133533	1.826466	1.720375
O	3.600288	2.956444	-1.360176	O	4.446857	2.074264	-0.317017
H	1.254079	-3.153544	1.908629	H	-0.887936	-2.832466	0.845932
H	5.006736	-2.367467	-0.009986	H	3.389472	-3.103385	0.597831
C	0.733413	3.707309	0.174013	C	1.561109	3.913156	-0.094025
H	1.514590	4.372528	0.559565	H	2.197435	4.495685	0.585299
H	0.959843	3.489894	-0.872171	H	2.171488	3.540941	-0.920154
H	-0.215737	4.248886	0.223824	H	0.796949	4.591577	-0.493158
C	-0.254018	2.466579	2.184364	C	-0.223471	3.194264	1.592857
H	-1.281776	2.661323	1.851739	H	-1.052376	3.632415	1.022507
H	-0.222500	1.525494	2.736326	H	-0.589197	2.346254	2.174391
H	0.015401	3.286707	2.859845	H	0.142222	3.967581	2.280925
C	0.682105	2.437368	0.996271	C	0.894051	2.774619	0.653585
C	2.993873	-2.639121	0.738962	C	1.248103	-2.791475	0.471444
N	1.368616	1.402720	0.763506	N	1.203042	1.558551	0.527414
N	2.536838	-1.486510	0.497719	N	1.247402	-1.553057	0.221231
O	-5.846430	0.757251	-1.341828	O	-4.774382	0.064509	-2.156558
C	-4.866217	0.367457	-0.865188	C	-3.929131	0.014592	-1.371442
C	-2.825495	-1.230072	-1.694250	C	-1.560260	-1.209129	-1.227818
O	-2.592188	-1.808247	-2.667475	O	-1.012855	-1.903309	-1.966663
Cr	-3.241112	-0.276944	-0.069854	Cr	-2.557591	-0.071423	-0.069712
Cr	2.609645	0.300887	-0.181530	Cr	2.167607	0.078212	-0.109901
C	-4.179662	-1.821876	0.584873	C	-3.425959	-1.581419	0.676653
C	3.732196	-0.497550	-1.503243	C	3.286071	-1.032528	-1.147208
O	-4.745965	-2.751064	0.979183	O	-3.956453	-2.504683	1.123494

O	4.361336	-1.010154	-2.333481	O	3.921094	-1.713732	-1.832226

Table S17. Optimized coordinates for the $(Me_2C{=}N)_2Cr_2(CO)_{10}$ structure **10S-3.**

	M06-L				B3PW91-D3		
	x	y	z		x	y	z
C	3.303481	1.070224	1.129556	C	3.445229	-0.106823	1.512945
C	1.622110	-1.658426	-0.980666	C	1.569254	-0.965066	-1.669368
C	-5.074768	0.266748	1.619202	C	-5.442170	-1.503474	1.741985
C	-1.430451	-0.578078	-1.955596	C	-1.400453	0.780403	-2.194661
O	3.873081	1.900071	1.714395	O	4.104616	0.105697	2.444037
O	1.261688	-2.507765	-1.679464	O	1.186677	-1.292736	-2.707293
O	-6.105083	0.166517	1.133561	O	-6.493036	-1.942757	1.685121
O	-1.028522	-0.616930	-3.054782	O	-1.025953	1.152444	-3.232930
C	-1.761631	0.843846	2.374582	C	-2.025671	-0.329789	2.145212
C	0.415003	2.217017	2.353843	C	0.207495	0.394129	3.190704
H	-0.133781	2.988460	2.902449	H	-0.179976	1.200311	3.829145
H	0.994101	1.653748	3.096370	H	0.162601	-0.529253	3.779916
H	-2.525762	1.627544	2.408728	H	-2.521306	0.079482	3.033165
H	-1.529202	0.557491	3.404920	H	-1.955750	-1.416531	2.272822
C	1.496566	-0.896800	1.707971	C	1.431787	-1.797822	0.931705
C	-3.491552	-1.432829	-0.535417	C	-3.628182	-0.195006	-1.243406
O	1.010485	-1.280705	2.693466	O	0.889282	-2.643721	1.509805
O	-4.432674	-2.094328	-0.743230	O	-4.673143	-0.479146	-1.675711
H	-2.223627	-0.053237	1.919739	H	-2.736149	-0.129448	1.313166
H	1.117035	2.692028	1.666652	H	1.247705	0.610449	2.943429
C	1.816176	2.434861	-2.018396	C	2.409620	3.007960	0.266275
H	2.744856	2.554166	-1.452889	H	3.249848	2.414123	0.630784
H	1.966750	1.611525	-2.723607	H	2.517002	3.117392	-0.820296
H	1.633217	3.345727	-2.590499	H	2.457898	4.008682	0.706237
C	-0.540755	3.003790	-1.262371	C	0.007642	3.330790	0.978408
H	-1.043472	2.763940	-2.207586	H	-0.068720	4.117005	0.217075
H	-1.266043	2.892713	-0.457437	H	-0.970037	2.872480	1.110334
H	-0.230839	4.050467	-1.332446	H	0.305024	3.828270	1.911506
C	0.664187	2.136085	-1.115332	C	1.089156	2.369542	0.582338
C	-0.534922	1.308075	1.658793	C	-0.655954	0.261739	1.977027
N	-0.411483	0.851648	0.455851	N	-0.348652	0.594136	0.769033
N	0.762997	1.145382	-0.281479	N	0.953078	1.082644	0.502202
C	3.742178	-1.515873	0.634258	C	3.620703	-1.891299	-0.366522
O	4.570197	-2.272743	0.949348	O	4.365852	-2.759170	-0.569898
O	-0.832485	-3.122479	0.568345	O	-1.414210	-2.646876	-1.306575
C	-1.188765	-2.048640	0.303971	C	-1.546566	-1.548421	-0.962495
Cr	-1.993027	-0.383285	-0.227968	Cr	-1.987333	0.252606	-0.550423
Cr	2.421094	-0.300443	0.143807	Cr	2.437366	-0.515475	-0.028274
C	3.565586	0.088742	-1.334080	C	3.586613	0.548945	-1.093176
C	-3.088156	1.053420	-0.803662	C	-2.745519	1.965004	-0.389163
O	4.355266	0.229056	-2.176034	O	4.356116	1.080425	-1.776948

O	-3.837351	1.848394	-1.217490	O	-3.305929	2.985036	-0.417614

Table S18. Optimized coordinates for the $(Me_2C{=}N)_2Cr_2(CO)_{10}$ structure **10S-4.**

	M06-L				B3PW91-D3		
	x	y	z		x	y	z
C	2.555381	-0.440192	-1.858803	C	-4.277232	0.435084	-1.658479
C	4.317207	0.115962	0.241086	C	-5.063800	-0.776628	0.622336
C	-0.963521	0.317993	1.537826	C	1.875279	-0.198857	1.267431
C	-2.288202	2.455744	0.353800	C	3.579763	-2.202475	0.543496
O	2.593133	-0.326789	-3.009747	O	-4.719069	0.552670	-2.717775
O	5.384615	0.543401	0.379544	O	-5.957882	-1.441737	0.921403
O	-0.282510	0.212530	2.467846	O	0.969770	-0.078221	1.973080
O	-2.411389	3.602871	0.509718	O	3.710104	-3.323482	0.815819
C	-2.883505	-3.733948	-0.549816	C	2.219632	3.236209	-1.732918
C	-4.876700	-2.151787	-0.340245	C	3.744941	3.769867	0.275818
H	-5.168334	-1.111238	-0.185060	H	4.319800	3.312426	1.084656
H	-5.292024	-2.499043	-1.292536	H	4.416318	4.368971	-0.352923
H	-3.266247	-4.120076	-1.500848	H	2.853691	3.829686	-2.404323
H	-3.279135	-4.381342	0.240418	H	1.428263	3.893988	-1.352026
C	3.399524	-2.307313	-0.124406	C	-4.531005	1.896365	0.492433
C	-3.594732	0.455112	1.457071	C	4.558327	-0.006346	1.560882
O	3.935409	-3.330717	-0.256163	O	-5.114422	2.873587	0.716015
O	-4.402047	0.366610	2.283062	O	5.277531	0.171913	2.444782
H	-1.797021	-3.783623	-0.548093	H	1.764851	2.415371	-2.291683
H	-5.320131	-2.767479	0.449777	H	2.995909	4.449509	0.702388
C	3.182461	3.018120	-0.945193	C	-1.990920	-2.939590	-1.960698
H	2.817946	3.697959	-1.722303	H	-0.957671	-2.962897	-2.329628
H	3.836582	3.599528	-0.285730	H	-2.368074	-3.970339	-1.953052
H	3.769290	2.224088	-1.411602	H	-2.600841	-2.334847	-2.636161
C	0.976007	3.462777	0.268601	C	-1.251467	-3.174658	0.499086
H	0.375595	3.759970	-0.600048	H	-0.214747	-3.326030	0.172609
H	0.313688	3.035447	1.023748	H	-1.258591	-2.665411	1.465483
H	1.447042	4.369224	0.663206	H	-1.712133	-4.165154	0.607570
C	2.016406	2.454215	-0.157595	C	-2.015087	-2.366486	-0.546607
C	-3.368220	-2.316800	-0.341984	C	3.053763	2.703968	-0.571056
N	1.894040	1.230913	0.114539	N	-2.618999	-1.310964	-0.262360
N	-2.575157	-1.350904	-0.185644	N	3.160471	1.483154	-0.330029
O	-4.297149	0.973606	-2.168307	O	5.739872	-0.438838	-1.824729
C	-3.528174	0.834287	-1.312976	C	4.845773	-0.424151	-1.095444
C	2.552530	-0.760695	1.993082	C	-2.996635	0.368010	1.955033
O	2.612637	-0.889044	3.140814	O	-2.685748	0.453692	3.061834
Cr	2.548131	-0.618360	0.059940	Cr	-3.580197	0.308870	0.123456
Cr	-2.246561	0.575684	0.093445	Cr	3.366805	-0.383461	0.103107
C	0.892402	-1.635807	-0.034111	C	-2.035765	1.283318	-0.401122
C	-0.892523	0.722567	-1.298425	C	2.207379	-0.975038	-1.300886
O	0.048034	-2.430732	-0.079566	O	-1.098176	1.873980	-0.725731

O	-0.166379	0.890679	-2.184939	O	1.518853	-1.397561	-2.127137

Table S19. Optimized coordinates for the $(Me_2C=N)_2Cr_2(CO)_{10}$ structure **10S-5.**

	M06-L				B3PW91-D3		
	x	y	z		x	y	z
C	-3.440128	-1.617689	-0.755633	C	-3.558895	-1.488188	-1.098635
C	-4.244682	-0.699722	1.706010	C	-4.541017	-1.012634	1.407125
C	4.693253	-1.880847	-0.101002	C	4.841295	-1.714336	-0.334895
C	1.956607	-2.194732	0.295252	C	2.258955	-2.300472	0.326100
O	-3.023345	-2.612051	-1.168430	O	-3.230044	-2.415307	-1.695175
O	-4.296542	-1.150071	2.770049	O	-4.760647	-1.655072	2.337805
O	5.661198	-2.516347	-0.210464	O	5.834239	-2.261229	-0.580946
O	1.261410	-3.119956	0.433781	O	1.694674	-3.307423	0.476439
C	6.654858	1.205304	-0.479360	C	6.429536	1.620909	-0.865876
C	5.133354	3.259064	-0.366652	C	4.729867	3.480222	-0.383548
H	5.748854	3.713909	0.417656	H	5.308915	3.926953	0.436749
H	5.520382	3.640796	-1.318154	H	5.057823	3.975402	-1.306694
H	7.318889	1.597612	0.299097	H	7.169429	2.159828	-0.259908
H	7.088317	1.516286	-1.436588	H	6.605541	1.911612	-1.911009
C	-5.910478	-0.644778	-0.477260	C	-5.886339	-0.300990	-0.868355
C	2.760726	-0.820273	-1.803190	C	2.668743	-0.780072	-1.737267
O	-6.960695	-1.071397	-0.726251	O	-6.927484	-0.522001	-1.318903
O	2.453110	-0.888398	-2.923019	O	2.249118	-0.838409	-2.817313
H	6.647714	0.115999	-0.427629	H	6.587927	0.545197	-0.765989
H	4.096710	3.569294	-0.243925	H	3.668225	3.664224	-0.212684
C	-0.105583	0.069780	0.244256	C	-0.222667	-0.411615	0.830175
H	0.594631	0.036508	1.090622	H	0.126417	-0.771664	1.806248
H	-0.496629	-0.927254	0.036862	H	-0.585666	-1.251506	0.234927
H	0.450087	0.443063	-0.623424	H	0.621096	0.080191	0.324289
C	-0.814900	2.390360	1.116989	C	-0.944302	1.856698	1.858757
H	0.002906	2.790716	0.510421	H	-0.037817	2.285989	1.414931
H	-1.652205	3.087965	1.160515	H	-1.755589	2.588530	1.876019
H	-0.413798	2.240850	2.125014	H	-0.704174	1.552985	2.885607
C	-1.237811	1.057017	0.557257	C	-1.330435	0.627096	1.060404
C	5.282343	1.771759	-0.324016	C	5.037584	2.009802	-0.460175
N	-2.418295	0.731405	0.334564	N	-2.469813	0.423913	0.607721
N	4.284420	0.972087	-0.160491	N	4.174434	1.094654	-0.199011
O	-5.616058	2.597394	0.953853	O	-5.517380	2.447409	1.263470
C	-5.059809	1.657448	0.577594	C	-4.992235	1.560807	0.749907
C	3.288514	-0.653824	1.970335	C	3.591192	-0.741931	1.914258
O	3.306605	-0.620992	3.133212	O	3.729653	-0.775090	3.065888
Cr	3.174534	-0.750969	0.063312	Cr	3.278784	-0.746582	0.051739
Cr	-4.173075	0.049569	-0.069919	Cr	-4.170250	0.059330	-0.128179
C	2.916993	1.218273	0.007901	C	2.817100	1.157864	0.172910
C	-4.028301	0.830974	-1.827820	C	-3.693118	1.153822	-1.620087
O	2.281688	2.259318	0.034137	O	2.101667	2.124949	0.379818

O	-3.951556	1.296843	-2.883665	O	-3.402657	1.813976	-2.517793

Table S20. Optimized coordinates for the $(Me_2C{=}N)_2Cr_2(CO)_{10}$ structure **10S-6.**

	M06-L				B3PW91-D3		
	x	y	z		x	y	z
C	0.725949	-2.019467	-1.245555	C	0.864442	-1.955948	-1.235063
C	2.738228	0.492976	0.845982	C	2.662446	0.470316	1.041730
C	-2.190252	-1.830843	-0.997713	C	-2.118384	-1.788430	-1.151147
C	-2.772517	0.572802	-0.863618	C	-2.693622	0.591006	-0.993183
O	0.440349	-2.945834	-1.879302	O	0.647723	-2.872894	-1.901653
O	3.508481	1.069002	1.492520	O	3.394177	1.009580	1.754300
O	-2.642720	-2.653106	-1.680358	O	-2.551781	-2.597476	-1.854133
O	-3.600358	1.111823	-1.472804	O	-3.487215	1.147704	-1.624788
C	0.406889	3.002697	2.381775	C	0.172266	2.894444	2.609743
C	-1.770698	3.150245	1.073611	C	-1.967232	3.063062	1.245273
H	-2.454471	3.307933	1.915987	H	-2.642215	3.171495	2.105295
H	-1.444322	4.143425	0.747400	H	-1.654091	4.075241	0.961102
H	-0.062230	3.307492	3.323555	H	-0.375384	3.188385	3.514614
H	0.752203	3.924856	1.899797	H	0.561569	3.819289	2.163896
C	1.994216	-1.983324	1.015409	C	1.956336	-1.977387	1.054507
C	-0.485927	0.303985	-1.690666	C	-0.390208	0.335270	-1.660574
O	2.319614	-2.825138	1.745617	O	2.264965	-2.848598	1.750023
O	-0.779649	0.096940	-2.851116	O	-0.614346	0.147033	-2.842188
H	1.258228	2.367605	2.625237	H	0.996612	2.246816	2.906902
H	-2.330868	2.670939	0.270264	H	-2.524972	2.611627	0.424740
C	2.204812	2.866436	-1.211045	C	2.336955	2.897516	-1.240467
H	3.015306	2.139446	-1.138146	H	3.033467	2.205298	-0.767915
H	2.489020	3.652678	-1.914937	H	2.814122	3.333618	-2.127106
H	2.089458	3.342688	-0.228227	H	2.132475	3.729749	-0.553166
C	-0.051007	3.067472	-2.383719	C	0.151890	3.041434	-2.530293
H	-0.119114	4.059881	-1.923972	H	0.174974	4.094626	-2.225749
H	0.313603	3.221340	-3.405160	H	0.530254	2.991923	-3.559937
H	-1.044253	2.621026	-2.452772	H	-0.876884	2.677918	-2.545014
C	0.923240	2.220201	-1.628709	C	1.054880	2.230232	-1.640529
C	-0.611519	2.322519	1.521247	C	-0.783405	2.236205	1.652376
N	0.698609	0.988690	-1.315828	N	0.778590	1.037852	-1.239662
N	-0.536663	1.080980	1.180054	N	-0.633470	1.040476	1.201715
O	0.718128	-0.033712	2.772498	O	0.560031	-0.138697	2.779928
C	0.504503	0.206422	1.598110	C	0.408666	0.151541	1.603813
C	-2.831256	-0.393989	1.416912	C	-2.910602	-0.428007	1.212228
O	-3.644353	-0.295007	2.239990	O	-3.787822	-0.375945	1.964760
Cr	-1.492584	-0.471024	0.090788	Cr	-1.489613	-0.461802	-0.000501
Cr	1.478066	-0.600534	-0.136643	Cr	1.489169	-0.563104	-0.058666
C	-0.936666	-1.968796	1.166327	C	-1.057053	-1.974439	1.063912
C	3.474686	-1.418348	-1.170893	C	3.584356	-1.316343	-0.985252
O	3.050494	-0.482008	-1.742341	O	3.178409	-0.386438	-1.564271

O	-0.767965	-2.927406	1.796194	O	-0.954828	-2.949188	1.676214

Table S21. Optimized coordinates for the $(Me_2C{=}N)_2Cr_2(CO)_{10}$ structure **10S-7.**

	M06-L				B3PW91-D3		
	x	y	z		x	y	z
C	-1.105945	0.225479	-1.775018	C	-0.014022	-0.555403	-0.293840
C	1.895837	-0.467114	-0.826485	C	3.385436	0.266497	-1.423670
C	-3.435714	0.698447	-0.572853	C	-3.154266	-0.409764	1.926366
C	-0.202433	0.892997	1.492153	C	-3.507381	0.336209	-1.782359
O	-1.470820	0.774300	-2.749703	O	-1.177802	-0.497115	-0.217018
O	2.958539	-1.031283	-0.839681	O	4.393952	-0.078524	-1.969432
O	-4.486779	0.595137	-1.046040	O	-3.119857	-0.463066	3.082695
O	0.569309	0.962701	2.359284	O	-3.705469	0.723880	-2.851582
C	3.821078	2.748199	1.667899	C	0.598855	2.171727	1.758514
C	4.757503	1.650207	-0.443232	C	2.017892	3.904109	0.536735
H	5.437393	2.501803	-0.544334	H	1.416948	4.399437	-0.237508
H	5.314132	0.859754	0.071200	H	1.863314	4.434499	1.480976
H	4.290009	3.718001	1.470140	H	-0.317147	2.760910	1.616405
H	4.529604	2.181184	2.280819	H	1.025251	2.486059	2.720059
C	0.422119	-1.312642	-2.399733	C	1.487837	-0.821842	-2.220213
C	-2.164818	-0.779455	1.163661	C	-3.107402	1.615471	0.414156
O	0.707004	-1.538098	-3.506561	O	1.301723	-1.034104	-3.346753
O	-2.504635	-1.723067	1.762593	O	-2.988340	2.750736	0.649591
H	2.896816	2.900504	2.224088	H	0.355249	1.109194	1.800987
H	4.478707	1.284486	-1.432328	H	3.060583	3.987745	0.219201
C	2.241389	-3.543634	1.311361	C	5.118017	-0.903331	1.882357
H	1.927419	-4.546686	1.621905	H	5.552235	-1.855494	2.214415
H	2.394570	-3.527783	0.231484	H	5.364858	-0.745534	0.829957
H	3.195950	-3.338720	1.808756	H	5.574767	-0.109847	2.488440
C	0.908046	-2.413343	3.199821	C	3.124748	-1.164677	3.503009
H	1.806285	-2.102139	3.744874	H	3.539595	-0.411731	4.185892
H	0.110679	-1.693501	3.388148	H	2.033152	-1.134286	3.545988
H	0.610413	-3.389022	3.598990	H	3.469080	-2.146178	3.854396
C	1.207330	-2.527845	1.726217	C	3.618106	-0.923042	2.091166
C	3.565604	2.033990	0.380380	C	1.567287	2.475846	0.661654
N	0.604630	-1.805652	0.883018	N	2.810161	-0.761124	1.135679
N	2.352478	1.791167	0.044000	N	1.959264	1.516403	-0.116499
C	-1.944894	3.070759	-0.830740	C	-3.349643	-2.584386	0.366256
O	-0.843893	2.824079	-0.560012	O	-3.327704	-2.311977	-0.762916
C	1.922029	1.111681	-1.051062	C	2.888560	1.702259	-1.177857
O	1.556938	1.552125	-2.122094	O	3.081163	2.684703	-1.854629
Cr	-0.054152	-1.004416	-0.636064	Cr	1.837672	-0.635998	-0.383099
Cr	-1.733627	0.814347	0.328016	Cr	-3.286524	-0.166231	0.053025
C	-2.623827	1.684569	1.735723	C	-5.110875	-0.035446	0.238424
C	-0.920769	-2.640675	-0.898596	C	2.030515	-2.486551	-0.508924
O	-3.171889	2.163117	2.641916	O	-6.262643	0.068497	0.357202

O	-1.418806	-3.675831	-1.044290	O	2.165802	-3.634814	-0.557447

Table S22. Optimized coordinates for the $(Me_2C{=}N)_2Cr_2(CO)_{10}$ structure **10S-8.**

	M06-L				B3PW91-D3		
	x	y	z		x	y	z
C	-3.026012	-1.627790	0.150345	C	-3.063353	-1.686464	0.122578
C	-1.188592	-0.999677	-1.201746	C	-1.195882	-1.159614	-1.206594
C	2.485764	-0.081740	1.956865	C	2.492605	0.055535	1.964393
C	1.622556	1.239875	-1.575691	C	1.657108	1.118089	-1.616377
O	-3.873176	-2.292740	0.670375	O	-3.904964	-2.330775	0.675145
O	-0.251724	-1.021308	-1.945784	O	-0.231515	-1.272959	-1.905008
O	2.686139	-0.509710	3.011313	O	2.710341	-0.310736	3.033960
O	1.345317	1.626562	-2.626434	O	1.408474	1.424935	-2.695025
C	-4.734774	1.902152	-0.916957	C	-4.752173	1.958039	-0.814821
C	-2.630964	2.436335	-2.300697	C	-2.674612	2.701211	-2.155764
H	-3.045914	1.956495	-3.193631	H	-3.157511	2.437884	-3.105295
H	-2.878864	3.502044	-2.349942	H	-2.870489	3.764590	-1.965945
H	-5.217729	1.415651	-1.770979	H	-5.280706	1.600605	-1.707606
H	-5.052262	2.950343	-0.905019	H	-5.040770	3.004108	-0.649883
C	-0.571006	-1.578787	1.234181	C	-0.689364	-1.628672	1.269404
C	2.083710	2.480900	0.770994	C	1.903032	2.506499	0.626485
O	0.099714	-2.505369	1.465104	O	-0.097731	-2.571529	1.601953
O	2.029381	3.589141	1.097674	O	1.743687	3.622932	0.861469
H	-5.062198	1.410171	-0.000172	H	-5.056832	1.354512	0.043320
H	-1.546588	2.313085	-2.307981	H	-1.598119	2.540019	-2.246678
C	1.930516	-3.285353	-1.332254	C	2.299641	-3.323048	-1.059810
H	2.504807	-4.166252	-1.027366	H	2.935104	-4.126117	-0.665608
H	1.018606	-3.223012	-0.740692	H	1.374754	-3.272038	-0.485238
H	1.664169	-3.409714	-2.386956	H	2.061579	-3.561630	-2.103743
C	4.132902	-2.070403	-1.831747	C	4.423157	-1.987691	-1.649416
H	4.023861	-2.310419	-2.894325	H	4.342932	-2.321623	-2.691493
H	4.646665	-1.113170	-1.733284	H	4.863548	-0.988430	-1.625574
H	4.750995	-2.856304	-1.385395	H	5.089050	-2.688573	-1.130055
C	2.774850	-2.046010	-1.173772	C	3.051809	-2.009472	-1.004401
C	-3.242261	1.800720	-1.081488	C	-3.258781	1.859272	-1.042019
N	2.350260	-1.046480	-0.536480	N	2.545896	-0.996339	-0.457040
N	-2.534031	1.168305	-0.245935	N	-2.536008	1.099615	-0.341069
C	3.992852	0.945757	0.041116	C	3.946243	1.147791	0.114033
O	5.135173	1.113660	-0.029727	O	5.065124	1.425081	0.094805
C	-2.630634	-1.417919	-1.300204	C	-2.644015	-1.609023	-1.351237
O	-3.400198	-0.973631	-2.151677	O	-3.385198	-1.297661	-2.269076
Cr	-1.627184	-0.079658	0.702511	Cr	-1.650279	-0.141573	0.607895
Cr	2.119310	0.632882	0.208368	Cr	2.129032	0.652777	0.194690
C	0.235725	0.856127	0.906049	C	0.210564	0.744822	0.821436
C	-2.534888	-0.328633	2.366224	C	-2.636000	-0.223588	2.224870
O	-0.474691	1.475203	1.661634	O	-0.512468	1.378681	1.555300

O	-3.112014	-0.495859	3.353055	O	-3.250962	-0.257623	3.196719

Table S23. Optimized coordinates for the $(Me_2C{=}N)_2Fe_2(CO)_7$ structure **7S-1.**

	M06-L				B3PW91-D3		
	x	y	z		x	y	z
C	2.420234	1.477447	-0.694831	C	2.522079	-1.124519	0.672128
C	1.985779	0.401155	1.785997	C	2.363940	1.477391	0.784354
C	-2.464680	-1.483836	0.118012	C	-2.260952	-0.371012	-1.313080
C	-0.033679	-2.333731	0.657340	C	-1.922242	-1.354952	1.328255
O	3.057245	2.326885	-1.167536	O	3.321608	-1.883603	1.032263
O	2.326629	0.509978	2.888686	O	3.066728	2.298346	1.204878
O	-3.472304	-2.042776	-0.050279	O	-2.874917	-0.525565	-2.289297
O	0.473466	-3.372272	0.759858	O	-2.277283	-2.148530	2.092401
C	-1.974954	3.409365	-0.606440	C	0.798220	3.582168	-1.092118
C	-3.527245	1.393781	-0.469277	C	-1.721594	3.369462	-0.753266
H	-3.812389	0.712374	0.334348	H	-2.103872	3.605716	0.246969
H	-4.281423	2.177662	-0.566121	H	-1.678141	4.298571	-1.329735
H	-2.399026	4.041533	0.181296	H	0.862636	4.478047	-0.459748
H	-2.531740	3.626429	-1.523582	H	0.619475	3.927511	-2.118726
C	2.493537	-1.178551	-0.314051	C	0.672541	0.016319	1.979617
C	-0.999478	-0.576592	2.285066	C	-2.732048	0.880781	0.749818
O	3.198916	-2.046228	-0.619987	O	0.539160	-0.030994	3.131748
O	-1.075545	-0.447641	3.433893	O	-3.700550	1.411588	1.100195
H	-0.927842	3.685692	-0.715762	H	1.749159	3.050383	-1.058117
H	-3.524817	0.815543	-1.401859	H	-2.431753	2.685540	-1.229876
C	1.025610	-0.179639	-3.410033	C	1.408199	-3.216806	-1.341828
H	0.585316	0.348096	-4.262665	H	1.244614	-3.868002	-2.207722
H	1.769181	0.459385	-2.931221	H	2.141270	-2.450065	-1.601266
H	1.537026	-1.061210	-3.814761	H	1.827113	-3.834803	-0.537086
C	-1.217149	-1.313487	-3.042961	C	-1.109181	-3.488610	-1.090255
H	-0.889567	-2.199805	-3.597665	H	-0.921315	-4.486370	-0.673412
H	-1.937420	-1.621572	-2.284038	H	-2.004420	-3.065904	-0.631262
H	-1.716162	-0.661335	-3.769415	H	-1.292182	-3.615540	-2.165553
Fe	-0.863629	-0.762060	0.488492	Fe	0.100839	-2.608909	-0.890271
Fe	1.410109	0.239137	0.090792	Fe	-0.357291	2.722843	-0.648750
C	-0.035264	-0.611719	-2.435868	C	0.024783	-1.442304	-0.384149
C	-2.167980	1.974065	-0.234666	C	-0.211709	1.527720	-0.223280
N	0.084238	-0.410176	-1.182831	N	1.998328	0.291048	-1.564802
N	-1.232878	1.224314	0.239016	N	2.486177	0.409783	-2.605862
C	0.120335	1.697647	0.540324	C	1.233649	0.127752	0.167782
O	0.267901	2.761905	1.108504	O	-1.223504	-0.070353	0.155889

Table S24. Optimized coordinates for the $(Me_2C{=}N)_2Fe_2(CO)_7$ structure **7S-2.**

	M06-L				B3PW91-D3		
	x	y	z		x	y	z
C	-1.804023	-1.465133	-1.629817	C	-1.838192	1.731935	1.181515
C	0.866254	-0.227377	-1.435218	C	-2.506280	-0.935296	1.081327
C	2.685020	1.469595	0.021280	C	2.524464	-0.334166	-1.124044
C	0.870214	0.454042	2.179592	C	2.506255	0.935271	1.081401
O	-2.664261	-1.595720	-2.406934	O	-2.152549	2.680744	1.758872
O	1.215322	-0.061895	-2.558755	O	-3.302332	-1.620926	1.565263
O	3.622664	2.127261	-0.200717	O	3.327818	-0.379286	-1.960437
O	0.534838	0.504871	3.293665	O	3.302296	1.620889	1.565371
C	0.581597	3.409232	-1.264874	C	-1.721286	-2.916667	-1.660001
C	-1.852929	2.718546	-1.596844	C	0.783459	-3.342291	-1.546306
H	-2.360920	3.592248	-1.170738	H	0.810564	-4.163339	-0.816624
H	-1.668266	2.951163	-2.649289	H	0.653638	-3.793094	-2.537459
H	0.205722	4.386410	-1.576840	H	-1.856632	-3.965014	-1.364673
H	1.142944	2.983034	-2.105422	H	-1.782388	-2.881585	-2.756136
C	0.350154	-2.672036	-0.931536	C	-0.000027	-0.000018	1.906306
C	2.415206	-1.003654	0.627882	C	1.838165	-1.731960	1.181519
O	0.916200	-3.621522	-1.285813	O	-0.000034	-0.000030	3.074775
O	3.122770	-1.914816	0.772456	O	2.152511	-2.680778	1.758866
H	1.276520	3.529191	-0.432928	H	-2.532879	-2.317822	-1.244348
H	-2.518126	1.855894	-1.532971	H	1.740099	-2.815930	-1.503791
C	-3.324185	-0.112337	1.293218	C	-0.783425	3.342327	-1.546246
H	-3.295085	-0.956491	0.601692	H	-0.653587	3.793149	-2.537388
H	-3.140434	-0.496158	2.304385	H	-1.740068	2.815969	-1.503755
H	-4.323703	0.332978	1.297492	H	-0.810538	4.163362	-0.816549
C	-2.475929	2.273732	1.558185	C	1.721320	2.916696	-1.659912
H	-2.822157	2.165443	2.589802	H	1.856658	3.965042	-1.364579
H	-1.554554	2.858254	1.554111	H	2.532905	2.317850	-1.244243
H	-3.251849	2.838847	1.026619	H	1.782444	2.881615	-2.756046
C	-2.307393	0.920010	0.950989	C	0.366517	2.420161	-1.216803
C	-0.544860	2.499271	-0.914831	C	-0.366492	-2.420136	-1.216862
N	-0.308049	1.535732	-0.081975	N	0.195400	1.318910	-0.598455
N	-1.311684	0.584429	0.184812	N	-0.195388	-1.318898	-0.598488
Fe	1.330317	0.375140	0.404200	Fe	-2.524436	0.334191	-1.124092
Fe	-0.530945	-1.222361	-0.405826	Fe	-3.327768	0.379333	-1.960505
O	-1.069726	-2.500825	2.252146	O	-1.238116	0.188932	0.216235
C	-0.885474	-1.989397	1.223329	C	1.238112	-0.188938	0.216254

Table S25. Optimized coordinates for the $(Me_2C{=}N)_2Fe_2(CO)_7$ structure **7T-3.**

	M06-L				B3PW91-D3		
	x	y	z		x	y	z
C	-2.894307	-1.017188	-0.628819	C	-0.672536	0.016297	1.979616
C	-2.408258	1.657048	-0.790584	C	-1.998333	0.291088	-1.564795
C	2.702358	-0.587397	0.976390	C	2.732032	0.880774	0.749846
C	1.710444	-1.741518	-1.350189	C	-0.539156	-0.031031	3.131746
O	-3.802935	-1.691361	-0.879577	O	-2.486178	0.409848	-2.605853
O	-2.990859	2.596959	-1.142712	O	3.700527	1.411582	1.100238
O	3.496560	-0.764068	1.805328	O	1.109160	-3.488607	-1.090284
O	1.902126	-2.679103	-2.009984	O	-1.408219	-3.216784	-1.341864
C	-0.151401	3.452327	1.335319	C	-1.244637	-3.867956	-2.207776
C	2.299084	2.814742	1.226676	C	-1.827130	-3.834803	-0.537137
H	2.591506	3.638456	0.564595	H	1.292173	-3.615512	-2.165583
H	2.402520	3.182779	2.253367	H	0.921282	-4.486376	-0.673468
H	0.118897	4.432666	0.927737	H	-2.522088	-1.124512	0.672115
H	-0.178964	3.560895	2.426027	H	2.260962	-0.371012	-1.313068
C	-0.750292	0.076804	-1.965804	C	-3.321618	-1.883601	1.032241
C	2.402691	0.786288	-1.376295	C	2.874940	-0.525556	-2.289279
O	-0.544334	0.014209	-3.104924	O	2.004396	-3.065918	-0.631271
O	3.025150	1.502840	-2.046229	O	-2.141289	-2.450035	-1.601278
H	-1.151129	3.188111	0.985757	H	1.721620	3.369430	-0.753303
H	2.991855	1.985694	1.071310	H	2.431776	2.685472	-1.229866
C	-1.673231	-2.689125	1.753535	C	1.678181	4.298505	-1.329827
H	-1.558912	-3.043317	2.783204	H	2.103891	3.605742	0.246921
H	-2.429847	-1.903818	1.732159	H	-0.798193	3.582175	-1.092117
H	-2.045995	-3.538154	1.168092	H	-0.619478	3.927470	-2.118746
C	0.800110	-3.142109	1.575011	C	-1.749148	3.050422	-1.058056
H	0.497218	-4.191337	1.495832	H	-0.862556	4.478083	-0.459782
H	1.676524	-2.974739	0.947833	H	0.357308	2.722833	-0.648758
H	1.087743	-2.972864	2.619510	H	-0.100856	-2.608901	-0.890294
Fe	1.425383	-0.286754	-0.314720	Fe	0.211714	1.527718	-0.223270
Fe	-1.536307	0.184079	-0.229889	Fe	-0.024795	-1.442302	-0.384161
C	-0.345192	-2.233918	1.206229	C	2.277277	-2.148556	2.092388
C	0.871023	2.413194	0.941407	C	1.922237	-1.354975	1.328247
N	-0.184101	-1.183413	0.497588	N	-2.363926	1.477398	0.784384
N	0.535075	1.306175	0.413043	N	-3.066700	2.298354	1.204928
C	-1.874351	0.583269	1.530560	C	-1.233652	0.127758	0.167785
O	-2.048992	0.796941	2.654269	O	1.223500	-0.070364	0.155892

Table S26. Optimized coordinates for the $(Me_2C{=}N)_2Fe_2(CO)_7$ structure **7T-4.**

	M06-L				B3PW91-D3		
	x	y	z		x	y	z
C	-1.881858	-2.416354	0.068551	C	-3.342565	-0.245438	-0.010261
C	-1.980934	0.004969	1.490634	C	-2.013034	1.151391	1.470607
C	0.924690	2.372824	1.214235	C	1.914532	-0.093291	-1.490605
C	1.170326	1.582422	-1.402573	C	1.938436	-0.060345	1.472878
O	-2.013713	-3.559600	0.232535	O	-4.486393	-0.463496	-0.013915
O	-2.286688	0.399099	2.544826	O	-2.247536	1.722531	2.453639
O	1.397791	3.199473	1.883192	O	2.449102	0.162455	-2.488662
O	1.771873	1.874933	-2.353359	O	2.490491	0.222973	2.453426
C	0.878010	-0.411920	3.076938	C	-2.258924	-2.759607	-1.287979
C	2.163457	-2.356260	2.014357	C	-2.268169	-2.756714	1.278900
H	3.239883	-2.248586	1.838187	H	-3.363465	-2.756281	1.295061
H	2.021054	-2.767691	3.014911	H	-1.932815	-3.802138	1.331037
H	1.651388	-0.283185	3.841703	H	-1.873329	-2.225822	-2.160576
H	0.114441	-1.068372	3.511451	H	-3.354094	-2.760609	-1.311329
C	-3.260443	-0.204030	-0.890121	C	-2.013283	1.203173	-1.442155
C	-1.226934	2.115167	-0.342564	C	2.046103	-2.053680	0.011428
O	-4.290035	0.079395	-1.342407	O	-2.246398	1.811767	-2.402306
O	-2.106396	2.822967	-0.630461	O	2.785394	-2.945680	0.015399
H	0.418763	0.554710	2.865129	H	-1.921825	-3.804664	-1.336214
H	1.795499	-3.064814	1.264470	H	-1.887768	-2.221842	2.153069
C	3.341826	-1.635277	-2.133508	C	-0.461512	3.621932	-0.022578
H	2.660566	-2.458089	-2.351496	H	-0.407503	4.122178	-0.998634
H	3.338494	-0.943731	-2.984470	H	-1.487684	3.291110	0.142825
H	4.364363	-2.014764	-2.036158	H	-0.191071	4.363930	0.738580
C	3.839215	0.158533	-0.377699	C	1.968578	2.899054	0.030230
H	4.199341	0.787855	-1.196695	H	2.195138	3.344137	1.008593
H	3.360975	0.789312	0.375216	H	2.648062	2.061575	-0.134919
H	4.723533	-0.310677	0.069677	H	2.150140	3.672432	-0.726477
C	2.921662	-0.903450	-0.901629	C	0.519925	2.476790	0.002457
C	1.457705	-1.046940	1.864339	C	-1.746651	-2.142962	-0.003377
N	1.319911	-0.488729	0.699688	N	0.172881	1.247490	0.001280
N	1.786781	-1.241841	-0.390610	N	-0.587675	-1.498743	0.000201
Fe	0.203555	1.144721	0.095057	Fe	4.804260	0.334343	-0.041789
Fe	-1.638434	-0.640717	-0.176075	Fe	5.369643	-0.655935	0.003490
O	-0.239606	-0.759308	-2.813673	O	0.917979	-0.558714	0.002695
C	-0.712407	-0.662643	-1.753336	C	-1.547596	0.149962	-0.002131

Table S27. Optimized coordinates for the $(Me_2C{=}N)_2Fe_2(CO)_7$ structure **7T-5.**

	M06-L				B3PW91-D3		
	x	y	z		x	y	z
C	-3.130691	0.895007	-0.080854	C	-2.367518	0.704100	-0.955698
C	-1.087131	-0.846020	-1.665002	C	-0.654181	-1.006431	-1.492565
C	3.748129	0.169913	-0.168460	C	2.046435	-1.081603	-1.729202
C	1.956793	-0.644021	1.776589	C	2.938223	-0.454130	0.501797
O	-3.889358	1.782176	-0.071442	O	-2.927155	1.429351	-1.679963
O	-0.615627	-1.113366	-2.697641	O	-0.698831	-1.439261	-2.576350
O	4.854585	0.538963	-0.213959	O	2.593158	-1.434958	-2.686918
O	1.896822	-0.700915	2.940688	O	4.009141	-0.494019	0.960576
C	1.559627	1.729541	-2.598171	C	0.963610	3.131695	-2.075727
C	-0.699085	2.637921	-1.802939	C	-0.641677	3.535421	-0.093080
H	-0.484538	3.416096	-2.536977	H	-0.188054	4.503058	0.155011
H	-1.503668	2.004225	-2.200405	H	-1.480122	3.730589	-0.772482
H	1.977810	2.725459	-2.776232	H	1.403083	4.129793	-1.958809
H	1.118234	1.395944	-3.544166	H	0.174295	3.209290	-2.835133
C	-3.181190	-1.615879	-0.463190	C	-2.471232	-1.844998	-0.094851
C	2.266500	-2.190541	-0.590631	C	1.024088	-2.140873	0.517609
O	-4.017927	-2.385617	-0.696804	O	-3.118366	-2.796673	-0.245544
O	2.441718	-3.295938	-0.906704	O	0.891770	-3.197885	0.992247
H	2.361807	1.040168	-2.321394	H	1.725202	2.429213	-2.424324
H	-1.088139	3.089519	-0.886739	H	-1.026402	3.065039	0.814800
C	-1.306731	1.648330	2.567534	C	0.467590	-0.140457	2.927355
H	-2.172805	1.039722	2.309379	H	-0.354685	-0.803766	2.639270
H	-0.821442	1.217963	3.452585	H	1.277730	-0.763963	3.319158
H	-1.627901	2.657869	2.843542	H	0.121095	0.515019	3.738795
C	0.879879	2.601718	1.657046	C	2.007398	1.724705	2.134735
H	0.926300	2.975884	2.680241	H	2.829150	1.261694	2.688581
H	1.804281	2.060893	1.420829	H	2.421503	2.228445	1.258713
H	0.834719	3.460530	0.977400	H	1.541113	2.475927	2.789453
C	-0.308296	1.713554	1.469183	C	0.971481	0.690914	1.779120
C	0.499724	1.786403	-1.556617	C	0.361821	2.667909	-0.786820
N	0.636451	1.028901	-0.511627	N	0.722154	1.516304	-0.355342
N	-0.458015	0.989025	0.399893	N	0.098415	0.895223	0.751332
Fe	2.027280	-0.448727	-0.046403	Fe	-3.488629	0.520255	2.190952
Fe	-1.911581	-0.434704	-0.099794	Fe	-2.661785	0.172449	1.455899
C	-1.451893	-1.380946	1.387515	C	1.238108	-0.454835	-0.172677
O	-1.177176	-1.984110	2.342507	O	-1.412363	-0.349653	0.157827

Table S28. Optimized coordinates for the $(Me_2C{=}N)_2Fe_2(CO)_7$ structure **7S-6.**

	M06-L				B3PW91-D3		
	x	y	z		x	y	z
C	2.112944	-0.722597	1.334666	C	-2.043744	0.998072	1.296326
C	2.393230	0.548085	-1.135622	C	-2.358938	-0.145857	-1.196652
C	-0.386352	0.492271	1.931340	C	0.117708	-0.544543	1.910461
C	-2.665302	-0.620021	-0.803554	C	2.790339	0.126062	-0.667617
O	2.666118	-1.435734	2.054948	O	-2.548367	1.751763	2.002029
O	3.124175	0.640327	-2.028187	O	-3.070060	-0.130453	-2.101829
O	-0.109223	0.139860	3.056218	O	-0.142065	-0.223762	3.049614
O	-3.664815	-0.718958	-1.392069	O	3.832931	0.044086	-1.167913
C	-0.158737	-3.286789	-1.868666	C	0.711982	3.188172	-1.922461
C	2.269764	-2.681492	-1.394819	C	-1.770201	3.043883	-1.376341
H	2.433525	-3.693336	-1.009070	H	-1.735909	4.071913	-0.993320
H	2.573375	-2.693621	-2.447820	H	-2.067926	3.107174	-2.431179
H	-0.172574	-4.206434	-1.271854	H	0.874294	4.135860	-1.391330
H	0.137804	-3.570650	-2.883976	H	0.443530	3.440393	-2.956516
C	1.591991	1.895679	1.054653	C	-1.926155	-1.620143	0.972079
C	-1.568842	-1.877138	1.138901	C	1.886359	1.565741	1.159204
O	1.874326	2.898128	1.557473	O	-2.413040	-2.548234	1.448866
O	-1.824082	-2.835584	1.740034	O	2.334944	2.426822	1.783637
H	-1.167211	-2.867668	-1.884707	H	1.640799	2.613404	-1.916033
H	2.919348	-1.992274	-0.852263	H	-2.535936	2.494023	-0.826170
C	0.335675	3.192240	-1.805803	C	-0.786874	-3.065690	-1.877839
H	0.109118	4.130635	-1.286699	H	-0.630341	-4.067641	-1.456423
H	1.356527	2.896512	-1.555232	H	-1.756568	-2.692981	-1.542408
H	0.275034	3.404228	-2.878527	H	-0.804540	-3.179113	-2.969698
C	-2.083651	2.372641	-1.879094	C	1.716369	-2.572887	-1.989656
H	-2.268356	1.820985	-2.807869	H	2.044703	-1.878199	-2.772714
H	-2.794001	2.021249	-1.127568	H	2.451596	-2.546447	-1.179778
H	-2.268540	3.431169	-2.079233	H	1.693849	-3.580956	-2.415542
Fe	-1.178132	-0.443606	0.120107	Fe	1.243288	0.252137	0.121475
Fe	1.178637	0.351163	0.208134	Fe	-1.206975	-0.168957	0.206844
C	-0.668059	2.145482	-1.418630	C	0.352790	-2.165020	-1.472338
C	0.818875	-2.307899	-1.274907	C	-0.403997	2.409283	-1.260244
N	-0.360772	1.113177	-0.738444	N	0.210457	-1.130086	-0.744744
N	0.389463	-1.250825	-0.709376	N	-0.150151	1.314353	-0.664599
C	-1.684890	0.937176	1.346029	C	1.439886	-1.115681	1.414265
O	-2.464985	1.832014	1.553874	O	2.088537	-2.067511	1.766232

Table S29. Optimized coordinates for the $(Me_2C=N)_2Fe_2(CO)_7$ structure **7S-7.**

	M06-L				B3PW91-D3		
	x	y	z		x	y	z
C	-1.377136	0.300231	1.772670	C	-1.690624	0.221305	1.795271
C	-1.768100	0.472122	-1.813266	C	-1.668412	0.626440	-1.788854
C	3.484546	-0.204552	-0.518430	C	3.441841	-0.421413	-0.471991
O	-1.112963	0.384651	2.898350	O	-1.652216	0.209430	2.949089
O	-1.679459	0.639135	-2.954188	O	-1.537561	0.840051	-2.913209
O	4.601532	0.053731	-0.665608	O	4.567228	-0.239559	-0.630037
C	-0.916206	-2.581214	1.128443	C	-1.146589	-2.585430	1.052230
C	-1.814552	-2.572691	-1.229584	C	-1.902192	-2.427568	-1.350596
H	-1.268141	-3.503461	-1.440789	H	-1.396313	-3.385157	-1.549921
H	-2.817542	-2.853166	-0.891975	H	-2.941978	-2.654006	-1.089333
H	-0.377639	-3.518125	0.928914	H	-0.640693	-3.534945	0.823012
H	-1.894506	-2.856838	1.535603	H	-2.160474	-2.835070	1.384756
C	-3.475119	-0.300117	0.216753	C	-3.551871	0.020919	0.001966
C	1.902034	-2.353788	-0.598125	C	1.769507	-2.436261	-0.564853
O	-4.565190	-0.679914	0.351081	O	-4.693579	-0.174104	-0.007607
O	1.978554	-3.487747	-0.815960	O	1.845089	-3.559872	-0.806032
H	-0.361400	-2.029031	1.890314	H	-0.612438	-2.107598	1.876630
H	-1.893586	-2.014911	-2.164150	H	-1.882736	-1.842982	-2.272774
C	1.249654	3.555014	-1.191696	C	1.703627	3.463577	-1.203667
H	2.156720	4.167491	-1.247559	H	2.667761	3.984422	-1.276642
H	1.012334	3.179313	-2.188130	H	1.423256	3.083842	-2.189033
H	0.439484	4.205196	-0.845226	H	0.958402	4.196597	-0.869882
C	1.661556	2.803257	1.218741	C	2.184713	2.734169	1.205720
H	0.784942	3.350801	1.584880	H	1.451232	3.450000	1.598449
H	1.823654	1.928122	1.851309	H	2.234902	1.872168	1.874313
H	2.518496	3.479188	1.315123	H	3.158621	3.241626	1.199274
Fe	1.714145	-0.566150	-0.246860	Fe	1.664471	-0.657699	-0.200834
Fe	-1.788149	0.279938	0.005774	Fe	-1.806732	0.353622	-0.000409
C	1.458494	2.412817	-0.224977	C	1.804305	2.324691	-0.204165
C	-1.049095	-1.805326	-0.167015	C	-1.167926	-1.727335	-0.208844
N	1.440206	1.221386	-0.650662	N	1.570148	1.140786	-0.582584
N	-0.150472	-0.899377	-0.558830	N	-0.198959	-0.857951	-0.501080
O	1.949673	-0.752375	2.656996	O	1.821874	-0.836403	2.690402
C	1.848371	-0.680953	1.501341	C	1.762724	-0.773565	1.537364
C	-1.692469	2.059091	0.094206	C	-1.498719	2.090587	0.209629
O	-1.709487	3.221211	0.188331	O	-1.401008	3.235029	0.374125

Table S30. Optimized coordinates for the $(Me_2C{=}N)_2Fe_2(CO)_7$ structure **7T-8.**

	M06-L				B3PW91-D3		
	x	y	z		x	y	z
C	1.761452	-1.197682	1.656299	C	2.478192	-1.241339	1.499530
C	3.091416	0.201278	-0.299475	C	3.159501	-0.024801	-0.878741
C	-1.728229	1.426570	1.054776	C	-1.868712	1.238677	1.083467
C	-2.310479	-0.197288	-1.407547	C	-2.086069	0.021407	-1.375768
O	1.949858	-2.059515	2.410933	O	2.879187	-2.049506	2.223768
O	4.153697	0.273323	-0.767161	O	3.959794	-0.056386	-1.712698
O	-1.895533	2.505022	1.431927	O	-2.399539	2.047737	1.717584
O	-3.126363	-0.267521	-2.241675	O	-2.714107	0.051706	-2.345997
C	-0.258502	-3.276112	-1.762251	C	-0.673104	-3.112106	-1.435230
C	2.223198	-2.981980	-1.276524	C	1.840552	-3.085208	-1.208988
H	2.275001	-3.921468	-0.713513	H	1.764472	-4.136336	-0.902652
H	2.481100	-3.224110	-2.313078	H	2.039897	-3.082334	-2.289314
H	-0.378998	-4.180200	-1.153401	H	-0.815266	-4.026428	-0.843108
H	-0.004379	-3.607788	-2.774553	H	-0.520099	-3.427660	-2.475293
C	1.578823	1.429079	1.634389	C	2.503086	1.309724	1.429233
C	-1.508492	-1.678948	1.074467	C	-1.881038	-1.306672	1.013452
O	1.646092	2.319970	2.375654	O	2.922163	2.149738	2.105149
O	-1.547879	-2.756626	1.483916	O	-2.421681	-2.145117	1.598989
H	-1.210497	-2.739860	-1.781318	H	-1.581496	-2.511301	-1.374083
H	2.966211	-2.292669	-0.872204	H	2.687110	-2.624302	-0.698415
C	1.769122	3.211738	-1.331687	C	1.781735	3.108865	-1.210459
H	1.688708	4.156465	-0.780899	H	1.813410	4.021603	-0.599942
H	2.604859	2.641198	-0.923335	H	2.663517	2.507419	-0.985514
H	1.984612	3.474324	-2.372906	H	1.822112	3.427117	-2.260107
C	-0.731243	3.139810	-1.806199	C	-0.730346	3.080260	-1.453927
H	-0.529571	3.494725	-2.822371	H	-0.726756	3.074030	-2.552477
H	-1.594928	2.470310	-1.816877	H	-1.656478	2.620139	-1.106959
H	-0.981779	4.022587	-1.205763	H	-0.711927	4.132327	-1.141970
Fe	-1.007930	-0.090099	-0.145346	Fe	-1.018043	-0.013581	0.120937
Fe	1.451810	0.090396	0.434065	Fe	1.829606	0.012025	0.390830
C	0.476534	2.451437	-1.229431	C	0.495959	2.360460	-0.938222
C	0.834412	-2.412882	-1.191046	C	0.540622	-2.363958	-0.930399
N	0.373738	1.310429	-0.669824	N	0.451807	1.228694	-0.343944
N	0.564309	-1.290311	-0.650603	N	0.473733	-1.231324	-0.339941
C	-4.361027	-0.294302	1.300972	C	-5.459358	0.013263	0.401818
O	-5.233555	0.064551	0.652163	O	-6.596319	0.013476	0.505267

Table S31. Optimized coordinates for the $(Me_2C{=}N)_2Fe_2(CO)_7$ structure **7S-9.**

	M06-L				B3PW91-D3		
	x	y	z		x	y	z
C	3.123311	0.551413	-1.099837	C	-3.129994	0.592043	1.096870
C	1.295492	1.151975	1.403501	C	-1.504550	0.976362	-1.560349
C	-3.944666	0.089666	-0.388034	C	3.941496	0.256474	0.396944
C	-1.818196	-1.904572	0.736883	C	2.065566	-1.993623	-0.453797
O	3.767121	0.927806	-1.998522	O	-3.764751	1.028467	1.968400
O	0.811439	1.911186	2.143610	O	-1.154795	1.644378	-2.441529
O	-5.084075	0.252256	-0.609392	O	5.060577	0.499310	0.623629
O	-1.643721	-2.986861	1.145987	O	2.058423	-3.126207	-0.727499
C	-1.584960	2.969446	-0.257628	C	1.546886	3.037581	-0.096009
C	0.663004	2.700612	-1.455073	C	-0.653659	2.818249	1.175933
H	0.371628	3.629960	-1.949226	H	-0.407067	3.859778	1.398760
H	1.419652	2.949305	-0.700140	H	-1.529918	2.799132	0.515652
H	-1.700986	3.866594	-0.869691	H	1.944443	3.708888	0.675765
H	-1.301901	3.291258	0.752576	H	1.081275	3.670420	-0.864056
C	3.531463	-0.026393	1.324512	C	-3.658017	-0.231378	-1.182213
C	-2.559073	0.287602	1.674985	C	2.642893	0.048881	-1.703664
O	4.470327	-0.032418	2.007437	O	-4.640990	-0.333511	-1.783328
O	-2.730752	0.728812	2.740851	O	2.856091	0.281472	-2.821074
H	-2.535138	2.431759	-0.177080	H	2.363476	2.463357	-0.535687
H	1.139694	2.033988	-2.175437	H	-0.941210	2.305514	2.098471
C	1.094413	-1.949468	-2.326544	C	-1.009200	-1.658812	2.515369
H	2.114239	-1.630617	-2.111586	H	-2.045183	-1.445976	2.251172
H	0.925374	-2.911751	-1.826670	H	-0.773557	-2.684529	2.199442
H	0.968373	-2.122880	-3.399642	H	-0.879150	-1.619167	3.603864
C	-1.307752	-1.175997	-2.353168	C	1.353662	-0.773742	2.385128
H	-1.585654	-2.224943	-2.222165	H	1.670147	-1.815680	2.481709
H	-2.083552	-0.512129	-1.921308	H	2.126850	-0.167886	1.850493
H	-1.366801	-0.969816	-3.428143	H	1.392727	-0.334408	3.393021
C	0.086596	-0.965484	-1.849535	C	-0.055371	-0.713427	1.860086
C	-0.520477	2.079532	-0.795144	C	0.503158	2.147650	0.495933
N	-0.677187	0.801055	-0.613066	N	0.643431	0.863971	0.380423
N	0.414025	-0.036384	-1.003026	N	-0.422172	0.064869	0.892341
Fe	-2.270635	-0.310118	0.075868	Fe	2.312894	-0.287424	-0.031391
Fe	2.107075	-0.016985	0.276219	Fe	-2.165228	-0.075553	-0.255401
C	1.838318	-1.779827	0.645733	C	-1.867334	-1.838037	-0.507873
O	1.702778	-2.907003	0.897904	O	-1.720078	-2.970566	-0.700538

Table S32. Optimized coordinates for the $(Me_2C{=}N)_2Cr_2(CO)_7$ structure **9S-1.**

	M06-L				B3PW91-D3		
	x	y	z		x	y	z
C	3.448144	1.161140	0.272719	C	-3.343608	1.200899	-0.431259
C	2.025832	-0.450466	1.709305	C	-1.989035	-0.517758	-1.708654
C	-3.110872	0.950010	-0.948498	C	3.119051	0.900990	0.881179
C	-1.332557	-1.022711	-1.575640	C	1.355578	-1.025922	1.557419
O	4.214570	1.993292	0.562381	O	-4.060839	2.030793	-0.819940
O	1.786909	-0.448430	2.855700	O	-1.765949	-0.603746	-2.849130
O	-3.755527	1.726620	-1.528605	O	3.789194	1.664615	1.441371
O	-0.945883	-1.466335	-2.580017	O	0.993340	-1.473733	2.563982
C	-1.336497	2.562705	1.922271	C	1.442034	2.693368	-1.745296
C	0.922719	3.103274	0.895499	C	-0.802476	3.234220	-0.687973
H	0.603289	4.148819	0.929449	H	-0.418327	4.245359	-0.506460
H	1.523861	2.931861	1.797130	H	-1.327927	3.260012	-1.651609
H	-1.121073	3.520013	2.399310	H	1.262759	3.702095	-2.126722
H	-1.390772	1.796436	2.701932	H	1.466223	2.004178	-2.596511
C	3.900190	-1.308439	0.027507	C	-3.900919	-1.203994	-0.030749
C	-3.091272	0.147199	1.592867	C	2.979818	0.099720	-1.645140
O	4.890244	-1.923839	0.105415	O	-4.909496	-1.782933	-0.093635
O	-3.791940	0.336113	2.501470	O	3.630766	0.266106	-2.589134
H	-2.321239	2.617037	1.449396	H	2.423918	2.665554	-1.262912
H	1.564603	2.947673	0.029506	H	-1.520659	2.976370	0.088026
C	1.807827	0.715414	-2.696553	C	-1.835978	0.581060	2.662096
H	2.339734	-0.138375	-2.230798	H	-2.696638	0.278770	2.023886
H	1.472789	0.342016	-3.669129	H	-1.506740	-0.296273	3.232304
H	2.535735	1.513142	-2.877694	H	-2.249889	1.312742	3.364410
C	-0.386067	2.054167	-2.545696	C	0.348247	1.953527	2.636704
H	-1.036232	1.427516	-3.169426	H	0.823988	1.326204	3.401608
H	-1.014719	2.577558	-1.823412	H	1.122882	2.347714	1.978083
H	0.083944	2.783224	-3.212504	H	-0.141928	2.785608	3.157148
C	0.649450	1.212008	-1.889932	C	-0.672578	1.150159	1.902385
C	-0.275304	2.214473	0.930064	C	0.355524	2.288540	-0.793368
N	-0.440081	1.167152	0.179918	N	0.463160	1.174444	-0.141806
N	0.657691	0.837273	-0.652925	N	-0.647389	0.827561	0.655051
C	1.512486	-2.037909	-0.398947	C	-1.571756	-1.986973	0.460247
O	1.154813	-3.134855	-0.540208	O	-1.251732	-3.084558	0.645853
O	-0.687292	-2.366618	1.879160	O	0.627515	-2.397401	-1.807300
C	-1.146015	-1.583408	1.161270	C	1.092296	-1.595498	-1.121463
Cr	2.329374	-0.326470	-0.087906	Cr	-2.307645	-0.283194	0.067181
Cr	-2.098620	-0.330211	0.033366	Cr	2.064605	-0.346741	-0.054558
C	-3.432916	-1.602000	-0.223617	C	3.383042	-1.621456	0.163055
O	-4.269915	-2.394733	-0.392730	O	4.213384	-2.420293	0.308930

Table S33. Optimized coordinates for the $(Me_2C{=}N)_2Cr_2(CO)_7$ structure **9S-2.**

	M06-L				B3PW91-D3		
	x	y	z		x	y	z
C	0.196264	-0.546632	-2.059156	C	0.139872	-0.736198	-1.978302
C	2.820002	-0.768398	0.573546	C	2.808615	-0.803972	0.494619
C	-1.695969	-1.927289	-0.391083	C	-1.801601	-1.875551	-0.191032
C	-1.703474	1.305667	1.175832	C	-1.663797	1.312562	1.137649
O	-0.134817	-0.694449	-3.171118	O	-0.190886	-1.024125	-3.061968
O	3.849209	-1.015920	1.054203	O	3.841856	-1.079992	0.941926
O	-1.817992	-3.074910	-0.413604	O	-1.966444	-3.010443	-0.102249
O	-1.791481	2.045612	2.074168	O	-1.739770	2.075329	2.012354
C	0.878558	3.876664	0.917824	C	1.009559	3.843909	0.876144
C	2.858685	2.304971	1.204542	C	2.997088	2.257703	1.015813
H	3.340145	3.244507	1.482794	H	3.504083	3.204416	1.224433
H	2.790662	1.685203	2.106615	H	2.987029	1.668310	1.941949
H	1.416043	4.638697	0.342578	H	1.449980	4.583670	0.194268
H	1.014222	4.128655	1.974632	H	1.301030	4.125578	1.895104
C	1.330984	-2.336084	-0.663872	C	1.287085	-2.359461	-0.554986
C	-2.372460	0.581820	-1.747753	C	-2.275858	0.558655	-1.797918
O	1.417457	-3.493288	-0.773433	O	1.362979	-3.516984	-0.602189
O	-2.895401	0.942088	-2.714824	O	-2.759547	0.896754	-2.786253
H	-0.176009	3.934969	0.654016	H	-0.074629	3.890739	0.773837
H	3.490063	1.772238	0.489553	H	3.565801	1.699004	0.268130
C	1.191546	-1.674409	2.967456	C	1.200931	-1.513924	3.007871
H	0.983848	-2.145458	3.931165	H	1.014888	-1.855053	4.030796
H	1.678866	-2.410348	2.319931	H	1.636642	-2.342546	2.438002
H	1.907371	-0.863055	3.136038	H	1.940663	-0.706792	3.041913
C	-1.309843	-1.271461	3.160065	C	-1.309361	-1.122584	3.198170
H	-1.182213	-0.735746	4.107873	H	-1.195633	-0.486991	4.086339
H	-2.191067	-0.884802	2.645941	H	-2.207868	-0.823976	2.655788
H	-1.484410	-2.322356	3.419079	H	-1.435564	-2.153041	3.555957
C	-0.069399	-1.155260	2.319904	C	-0.072358	-1.038195	2.339855
C	1.492441	2.543489	0.644603	C	1.588050	2.496630	0.556383
N	-0.070626	-0.678131	1.134025	N	-0.075489	-0.624311	1.132229
N	0.914073	1.594312	-0.017440	N	0.951614	1.555565	-0.056069
C	-3.309336	-0.167609	0.375689	C	-3.301577	-0.022242	0.279679
O	-4.429477	-0.255121	0.685755	O	-4.431699	-0.044900	0.546789
C	2.510006	-0.000255	-1.730069	C	2.397254	-0.052777	-1.776763
O	3.310033	0.320743	-2.511069	O	3.146624	0.268793	-2.600479
Cr	-1.531753	0.009927	-0.142915	Cr	-1.517431	0.026935	-0.153687
Cr	1.226157	-0.489943	-0.444622	Cr	1.196854	-0.519461	-0.433126
C	-0.365587	1.778965	-0.622889	C	-0.357403	1.766916	-0.594185
O	-0.616513	2.700189	-1.364237	O	-0.613673	2.714279	-1.302197

Table S34. Optimized coordinates for the $(Me_2C=N)_2Cr_2(CO)_7$ structure **9S-3.**

	M06-L				B3PW91-D3		
	x	y	z		x	y	z
C	3.319671	-0.764365	-0.705059	C	3.322646	0.771894	0.626245
C	1.177548	-1.961769	-1.459927	C	1.239159	1.961380	1.424265
C	-1.898683	0.745690	1.843481	C	-1.725481	-0.768523	-1.794582
C	-0.783018	1.954792	-0.219040	C	-0.821658	-1.912733	0.375270
O	4.442418	-0.921672	-0.954374	O	4.450351	0.934309	0.821536
O	0.911026	-2.763303	-2.251089	O	1.006304	2.775789	2.206043
O	-1.839891	0.939083	2.987839	O	-1.540566	-0.966835	-2.920789
O	-0.274834	2.997861	-0.340672	O	-0.343831	-2.952866	0.580857
C	0.206065	-3.539183	0.973720	C	0.281887	3.525234	-0.996908
C	-1.976778	-2.584994	1.666907	C	-1.949631	2.621407	-1.619463
H	-2.706866	-3.032138	0.983371	H	-2.684676	2.996270	-0.897247
H	-1.809350	-3.308476	2.470436	H	-1.800916	3.406047	-2.369271
H	-0.353692	-4.395400	0.580253	H	-0.258685	4.408901	-0.633839
H	0.458215	-3.787682	2.010802	H	0.538436	3.718550	-2.046467
C	1.126034	0.565646	-1.786366	C	1.129627	-0.485532	1.828402
C	-3.215631	1.721421	-0.372038	C	-3.199411	-1.695117	0.353572
O	1.085436	1.235489	-2.726938	O	1.061184	-1.087857	2.805347
O	-4.035863	2.527952	-0.569296	O	-4.017221	-2.502434	0.536436
H	1.130510	-3.439949	0.406755	H	1.206548	3.426879	-0.428678
H	-2.416485	-1.682116	2.088706	H	-2.365774	1.741616	-2.111186
C	3.727370	2.725602	0.154617	C	3.694022	-2.772087	-0.172521
H	3.471267	3.658147	-0.360116	H	3.468576	-3.709604	0.351716
H	4.517822	2.961374	0.874638	H	4.436031	-2.998283	-0.948620
H	4.108182	2.012236	-0.578332	H	4.117289	-2.056910	0.537445
C	1.896657	3.067441	1.928595	C	1.726496	-3.112720	-1.813614
H	1.570607	4.014750	1.486666	H	1.372469	-4.024516	-1.317209
H	1.036661	2.580583	2.390983	H	0.870722	-2.593647	-2.251756
H	2.641550	3.296849	2.697985	H	2.426536	-3.404421	-2.606611
C	2.507293	2.193851	0.861277	C	2.429000	-2.232979	-0.801640
C	-0.679113	-2.315009	0.940737	C	-0.639135	2.320119	-0.916903
N	1.977784	1.086743	0.572170	N	1.938893	-1.116613	-0.493123
N	-0.403241	-1.191652	0.378529	N	-0.376595	1.198237	-0.347072
O	-1.721574	-0.345416	-3.019986	O	-1.799389	0.563560	2.938983
C	-1.776377	-0.037464	-1.901880	C	-1.845684	0.167491	1.852888
C	2.126809	-1.347145	1.428523	C	2.053696	1.201589	-1.481877
O	2.559057	-1.764522	2.416217	O	2.410357	1.528785	-2.525683
Cr	1.522126	-0.519786	-0.211598	Cr	1.525365	0.501560	0.217615
Cr	-1.915357	0.435609	-0.045245	Cr	-1.909723	-0.417266	0.052198
C	-3.463298	-0.693143	0.015589	C	-3.469247	0.629887	-0.152061
O	-4.500772	-1.219448	-0.011405	O	-4.526660	1.104901	-0.210085

Table S35. Optimized coordinates for the $(Me_2C{=}N)_2Cr_2(CO)_7$ structure **9S-4.**

	M06-L				B3PW91-D3		
	x	y	z		x	y	z
C	2.126297	-0.951489	1.321536	C	2.063972	-1.038406	1.309296
C	0.458174	-1.936535	-0.826881	C	0.333243	-1.918323	-0.855634
C	-2.855215	-0.618031	0.655827	C	-2.849696	-0.487770	0.656859
C	1.264957	0.949938	-1.882870	C	1.350379	0.929137	-1.837412
O	2.586352	-1.584533	2.182021	O	2.508767	-1.691769	2.155524
O	0.903935	-2.858877	-1.390357	O	0.745807	-2.835346	-1.446239
O	-3.951640	-0.435719	0.997956	O	-3.924708	-0.251458	1.017233
O	1.226613	1.445972	-2.929808	O	1.395896	1.445903	-2.869032
C	0.621167	1.083901	2.715857	C	0.621749	1.022763	2.719073
C	0.717897	3.080084	1.176475	C	0.889141	2.989957	1.169394
H	-0.085841	3.688079	1.613727	H	0.104972	3.618454	1.612847
H	1.635478	3.322098	1.717857	H	1.812541	3.179683	1.723670
H	0.020592	1.699697	3.401881	H	0.017394	1.691332	3.351854
H	1.663378	1.185311	3.031444	H	1.652127	1.074275	3.083286
C	2.917615	-0.850669	-1.097144	C	2.843802	-0.961603	-1.081604
C	-1.845256	-2.675208	-0.371084	C	-1.966436	-2.566447	-0.380580
O	3.771672	-1.400138	-1.663231	O	3.672306	-1.534410	-1.651780
O	-2.296894	-3.715058	-0.628767	O	-2.473360	-3.576765	-0.635127
H	0.309536	0.048339	2.832206	H	0.246499	0.009879	2.844943
H	0.829061	3.367721	0.129509	H	1.026712	3.290716	0.128920
C	-2.551867	3.314494	-1.385427	C	-2.363616	3.453154	-1.360136
H	-1.945358	3.224406	-2.287664	H	-1.785181	3.322159	-2.277828
H	-3.565637	2.958422	-1.602407	H	-3.411310	3.186599	-1.553176
H	-2.641174	4.370061	-1.106947	H	-2.348383	4.509622	-1.062324
C	-2.638413	2.590177	1.064924	C	-2.481829	2.691540	1.081468
H	-3.710967	2.405392	0.953319	H	-3.558175	2.519269	0.968982
H	-2.236363	1.880075	1.790904	H	-2.089319	1.980636	1.811000
H	-2.539724	3.604120	1.470785	H	-2.360185	3.710734	1.470476
C	-1.958687	2.518636	-0.268127	C	-1.811548	2.592065	-0.261641
C	0.405289	1.620911	1.336532	C	0.514666	1.532599	1.306477
N	-0.384621	0.983661	0.458899	N	-0.318869	0.952036	0.416254
N	-0.912008	1.829114	-0.571369	N	-0.820829	1.839636	-0.584458
O	3.713463	2.076669	0.490096	O	3.804738	1.855044	0.519283
C	2.880879	1.304506	0.210467	C	2.925098	1.145923	0.236819
C	-0.719593	-1.773243	1.759581	C	-0.813921	-1.750410	1.717036
O	-0.583253	-2.340525	2.764397	O	-0.728546	-2.339437	2.709922
Cr	1.574981	0.065664	-0.190710	Cr	1.554075	0.010670	-0.173138
Cr	-1.107874	-1.013543	0.048726	Cr	-1.148250	-0.959725	0.032020
C	-1.622730	-0.431376	-1.732026	C	-1.670901	-0.365188	-1.714907
O	-2.004026	-0.194118	-2.799249	O	-2.072678	-0.130722	-2.770466

Table S36. Optimized coordinates for the $(Me_2C{=}N)_2Cr_2(CO)_7$ structure **9S-5.**

	M06-L				B3PW91-D3		
	x	y	z		x	y	z
C	2.693118	1.645299	-0.809110	C	2.835780	0.565307	-1.734266
C	2.482811	-0.227602	1.094027	C	2.771619	0.570828	0.917507
C	-3.559827	-0.815262	0.635040	C	-3.815807	-0.244624	-0.277795
C	-2.710396	0.185141	-1.639201	C	-1.927791	0.422424	-1.902875
O	2.530656	2.792436	-0.842548	O	2.689475	1.380328	-2.539363
O	2.117786	-0.217682	2.203146	O	2.633498	1.384931	1.734056
O	-4.579871	-0.749034	1.208206	O	-4.928948	0.094711	-0.176059
O	-3.239917	0.771948	-2.496800	O	-1.917580	1.082836	-2.859402
C	-1.277961	0.189694	3.084994	C	-2.642854	0.255711	2.958648
C	-0.031908	2.319711	2.432350	C	-0.766915	1.996822	3.015897
H	-0.687240	3.190766	2.555228	H	-1.148199	3.013092	2.845336
H	0.457200	2.138163	3.391968	H	-0.844861	1.786124	4.086718
H	-1.607966	0.763627	3.956440	H	-3.139909	0.931563	3.664537
H	-0.443393	-0.439849	3.416377	H	-2.196311	-0.557376	3.547184
C	4.816067	0.101734	-0.141611	C	4.911518	-0.369628	-0.281277
C	-1.303939	-2.162042	1.023825	C	-2.338839	-2.077620	0.887213
O	5.890341	0.313787	0.237168	O	6.033336	-0.109367	-0.199577
O	-0.985426	-3.013544	1.751766	O	-2.595211	-2.956187	1.602071
H	-2.091909	-0.455585	2.753272	H	-3.389033	-0.169586	2.287018
H	0.715984	2.581220	1.678457	H	0.283194	1.985485	2.706578
C	-0.904737	3.667799	-1.601582	C	0.520994	3.394467	-0.821151
H	0.140852	3.473414	-1.842358	H	1.487151	2.984728	-0.518706
H	-1.510303	3.547074	-2.507528	H	0.429838	3.323892	-1.913138
H	-1.017086	4.710745	-1.286038	H	0.474739	4.458371	-0.557557
C	-2.784897	2.962968	-0.015959	C	-1.981315	3.240116	-0.276318
H	-3.441917	3.328180	-0.809454	H	-2.144658	3.624187	-1.289053
H	-3.210185	2.052548	0.415206	H	-2.764414	2.517532	-0.034881
H	-2.767877	3.732760	0.765189	H	-2.060449	4.092014	0.412708
C	-1.398682	2.739384	-0.541700	C	-0.604327	2.642814	-0.174241
C	-0.822606	1.119597	2.013582	C	-1.572198	1.002222	2.225083
N	-1.117611	0.882786	0.772253	N	-1.360650	0.797367	0.964290
N	-0.598868	1.800676	-0.160864	N	-0.306587	1.538017	0.411800
C	3.343801	-2.141710	-0.577965	C	3.204928	-2.024433	1.029142
O	3.541055	-3.278098	-0.480848	O	3.285037	-2.769582	1.907043
O	-2.898565	-3.290012	-1.828666	O	-2.784503	-2.869450	-2.412173
C	-2.517048	-2.361347	-1.229233	C	-2.490062	-2.046280	-1.640732
Cr	2.996460	-0.248632	-0.666237	Cr	3.038218	-0.721211	-0.350969
Cr	-1.952208	-0.888372	-0.254573	Cr	-2.064095	-0.735762	-0.420419
C	1.172698	-0.594273	-1.055298	C	1.183512	-1.023656	-0.438092
O	0.028714	-0.816518	-1.088923	O	0.031793	-1.137871	-0.466161

Table S37. Optimized coordinates for the $(Me_2C{=}N)_2Cr_2(CO)_7$ structure **9T-6.**

	M06-L				B3PW91-D3		
	x	y	z		x	y	z
C	3.878414	-0.289932	-1.045820	C	3.281581	-0.374524	-1.259013
C	1.940740	-2.231740	-0.599717	C	1.608798	-2.310375	-0.186255
C	-3.128738	1.355288	0.914088	C	-1.845764	1.580706	0.821204
C	-1.280727	1.384981	-1.074102	C	-1.104698	1.036254	-1.588524
O	4.951327	-0.215076	-1.483915	O	4.294801	-0.353603	-1.813023
O	1.820754	-3.376344	-0.756620	O	1.537592	-3.459606	-0.093318
O	-3.621005	2.173359	1.569970	O	-1.855358	2.574880	1.417539
O	-0.769161	2.256117	-1.643637	O	-0.874340	1.744156	-2.478359
C	-0.065629	-2.388818	2.206022	C	1.117875	-1.624965	2.947950
C	0.155919	-0.091272	3.347028	C	-1.141477	-0.616765	3.215873
H	-0.250688	-0.509547	4.274046	H	-1.770522	-1.507403	3.337621
H	1.247061	-0.141615	3.423274	H	-0.717755	-0.388545	4.201574
H	-0.595348	-2.836064	3.054419	H	0.692360	-2.392500	3.606396
H	1.002703	-2.586328	2.352308	H	1.670772	-0.927709	3.590984
C	1.407487	-0.000705	-2.063675	C	0.922377	-0.761949	-2.097160
C	-3.654836	0.265999	-1.506645	C	-3.387036	0.385982	-1.056649
O	1.033485	0.143917	-3.150866	O	0.702635	-1.037398	-3.194209
O	-4.485469	0.417638	-2.305864	O	-4.381969	0.644407	-1.598754
H	-0.399877	-2.873446	1.285702	H	1.819020	-2.108863	2.268394
H	-0.150574	0.952512	3.262596	H	-1.771245	0.216713	2.906164
C	2.694490	3.668911	0.196447	C	1.000804	3.841289	-0.752268
H	2.261825	4.413952	-0.480524	H	-0.014335	4.210821	-0.557916
H	2.997614	4.197666	1.106858	H	1.699624	4.661031	-0.543289
H	3.575002	3.229983	-0.274819	H	1.075794	3.541783	-1.799527
C	0.421204	3.066846	1.236818	C	1.350562	2.977859	1.655115
H	-0.065975	3.875091	0.679928	H	0.520929	3.634283	1.938825
H	-0.282581	2.236528	1.350087	H	1.296699	2.056887	2.240469
H	0.677267	3.462775	2.226590	H	2.290953	3.497314	1.879563
C	1.667722	2.612083	0.518456	C	1.281578	2.675815	0.170098
C	-0.332948	-0.901272	2.171283	C	-0.014184	-0.900153	2.239481
N	1.840241	1.406277	0.193574	N	1.430022	1.508174	-0.271616
N	-0.979582	-0.374954	1.225989	N	-0.021498	-0.574556	1.001033
O	-1.085396	-2.183330	-2.009483	O	-1.615164	-2.714951	-1.603850
C	-1.520872	-1.353484	-1.329042	C	-1.658438	-1.683856	-1.084829
C	2.930678	-0.713269	1.386953	C	2.809673	-0.058435	1.145655
O	3.450663	-0.987010	2.392394	O	3.597246	0.263843	1.924809
Cr	2.152733	-0.331860	-0.309265	Cr	1.629678	-0.418051	-0.321971
Cr	-2.309878	0.003790	-0.200021	Cr	-1.808491	-0.013111	-0.180823
C	-3.423347	-1.323611	0.638366	C	-3.007585	-0.913260	0.980454
O	-4.096062	-2.120162	1.143858	O	-3.861049	-1.437102	1.562299

Table S38. Optimized coordinates for the $(Me_2C{=}N)_2Cr_2(CO)_7$ structure **9T-7.**

	M06-L				B3PW91-D3		
	x	y	z		x	y	z
C	-0.092799	-1.304514	0.294888	C	-0.436892	0.139660	-1.257703
C	2.294534	-2.499502	0.548991	C	-3.869161	-0.202426	0.320422
C	-3.706102	-0.595580	1.675584	C	3.465849	-0.557750	-1.764733
C	-1.979676	0.370003	-1.616607	C	1.489619	0.510424	1.294101
O	-1.211608	-1.653837	0.460953	O	0.647620	0.225928	-1.694617
O	2.678268	-3.554387	0.848622	O	-4.901544	-0.357322	0.804662
O	-4.278011	-0.854382	2.650041	O	4.151885	-0.882807	-2.634563
O	-1.509758	0.672487	-2.635704	O	1.026958	0.855712	2.298385
C	5.576262	0.155403	-1.366624	C	-1.376657	-3.624499	0.991712
C	5.330974	0.967920	1.064308	C	-0.496459	-2.113916	2.881717
H	6.236234	0.435827	1.374680	H	-1.074555	-2.700566	3.606768
H	5.630170	1.988655	0.802451	H	0.516145	-2.536156	2.846628
H	6.477319	-0.421234	-1.132735	H	-2.086295	-4.139673	1.651995
H	5.904181	1.144666	-1.702891	H	-0.436975	-4.189124	1.002996
C	1.612808	-0.287628	1.955174	C	-2.848385	1.176617	-1.826271
C	-4.170900	1.117276	-0.306867	C	3.911539	-0.231692	0.794136
O	1.540296	-0.006752	3.076402	O	-3.274544	1.886448	-2.635238
O	-5.016909	1.892098	-0.524603	O	4.851952	-0.362688	1.465669
H	5.036460	-0.337625	-2.175964	H	-1.776111	-3.610480	-0.024664
H	4.632828	1.003859	1.902660	H	-0.440807	-1.073800	3.208860
C	1.626432	3.188293	0.771218	C	-1.837731	3.183361	2.394732
H	0.842960	3.875473	1.105872	H	-2.137670	4.231273	2.270040
H	1.926759	2.556307	1.609329	H	-2.695298	2.593140	2.727510
H	2.482959	3.793586	0.452861	H	-1.054703	3.147248	3.162917
C	0.528197	3.115914	-1.550363	C	-0.131656	3.409183	0.465784
H	1.171998	3.955128	-1.832473	H	0.698266	3.491043	1.177700
H	0.371007	2.465990	-2.410827	H	0.219069	2.911846	-0.441509
H	-0.438241	3.535640	-1.246197	H	-0.460811	4.425240	0.215219
C	1.129167	2.359771	-0.391360	C	-1.276986	2.641730	1.096883
C	4.711575	0.286173	-0.134676	C	-1.131980	-2.218883	1.508321
N	1.203375	1.104577	-0.379518	N	-1.721065	1.591481	0.575789
N	3.534421	-0.150099	-0.106657	N	-1.429499	-1.193668	0.846260
C	-1.869026	1.259434	0.976072	C	2.904401	1.781320	-0.573819
O	-1.341306	2.126357	1.545321	O	3.236394	2.878640	-0.725320
C	1.557275	-1.308566	-1.805675	C	-2.494011	-1.459818	-1.639842
O	1.455118	-1.654988	-2.902876	O	-2.694307	-2.363839	-2.335505
Cr	-2.828257	-0.101316	0.035751	Cr	2.424265	-0.028082	-0.258173
Cr	1.655983	-0.769833	0.079769	Cr	-2.126027	0.015930	-0.518675
C	-3.846209	-1.450237	-0.888341	C	1.976546	-1.840631	0.066224
O	-4.506051	-2.218471	-1.451949	O	1.772807	-2.954959	0.303474

Table S39. Optimized coordinates for the $(Me_2C{=}N)_2Cr_2(CO)_7$ structure **9T-8.**

	M06-L				B3PW91-D3		
	x	y	z		x	y	z
C	2.126296	-1.917073	0.843697	C	1.548728	-2.350698	0.213014
C	-1.195824	-0.560271	-1.848659	C	-1.281611	-1.058936	-1.719425
C	-2.296609	-0.391348	1.829880	C	-2.553886	0.296194	1.589902
C	2.444928	0.737100	-1.722711	C	3.127075	0.731728	-0.866967
O	2.442346	-2.830345	1.487778	O	1.548811	-3.486199	0.424691
O	-1.022957	-0.547655	-2.994702	O	-1.093105	-1.497548	-2.771989
O	-2.724523	-0.273892	2.904266	O	-3.052223	0.689089	2.559496
O	2.903211	1.367690	-2.578092	O	3.981937	1.399719	-1.266932
C	0.711909	0.046826	2.712373	C	0.495931	-0.154492	2.718675
C	1.643281	2.223513	1.810014	C	1.741028	1.910999	1.977392
H	1.023822	2.885465	2.431919	H	1.132551	2.581025	2.602689
H	2.548567	2.008392	2.382999	H	2.577999	1.572823	2.595942
H	0.089366	0.558836	3.460896	H	-0.177609	0.411346	3.379415
H	1.670031	-0.182353	3.188541	H	1.370762	-0.439318	3.311929
C	1.596092	-1.750302	-1.617901	C	1.953027	-1.156316	-1.998655
C	-3.191927	-1.569835	-0.378576	C	-3.512021	-1.077187	-0.391472
O	1.548835	-2.613107	-2.393061	O	2.158328	-1.577970	-3.055523
O	-4.134096	-2.209605	-0.624938	O	-4.565239	-1.515448	-0.612836
H	0.214455	-0.888646	2.445478	H	-0.022753	-1.058277	2.400445
H	1.925634	2.763328	0.904305	H	2.137956	2.484391	1.138338
C	-0.468766	3.926019	-1.392514	C	0.280407	3.869610	-1.480762
H	0.173186	3.594670	-2.209629	H	0.944682	3.415540	-2.219634
H	-1.482420	4.087169	-1.776920	H	-0.644353	4.190232	-1.977859
H	-0.115841	4.893198	-1.018837	H	0.752594	4.769743	-1.066414
C	-1.263384	3.268388	0.946111	C	-0.896389	3.402707	0.743726
H	-2.245340	3.669434	0.679593	H	-1.802516	3.868176	0.338838
H	-1.399458	2.414740	1.616114	H	-1.182725	2.618958	1.446343
H	-0.735654	4.062004	1.488233	H	-0.351277	4.187328	1.284607
C	-0.499331	2.915801	-0.293095	C	-0.043763	2.893953	-0.385145
C	0.899866	0.950192	1.530588	C	0.907462	0.719142	1.561031
N	0.066918	0.791509	0.475941	N	0.144153	0.696581	0.431354
N	0.122087	1.807652	-0.516275	N	0.423695	1.704201	-0.522225
O	4.341122	0.459327	1.165842	O	3.906799	-0.814992	1.951789
C	3.368908	0.141101	0.600569	C	3.101995	-0.698140	1.117763
C	-0.884212	-2.262589	0.390555	C	-1.535035	-2.050955	0.800249
O	-0.580883	-3.367328	0.581715	O	-1.518869	-3.106162	1.276770
Cr	1.770735	-0.383966	-0.257932	Cr	1.804693	-0.490828	-0.202360
Cr	-1.691880	-0.547288	0.024338	Cr	-1.847874	-0.373077	-0.032152
C	-2.788407	0.942720	-0.427508	C	-2.476798	1.146324	-0.962943
O	-3.535404	1.775946	-0.748939	O	-2.967791	2.007346	-1.564036

Table S40. Optimized coordinates for the $(Me_2C{=}N)_2Cr_2(CO)_7$ structure **9T-9.**

	M06-L				B3PW91-D3		
	x	y	z		x	y	z
C	2.639291	0.240236	-1.663870	C	3.183471	0.955386	-1.524982
C	3.007255	1.564971	0.688206	C	3.823399	1.583206	1.022185
C	-3.710007	-0.620972	-0.431574	C	-3.579042	-1.357544	-0.428323
C	-1.527469	-0.361349	-1.939381	C	-1.846316	-0.256536	-2.009062
O	2.339565	0.536829	-2.748047	O	3.112397	1.552529	-2.513754
O	2.938475	2.672135	1.032493	O	4.136672	2.546494	1.576330
O	-4.840659	-0.376225	-0.598153	O	-4.722301	-1.565513	-0.532872
O	-1.328696	-0.046200	-3.041440	O	-1.920886	0.101364	-3.111293
C	-2.999309	1.053237	2.563492	C	-3.410523	0.276449	2.627732
C	-1.275476	2.906246	2.206403	C	-2.328621	2.586023	2.495209
H	-1.671152	3.761756	1.645860	H	-2.997009	3.334868	2.048994
H	-1.488441	3.078051	3.263373	H	-2.544322	2.546624	3.567247
H	-3.659501	1.855328	2.907449	H	-4.309826	0.797852	2.979268
H	-2.593971	0.572382	3.461817	H	-2.879806	-0.080729	3.521009
C	4.500487	-1.400848	-0.534378	C	4.531545	-1.244581	-0.710227
C	-2.298335	-1.796026	1.529449	C	-1.714257	-2.065358	1.306523
O	5.359885	-2.069293	-0.930319	O	5.275728	-1.979698	-1.196273
O	-2.592839	-2.377642	2.492164	O	-1.700218	-2.806135	2.199484
H	-3.589633	0.319289	2.014223	H	-3.708383	-0.582171	2.026227
H	-0.194614	2.892028	2.038149	H	-1.300888	2.927502	2.333054
C	0.352796	3.076782	-1.759357	C	-0.779177	3.652279	-1.378996
H	1.317015	2.873997	-1.290378	H	0.210650	3.616007	-0.917330
H	0.369708	2.688657	-2.784342	H	-0.683394	3.424525	-2.448941
H	0.204219	4.159503	-1.831718	H	-1.182316	4.670443	-1.302615
C	-2.162327	2.772028	-1.431124	C	-3.147324	2.689145	-1.165068
H	-2.212417	2.855907	-2.519933	H	-3.205118	2.749693	-2.257614
H	-2.884922	2.025420	-1.091697	H	-3.699595	1.810457	-0.824488
H	-2.461962	3.745373	-1.024145	H	-3.636691	3.589811	-0.769343
C	-0.764610	2.446388	-0.997197	C	-1.707041	2.666653	-0.727460
C	-1.900615	1.629360	1.737978	C	-2.547717	1.235262	1.868427
N	-1.513581	1.037813	0.649686	N	-2.020092	0.908100	0.731096
N	-0.446498	1.663646	-0.020872	N	-1.194441	1.881197	0.153782
C	3.419519	-0.829787	1.926976	C	3.372653	-0.990918	1.727392
O	3.622784	-1.161594	3.020972	O	3.420373	-1.581941	2.720378
O	-2.302964	-3.742303	-1.362246	O	-1.218478	-3.620581	-1.708164
C	-2.130623	-2.686344	-0.900188	C	-1.422793	-2.615923	-1.155711
Cr	3.046631	-0.280490	0.125619	Cr	3.281494	-0.017356	0.099341
Cr	-1.909221	-0.979809	-0.165558	Cr	-1.780680	-1.029483	-0.283019
C	1.283731	-0.914412	0.204788	C	1.420624	-0.382031	-0.022109
O	0.139761	-1.181680	0.205433	O	0.282413	-0.595881	-0.095651

Table S41. Optimized coordinates for the $(Me_2C{=}N)_2Cr_2(CO)_7$ structure **9S-10.**

	M06-L				B3PW91-D3		
	x	y	z		x	y	z
C	0.051056	-1.124613	-0.232017	C	3.010007	-0.743702	-0.957293
C	3.233547	-1.662165	0.952934	C	3.550360	0.695001	1.066606
C	-3.429285	-1.093413	1.502880	C	0.230682	-2.492054	-1.051588
C	-2.628292	0.900791	-1.684524	C	-2.096083	-1.526610	-1.674058
O	-1.111110	-1.219177	-0.328758	O	3.700613	-1.244177	-1.742823
O	4.102974	-2.152061	1.544036	O	4.545009	1.159376	1.447180
O	-3.723597	-1.655686	2.472725	O	0.675618	-3.530240	-1.262821
O	-2.430546	1.543084	-2.630653	O	-2.943666	-1.905254	-2.365024
C	5.523586	0.945483	-1.765983	C	-4.799901	1.941776	1.744543
C	4.940877	1.084410	0.714935	C	-4.734504	1.196634	-0.715311
H	5.853594	0.596446	1.076739	H	-4.362404	0.404019	-1.369384
H	5.151599	2.159530	0.680347	H	-5.829611	1.201845	-0.752110
H	6.538888	0.603887	-1.532815	H	-5.893895	1.887616	1.783271
H	5.574092	2.035475	-1.871330	H	-4.515524	2.967691	1.484842
C	1.219270	-0.548206	1.695007	C	2.157152	-1.649163	1.237522
C	-4.596896	0.839862	0.097827	C	0.708799	0.543115	-1.059488
O	0.795098	-0.377262	2.763695	O	2.322816	-2.674654	1.758036
O	-5.590241	1.438205	0.220784	O	0.118908	0.288732	-2.143582
H	5.207340	0.509881	-2.714010	H	-4.393045	1.690061	2.727119
H	4.135883	0.903010	1.433427	H	-4.377728	2.165927	-1.081493
C	1.615863	2.989991	1.231160	C	2.244862	3.903487	-0.448895
H	0.750551	3.513291	1.650944	H	2.958318	3.428408	0.227801
H	2.002315	2.288336	1.973577	H	2.789953	4.431114	-1.242744
H	2.382316	3.745827	1.024249	H	1.677895	4.665187	0.104002
C	0.587200	3.136557	-1.112652	C	0.319204	3.381216	-2.066388
H	1.229246	3.989669	-1.356968	H	-0.205326	4.266950	-1.685526
H	0.395673	2.558546	-2.017058	H	0.857415	3.696636	-2.970968
H	-0.360957	3.544518	-0.741868	H	-0.398966	2.607699	-2.343575
C	1.228875	2.282890	-0.045381	C	1.307867	2.897014	-1.044155
C	4.569741	0.567912	-0.656909	C	-4.287123	0.973735	0.708213
N	1.421324	1.051024	-0.233707	N	1.372096	1.673025	-0.649460
N	3.544611	-0.133372	-0.911715	N	-2.962728	0.467308	0.948848
C	-2.123669	1.223488	0.946763	O	0.586430	0.934002	2.993451
O	-1.623416	2.063174	1.576423	C	1.066555	0.596013	1.997859
C	2.050017	-2.401558	-1.084916	C	-0.964730	-1.733814	0.995726
O	2.106572	-3.366825	-1.713906	O	-1.137439	-2.264006	2.010276
Cr	-3.019881	-0.104586	-0.097360	Cr	-0.738182	-0.837358	-0.611005
Cr	1.901585	-0.732649	-0.029792	Cr	1.958322	0.006231	0.410302
C	-3.961975	-1.414662	-1.151102	C	-2.001018	0.102002	0.143010
O	-4.583542	-2.165822	-1.777317	O	-4.209389	-0.383322	1.128069

Table S42. Optimized coordinates for the $(Me_2C=N)_2Cr_2(CO)_7$ structure **9T-11.**

	M06-L				B3PW91-D3		
	x	y	z		x	y	z
C	3.165240	0.052943	1.485480	C	3.235097	0.124748	1.300036
C	1.886502	-1.696119	0.077193	C	2.196089	-1.280992	-0.568906
C	-3.193566	1.476408	0.252706	C	-3.340308	1.225604	0.720584
C	-1.573842	1.435801	-1.927346	C	-2.021315	1.776489	-1.520230
O	3.797727	-0.047237	2.461860	O	3.965995	-0.166516	2.157302
O	1.628280	-2.818172	0.290163	O	2.186939	-2.432828	-0.752794
O	-3.881062	2.274592	0.760422	O	-4.060128	1.838712	1.402869
O	-1.272582	2.220047	-2.733414	O	-1.971049	2.720761	-2.190921
C	-1.954300	-0.504208	2.804414	C	-1.715955	-1.798738	2.206161
C	0.426222	0.295250	3.235634	C	0.560224	-0.920709	2.953371
H	0.046642	0.430683	4.250901	H	0.229546	-1.314549	3.919459
H	1.045024	-0.611175	3.239740	H	1.286155	-1.633237	2.539646
H	-2.444343	0.207445	3.480096	H	-2.105750	-1.740858	3.229949
H	-1.725434	-1.400283	3.388357	H	-1.322756	-2.813371	2.057511
C	3.927061	-0.344951	-0.896079	C	3.832041	0.689008	-1.069754
C	-1.322916	-3.238591	1.078573	C	-0.007220	-4.776307	0.486549
O	4.975531	-0.609607	-1.339509	O	4.862087	0.810772	-1.600576
O	-1.459058	-4.231343	0.527855	O	0.353371	-5.640710	-0.164509
H	-2.650020	-0.766534	2.004364	H	-2.532535	-1.627625	1.498712
H	1.077740	1.130924	2.973900	H	1.077280	0.028503	3.105600
C	1.807839	2.841115	-0.230262	C	1.525081	2.968263	0.850464
H	2.670925	2.144620	-0.181895	H	2.153376	2.551705	0.033035
H	1.598366	3.052859	-1.285290	H	1.180291	3.936843	0.467487
H	2.165636	3.765983	0.229229	H	2.170103	3.161985	1.715676
C	-0.484380	3.285168	0.856672	C	-0.736059	2.749563	2.048739
H	-0.999003	3.681974	-0.027783	H	-1.347805	3.399997	1.409754
H	-1.234814	2.826181	1.501564	H	-1.393723	2.016444	2.517656
H	-0.037648	4.140713	1.372052	H	-0.289110	3.386688	2.821702
C	0.575142	2.326065	0.444294	C	0.348480	2.117434	1.238296
C	-0.702415	0.106725	2.279299	C	-0.607691	-0.812223	2.020306
N	-0.664427	0.445902	1.026971	N	-0.703876	0.046281	1.054950
N	0.530305	1.035960	0.556773	N	0.402431	0.893454	0.832135
C	1.664102	-0.013256	-2.009069	C	1.472733	0.816015	-1.952960
O	1.439376	-0.117028	-3.144737	O	1.168787	0.957776	-3.061643
O	-0.614263	-2.175423	-1.934151	O	-0.831171	-1.711320	-2.352836
C	-1.096358	-1.273883	-1.381078	C	-1.313503	-0.963105	-1.613767
Cr	2.267056	0.072209	-0.184034	Cr	2.206575	0.505998	-0.227320
Cr	-2.089784	0.167267	-0.562884	Cr	-2.261493	0.216159	-0.438353
C	-3.638393	-0.985107	-0.634682	C	-3.839970	-0.805882	-0.760589
O	-4.581993	-1.658829	-0.728477	O	-4.825238	-1.366328	-1.009323

Table S43. Optimized coordinates for the $(Me_2C{=}N)_2Cr_2(CO)_7$ structure **9S-12.**

	M06-L				B3PW91-D3		
	x	y	z		x	y	z
C	-4.852976	0.312762	-0.807708	C	-5.380076	0.254636	0.106476
C	-3.779939	-2.143976	-0.251990	C	-3.470575	0.002789	1.986587
C	3.791178	-0.903238	0.111374	C	3.399688	-1.558161	0.056030
C	1.976346	-0.700962	1.890431	C	1.681322	-1.178270	1.936506
O	-5.922787	0.733831	-0.951751	O	-6.493286	0.561634	0.110762
O	-4.206414	-3.200270	-0.049599	O	-3.427680	0.158896	3.129658
O	4.956773	-0.790726	0.169723	O	4.558507	-1.714699	0.058744
O	2.104935	-0.483126	3.029603	O	1.772415	-1.252513	3.094240
C	3.600426	2.223950	-0.764172	C	3.380675	1.236740	-2.234752
C	1.604302	2.726791	-2.298494	C	1.251778	2.662375	-2.001900
H	1.872675	3.787706	-2.343840	H	1.762065	3.544405	-2.408217
H	2.047993	2.237245	-3.170803	H	0.743223	2.160001	-2.833550
H	3.911462	3.272879	-0.813258	H	3.941441	2.091931	-2.631779
H	4.173364	1.670493	-1.514590	H	2.987595	0.672927	-3.088236
C	-3.243017	-0.054223	1.309809	C	-3.101096	1.595692	-0.098410
C	2.162264	-2.910936	0.471535	C	1.270722	-3.070485	0.302147
O	-3.336306	0.146443	2.454421	O	-2.822684	2.717464	-0.219854
O	2.307691	-4.036109	0.756337	O	1.082440	-4.212567	0.454475
H	3.853353	1.817761	0.217949	H	4.059345	0.586459	-1.676227
H	0.518708	2.627965	-2.361868	H	0.496681	2.980892	-1.277222
C	-0.187866	1.817710	2.000183	C	2.931824	1.981837	2.410809
H	-0.322200	0.796798	1.629444	H	1.976019	1.475465	2.568437
H	0.245056	1.752597	3.004383	H	3.731631	1.270479	2.657908
H	-1.170736	2.292391	2.101302	H	3.008040	2.823973	3.106955
C	0.833303	4.086176	1.256225	C	3.882685	3.689432	0.688774
H	1.197517	4.327718	2.260604	H	4.913533	3.558642	1.044624
H	1.528683	4.506084	0.525729	H	3.910941	3.895170	-0.385054
H	-0.135543	4.587723	1.139967	H	3.473591	4.568912	1.204136
C	0.678147	2.613845	1.087159	C	3.066984	2.470918	1.001884
C	2.129049	2.073629	-1.046991	C	2.227178	1.693939	-1.368343
N	1.428456	0.874690	-0.568401	N	1.705871	0.805733	-0.317254
N	1.263699	2.030783	0.091433	N	2.485862	1.837057	0.038022
O	-1.942392	2.478825	-0.780313	O	-3.617944	-0.442776	-2.954148
C	-2.375196	1.401235	-0.718665	C	-3.589527	-0.372159	-1.802018
C	2.232979	-1.576404	-1.815966	C	1.582280	-1.661767	-1.798244
O	2.495352	-1.887382	-2.906517	O	1.627617	-2.005189	-2.907801
Cr	-3.075561	-0.361473	-0.495274	Cr	-3.523569	-0.174083	0.090696
Cr	1.970435	-1.118535	0.030580	Cr	1.601049	-1.276250	0.054597
C	-1.287652	-0.973874	-0.278640	C	-1.681695	-0.614424	0.076239
O	-0.173158	-1.267786	-0.102918	O	-0.544232	-0.825168	0.060814

Table S44. Optimized coordinates for the $(Me_2C{=}N)_2Cr_2(CO)_7$ structure **9T-13.**

	M06-L				B3PW91-D3		
	x	y	z		x	y	z
C	-3.167363	1.599940	-1.202031	C	-3.528811	2.106618	-0.632400
C	-4.206387	-0.851844	-0.823986	C	-3.408533	-0.228911	-1.966014
C	3.115020	-1.242267	0.575052	C	3.225877	-1.932594	0.324506
C	0.660417	-1.278598	1.625092	C	1.590282	-0.838913	2.022859
O	-3.315506	2.449505	-1.985885	O	-3.607312	3.223044	-0.929845
O	-4.986060	-1.501971	-1.388747	O	-3.412291	-0.533897	-3.083504
O	4.179748	-1.114779	1.035590	O	4.306923	-2.352633	0.421173
O	0.259178	-1.291137	2.713458	O	1.712248	-0.660168	3.162537
C	2.815688	1.776178	-2.113150	C	4.537387	0.937881	-1.079202
C	0.352288	2.496817	-1.894116	C	2.682641	1.937932	-2.557572
H	0.607519	3.501171	-2.246782	H	3.307714	2.801350	-2.815643
H	-0.014783	1.923920	-2.751675	H	2.813608	1.184428	-3.343203
H	3.166004	2.790342	-2.330267	H	5.204844	1.772125	-1.326394
H	2.630092	1.282345	-3.071075	H	4.757464	0.117610	-1.772202
C	-3.295805	-0.670641	1.748848	C	-4.655499	-1.094934	0.270502
C	1.484137	-3.254777	-0.057895	C	0.865912	-2.974788	0.702746
O	-3.516476	-1.196054	2.761531	O	-5.425338	-1.920051	0.519430
O	1.580132	-4.410690	0.000210	O	0.491367	-4.023573	1.031725
H	3.603742	1.228913	-1.588123	H	4.748673	0.593953	-0.062909
H	-0.455573	2.561369	-1.161666	H	1.631249	2.239821	-2.541853
C	2.104088	1.730514	2.498300	C	1.162138	2.554831	1.887441
H	2.237999	0.655694	2.355735	H	0.472852	1.745029	1.632730
H	2.912005	2.110057	3.128770	H	1.633926	2.311444	2.848732
H	1.158973	1.879521	3.033021	H	0.588828	3.478903	2.027370
C	2.438599	3.894124	1.100716	C	2.952917	4.038911	0.715110
H	3.461768	4.067913	1.450990	H	3.489938	4.237226	1.652311
H	2.348403	4.268827	0.078750	H	3.679385	4.017274	-0.102293
H	1.777862	4.485805	1.744567	H	2.267447	4.881768	0.553431
C	2.085114	2.450859	1.197912	C	2.187659	2.753840	0.815184
C	1.545327	1.796462	-1.304587	C	3.084704	1.334386	-1.229106
N	1.274805	0.681755	-0.393909	N	2.061400	0.601331	-0.481879
N	1.701225	1.836317	0.123173	N	2.412516	1.815840	-0.046558
O	-0.936043	2.009330	1.615547	O	-3.243768	1.073169	2.800021
C	-1.688599	1.325679	1.033992	C	-3.307218	0.766048	1.683614
C	2.101300	-1.536921	-1.944350	C	1.490385	-1.879750	-1.627232
O	2.566080	-1.702844	-2.994788	O	1.539121	-2.298227	-2.705245
Cr	-2.873996	0.200218	0.079626	Cr	-3.362526	0.266714	-0.138308
Cr	1.403227	-1.358267	-0.157194	Cr	1.498135	-1.294673	0.186329
C	-1.509284	-0.782299	-0.586087	C	-1.596392	-0.286879	-0.032681
O	-0.577929	-1.404934	-1.007881	O	-0.469530	-0.606690	0.034951

Table S45. Optimized coordinates for the $(Me_2C{=}N)_2Cr_2(CO)_7$ structure **9S-14.**

	M06-L				B3PW91-D3		
	x	y	z		x	y	z
C	3.083689	-0.812215	-0.654982	C	0.263890	-0.423167	0.045512
C	3.299489	0.841000	1.313350	C	3.490268	-1.964265	0.467539
C	0.532192	-2.515255	-0.931392	C	-3.257073	-1.188029	1.337476
C	-2.084082	-1.671088	-1.770961	C	-2.899888	1.122024	-1.663658
O	3.872060	-1.382724	-1.292981	O	-0.882021	-0.232590	-0.041860
O	4.219387	1.379265	1.784860	O	4.367963	-2.698313	0.615186
O	1.150275	-3.482310	-1.005846	O	-3.443146	-1.883241	2.241456
O	-2.969544	-2.093890	-2.393853	O	-2.868262	1.827591	-2.579625
C	-4.753711	2.310177	0.805739	C	4.541875	1.580752	-2.274979
C	-4.687781	-0.160680	0.134909	C	5.745237	1.073213	-0.056590
H	-4.072900	-1.060042	0.218360	H	6.613986	0.636690	-0.567161
H	-5.633539	-0.332864	0.654855	H	5.962590	2.138134	0.098646
H	-5.644584	2.176580	1.425560	H	5.382986	1.188510	-2.861966
H	-5.080098	2.609426	-0.193807	H	4.700377	2.661593	-2.163974
C	1.885816	-1.563336	1.469882	C	1.469000	-1.542051	1.810994
C	0.795287	0.414103	-1.211573	C	-4.880342	0.124746	-0.258962
O	1.928559	-2.559965	2.074184	O	1.109976	-2.041379	2.786850
O	0.349174	0.022034	-2.324943	O	-6.036405	0.224292	-0.320725
H	-4.135916	3.097530	1.238250	H	3.611111	1.406726	-2.820803
H	-4.910734	0.023685	-0.919893	H	5.615263	0.589379	0.914592
C	2.074015	3.872991	-0.656780	C	1.434123	3.243583	2.002891
H	2.701895	3.487407	0.147808	H	0.464525	3.676197	2.284069
H	2.703840	4.374653	-1.399659	H	1.888203	2.774165	2.878857
H	1.405928	4.641307	-0.250944	H	2.071738	4.069886	1.661389
C	0.399790	3.125150	-2.450454	C	0.586425	2.759779	-0.366545
H	-0.251908	3.964869	-2.185428	H	1.072327	3.692457	-0.679730
H	1.009310	3.462657	-3.296469	H	0.644801	2.037860	-1.185092
H	-0.203202	2.276389	-2.773415	H	-0.465657	3.002850	-0.169173
C	1.281671	2.783695	-1.294335	C	1.251784	2.226359	0.889192
C	-3.987263	1.019010	0.750657	C	4.499379	0.912755	-0.910666
N	1.361338	1.590693	-0.805639	N	1.656829	1.039904	1.057705
N	-2.560525	1.184364	0.494637	N	3.490147	0.274947	-0.518607
O	-0.010220	1.008644	2.785346	C	-3.010247	1.466776	1.004718
C	0.647359	0.679891	1.889867	O	-3.041847	2.384688	1.708438
C	-1.198602	-1.747899	0.856040	C	1.870268	-1.698596	-1.300906
O	-1.505973	-2.225115	1.868943	O	1.719891	-2.267068	-2.296047
Cr	-0.712523	-0.896180	-0.746964	Cr	-3.049254	-0.031309	-0.161036
Cr	1.826231	0.032023	0.488058	Cr	2.101075	-0.678653	0.237395
C	-1.790091	0.315673	-0.111095	C	-3.140324	-1.533870	-1.327516
O	-3.184897	0.739742	1.865829	O	-3.253780	-2.438903	-2.037042

Table S46. Optimized coordinates for the $(Me_2C{=}N)_2Fe_2(CO)_6$ structure **6S-1.**

	M06-L				B3PW91-D3		
	x	y	z		x	y	z
C	1.320374	1.653364	1.414951	C	1.298489	1.662331	1.428242
C	0.000000	2.696005	-0.756248	C	0.000000	2.645498	-0.753717
C	-1.320374	-1.653364	1.414951	C	-1.298489	-1.662331	1.428242
C	0.000000	-2.696005	-0.756248	C	0.000000	-2.645498	-0.753717
O	2.207507	1.889025	2.124812	O	2.160438	1.933846	2.147648
O	0.000000	3.660539	-1.406255	O	0.000000	3.588157	-1.426319
O	-2.207507	-1.889025	2.124812	O	-2.160438	-1.933846	2.147648
O	0.000000	-3.660539	-1.406255	O	0.000000	-3.588157	-1.426319
C	3.032579	-1.272861	-1.641337	C	2.979861	-1.274943	-1.688077
C	3.032579	1.272861	-1.641337	C	2.979861	1.274943	-1.688077
H	3.981224	1.349308	-1.096901	H	3.992811	1.304311	-1.265583
H	3.280041	1.280315	-2.708193	H	3.083882	1.314670	-2.780197
H	3.981224	-1.349308	-1.096901	H	3.992811	-1.304311	-1.265583
H	3.280041	-1.280315	-2.708193	H	3.083882	-1.314670	-2.780197
C	-1.320374	1.653364	1.414951	C	-1.298489	1.662331	1.428242
C	1.320374	-1.653364	1.414951	C	1.298489	-1.662331	1.428242
O	-2.207507	1.889025	2.124812	O	-2.160438	1.933846	2.147648
O	2.207507	-1.889025	2.124812	O	2.160438	-1.933846	2.147648
H	2.427955	-2.150171	-1.405100	H	2.430287	-2.153412	-1.345583
H	2.427955	2.150171	-1.405100	H	2.430287	2.153412	-1.345583
C	-3.032579	1.272861	-1.641337	C	-2.979861	1.274943	-1.688077
H	-3.981224	1.349308	-1.096901	H	-3.992811	1.304311	-1.265583
H	-2.427955	2.150171	-1.405100	H	-2.430287	2.153412	-1.345583
H	-3.280041	1.280315	-2.708193	H	-3.083882	1.314670	-2.780197
C	-3.032579	-1.272861	-1.641337	C	-2.979861	-1.274943	-1.688077
H	-3.280041	-1.280315	-2.708193	H	-3.083882	-1.314670	-2.780197
H	-2.427955	-2.150171	-1.405100	H	-2.430287	-2.153412	-1.345583
H	-3.981224	-1.349308	-1.096901	H	-3.992811	-1.304311	-1.265583
Fe	0.000000	-1.221041	0.268327	Fe	0.000000	-1.204137	0.282962
Fe	0.000000	1.221041	0.268327	Fe	0.000000	1.204137	0.282962
C	-2.328106	0.000000	-1.264152	C	-2.287670	0.000000	-1.273730
C	2.328106	0.000000	-1.264152	C	2.287670	0.000000	-1.273730
N	-1.219127	0.000000	-0.637721	N	-1.206128	0.000000	-0.606203
N	1.219127	0.000000	-0.637721	N	1.206128	0.000000	-0.606203

Table S47. Optimized coordinates for the $(Me_2C{=}N)_2Fe_2(CO)_6$ structure **6T-2.**

	M06-L				B3PW91-D3		
	x	y	z		x	y	z
C	1.296195	2.253739	1.261370	C	1.282129	2.232209	1.283552
C	0.000000	2.579953	-1.165909	C	0.000000	2.586356	-1.143673
C	-1.296195	-2.253739	1.261370	C	-1.282129	-2.232209	1.283552
C	0.000000	-2.579953	-1.165909	C	0.000000	-2.586356	-1.143673
O	2.146628	2.752532	1.875963	O	2.123125	2.710436	1.917351
O	0.000000	3.265167	-2.102877	O	0.000000	3.231957	-2.102343
O	-2.146628	-2.752532	1.875963	O	-2.123125	-2.710436	1.917351
O	0.000000	-3.265167	-2.102877	O	0.000000	-3.231957	-2.102343
C	3.138677	-1.261685	-1.192394	C	3.098517	-1.262931	-1.251420
C	3.138677	1.261685	-1.192394	C	3.098517	1.262931	-1.251420
H	4.053256	1.335825	-0.592208	H	4.076590	1.291737	-0.753618
H	3.453656	1.242483	-2.241193	H	3.287326	1.271785	-2.332656
H	4.053256	-1.335825	-0.592208	H	4.076590	-1.291737	-0.753618
H	3.453656	-1.242483	-2.241193	H	3.287326	-1.271785	-2.332656
C	-1.296195	2.253739	1.261370	C	-1.282129	2.232209	1.283552
C	1.296195	-2.253739	1.261370	C	1.282129	-2.232209	1.283552
O	-2.146628	2.752532	1.875963	O	-2.123125	2.710436	1.917351
O	2.146628	-2.752532	1.875963	O	2.123125	-2.710436	1.917351
H	2.538165	-2.155373	-1.018934	H	2.539192	-2.159915	-0.983175
H	2.538165	2.155373	-1.018934	H	2.539192	2.159915	-0.983175
C	-3.138677	1.261685	-1.192394	C	-3.098517	1.262931	-1.251420
H	-4.053256	1.335825	-0.592208	H	-4.076590	1.291737	-0.753618
H	-2.538165	2.155373	-1.018934	H	-2.539192	2.159915	-0.983175
H	-3.453656	1.242483	-2.241193	H	-3.287326	1.271785	-2.332656
C	-3.138677	-1.261685	-1.192394	C	-3.098517	-1.262931	-1.251420
H	-3.453656	-1.242483	-2.241193	H	-3.287326	-1.271785	-2.332656
H	-2.538165	-2.155373	-1.018934	H	-2.539192	-2.159915	-0.983175
H	-4.053256	-1.335825	-0.592208	H	-4.076590	-1.291737	-0.753618
Fe	0.000000	-1.453366	0.298773	Fe	0.000000	-1.453718	0.302787
Fe	0.000000	1.453366	0.298773	Fe	0.000000	1.453718	0.302787
C	-2.396171	0.000000	-0.841742	C	-2.363861	0.000000	-0.864992
C	2.396171	0.000000	-0.841742	C	2.363861	0.000000	-0.864992
N	-1.240883	0.000000	-0.291091	N	-1.228742	0.000000	-0.275147
N	1.240883	0.000000	-0.291091	N	1.228742	0.000000	-0.275147

Table S48. Optimized coordinates for the $(Me_2C{=}N)_2Fe_2(CO)_6$ structure **6T-3.**

	M06-L				B3PW91-D3		
	x	y	z		x	y	z
C	-1.420429	-2.138447	0.741941	C	-1.447285	-2.133729	0.421955
C	-2.191071	-0.835674	-1.517150	C	-2.059632	-0.632193	-1.742602
C	1.763213	1.490817	1.327916	C	1.837745	1.255860	1.561361
C	1.780718	-1.213674	1.638660	C	1.796048	-1.399390	1.515330
O	-1.804919	-2.978825	1.438658	O	-1.873418	-3.041187	0.991522
O	-3.073559	-0.904313	-2.269865	O	-2.873808	-0.663658	-2.563513
O	1.950658	2.475603	1.918453	O	2.100426	2.109236	2.298685
O	1.952319	-2.002919	2.475664	O	2.010522	-2.269119	2.249226
C	1.266878	2.707419	-1.927650	C	1.417639	2.947052	-1.468753
C	-1.080699	1.928326	-2.596751	C	-0.901248	2.351987	-2.345032
H	-1.087942	2.921646	-3.052135	H	-1.007811	3.442194	-2.380175
H	-1.147071	1.187466	-3.400649	H	-0.741540	2.005787	-3.375318
H	0.935419	3.687349	-1.564369	H	1.104399	3.870299	-0.962672
H	1.525054	2.835139	-2.984721	H	1.692223	3.222178	-2.495627
C	0.296585	-1.932927	-1.179465	C	0.348430	-1.725643	-1.336350
C	3.055720	-0.150412	-0.545704	C	3.023164	-0.138314	-0.520938
O	0.951927	-2.704524	-1.749000	O	0.978245	-2.464647	-1.968311
O	4.079113	-0.212591	-1.093174	O	4.037366	-0.161301	-1.077532
H	2.156862	2.406623	-1.370634	H	2.291420	2.539885	-0.956172
H	-1.974694	1.815677	-1.975430	H	-1.828137	1.912304	-1.971792
C	-3.590363	1.475886	0.964147	C	-3.854035	1.202872	1.042312
H	-3.717141	2.540146	0.735666	H	-4.061292	2.280317	1.006501
H	-3.762672	0.891623	0.057532	H	-3.978887	0.777294	0.043652
H	-4.354232	1.216782	1.705052	H	-4.592525	0.752648	1.718419
C	-1.848560	1.978545	2.779833	C	-2.156740	1.509501	2.953807
H	-2.576960	1.769310	3.570691	H	-2.854921	1.082094	3.685294
H	-0.851542	1.706457	3.127736	H	-1.132647	1.276653	3.254153
H	-1.879768	3.057728	2.593062	H	-2.298733	2.598062	2.964146
Fe	1.461209	-0.005279	0.337623	Fe	1.446719	-0.056354	0.382447
Fe	-0.839776	-0.795589	-0.330941	Fe	-0.824913	-0.682360	-0.446243
C	-2.209386	1.221606	1.524136	C	-2.451048	0.956508	1.570992
C	0.162312	1.701437	-1.775516	C	0.276839	1.958108	-1.483949
N	-1.387358	0.433597	0.984559	N	-1.572821	0.336455	0.918460
N	0.276443	0.710655	-0.982816	N	0.319621	0.871620	-0.821480

Table S49. Optimized coordinates for the $(Me_2C{=}N)_2Fe_2(CO)_6$ structure **6S-4.**

	M06-L				B3PW91-D3		
	x	y	z		x	y	z
C	-2.398527	-1.418086	0.454868	C	-1.616151	2.225662	-0.088274
C	-0.795332	-0.627766	-1.566879	C	-0.085761	0.774757	-1.650364
C	-2.363238	1.474607	-0.454732	C	1.616239	2.225638	0.088338
C	-0.779857	0.646331	1.567094	C	0.085820	0.774670	1.650344
O	-3.519660	-1.481694	0.743640	O	-1.880999	3.330076	0.125398
O	-0.874312	-0.376191	-2.705504	O	0.455121	0.958845	-2.667044
O	-3.482631	1.565159	-0.743099	O	1.881107	3.330059	-0.125277
O	-0.865033	0.397112	2.705788	O	-0.455048	0.958711	2.667041
C	3.125140	1.620536	0.581678	C	1.846723	-3.403665	-0.418193
C	3.058712	1.695363	-1.992534	C	2.842211	-2.015486	-2.354544
H	3.564441	2.666779	-2.029173	H	3.834164	-2.469934	-2.230471
H	3.836359	0.927310	-2.083231	H	2.304705	-2.602873	-3.110483
H	3.479521	2.647471	0.730224	H	2.806531	-3.906173	-0.238998
H	4.017726	0.984703	0.526048	H	1.270173	-4.040589	-1.101793
C	-0.610938	-3.075686	-0.449308	C	-2.708300	0.339662	-1.446589
C	-0.536349	3.088975	0.448268	C	2.708350	0.339542	1.446551
O	-0.590206	-4.170643	-0.831628	O	-3.601664	0.231254	-2.169289
O	-0.489336	4.183333	0.829997	O	3.601707	0.231076	2.169252
H	2.520616	1.329626	1.444509	H	1.310382	-3.305375	0.528207
H	2.377553	1.615345	-2.840609	H	2.955022	-0.989431	-2.712061
C	3.084468	-1.690622	-0.581597	C	-1.846942	-3.403620	0.418276
H	3.412956	-2.725430	-0.734778	H	-2.806785	-3.906073	0.239116
H	2.487251	-1.380442	-1.442880	H	-1.310596	-3.305431	-0.528132
H	3.992443	-1.077172	-0.523284	H	-1.270434	-4.040537	1.101917
C	3.016796	-1.768584	1.992670	C	-2.842372	-2.015242	2.354513
H	3.810301	-1.017099	2.084890	H	-2.304944	-2.602640	3.110499
H	2.337439	-1.676041	2.840905	H	-2.955095	-0.989157	2.711973
H	3.502098	-2.750434	2.027326	H	-3.834364	-2.469602	2.230432
Fe	-0.594474	1.375209	-0.045859	Fe	1.303476	0.489867	0.373710
Fe	-0.627871	-1.361302	0.045908	Fe	-1.303422	0.489904	-0.373738
C	2.278977	-1.597067	0.690476	C	-2.090836	-2.044285	1.038636
C	2.317406	1.542224	-0.689992	C	2.090712	-2.044389	-1.038643
N	1.027518	-1.419563	0.673034	N	-1.705403	-0.977542	0.490420
N	1.062216	1.393257	-0.672295	N	1.705387	-0.977580	-0.490479

Table S50. Optimized coordinates for the $(Me_2C{=}N)_2Fe_2(CO)_6$ structure **6S-5.**

	M06-L				B3PW91-D3		
	x	y	z		x	y	z
C	0.066928	2.934430	0.189143	C	-0.123448	2.897481	-0.177141
C	-1.433916	0.610538	0.954613	C	1.365150	0.651063	-0.996353
C	-0.066928	-2.934430	0.189143	C	0.123448	-2.897481	-0.177141
C	1.433916	-0.610538	0.954613	C	-1.365150	-0.651063	-0.996353
O	-0.066928	4.066773	-0.072349	O	0.035017	4.021484	0.082715
O	-2.558946	0.753128	1.277923	O	2.451504	0.908858	-1.365687
O	0.066928	-4.066773	-0.072349	O	-0.035017	-4.021484	0.082715
O	2.558946	-0.753128	1.277923	O	-2.451504	-0.908858	-1.365687
C	-2.393784	-1.720317	-2.051878	C	2.451504	-1.647748	2.069909
C	-1.576371	0.327419	-3.338624	C	1.598893	0.415151	3.311241
H	-1.241443	-0.120554	-4.282101	H	1.280967	-0.021725	4.267526
H	-2.640739	0.545132	-3.458399	H	2.665213	0.646732	3.408567
H	-2.579052	-2.224153	-3.005617	H	2.622950	-2.132694	3.039133
H	-3.345661	-1.279892	-1.732070	H	3.401201	-1.188281	1.763733
C	0.660349	1.676526	2.247545	C	-0.771717	1.696141	-2.206195
C	-0.660349	-1.676526	2.247545	C	0.771717	-1.696141	-2.206195
O	0.937553	1.991483	3.332882	O	-1.088567	2.046883	-3.264555
O	-0.937553	-1.991483	3.332882	O	1.088567	-2.046883	-3.264555
H	-2.085548	-2.446014	-1.297174	H	2.171937	-2.391017	1.320773
H	-1.036393	1.263414	-3.186456	H	1.046701	1.342694	3.144629
C	2.393784	1.720317	-2.051878	C	-2.451504	1.647748	2.069909
H	2.085548	2.446014	-1.297174	H	-2.171937	2.391017	1.320773
H	3.345661	1.279892	-1.732070	H	-3.401201	1.188281	1.763733
H	2.579052	2.224153	-3.005617	H	-2.622950	2.132694	3.039133
C	1.576371	-0.327419	-3.338624	C	-1.598893	-0.415151	3.311241
H	2.640739	-0.545132	-3.458399	H	-2.665213	-0.646732	3.408567
H	1.036393	-1.263414	-3.186456	H	-1.046701	-1.342694	3.144629
H	1.241443	0.120554	-4.282101	H	-1.280967	0.021725	4.267526
C	1.386295	0.636483	-2.215075	C	-1.412432	0.579080	2.202615
C	-1.386295	-0.636483	-2.215075	C	1.412432	-0.579080	2.202615
N	-0.436224	-0.553275	-1.335684	N	0.455322	-0.535612	1.332796
N	0.436224	0.553275	-1.335684	N	-0.455322	0.535612	1.332796
Fe	-0.245002	-1.225628	0.588062	Fe	0.311778	-1.200747	-0.581468
Fe	0.245002	1.225628	0.588062	Fe	-0.311778	1.200747	-0.581468

Table S51. Optimized coordinates for the $(Me_2C{=}N)_2Fe_2(CO)_6$ structure **6T-6.**

	M06-L				B3PW91-D3		
	x	y	z		x	y	z
C	0.026773	3.089369	0.319821	C	-0.037141	3.074369	-0.311279
C	-1.774740	1.032343	0.732792	C	1.797966	1.176916	-0.793171
C	-0.026773	-3.089369	0.319821	C	0.037141	-3.074369	-0.311279
C	1.774740	-1.032343	0.732792	C	-1.797966	-1.176916	-0.793171
O	-0.018703	4.250230	0.207120	O	-0.007065	4.233305	-0.222938
O	-2.917130	0.813526	0.830708	O	2.941137	1.081761	-0.973926
O	0.018703	-4.250230	0.207120	O	0.007065	-4.233305	-0.222938
O	2.917130	-0.813526	0.830708	O	-2.941137	-1.081761	-0.973926
C	-2.334280	-1.805311	-1.986472	C	2.298787	-1.852728	2.027644
C	-1.651703	0.328551	-3.223065	C	1.672377	0.327655	3.208011
H	-1.343150	-0.071845	-4.197059	H	1.333341	-0.042239	4.185942
H	-2.725928	0.521554	-3.291785	H	2.756943	0.466446	3.281106
H	-2.598171	-2.232981	-2.958512	H	2.539253	-2.246703	3.022507
H	-3.263974	-1.444705	-1.530275	H	3.243163	-1.527540	1.569508
C	0.563420	1.352975	2.268675	C	-0.564686	1.365431	-2.221753
C	-0.563420	-1.352975	2.268675	C	0.564686	-1.365431	-2.221753
O	0.902287	1.401093	3.380672	O	-0.925093	1.450264	-3.320690
O	-0.902287	-1.401093	3.380672	O	0.925093	-1.450264	-3.320690
H	-1.922230	-2.581165	-1.337001	H	1.874879	-2.644882	1.406444
H	-1.130020	1.272400	-3.054983	H	1.200817	1.293962	3.014968
C	2.334280	1.805311	-1.986472	C	-2.298787	1.852728	2.027644
H	1.922230	2.581165	-1.337001	H	-1.874879	2.644882	1.406444
H	3.263974	1.444705	-1.530275	H	-3.243163	1.527540	1.569508
H	2.598171	2.232981	-2.958512	H	-2.539253	2.246703	3.022507
C	1.651703	-0.328551	-3.223065	C	-1.672377	-0.327655	3.208011
H	2.725928	-0.521554	-3.291785	H	-2.756943	-0.466446	3.281106
H	1.130020	-1.272400	-3.054983	H	-1.200817	-1.293962	3.014968
H	1.343150	0.071845	-4.197059	H	-1.333341	0.042239	4.185942
C	1.385970	0.668950	-2.146008	C	-1.371692	0.684044	2.141628
C	-1.385970	-0.668950	-2.146008	C	1.371692	-0.684044	2.141628
N	-0.406405	-0.575860	-1.296762	N	0.397468	-0.578632	1.290821
N	0.406405	0.575860	-1.296762	N	-0.397468	0.578632	1.290821
Fe	-0.018703	-1.318688	0.550172	Fe	-0.007065	-1.314288	-0.526241
Fe	0.018703	1.318688	0.550172	Fe	0.007065	1.314288	-0.526241

Table S52. Optimized coordinates for the $(Me_2C{=}N)_2Fe_2(CO)_6$ structure **6T-7.**

	M06-L				B3PW91-D3		
	x	y	z		x	y	z
C	-0.234609	1.944070	0.926302	C	4.778481	-1.066040	-1.429560
C	-0.979508	0.786687	-1.413156	C	5.027577	-0.101211	1.046131
C	2.464448	1.025181	-1.159263	C	-2.680200	1.338340	0.192534
C	3.407889	0.726044	1.279287	C	-4.778489	1.066045	-1.429558
O	0.616256	2.598964	1.368537	O	5.385129	-1.708728	-2.176629
O	-0.668946	0.692085	-2.528776	O	5.755974	-0.135429	1.943795
O	2.448477	1.848958	-1.978770	O	-1.934280	2.148375	0.553304
O	3.985634	1.350803	2.070032	O	-5.385142	1.708729	-2.176626
C	-0.395399	-2.850805	1.426578	C	-1.977952	-3.623200	-0.799229
C	-0.449351	-2.616152	-1.132980	C	-1.775350	-2.607714	1.564488
H	-0.404800	-3.701979	-1.271416	H	-2.147066	-3.531175	2.027389
H	-1.510594	-2.332427	-1.132184	H	-0.679644	-2.667578	1.551203
H	-0.462289	-3.939212	1.322839	H	-2.384079	-4.565970	-0.410065
H	-1.412073	-2.466195	1.577440	H	-0.890037	-3.744781	-0.879434
C	-2.738319	2.353804	0.095635	C	2.680200	-1.338341	0.192521
C	3.901560	-1.260337	-0.488674	C	-5.027583	0.101190	1.046126
O	-3.471145	3.234929	-0.087822	O	1.934283	-2.148382	0.553284
O	4.800632	-1.875126	-0.888415	O	-5.755981	0.135389	1.943789
H	0.206859	-2.601131	2.300224	H	-2.397100	-3.428252	-1.789116
H	0.050958	-2.123679	-1.967689	H	-2.086514	-1.749375	2.164806
C	-4.388961	-1.074431	-1.100661	C	1.775324	2.607715	1.564485
H	-5.426693	-0.750452	-0.963647	H	0.679619	2.667576	1.551171
H	-3.879387	-0.361744	-1.752562	H	2.086475	1.749373	2.164805
H	-4.426606	-2.054369	-1.590339	H	2.147026	3.531174	2.027402
C	-4.190105	-2.241822	1.175678	C	1.977989	3.623221	-0.799218
H	-3.977251	-3.235261	0.761005	H	2.384101	4.565988	-0.410032
H	-3.714464	-2.155588	2.153901	H	2.397167	3.428284	-1.789094
H	-5.276429	-2.175295	1.297030	H	0.890076	3.744799	-0.879455
Fe	2.472654	-0.266663	0.100494	Fe	-3.825413	0.034825	-0.303313
Fe	-1.607846	0.939380	0.277458	Fe	3.825412	-0.034821	-0.303311
C	-3.697337	-1.174534	0.233943	C	2.298295	2.491276	0.151074
C	0.177077	-2.232756	0.180660	C	-2.298285	-2.491264	0.151065
N	-2.737314	-0.408722	0.560632	N	2.973196	1.493550	-0.242123
N	1.136771	-1.406694	0.247975	N	-2.973179	-1.493537	-0.242140

Table S53. Optimized coordinates for the $(Me_2C{=}N)_2Fe_2(CO)_6$ structure **6S-8.**

	M06-L				B3PW91-D3		
	x	y	z		x	y	z
C	2.658629	0.984613	-1.310273	C	-2.722977	-0.925680	-1.316244
C	2.102630	0.412804	1.734898	C	-2.012726	-0.461416	1.735966
C	-3.578334	0.286193	-1.027881	C	3.635372	-0.125490	-0.908045
C	-2.838349	-1.550395	0.895145	C	2.656138	1.471827	1.104499
O	2.765363	1.846951	-2.096731	O	-2.887547	-1.723934	-2.151339
O	1.862072	0.916492	2.763477	O	-1.747737	-0.974012	2.748683
O	-4.369238	0.471789	-1.857776	O	4.503984	-0.184923	-1.668845
O	-3.209690	-2.539122	1.373666	O	2.937512	2.409142	1.715810
C	-0.254664	2.288731	0.253556	C	0.309868	-2.364322	-0.061953
C	-0.827812	1.506747	-2.101912	C	0.984794	-1.358940	-2.301663
H	-1.472538	2.368367	-2.304315	H	1.649207	-2.201737	-2.526606
H	0.177634	1.763342	-2.463835	H	0.002728	-1.592324	-2.739702
H	-0.841012	3.205011	0.125471	H	0.955953	-3.234504	-0.231324
H	0.785874	2.526835	-0.013978	H	-0.693671	-2.624718	-0.427220
C	4.213917	0.060216	0.434171	C	-4.156783	-0.113383	0.546199
C	-3.008327	1.156412	1.303967	C	2.943706	-1.225288	1.257752
O	5.354891	0.121542	0.663552	O	-5.279183	-0.201041	0.831635
O	-3.417398	1.936357	2.058681	O	3.348686	-2.037565	1.971321
H	-0.269052	2.012461	1.311622	H	0.237983	-2.186532	1.014800
H	-1.195240	0.658914	-2.688793	H	1.378273	-0.466021	-2.798924
C	2.057104	-2.628508	-0.485107	C	-2.058025	2.647709	-0.486759
H	2.754827	-1.935385	-1.011683	H	-2.837348	1.876768	-0.707034
H	2.379175	-2.681463	0.566213	H	-2.217073	3.026919	0.531200
H	2.178154	-3.616324	-0.932253	H	-2.226434	3.472115	-1.188139
C	-0.491893	-2.947388	-0.911476	C	0.461257	2.933309	-1.016787
H	-0.901141	-3.428596	-0.014351	H	0.809018	3.522709	-0.157106
H	-1.301332	-2.365693	-1.362708	H	1.306617	2.350931	-1.391385
H	-0.185533	-3.745957	-1.591910	H	0.132070	3.645479	-1.782129
C	0.673070	-2.093334	-0.574008	C	-0.686997	2.063161	-0.625495
C	-0.801090	1.192502	-0.625210	C	0.860966	-1.162274	-0.801951
N	-0.550156	-0.144543	-0.276208	N	0.555817	0.131350	-0.355668
N	0.640133	-0.806860	-0.324175	N	-0.643316	0.787793	-0.368020
Fe	-2.264722	0.016607	0.143375	Fe	2.226436	-0.020262	0.161942
Fe	2.493301	-0.099119	0.080818	Fe	-2.464055	0.074120	0.112602

Table S54. Optimized coordinates for the $(Me_2C{=}N)_2Fe_2(CO)_6$ structure **6T-9.**

	M06-L				B3PW91-D3		
	x	y	z		x	y	z
C	3.464035	-0.209303	-1.216022	C	3.644031	-0.035892	-1.181846
C	2.866671	1.977371	0.436256	C	2.820617	1.898189	0.599254
C	-3.652089	0.047168	-1.020797	C	-3.751426	-0.046270	-0.841704
C	-2.674495	-1.191058	1.517313	C	-2.447285	-1.195926	1.582863
O	4.159577	-0.488513	-2.111473	O	4.436112	-0.142711	-2.027593
O	3.209715	3.082371	0.575990	O	3.107210	2.990927	0.867631
O	-4.526101	0.116718	-1.784313	O	-4.708908	-0.025165	-1.493109
O	-3.016556	-1.851641	2.407745	O	-2.659037	-1.843895	2.515949
C	-0.844716	2.509096	-0.618191	C	-1.064497	2.553246	-0.755679
C	-0.387933	0.658503	-2.264844	C	-0.608367	0.721808	-2.426760
H	-1.042545	1.170383	-2.975072	H	-1.349778	1.204618	-3.071530
H	0.641327	0.996610	-2.461781	H	0.380167	1.119457	-2.701350
H	-1.672847	2.974654	-1.161633	H	-1.970793	2.939888	-1.234450
H	0.088897	2.967580	-0.977266	H	-0.202819	3.071883	-1.202592
C	3.387241	-0.537985	1.533309	C	3.403807	-0.647162	1.486934
C	-2.908873	1.279320	1.010543	C	-2.884114	1.209528	1.096363
O	4.050779	-1.014629	2.365206	O	4.041834	-1.150956	2.317822
O	-3.356958	2.163710	1.613585	O	-3.316779	2.054205	1.754218
H	-0.930971	2.747045	0.444061	H	-1.075349	2.805346	0.307499
H	-0.421036	-0.412232	-2.483460	H	-0.603127	-0.346743	-2.654702
C	1.330523	-2.957543	-0.600635	C	1.276884	-2.884753	-0.791231
H	2.277387	-2.598372	-0.190529	H	2.265857	-2.499577	-0.529946
H	1.051157	-3.895335	-0.108787	H	1.054972	-3.760289	-0.166584
H	1.474222	-3.183811	-1.663920	H	1.285666	-3.224140	-1.835568
C	-1.129387	-2.344127	-0.785956	C	-1.183490	-2.246802	-0.884817
H	-1.611417	-2.951858	-0.017247	H	-1.592224	-2.916673	-0.124627
H	-1.796757	-1.501792	-1.083293	H	-1.899479	-1.413471	-1.115687
H	-1.100897	-2.956697	-1.695625	H	-1.199117	-2.804388	-1.832612
C	0.262914	-1.930783	-0.429576	C	0.231447	-1.830227	-0.594740
C	-0.772803	1.018546	-0.851304	C	-0.892930	1.061700	-0.976758
N	-0.308389	0.287688	0.239880	N	-0.336131	0.369798	0.073111
N	0.606420	-0.744112	-0.048922	N	0.589552	-0.643945	-0.236119
Fe	-2.266734	-0.075534	0.116783	Fe	-2.260454	-0.110973	0.135430
Fe	2.387776	0.223974	0.200269	Fe	2.439705	0.162482	0.170439

Table S55. Optimized coordinates for the $(Me_2C{=}N)_2Cr_2(CO)_8$ structure **8S-1.**

	M06-L				B3PW91-D3		
	x	y	z		x	y	z
C	1.136378	2.818337	-0.381817	C	0.759589	2.989697	-0.072068
C	-1.164779	2.793595	0.416946	C	-1.474274	2.418030	0.747516
C	0.347931	-1.418069	-1.893127	C	0.326819	-1.558837	-1.777832
C	1.164779	-2.793595	0.416946	C	1.474274	-2.418030	0.747516
O	1.743281	3.760793	-0.696369	O	1.202441	4.052682	-0.211800
O	-1.788892	3.718160	0.750334	O	-2.282564	3.085340	1.244462
O	0.620803	-1.541217	-3.013511	O	0.450192	-1.743898	-2.911637
O	1.788892	-3.718160	0.750334	O	2.282564	-3.085340	1.244462
C	-3.628165	1.223949	-0.318784	C	-3.581808	0.947100	-0.873603
C	-3.628165	-1.244242	0.243038	C	-3.436705	-1.557781	-0.452828
H	-3.858383	-1.763234	-0.694520	H	-3.143332	-2.143045	-1.333738
H	-3.100962	-1.938750	0.899623	H	-3.172211	-2.131479	0.439753
H	-3.915220	1.726815	0.611849	H	-4.266003	1.218046	-0.058759
H	-4.553753	0.947224	-0.832192	H	-4.198584	0.657525	-1.733240
C	0.355998	1.406849	1.903118	C	0.450192	1.286520	1.924887
C	-1.136378	-2.818337	-0.381817	C	-0.759589	-2.989697	-0.072068
O	0.641564	1.527906	3.020562	O	0.875831	1.376421	2.995550
O	-1.743281	-3.760793	-0.696369	O	-1.202441	-4.052682	-0.211800
H	-3.077621	1.933905	-0.938479	H	-2.988678	1.822365	-1.142174
H	-4.582763	-0.974398	0.704030	H	-4.522701	-1.423130	-0.479178
C	3.628165	1.244242	0.243038	C	3.436705	1.557781	-0.452828
H	3.100962	1.938750	0.899623	H	3.172211	2.131479	0.439753
H	4.582763	0.974398	0.704030	H	4.522701	1.423130	-0.479178
H	3.858383	1.763234	-0.694520	H	3.143332	2.143045	-1.333738
C	3.628165	-1.223949	-0.318784	C	3.581808	-0.947100	-0.873603
H	3.915220	-1.726815	0.611849	H	4.266003	-1.218046	-0.058759
H	3.077621	-1.933905	-0.938479	H	2.988678	-1.822365	-1.142174
H	4.553753	-0.947224	-0.832192	H	4.198584	-0.657525	-1.733240
C	2.812731	0.007063	-0.025043	C	2.722275	0.223332	-0.462070
C	-2.812731	-0.007063	-0.025043	C	-2.722275	-0.223332	-0.462070
N	1.536329	0.004471	-0.006738	N	1.485663	0.131454	-0.166115
N	-1.536329	-0.004471	-0.006738	N	-1.485663	-0.131454	-0.166115
O	-0.641564	-1.527906	3.020562	O	-0.875831	-1.376421	2.995550
C	-0.355998	-1.406849	1.903118	C	-0.450192	-1.286520	1.924887
C	-0.347931	1.418069	-1.893127	C	-0.326819	1.558837	-1.777832
O	-0.620803	1.541217	-3.013511	O	-0.450192	1.743898	-2.911637
Cr	-0.002726	1.364172	0.005947	Cr	-0.095033	1.341795	0.097230
Cr	0.002726	-1.364172	0.005947	Cr	0.095033	-1.341795	0.097230

Table S56. Optimized coordinates for the $(Me_2C{=}N)_2Cr_2(CO)_8$ structure **8T-2.**

	M06-L				B3PW91-D3		
	x	y	z		x	y	z
C	-0.103473	3.326401	0.090672	C	1.032410	3.170624	-0.102630
C	-2.252373	2.141725	0.683757	C	-1.326190	2.872006	0.590920
C	0.670310	-1.613184	-1.780935	C	0.088312	-1.658538	-1.818102
C	2.252373	-2.141725	0.683757	C	1.326190	-2.872006	0.590920
O	0.054534	4.475469	0.019773	O	1.568365	4.181007	-0.281793
O	-3.240584	2.578018	1.111021	O	-2.063877	3.663903	1.001764
O	0.706084	-1.706245	-2.936124	O	0.109449	-1.698233	-2.971435
O	3.240584	-2.578018	1.111021	O	2.063877	-3.663903	1.001764
C	-3.559585	-0.051090	-0.992485	C	-3.440391	1.164750	-0.770327
C	-2.683436	-2.413043	-0.708413	C	-3.440391	-1.329034	-0.321373
H	-2.060282	-2.851175	-1.497544	H	-3.270334	-1.914568	-1.234755
H	-2.447295	-2.932623	0.224252	H	-3.157061	-1.940881	0.539665
H	-4.314031	-0.007205	-0.197643	H	-4.007808	1.549773	0.087713
H	-4.057326	-0.466061	-1.874856	H	-4.169612	0.885502	-1.540637
C	-0.234126	1.400585	2.049864	C	0.218129	1.475862	1.988902
C	0.103473	-3.326401	0.090672	C	-1.032410	-3.170624	-0.102630
O	-0.054534	1.535531	3.187411	O	0.399141	1.514425	3.128224
O	-0.054534	-4.475469	0.019773	O	-1.568365	-4.181007	-0.281793
H	-3.237020	0.967229	-1.217402	H	-2.810677	1.965805	-1.163413
H	-3.728903	-2.612897	-0.956201	H	-4.513197	-1.118073	-0.256189
C	2.683436	2.413043	-0.708413	C	3.440391	1.329034	-0.321373
H	2.447295	2.932623	0.224252	H	3.157061	1.940881	0.539665
H	3.728903	2.612897	-0.956201	H	4.513197	1.118073	-0.256189
H	2.060282	2.851175	-1.497544	H	3.270334	1.914568	-1.234755
C	3.559585	0.051090	-0.992485	C	3.440391	-1.164750	-0.770327
H	4.314031	0.007205	-0.197643	H	4.007808	-1.549773	0.087713
H	3.237020	-0.967229	-1.217402	H	2.810677	-1.965805	-1.163413
H	4.057326	0.466061	-1.874856	H	4.169612	-0.885502	-1.540637
C	2.406879	0.931334	-0.586408	C	2.627821	0.047826	-0.367222
C	-2.406879	-0.931334	-0.586408	C	-2.627821	-0.047826	-0.367222
N	1.278119	0.484335	-0.182752	N	1.377963	0.016306	-0.106692
N	-1.278119	-0.484335	-0.182752	N	-1.377963	-0.016306	-0.106692
O	0.054534	-1.535531	3.187411	O	-0.399141	-1.514425	3.128224
C	0.234126	-1.400585	2.049864	C	-0.218129	-1.475862	1.988902
C	-0.670310	1.613184	-1.780935	C	-0.088312	1.658538	-1.818102
O	-0.706084	1.706245	-2.936124	O	-0.109449	1.698233	-2.971435
Cr	-0.540256	1.459253	0.133571	Cr	-0.011289	1.592928	0.087474
Cr	0.540256	-1.459253	0.133571	Cr	0.011289	-1.592928	0.087474

Table S57. Optimized coordinates for the $(Me_2C=N)_2Cr_2(CO)_8$ structure **8S-3.**

	M06-L				B3PW91-D3		
	x	y	z		x	y	z
C	-0.522095	2.983435	-0.438342	C	-0.585406	3.003680	-0.393113
C	1.857202	2.178518	0.089394	C	1.754037	2.223080	0.178487
C	0.522095	-2.983435	-0.438342	C	0.585406	-3.003680	-0.393113
C	-1.857202	-2.178518	0.089394	C	-1.754037	-2.223080	0.178487
O	-1.016750	4.043693	-0.443176	O	-1.086018	4.056663	-0.360261
O	2.808594	2.751834	0.432356	O	2.677434	2.813225	0.556060
O	1.016750	-4.043693	-0.443176	O	1.086018	-4.056663	-0.360261
O	-2.808594	-2.751834	0.432356	O	-2.677434	-2.813225	0.556060
C	2.068279	-2.086711	2.193644	C	2.254000	-1.896441	2.113705
C	1.707882	0.180061	3.321322	C	1.703186	0.343362	3.240729
H	1.423009	-0.170003	4.320764	H	1.440215	-0.026520	4.241144
H	2.798825	0.264662	3.324189	H	2.786208	0.511851	3.236051
H	2.572550	-2.304430	3.137734	H	2.844349	-2.020228	3.026329
H	2.839805	-1.995821	1.418764	H	2.947004	-1.765230	1.271306
C	0.736834	1.624465	-2.277115	C	0.760562	1.728507	-2.211089
C	-0.736834	-1.624465	-2.277115	C	-0.760562	-1.728507	-2.211089
O	1.016750	1.825348	-3.389874	O	1.086018	1.974922	-3.297451
O	-1.016750	-1.825348	-3.389874	O	-1.086018	-1.974922	-3.297451
H	1.432004	-2.928922	1.918652	H	1.679183	-2.806143	1.925169
H	1.275147	1.168258	3.157749	H	1.194044	1.293390	3.066453
C	-2.068279	2.086711	2.193644	C	-2.254000	1.896441	2.113705
H	-1.432004	2.928922	1.918652	H	-1.679183	2.806143	1.925169
H	-2.839805	1.995821	1.418764	H	-2.947004	1.765230	1.271306
H	-2.572550	2.304430	3.137734	H	-2.844349	2.020228	3.026329
C	-1.707882	-0.180061	3.321322	C	-1.703186	-0.343362	3.240729
H	-2.798825	-0.264662	3.324189	H	-2.786208	-0.511851	3.236051
H	-1.275147	-1.168258	3.157749	H	-1.194044	-1.293390	3.066453
H	-1.423009	0.170003	4.320764	H	-1.440215	0.026520	4.241144
C	-1.292622	0.815692	2.290275	C	-1.365391	0.695097	2.215214
C	1.292622	-0.815692	2.290275	C	1.365391	-0.695097	2.215214
N	0.328865	-0.623816	1.443632	N	0.381493	-0.591117	1.382254
N	-0.328865	0.623816	1.443632	N	-0.381493	0.591117	1.382254
O	-2.587103	0.799586	-1.544070	O	-2.529619	0.814211	-1.698973
C	-1.489590	0.799059	-1.142250	C	-1.468182	0.855701	-1.225139
C	1.489590	-0.799059	-1.142250	C	1.468182	-0.855701	-1.225139
O	2.587103	-0.799586	-1.544070	O	2.529619	-0.814211	-1.698973
Cr	0.252531	1.317021	-0.501947	Cr	0.200767	1.365019	-0.481250
Cr	-0.252531	-1.317021	-0.501947	Cr	-0.200767	-1.365019	-0.481250

Table S58. Optimized coordinates for the $(Me_2C{=}N)_2Cr_2(CO)_8$ structure **8S-4.**

	M06-L				B3PW91-D3		
	x	y	z		x	y	z
C	-2.744843	-0.820706	0.884990	C	2.686460	0.922727	0.866380
C	-1.417614	1.370467	0.978453	C	1.436312	-1.297744	1.014559
C	2.434119	0.983273	-0.750016	C	-2.366475	-1.064668	-0.774628
C	0.134646	0.900189	-1.754314	C	-0.069320	-0.996098	-1.716334
O	-3.577229	-1.224460	1.598820	O	3.506945	1.380761	1.552988
O	-1.807203	2.202261	1.701876	O	1.858901	-2.100519	1.744084
O	3.466852	0.844504	-1.280853	O	-3.391768	-0.980330	-1.319719
O	0.036964	0.971564	-2.916097	O	0.049579	-1.137364	-2.866000
C	-0.345302	-0.878066	2.786579	C	0.294523	0.954758	2.765242
C	-0.404155	-3.034813	1.434455	C	0.363313	3.051318	1.335008
H	0.437638	-3.568050	1.896111	H	-0.495894	3.560693	1.794341
H	-1.299115	-3.274952	2.013728	H	1.256048	3.314937	1.910166
H	0.281893	-1.395453	3.525754	H	-0.385187	1.500451	3.436571
H	-1.377453	-0.950925	3.141319	H	1.308294	1.080019	3.157658
C	-2.612508	0.807992	-1.206335	C	2.630102	-0.763994	-1.133979
C	0.607656	2.936065	-0.312491	C	-0.528423	-2.938865	-0.218676
O	-3.380220	1.420150	-1.828343	O	3.433822	-1.362014	-1.713445
O	0.485333	4.071975	-0.539291	O	-0.381975	-4.075930	-0.398826
H	-0.047541	0.170681	2.759229	H	0.027712	-0.101607	2.790661
H	-0.510787	-3.404954	0.413186	H	0.462250	3.413804	0.310101
C	2.786632	-2.979008	-1.572134	C	-2.873712	2.922210	-1.585935
H	1.996916	-3.240532	-2.276632	H	-2.101063	3.168227	-2.318523
H	3.558850	-2.407545	-2.099485	H	-3.668887	2.349452	-2.081038
H	3.266360	-3.890103	-1.199586	H	-3.328062	3.844236	-1.201641
C	3.186098	-1.836460	0.680740	C	-3.224261	1.764151	0.673536
H	4.162507	-1.540184	0.288073	H	-4.113375	1.258294	0.279610
H	2.804760	-1.041902	1.326287	H	-2.754371	1.119692	1.418633
H	3.349471	-2.733845	1.289432	H	-3.565036	2.689118	1.156024
C	2.250824	-2.163332	-0.442700	C	-2.301852	2.108670	-0.462934
C	-0.147726	-1.562179	1.470703	C	0.145886	1.563972	1.396020
N	0.552983	-0.951974	0.514728	N	-0.559805	0.928431	0.452786
N	1.015684	-1.801087	-0.532017	N	-1.070029	1.759829	-0.577723
O	-2.058956	-2.543691	-2.036965	O	1.997812	2.474927	-2.110341
C	-1.763457	-1.660757	-1.346313	C	1.709014	1.628757	-1.379654
C	1.762480	1.630844	1.604074	C	-1.762284	-1.583267	1.577313
O	2.419140	1.972165	2.501413	O	-2.449504	-1.890247	2.458656
Cr	0.781756	1.117976	0.055531	Cr	-0.742915	-1.123533	0.064875
Cr	-1.391869	-0.173293	-0.192823	Cr	1.365423	0.201826	-0.177070

Table S59. Optimized coordinates for the $(Me_2C{=}N)_2Cr_2(CO)_8$ structure **8S-5.**

	M06-L				B3PW91-D3		
	x	y	z		x	y	z
C	3.084379	-0.523986	-0.278273	C	3.078327	-0.513930	-0.255489
C	1.919408	1.667835	0.574841	C	1.919614	1.670860	0.498541
C	-2.590102	-1.118282	-0.823997	C	-2.542597	-1.133833	-0.801882
C	-1.446719	-1.908445	1.450808	C	-1.397927	-1.888877	1.467947
O	4.150191	-0.759183	-0.697577	O	4.142194	-0.758005	-0.658589
O	2.187425	2.800645	0.648916	O	2.203668	2.797239	0.533690
O	-3.462052	-1.514235	-1.484199	O	-3.401127	-1.537410	-1.467882
O	-1.554655	-2.856851	2.124126	O	-1.480758	-2.828559	2.150193
C	-0.366871	3.506802	-1.929567	C	-0.396633	3.465789	-1.948930
C	-2.566567	2.618851	-1.035091	C	-2.610663	2.577122	-1.071050
H	-2.679876	3.059927	-0.037413	H	-2.734577	3.014192	-0.070776
H	-2.980323	3.332282	-1.752512	H	-3.008903	3.293058	-1.797840
H	-0.752787	4.454433	-1.543401	H	-0.809507	4.414351	-1.587830
H	-0.540958	3.508512	-3.011428	H	-0.541520	3.435007	-3.037034
C	2.239650	-0.213829	2.173555	C	2.207063	-0.105975	2.172087
C	-2.716408	0.398562	1.166646	C	-2.710144	0.365829	1.142774
O	2.767184	-0.270871	3.203271	O	2.719399	-0.108302	3.205164
O	-3.628032	0.880071	1.716562	O	-3.638901	0.830201	1.669931
H	0.709748	3.451521	-1.771920	H	0.676285	3.432117	-1.753339
H	-3.142069	1.691385	-1.052418	H	-3.186595	1.649826	-1.104704
C	1.713250	-2.080946	-2.517909	C	1.724864	-2.107422	-2.486963
H	2.127220	-1.076684	-2.613804	H	2.121262	-1.101595	-2.631837
H	2.499584	-2.744628	-2.139662	H	2.523318	-2.747716	-2.088588
H	1.425084	-2.461744	-3.502182	H	1.420199	-2.531274	-3.450803
C	-0.402243	-3.274498	-1.754994	C	-0.371640	-3.300812	-1.664055
H	0.158073	-4.197620	-1.930181	H	0.200500	-4.212506	-1.870958
H	-1.053100	-3.391259	-0.886736	H	-0.977262	-3.432721	-0.765322
H	-1.028387	-3.110245	-2.640518	H	-1.042976	-3.134442	-2.518158
C	0.535947	-2.115749	-1.592594	C	0.556720	-2.122553	-1.540052
C	-1.123077	2.373244	-1.314987	C	-1.156345	2.329056	-1.326023
N	0.295552	-1.209220	-0.709288	N	0.315313	-1.193866	-0.682293
N	-0.613632	1.220224	-0.990314	N	-0.643040	1.189602	-0.980497
O	-0.347720	1.187990	2.793736	O	-0.386498	1.239019	2.746476
C	-0.421132	0.544589	1.813628	C	-0.460006	0.571613	1.786260
C	0.765328	0.964520	-1.282273	C	0.738305	0.922845	-1.266006
O	1.322356	1.212215	-2.319988	O	1.285924	1.146242	-2.314100
Cr	-1.259210	-0.421172	0.356067	Cr	-1.241376	-0.421433	0.364292
Cr	1.436615	-0.123126	0.435677	Cr	1.434252	-0.106488	0.433820

Table S60. Optimized coordinates for the $(Me_2C{=}N)_2Cr_2(CO)_8$ structure **8T-6.**

	M06-L				B3PW91-D3		
	x	y	z		x	y	z
C	3.193806	0.104066	-0.724284	C	3.118759	0.287069	-0.798748
C	2.047304	1.810191	1.129437	C	2.021916	1.717191	1.233696
C	-2.484952	-1.312449	-0.737448	C	-2.501955	-1.204603	-0.790960
C	-0.896332	-2.192825	1.253947	C	-1.033624	-2.190776	1.167766
O	4.244228	0.071411	-1.216089	O	4.130694	0.426062	-1.334411
O	2.359989	2.791207	1.658230	O	2.351553	2.660471	1.804353
O	-3.402179	-1.722472	-1.320934	O	-3.417939	-1.578388	-1.393758
O	-0.736476	-3.217377	1.789895	O	-0.963013	-3.234096	1.678844
C	-1.392414	3.661499	-1.470994	C	-1.296103	3.740825	-1.409450
C	-3.249358	2.141942	-0.672156	C	-3.206311	2.246493	-0.706227
H	-3.465870	2.543694	0.325029	H	-3.453397	2.668102	0.277992
H	-3.853159	2.714741	-1.381902	H	-3.757354	2.830845	-1.452449
H	-2.058369	4.422964	-1.054986	H	-1.971356	4.496194	-0.991491
H	-1.502357	3.692782	-2.560256	H	-1.376379	3.792200	-2.503226
C	2.307478	-0.792306	1.747714	C	2.398170	-0.846054	1.672343
C	-2.556541	-0.104089	1.444305	C	-2.568537	-0.052218	1.385090
O	2.798536	-1.284568	2.668395	O	2.975348	-1.343541	2.532074
O	-3.448085	0.147722	2.156051	O	-3.460728	0.202196	2.089448
H	-0.353403	3.906387	-1.252458	H	-0.261510	3.974157	-1.154027
H	-3.553526	1.094633	-0.684002	H	-3.540613	1.209214	-0.728499
C	1.791830	-1.641490	-2.862754	C	1.919523	-1.903458	-2.666228
H	2.108024	-0.598667	-2.930804	H	2.253744	-0.882444	-2.865704
H	2.640056	-2.233356	-2.497807	H	2.717150	-2.432718	-2.127070
H	1.550321	-2.009111	-3.863976	H	1.770634	-2.418812	-3.620955
C	-0.354024	-2.883412	-2.303132	C	-0.352733	-2.958149	-2.260939
H	0.180598	-3.742726	-2.719631	H	0.169611	-3.894643	-2.492812
H	-0.938406	-3.205767	-1.439179	H	-1.092824	-3.145306	-1.482270
H	-1.045688	-2.534386	-3.079812	H	-0.869871	-2.645938	-3.179335
C	0.613085	-1.793295	-1.938065	C	0.645096	-1.905070	-1.847798
C	-1.797831	2.308742	-0.973935	C	-1.732844	2.375448	-0.948409
N	0.445208	-1.045272	-0.908468	N	0.439737	-1.085694	-0.882419
N	-0.985518	1.316154	-0.770450	N	-0.945448	1.366170	-0.737276
O	-0.132458	0.787046	2.930182	O	-0.155065	0.559242	2.979048
C	-0.277216	0.312862	1.863263	C	-0.308488	0.180792	1.877630
C	0.425669	1.459337	-1.048896	C	0.472085	1.482870	-1.001204
O	0.851993	2.027041	-2.035758	O	0.911846	2.096526	-1.953900
Cr	-1.104259	-0.567721	0.374767	Cr	-1.128035	-0.545584	0.341975
Cr	1.502865	0.183551	0.192335	Cr	1.483070	0.143746	0.199890

Table S61. Optimized coordinates for the $(Me_2C{=}N)_2Cr_2(CO)_8$ structure **8S-7.**

	M06-L				B3PW91-D3		
	x	y	z		x	y	z
C	-3.360706	-1.135423	0.404588	C	3.366231	-1.184356	-0.493325
C	-1.965870	0.783560	1.438004	C	2.218190	0.896607	-1.460258
C	3.360708	-1.135413	-0.404616	C	-3.366228	-1.184360	0.493319
C	1.965875	0.783594	-1.437985	C	-2.218200	0.896607	1.460258
O	-4.127679	-1.876657	0.879199	O	4.091784	-1.953532	-0.979255
O	-1.746847	1.067509	2.555349	O	2.129265	1.300783	-2.551574
O	4.127678	-1.876639	-0.879244	O	-4.091781	-1.953537	0.979246
O	1.746862	1.067573	-2.555324	O	-2.129284	1.300786	2.551574
C	1.436244	-1.342283	2.603737	C	-1.676897	-1.381024	-2.453135
C	-0.818196	-2.445792	2.006058	C	0.637879	-2.420722	-2.031556
H	-0.505849	-3.283519	2.635250	H	0.243588	-3.257400	-2.619479
H	-1.512120	-1.837269	2.599944	H	1.289865	-1.834476	-2.693178
H	1.752235	-2.242921	3.136549	H	-2.274472	-2.299365	-2.501376
H	1.072547	-0.624908	3.348160	H	-1.343281	-1.144066	-3.470959
C	-3.754463	1.224509	-0.479618	C	3.926378	1.060417	0.514524
C	3.754459	1.224502	0.479649	C	-3.926377	1.060412	-0.514534
O	-4.727442	1.858742	-0.603759	O	4.942563	1.596432	0.706246
O	4.727434	1.858738	0.603804	O	-4.942563	1.596422	-0.706264
H	2.353494	-0.924345	2.144186	H	-2.355410	-0.549483	-2.168065
H	-1.364885	-2.823586	1.140638	H	1.246973	-2.806698	-1.212373
C	-1.436240	-1.342204	-2.603764	C	1.676903	-1.381031	2.453138
H	-2.353493	-0.924276	-2.144208	H	2.355419	-0.549494	2.168065
H	-1.072539	-0.624810	-3.348166	H	1.343290	-1.144072	3.470962
H	-1.752234	-2.242825	-3.136601	H	2.274474	-2.299375	2.501378
C	0.818190	-2.445741	-2.006115	C	-0.637877	-2.420722	2.031560
H	1.512111	-1.837212	-2.599999	H	-1.289859	-1.834475	2.693186
H	1.364886	-2.823551	-1.140707	H	-1.246975	-2.806693	1.212378
H	0.505834	-3.283457	-2.635318	H	-0.243588	-3.257403	2.619480
C	-0.356103	-1.625988	-1.609960	C	0.488352	-1.564049	1.552904
C	0.356103	-1.626036	1.609927	C	-0.488348	-1.564046	-1.552901
N	0.525346	-1.048119	0.464058	N	-0.556345	-0.941222	-0.424980
N	-0.525343	-1.048103	-0.464074	N	0.556349	-0.941224	0.424985
C	-1.296149	1.767562	-0.951742	C	1.525617	1.801836	0.887016
O	-0.872355	2.780261	-1.329042	O	1.165133	2.839137	1.251883
O	0.872349	2.780231	1.329093	O	-1.165134	2.839146	-1.251865
C	1.296144	1.767538	0.951779	C	-1.525616	1.801840	-0.887006
Cr	2.211217	0.211535	0.273217	Cr	-2.321011	0.206887	-0.217859
Cr	-2.211217	0.211543	-0.273211	Cr	2.321013	0.206887	0.217859

Table S62. Optimized coordinates for the $(Me_2C{=}N)_2Cr_2(CO)_8$ structure **8T-8.**

	M06-L				B3PW91-D3		
	x	y	z		x	y	z
C	3.150365	-0.414081	-0.139951	C	2.993195	-0.562426	-0.450173
C	1.506258	0.096250	2.117181	C	2.023946	0.005139	1.869972
C	-3.150276	-0.414309	0.139808	C	-2.899167	-0.569251	0.826593
C	-1.506275	0.096005	-2.117149	C	-2.018337	0.033853	-1.981659
O	4.282589	-0.412249	-0.418581	O	4.081057	-0.657001	-0.852083
O	1.615475	0.422301	3.229355	O	2.525670	0.325544	2.864812
O	-4.282534	-0.412453	0.418302	O	-3.831800	-0.737886	1.498253
O	-1.615608	0.422023	-3.229322	O	-2.449334	0.266250	-3.033443
C	-1.998910	2.113846	2.172965	C	-2.243378	2.447826	1.822155
C	0.192645	3.329406	1.689031	C	0.108461	3.422675	1.640780
H	-0.178567	4.309846	1.366522	H	-0.118479	4.410245	1.216539
H	0.295878	3.375364	2.776800	H	0.026412	3.521229	2.729279
H	-2.426349	3.089945	2.420090	H	-2.540430	3.484998	2.015497
H	-1.712390	1.640238	3.120955	H	-2.193236	1.935715	2.792757
C	1.516900	-2.272110	0.916011	C	1.614082	-2.331696	0.668677
C	-1.516645	-2.272315	-0.915934	C	-1.898121	-2.166764	-0.756655
O	1.626343	-3.363270	1.302910	O	1.848816	-3.437033	0.937556
O	-1.626026	-3.363494	-1.302804	O	-2.266111	-3.216612	-1.079511
H	-2.754789	1.487185	1.698152	H	-3.006043	1.959526	1.213406
H	1.176531	3.165909	1.245855	H	1.134031	3.152518	1.381782
C	1.998692	2.114107	-2.172941	C	2.248901	2.175141	-1.920422
H	2.754640	1.487528	-1.698129	H	3.009845	1.757892	-1.259681
H	1.712230	1.640475	-3.120937	H	2.166660	1.522112	-2.800196
H	2.426022	3.090257	-2.420053	H	2.568757	3.162133	-2.271988
C	-0.193030	3.329373	-1.689037	C	-0.002591	3.351136	-1.748760
H	-0.296348	3.375213	-2.776804	H	0.066642	3.387003	-2.842155
H	-1.176866	3.165815	-1.245776	H	-1.044217	3.194600	-1.462354
H	0.178110	4.309883	-1.366660	H	0.324966	4.333296	-1.381345
C	0.787265	2.265275	-1.322104	C	0.918177	2.278981	-1.241756
C	-0.787508	2.265168	1.322121	C	-0.897379	2.415454	1.166221
N	-0.617226	1.426333	0.346928	N	-0.665654	1.516189	0.266772
N	0.617094	1.426424	-0.346908	N	0.618773	1.433726	-0.306162
O	1.109298	-1.528866	-2.599819	O	0.750315	-1.476736	-2.630118
C	1.071667	-1.127710	-1.502804	C	0.760601	-1.111291	-1.521783
C	-1.071598	-1.127868	1.502842	C	-0.415765	-1.019824	1.475983
O	-1.109188	-1.529070	2.599839	O	-0.580549	-1.417251	2.570479
Cr	1.322490	-0.497247	0.291658	Cr	1.273752	-0.566714	0.231926
Cr	-1.322376	-0.497442	-0.291634	Cr	-1.375710	-0.428227	-0.246860

Table S63. Optimized coordinates for the $(Me_2C{=}N)_2Cr_2(CO)_8$ structure **8T-9.**

	M06-L				B3PW91-D3		
	x	y	z		x	y	z
C	-2.768344	-0.908159	0.826962	C	-2.805214	-0.702183	0.789791
C	-1.477251	1.344993	0.903296	C	-1.346772	1.430926	0.936816
C	2.449127	1.011537	-0.848522	C	2.478516	0.868791	-0.937640
C	0.109508	0.589252	-1.793697	C	0.030034	0.623047	-1.734441
O	-3.620269	-1.313201	1.512328	O	-3.715832	-1.057369	1.417811
O	-1.885001	2.214398	1.577512	O	-1.700466	2.319798	1.607437
O	3.450588	0.886743	-1.428782	O	3.430567	0.768348	-1.590905
O	0.104455	0.535966	-2.964589	O	0.048008	0.686031	-2.903659
C	-0.428579	-0.788985	2.804075	C	-0.474623	-0.797827	2.804553
C	-0.412277	-2.949988	1.478651	C	-0.693844	-2.920922	1.444451
H	0.422732	-3.440597	1.998340	H	0.090631	-3.480680	1.975073
H	-1.325892	-3.206436	2.021074	H	-1.632587	-3.084631	1.982688
H	0.285620	-1.218348	3.522208	H	0.190408	-1.337816	3.496179
H	-1.431821	-0.979469	3.195231	H	-1.491434	-0.883175	3.199980
C	-2.617340	0.626311	-1.346082	C	-2.556827	0.881080	-1.244852
C	0.689889	2.989391	-0.489817	C	0.998617	2.890197	-0.421492
O	-3.378332	1.146925	-2.052086	O	-3.303932	1.486599	-1.888848
O	0.650509	4.115441	-0.766264	O	1.087812	4.010582	-0.695027
H	-0.259746	0.286403	2.768651	H	-0.179267	0.250890	2.802358
H	-0.458484	-3.358245	0.467628	H	-0.773173	-3.329446	0.435609
C	3.070521	-2.970108	-1.124594	C	2.734954	-3.310143	-1.187008
H	2.368273	-3.318294	-1.882225	H	1.976736	-3.553666	-1.935011
H	3.882371	-2.421846	-1.616011	H	3.610525	-2.879964	-1.690747
H	3.527978	-3.829430	-0.622733	H	3.068942	-4.229238	-0.688369
C	3.194383	-1.617101	1.044281	C	3.095603	-1.962709	0.963195
H	4.230850	-1.427665	0.753092	H	4.076192	-1.657770	0.578635
H	2.778019	-0.716967	1.504235	H	2.688773	-1.160325	1.581574
H	3.217478	-2.406934	1.804939	H	3.265236	-2.846234	1.592928
C	2.392956	-2.080800	-0.135014	C	2.192070	-2.333456	-0.182276
C	-0.215025	-1.463590	1.483323	C	-0.354727	-1.450601	1.450095
N	0.569796	-0.873778	0.552428	N	0.501450	-0.940681	0.531817
N	1.164325	-1.778856	-0.385359	N	1.001185	-1.899757	-0.398188
O	-1.623181	-2.585832	-2.188987	O	-1.926729	-2.345810	-2.201023
C	-1.481865	-1.720253	-1.430562	C	-1.657601	-1.529094	-1.429179
C	1.280458	1.866533	1.723796	C	1.521252	1.732854	1.727214
O	1.612804	2.370927	2.717490	O	1.947807	2.212680	2.691733
Cr	0.772229	1.147830	0.010397	Cr	0.895509	1.059911	0.052743
Cr	-1.379970	-0.228615	-0.215125	Cr	-1.367007	-0.105391	-0.204485

Table S64. Optimized coordinates for the $(Me_2C{=}N)_2Cr_2(CO)_8$ structure **8T-10.**

	M06-L				B3PW91-D3		
	x	y	z		x	y	z
C	-3.211752	-1.237088	0.443428	C	-3.244853	-1.284814	0.357063
C	-1.984627	0.762337	1.539106	C	-2.175920	0.676380	1.611138
C	3.295343	-0.899090	-0.752024	C	3.313142	-0.801875	-0.882867
C	1.661192	0.954843	-1.822750	C	1.990841	1.392298	-1.590125
O	-3.939066	-2.041231	0.876688	O	-3.970214	-2.119256	0.720103
O	-1.831828	1.044152	2.665986	O	-2.140136	0.938723	2.746405
O	4.003070	-1.721321	-1.190904	O	4.022537	-1.532948	-1.450315
O	1.354389	1.190879	-2.920537	O	1.927199	1.986143	-2.583709
C	1.628242	-1.578076	2.616625	C	1.737429	-2.064485	2.251193
C	-0.610360	-2.568147	1.873535	C	-0.551110	-2.869262	1.460507
H	-0.302371	-3.429731	2.470367	H	-0.211721	-3.802561	1.920734
H	-1.348324	-2.013005	2.466272	H	-1.275255	-2.408790	2.145779
H	1.921788	-2.563996	2.987516	H	2.186107	-3.063840	2.182005
H	1.242118	-1.012998	3.473797	H	1.350879	-1.952921	3.272868
C	-3.772329	1.090143	-0.394093	C	-3.853329	1.038866	-0.358544
C	3.827698	1.102338	0.796379	C	3.844515	0.830860	1.019713
O	-4.792344	1.646632	-0.517556	O	-4.886104	1.560241	-0.497132
O	4.828468	1.503584	1.233518	O	4.838508	1.118167	1.545038
H	2.509066	-1.055700	2.231347	H	2.511095	-1.311478	2.076818
H	-1.113083	-2.914218	0.968495	H	-1.074469	-3.092985	0.528998
C	-1.460108	-1.397472	-2.438322	C	-1.583185	-1.036612	-2.539789
H	-2.044784	-0.481312	-2.219377	H	-2.200512	-0.187448	-2.173059
H	-1.100934	-1.262670	-3.463409	H	-1.257328	-0.728417	-3.541028
H	-2.146049	-2.250761	-2.436587	H	-2.233544	-1.911773	-2.655081
C	0.782362	-2.538973	-1.901335	C	0.684693	-2.214570	-2.277955
H	1.429948	-2.055752	-2.643630	H	1.289189	-1.618996	-2.974798
H	1.403922	-2.834688	-1.054899	H	1.351260	-2.637121	-1.524875
H	0.366321	-3.431658	-2.378504	H	0.229223	-3.025569	-2.859213
C	-0.313364	-1.617967	-1.502242	C	-0.390865	-1.363428	-1.685330
C	0.560448	-1.691880	1.584542	C	0.610809	-1.940894	1.274408
N	0.692560	-0.979049	0.507800	N	0.693337	-1.035241	0.352204
N	-0.395805	-0.958679	-0.391735	N	-0.425452	-0.872708	-0.491951
C	-1.385778	1.844868	-0.836110	C	-1.499503	1.909912	-0.595662
O	-1.064916	2.902648	-1.195271	O	-1.200115	3.005345	-0.824187
O	0.743290	2.745320	1.478879	O	0.854544	2.506946	1.833381
C	1.236660	1.870684	0.896406	C	1.322883	1.706766	1.141976
Cr	2.203933	0.467962	-0.029245	Cr	2.251251	0.435856	0.046984
Cr	-2.154982	0.203962	-0.190719	Cr	-2.223882	0.210636	-0.149149

Table S65. Optimized coordinates for the $(Me_2C{=}N)_2Cr_2(CO)_8$ structure **8T-11.**

	M06-L				B3PW91-D3		
	x	y	z		x	y	z
C	-0.725420	2.615336	-1.207144	C	2.006770	-0.310300	-1.611255
C	-1.832152	1.890541	1.434244	C	2.721657	-1.782465	0.784113
C	2.139035	0.903136	-1.558508	C	-0.107573	1.888575	-1.380867
C	2.865676	-1.745981	-0.646786	C	-2.479854	2.120495	-0.537142
O	-0.742801	3.449159	-2.015864	O	2.460365	0.032410	-2.619117
O	-2.606390	2.177390	2.247560	O	3.584919	-2.379528	1.283453
O	2.542186	1.763215	-2.222003	O	0.546251	2.688015	-1.916774
O	3.732514	-2.506231	-0.773138	O	-3.228302	3.002392	-0.537792
C	-1.671647	-2.743228	1.957672	C	-2.907097	-2.698366	1.890009
C	-0.139984	-3.682427	0.139070	C	-4.552876	-0.984235	0.921096
H	0.790063	-3.483463	-0.398961	H	-4.592840	0.023259	0.499882
H	-0.041734	-4.608209	0.715586	H	-5.073946	-1.673790	0.241669
H	-1.359089	-3.331893	2.826703	H	-3.597996	-3.478192	1.545597
H	-2.461100	-3.312946	1.454366	H	-3.137335	-2.515348	2.949046
C	0.839354	2.279532	0.886327	C	1.197980	-2.572914	-0.940559
C	-0.500949	-0.194985	-1.228876	C	-0.090865	1.334062	1.124352
O	1.646586	2.970992	1.355919	O	1.234587	-3.573897	-1.525932
O	0.079523	-1.066744	-1.929749	O	-0.503253	1.849993	2.144336
H	-2.071127	-1.798344	2.330808	H	-1.876562	-3.049588	1.820033
H	-0.930066	-3.855222	-0.602029	H	-5.096252	-1.008319	1.874085
C	-4.182094	-0.285173	-0.581970	C	3.684711	1.478608	0.984541
H	-4.673793	-1.150545	-0.121521	H	4.305289	1.797287	1.831070
H	-4.040859	0.489783	0.173593	H	3.832249	0.413734	0.798845
H	-4.873870	0.089047	-1.346071	H	4.032588	2.039967	0.106140
C	-2.887934	-1.787737	-2.205350	C	2.010329	3.207680	1.769442
H	-3.358319	-2.683573	-1.780784	H	2.790757	3.880727	1.398109
H	-3.505311	-1.503593	-3.065838	H	1.023295	3.598330	1.516899
H	-1.885560	-2.034262	-2.554557	H	2.078169	3.188356	2.865034
C	-2.884353	-0.687340	-1.195415	C	2.247595	1.817371	1.242753
C	-0.508644	-2.543494	1.034524	C	-3.137436	-1.434155	1.114837
N	-1.810305	-0.048822	-0.841330	N	1.318724	0.955496	0.980873
N	0.157510	-1.443008	0.962263	N	-2.188460	-0.732951	0.591014
O	-0.195781	-0.186726	2.891795	O	-0.105164	-1.535020	1.427096
C	-0.225116	-0.212496	1.677993	C	-0.818799	-0.876448	0.630632
C	2.583538	0.090022	1.003901	C	-1.404863	-0.173481	-2.192468
O	3.323319	0.407728	1.840076	O	-1.438900	-0.681199	-3.229417
Cr	-0.581933	1.236861	0.130334	Cr	1.255314	-0.951492	0.007142
Cr	1.437400	-0.479298	-0.409196	Cr	-1.222870	0.697903	-0.484365

Table S66. Optimized coordinates for the $(Me_2C{=}N)_2Cr_2(CO)_8$ structure **8S-12.**

	M06-L				B3PW91-D3		
	x	y	z		x	y	z
C	-1.524975	-0.602564	1.515969	C	-2.614526	-0.398825	1.808798
C	-4.005827	0.491047	0.986196	C	-4.695660	0.351365	0.315228
C	4.069291	-0.428045	1.375849	C	4.860903	-0.776225	0.368029
C	3.272979	1.467627	-0.796641	C	3.647359	1.713361	-0.426897
O	-0.890626	-0.851503	2.451577	O	-2.429460	-0.529195	2.941782
O	-4.861865	0.971141	1.605064	O	-5.759504	0.740175	0.550303
O	4.795031	-0.675481	2.253467	O	5.860479	-1.317975	0.607386
O	3.476887	2.473001	-1.356982	O	3.836547	2.827019	-0.701180
C	0.283195	-3.314397	0.207549	C	0.736326	-3.189043	0.881850
C	-0.438539	-1.796089	-1.696874	C	-0.334270	-1.684463	-0.908380
H	-0.799269	-2.668074	-2.251274	H	-0.714052	-2.579158	-1.413186
H	-1.381115	-1.437644	-1.168198	H	-1.193908	-1.335928	-0.238450
H	0.324960	-4.235177	-0.384710	H	0.613713	-4.098659	0.279356
H	-0.735669	-3.258092	0.619778	H	-0.140929	-3.114796	1.538997
C	-3.391414	-1.963032	0.050662	C	-3.543486	-2.010213	-0.165877
C	1.886909	0.946189	1.259419	C	3.092382	0.522288	1.813924
O	-3.877320	-3.015693	0.120905	O	-3.912758	-3.105644	-0.239785
O	1.392704	1.698998	1.995256	O	3.096236	0.898263	2.906876
H	0.996326	-3.378953	1.029512	H	1.636036	-3.279025	1.494756
H	-0.115306	-0.999126	-2.367059	H	-0.115152	-0.889512	-1.622506
C	-1.592014	3.663127	0.476217	C	-2.309487	3.801902	0.203739
H	-1.737215	4.519601	-0.190046	H	-2.135527	4.472991	-0.646990
H	-2.532087	3.428982	0.977823	H	-3.378435	3.591997	0.286310
H	-0.848959	3.953140	1.226981	H	-1.970178	4.321162	1.109123
C	0.147977	2.698233	-1.154730	C	-0.012898	2.665731	-0.116532
H	0.930073	3.195162	-0.570545	H	0.389758	3.170761	0.770886
H	0.535656	1.752345	-1.540777	H	0.467436	1.690997	-0.225232
H	-0.097174	3.358747	-1.993441	H	0.225201	3.291405	-0.986308
C	-1.074002	2.478640	-0.302311	C	-1.516690	2.520374	0.018996
C	0.554812	-2.112628	-0.650136	C	0.824469	-1.973754	-0.012526
N	-1.641400	1.356999	-0.232523	N	-2.089332	1.402688	-0.014170
N	1.517741	-1.301498	-0.404279	N	1.819600	-1.178591	0.022958
O	-4.167710	0.416803	-2.615585	O	-3.362842	0.110860	-3.066343
C	-3.563511	0.191279	-1.652680	C	-3.194658	-0.000794	-1.929324
C	4.218770	-0.837780	-1.147089	C	3.658745	-0.401213	-1.814801
O	5.116391	-1.165621	-1.804832	O	3.998698	-0.571274	-2.907679
Cr	2.820973	-0.167504	-0.001301	Cr	3.203545	-0.060814	-0.007639
Cr	-2.596159	-0.195922	-0.038274	Cr	-2.953797	-0.192172	-0.048882

Table S67. Optimized coordinates for the $(Me_2C{=}N)_2Cr_2(CO)_8$ structure **8S-13.**

M06-L				B3PW91-D3			
	x	y	z		x	y	z
C	2.224549	-1.656566	-1.093558	C	2.388194	-1.583594	-0.686534
C	2.540304	-0.401779	1.405164	C	2.208245	-0.325807	1.803404
C	-0.773133	-2.252266	-1.076556	C	-0.652966	-2.196416	-1.157466
C	-2.540299	-0.401857	-1.405167	C	-2.208245	-0.325806	-1.803403
O	2.808654	-2.313023	-1.859373	O	3.134293	-2.217490	-1.309357
O	3.335908	-0.195570	2.220149	O	2.846597	-0.131953	2.743784
O	-0.611269	-3.200043	-1.728970	O	-0.453735	-3.166480	-1.759624
O	-3.335914	-0.195680	-2.220151	O	-2.846594	-0.131950	-2.743784
C	-1.384029	3.372628	1.733444	C	-2.071456	3.168828	1.902323
C	-3.127415	2.713119	-0.027799	C	-3.457331	2.587717	-0.168450
H	-3.750295	1.848701	-0.265419	H	-3.732984	1.756037	-0.819575
H	-3.738556	3.489388	0.439903	H	-4.339398	2.920112	0.393329
H	-2.073594	3.580008	2.559982	H	-2.846374	3.049714	2.672780
H	-1.272195	4.313468	1.182595	H	-2.138089	4.201214	1.537933
C	0.773190	-2.252243	1.076574	C	0.652966	-2.196416	1.157465
C	0.422637	0.525687	-1.429482	C	0.675665	0.524790	-1.378267
O	0.611345	-3.200016	1.728997	O	0.453736	-3.166480	1.759623
O	-0.059487	0.826629	-2.510322	O	0.403513	0.778123	-2.539606
H	-0.432391	3.059240	2.162629	H	-1.105762	2.982804	2.373698
H	-2.755709	3.121276	-0.976896	H	-3.141368	3.435596	-0.792263
C	3.127344	2.713191	0.027800	C	3.457327	2.587722	0.168452
H	2.755620	3.121311	0.976906	H	3.141366	3.435607	0.792255
H	3.750262	1.848796	0.265402	H	3.732974	1.756046	0.819585
H	3.738451	3.489497	-0.439886	H	4.339398	2.920107	-0.393327
C	1.383949	3.372649	-1.733454	C	2.071456	3.168827	-1.902325
H	1.272107	4.313495	-1.182616	H	2.138086	4.201214	-1.537937
H	2.073499	3.580029	-2.560004	H	2.846376	3.049713	-2.672780
H	0.432310	3.059241	-2.162627	H	1.105763	2.982801	-2.373702
C	1.972945	2.331247	-0.839314	C	2.353769	2.214119	-0.776107
C	-1.972998	2.331207	0.839306	C	-2.353770	2.214117	0.776107
N	1.502924	1.131286	-0.757461	N	1.701688	1.119514	-0.595203
N	-1.502942	1.131261	0.757448	N	-1.701689	1.119513	0.595204
O	0.059471	0.826643	2.510314	O	-0.403512	0.778126	2.539606
C	-0.422642	0.525686	1.429473	C	-0.675664	0.524792	1.378267
C	-2.224507	-1.656607	1.093563	C	-2.388194	-1.583596	0.686534
O	-2.808592	-2.313077	1.859383	O	-3.134294	-2.217491	1.309357
Cr	1.174693	-0.669442	0.076967	Cr	1.129651	-0.609064	0.259436
Cr	-1.174679	-0.669464	-0.076970	Cr	-1.129651	-0.609064	-0.259435

Table S68. Optimized coordinates for the $(Me_2C{=}N)_2Fe_2(CO)_5$ structure **5S-1.**

	M06-L				B3PW91-D3		
	x	y	z		x	y	z
C	-2.133842	-0.447730	-1.441084	C	-2.074669	-0.467858	-1.473665
C	2.019561	-0.054554	-1.698727	C	1.999528	-0.088024	-1.708888
O	-2.930511	-0.222393	-2.257491	O	-2.833697	-0.271596	-2.325667
O	2.735571	-0.643846	-2.401945	O	2.710378	-0.680635	-2.404094
C	1.224399	-2.293067	2.301796	C	1.185027	-2.258146	2.327789
C	3.081857	-0.664546	1.643557	C	3.064309	-0.648945	1.659483
H	3.174552	-0.069660	2.559714	H	3.138928	-0.026810	2.561358
H	3.752090	-1.523253	1.749160	H	3.726232	-1.511977	1.797603
H	1.519029	-2.154634	3.347507	H	1.460270	-2.088199	3.376436
H	1.718138	-3.206490	1.949379	H	1.687783	-3.179025	2.002869
C	-1.973024	-1.336186	1.158987	C	-1.964412	-1.245462	1.165812
C	2.032873	2.047031	-0.014200	C	2.024547	2.011276	-0.032028
O	-2.669979	-1.742725	1.997558	O	-2.669356	-1.584476	2.020781
O	2.822959	2.772066	0.448293	O	2.815862	2.721814	0.435399
H	0.144310	-2.441031	2.244341	H	0.105491	-2.403082	2.249762
H	3.412569	-0.052020	0.803059	H	3.407314	-0.063139	0.804263
C	-3.011112	1.973486	0.849329	C	-2.980208	1.988767	0.867440
H	-3.653916	2.434693	0.090048	H	-3.624620	2.689106	0.321054
H	-3.260993	0.912829	0.913389	H	-3.315531	0.967347	0.678110
H	-3.248841	2.462890	1.800001	H	-3.095684	2.211055	1.937028
C	-1.076166	3.595939	0.529214	C	-1.034831	3.594914	0.481126
H	-1.305374	4.056113	1.496520	H	-1.317635	4.090939	1.418301
H	-0.000567	3.649764	0.348924	H	0.050866	3.624930	0.361954
H	-1.588867	4.196511	-0.231141	H	-1.494869	4.165436	-0.337080
Fe	0.890631	0.823523	-0.616418	Fe	0.881532	0.803437	-0.629426
Fe	-0.907122	-0.729940	-0.165503	Fe	-0.895953	-0.719539	-0.160740
C	-1.566871	2.175495	0.491051	C	-1.539975	2.173133	0.462368
C	1.661932	-1.123706	1.466932	C	1.638502	-1.104389	1.470926
N	-0.804765	1.206959	0.153236	N	-0.794866	1.196610	0.119934
N	0.858653	-0.563731	0.652211	N	0.853902	-0.557011	0.633909
C	-0.359293	-2.273006	-0.908536	C	-0.402799	-2.298121	-0.836126
O	0.040402	-3.262651	-1.367537	O	-0.055305	-3.319857	-1.252625

Table S69. Optimized coordinates for the $(Me_2C{=}N)_2Fe_2(CO)_5$ structure **5T-2.**

	M06-L				B3PW91-D3		
	x	y	z		x	y	z
C	-1.763726	1.312460	-1.171776	C	1.804046	1.291179	1.190342
C	1.086424	-0.000064	-2.301073	C	-1.261268	0.000000	2.313234
O	-2.185659	2.178983	-1.818201	O	2.247152	2.140878	1.836452
O	0.832523	-0.000063	-3.435194	O	-1.105921	0.000000	3.457488
C	-0.914472	-3.117784	1.622012	C	0.943538	-3.081835	-1.631841
C	1.608381	-3.152671	1.279368	C	-1.578062	-3.148054	-1.241074
H	1.759938	-3.407776	2.334013	H	-1.711556	-3.435814	-2.291991
H	1.595890	-4.098423	0.725632	H	-1.569616	-4.074064	-0.650418
H	-0.783452	-3.386299	2.675843	H	0.885164	-3.078983	-2.728628
H	-1.074772	-4.055932	1.077666	H	0.986142	-4.131368	-1.314132
C	-2.295465	0.000111	1.186076	C	2.269024	0.000000	-1.189481
C	3.152134	-0.000126	0.029303	C	-3.137232	-0.000001	-0.146566
O	-3.081080	0.000151	2.043097	O	2.997719	-0.000001	-2.088267
O	4.272217	-0.000163	0.355862	O	-4.220069	-0.000002	-0.560651
H	-1.807769	-2.499841	1.515621	H	1.861288	-2.576497	-1.324959
H	2.450105	-2.550955	0.928413	H	-2.421016	-2.530826	-0.919126
C	-0.914199	3.117879	1.621994	C	0.943536	3.081835	-1.631843
H	-1.074413	4.056039	1.077644	H	0.986140	4.131368	-1.314133
H	-1.807552	2.500016	1.515603	H	1.861285	2.576497	-1.324962
H	-0.783158	3.386385	2.675825	H	0.885161	3.078983	-2.728630
C	1.608656	3.152540	1.279355	C	-1.578065	3.148053	-1.241074
H	1.760233	3.407635	2.333999	H	-1.711559	3.435813	-2.291991
H	2.450328	2.550750	0.928403	H	-2.421018	2.530824	-0.919126
H	1.596249	4.098292	0.725617	H	-1.569620	4.074062	-0.650418
Fe	1.392364	-0.000060	-0.453801	Fe	-1.403238	0.000000	0.435932
Fe	-1.076133	0.000048	-0.142619	Fe	1.095338	0.000001	0.169572
C	0.307124	2.420519	1.092430	C	-0.275466	2.405711	-1.053129
C	0.306913	-2.420536	1.092444	C	-0.275464	-2.405712	-1.053128
N	0.269455	1.291528	0.501626	N	-0.238247	1.290711	-0.440793
N	0.269345	-1.291544	0.501635	N	-0.238246	-1.290711	-0.440793
C	-1.763844	-1.312330	-1.171739	C	1.804046	-1.291177	1.190343
O	-2.185852	-2.178841	-1.818132	O	2.247152	-2.140876	1.836453

Table S70. Optimized coordinates for the $(Me_2C{=}N)_2Fe_2(CO)_5$ structure **5T-3.**

	M06-L				B3PW91-D3		
	x	y	z		x	y	z
C	1.553815	-1.750385	1.311942	C	1.587501	-1.678002	1.299548
C	-0.733931	-2.561910	0.000000	C	-0.652042	-2.594488	0.000000
C	0.654739	2.674262	-1.285156	C	0.647470	2.650330	-1.280141
O	2.244532	-2.112669	2.172063	O	2.296327	-1.994431	2.156397
O	-1.520576	-3.416084	0.000000	O	-1.416983	-3.461511	0.000000
O	0.965229	3.418649	-2.127244	O	0.953490	3.394743	-2.115474
C	-1.773674	1.227681	2.919907	C	-1.835652	1.212647	2.853749
C	-1.345491	-1.271533	3.016073	C	-1.410553	-1.295623	2.960251
H	-1.166869	-1.151341	4.089968	H	-1.333685	-1.162333	4.046531
H	-2.391049	-1.583435	2.905860	H	-2.433304	-1.635364	2.747357
H	-1.251689	1.506583	3.843104	H	-1.336996	1.497285	3.790122
H	-2.808530	1.001573	3.199183	H	-2.878341	0.975630	3.100996
C	1.553815	-1.750385	-1.311942	C	1.587501	-1.678002	-1.299548
C	0.654739	2.674262	1.285156	C	0.647470	2.650330	1.280141
O	2.244532	-2.112669	-2.172063	O	2.296327	-1.994431	-2.156397
O	0.965229	3.418649	2.127244	O	0.953490	3.394743	2.115474
H	-1.763173	2.085152	2.243052	H	-1.811620	2.064069	2.168488
H	-0.705796	-2.069392	2.634860	H	-0.712905	-2.072635	2.641109
C	-1.345491	-1.271533	-3.016073	C	-1.410553	-1.295623	-2.960251
H	-1.166869	-1.151341	-4.089968	H	-1.333685	-1.162333	-4.046531
H	-0.705796	-2.069392	-2.634860	H	-0.712905	-2.072635	-2.641109
H	-2.391049	-1.583435	-2.905860	H	-2.433304	-1.635364	-2.747357
C	-1.773674	1.227681	-2.919907	C	-1.835652	1.212647	-2.853749
H	-2.808530	1.001573	-3.199183	H	-2.878341	0.975630	-3.100996
H	-1.763173	2.085152	-2.243052	H	-1.811620	2.064069	-2.168488
H	-1.251689	1.506583	-3.843104	H	-1.336996	1.497285	-3.790122
Fe	0.150254	1.545963	0.000000	Fe	0.154697	1.519035	0.000000
Fe	0.456192	-1.173773	0.000000	Fe	0.465440	-1.160360	0.000000
C	-1.115560	0.027041	-2.293244	C	-1.148759	0.009563	-2.247768
C	-1.115560	0.027041	2.293244	C	-1.148759	0.009563	2.247768
N	-0.433379	0.118889	-1.217240	N	-0.424208	0.100586	-1.202856
N	-0.433379	0.118889	1.217240	N	-0.424208	0.100586	1.202856

Table S71. Optimized coordinates for the $(Me_2C{=}N)_2Fe_2(CO)_5$ structure **5T-4.**

	M06-L				B3PW91-D3		
	x	y	z		x	y	z
C	-2.013724	2.200524	-0.004556	C	-2.176570	1.989546	-0.221070
C	0.809322	-2.524920	0.331379	C	1.106346	-2.479223	0.152238
C	2.452032	-0.971757	-1.028426	C	2.577568	-0.690657	-1.030478
O	-2.708142	3.114810	-0.220555	O	-2.940144	2.792349	-0.573628
O	0.731980	-3.670531	0.510276	O	1.132005	-3.631390	0.245607
O	3.397604	-1.107157	-1.688702	O	3.526494	-0.685701	-1.689867
C	-2.298609	-2.438350	-0.310707	C	-2.101989	-2.570067	-0.029104
C	-2.611248	-1.049796	-2.425829	C	-2.606731	-1.349962	-2.225030
H	-2.749104	-1.965411	-3.013024	H	-2.714204	-2.313256	-2.742272
H	-3.611512	-0.708540	-2.136409	H	-3.608690	-1.040589	-1.901252
H	-2.126726	-3.404969	-0.800698	H	-1.915422	-3.554485	-0.480280
H	-3.377670	-2.348574	-0.149129	H	-3.169169	-2.512246	0.212835
C	-2.242285	0.078615	1.420255	C	-2.265965	0.076404	1.415373
C	1.726802	-0.490404	1.660405	C	1.694616	-0.451491	1.666297
O	-3.001531	-0.299602	2.221082	O	-2.993529	-0.254685	2.258751
O	2.204188	-0.271908	2.697084	O	2.080582	-0.231170	2.734384
H	-1.787487	-2.445029	0.654319	H	-1.513000	-2.483900	0.887161
H	-2.136778	-0.288091	-3.044796	H	-2.200719	-0.603736	-2.911742
C	1.025277	3.595807	-0.485558	C	0.737118	3.644475	-0.420349
H	1.540686	4.099180	-1.310626	H	1.313044	4.234326	-1.144410
H	-0.048771	3.600054	-0.672115	H	-0.294421	3.549208	-0.762546
H	1.227552	4.185212	0.416142	H	0.745980	4.197700	0.528626
C	3.055243	2.081590	-0.336892	C	2.880596	2.312365	-0.107598
H	3.519089	2.941972	0.154763	H	3.211515	3.203234	0.438700
H	3.396573	1.164159	0.145629	H	3.256734	1.418718	0.394279
H	3.411246	2.076747	-1.374074	H	3.322539	2.361342	-1.112117
Fe	0.995116	-0.765708	0.035039	Fe	1.079821	-0.705897	-0.012387
Fe	-1.035994	0.745886	0.257863	Fe	-1.098845	0.674404	0.204762
C	1.555885	2.200428	-0.314502	C	1.375592	2.291395	-0.222886
C	-1.786759	-1.337331	-1.199353	C	-1.704787	-1.501244	-1.021656
N	0.781281	1.193315	-0.179909	N	0.684216	1.219305	-0.171816
N	-0.669662	-0.705125	-1.030977	N	-0.609588	-0.816731	-0.963200

Table S72. Optimized coordinates for the $(Me_2C{=}N)_2Fe_2(CO)_5$ structure **5S-5.**

	M06-L				B3PW91-D3		
	x	y	z		x	y	z
C	-0.754544	0.870945	1.275395	C	-0.782656	0.822497	1.265529
C	-0.141203	2.768368	-0.937872	C	-0.288396	2.725749	-0.923716
O	-0.879417	1.489454	2.279448	O	-0.944036	1.440607	2.263445
O	-0.043705	3.913664	-1.154873	O	-0.272022	3.875540	-1.132460
C	2.694447	2.403774	0.415597	C	2.565100	2.516214	0.370624
C	3.395691	0.181392	1.452068	C	3.374692	0.348268	1.426019
H	4.388153	0.076540	0.992187	H	4.373458	0.301182	0.971770
H	3.532908	0.785717	2.357305	H	3.472798	0.959577	2.331275
H	3.760292	2.628099	0.539333	H	3.617252	2.797768	0.489351
H	2.148918	2.919997	1.218309	H	1.993808	3.013901	1.166258
C	-2.466948	-1.067271	1.139663	C	-2.385798	-1.157277	1.122373
C	-2.112067	1.220987	-1.037905	C	-2.148535	1.110430	-1.007386
O	-3.561104	-1.097102	1.531565	O	-3.482202	-1.229733	1.498453
O	-3.212955	1.296257	-1.400866	O	-3.256523	1.151725	-1.349988
H	2.342365	2.800569	-0.537894	H	2.190163	2.880318	-0.587362
H	3.027162	-0.806089	1.736793	H	3.061808	-0.658395	1.709800
C	1.688760	-3.329615	-0.315193	C	1.822871	-3.243226	-0.282850
H	0.886541	-3.502855	0.403826	H	0.998211	-3.435760	0.405874
H	1.399203	-3.766214	-1.281077	H	1.594968	-3.711234	-1.249823
H	2.598344	-3.849747	0.014812	H	2.737589	-3.717527	0.096747
C	3.201916	-1.529341	-1.310198	C	3.261090	-1.397307	-1.289122
H	3.271141	-2.242366	-2.140119	H	3.349931	-2.099601	-2.125924
H	3.170702	-0.513357	-1.710905	H	3.199641	-0.379274	-1.680357
H	4.119050	-1.646935	-0.716741	H	4.182422	-1.494871	-0.699539
C	1.987441	-1.866051	-0.484261	C	2.063818	-1.777661	-0.468766
C	2.465864	0.921715	0.526219	C	2.412845	1.032599	0.500412
N	1.462233	0.392832	-0.108406	N	1.432650	0.449344	-0.120714
N	1.183724	-0.992776	0.048381	N	1.220655	-0.934102	0.048360
Fe	-0.419461	1.079102	-0.539206	Fe	-0.461210	1.035535	-0.536656
Fe	-0.783476	-1.079968	0.590720	Fe	-0.713892	-1.104381	0.591978
O	-1.590002	-2.920051	-1.539571	O	-1.475790	-2.927428	-1.548610
C	-1.224801	-2.261407	-0.643621	C	-1.119689	-2.273366	-0.647562

Table S73. Optimized coordinates for the $(Me_2C=N)_2Fe_2(CO)_5$ structure **5T-6.**

	M06-L				B3PW91-D3		
	x	y	z		x	y	z
C	0.652331	-0.291682	1.745459	C	-0.252686	0.732765	1.887760
C	-0.885802	-2.612085	-1.144899	C	2.796554	0.535181	-0.989571
O	0.359634	-1.033773	2.617123	O	0.302092	0.711688	2.920087
O	-1.397550	-3.485897	-1.731099	O	3.758997	0.484713	-1.641935
C	-3.071738	-1.370707	1.278689	C	2.504522	-2.050071	1.439281
C	-3.161248	1.166828	1.559717	C	0.274925	-3.305144	1.346603
H	-4.071702	1.437448	1.010406	H	0.519312	-4.196813	0.752780
H	-3.470232	0.931808	2.581303	H	0.510197	-3.542521	2.389853
H	-4.164967	-1.365037	1.229668	H	2.999021	-3.027582	1.384842
H	-2.798006	-1.621285	2.310063	H	2.468517	-1.761908	2.498628
C	2.785072	1.105814	1.100569	C	-2.873962	0.715052	0.953982
C	1.609505	-1.987862	-0.483817	C	1.327574	2.467716	-0.079095
O	3.862899	1.240985	1.525332	O	-3.963828	0.666789	1.361387
O	2.667991	-2.452343	-0.636314	O	1.388377	3.627469	-0.071107
H	-2.671987	-2.147031	0.623263	H	3.095975	-1.305958	0.899032
H	-2.495908	2.031813	1.572683	H	-0.795212	-3.104714	1.254422
C	-0.557839	3.343162	-1.172582	C	-2.463728	-1.817498	-1.705505
H	0.283985	3.428248	-0.478502	H	-3.064551	-1.258291	-0.984687
H	-0.152431	3.291493	-2.190539	H	-2.497740	-1.287401	-2.667508
H	-1.176438	4.245053	-1.119340	H	-2.898377	-2.811657	-1.868465
C	-2.675290	1.959104	-1.580166	C	-0.164573	-2.908452	-1.981480
H	-2.587835	2.279127	-2.622486	H	-0.365815	-2.834464	-3.056770
H	-3.036035	0.929263	-1.554264	H	0.899386	-2.732827	-1.806224
H	-3.434876	2.604624	-1.120822	H	-0.406529	-3.938936	-1.686757
C	-1.366498	2.125989	-0.884646	C	-1.044301	-1.939182	-1.244680
C	-2.523570	-0.029311	0.936770	C	1.110475	-2.142144	0.900839
N	-1.488033	0.023620	0.154828	N	0.684127	-1.192235	0.129136
N	-0.841809	1.262031	-0.064031	N	-0.660985	-1.167564	-0.277485
Fe	-0.016676	-1.305044	-0.290687	Fe	1.264174	0.706130	-0.095467
Fe	1.151316	0.833844	0.420740	Fe	-1.201553	0.791941	0.365203
O	2.166115	0.893487	-2.367735	O	-1.605924	1.883797	-2.369125
C	1.747071	0.908495	-1.274572	C	-1.430143	1.441142	-1.307850

Table S74. Optimized coordinates for the $(Me_2C{=}N)_2Fe_2(CO)_5$ structure **5T-7.**

	M06-L				B3PW91-D3		
	x	y	z		x	y	z
C	0.561439	0.105094	-1.971440	C	0.887598	0.443163	-1.882512
C	0.254600	1.670270	2.463420	C	-0.573767	1.540878	2.480072
O	0.241410	0.753687	-2.896900	O	0.722214	1.003208	-2.898539
O	0.318006	1.904456	3.602356	O	-0.737830	1.645532	3.618037
C	-2.944220	1.625941	-0.913961	C	-3.249762	0.883444	-1.176174
C	-2.960023	-0.805700	-1.725661	C	-2.580367	-1.450081	-2.015196
H	-3.911236	-1.197098	-1.344438	H	-3.365649	-2.127502	-1.652443
H	-3.168725	-0.363168	-2.703208	H	-2.917551	-1.051018	-2.977474
H	-4.037222	1.629515	-0.969011	H	-4.304025	0.585025	-1.235405
H	-2.573002	2.079857	-1.841329	H	-3.010214	1.411616	-2.108897
C	2.692085	-1.146396	-1.269328	C	2.959320	-0.739068	-0.950254
C	0.302444	2.861665	-0.352403	C	-0.283517	2.931117	-0.351671
O	3.746815	-1.311207	-1.741417	O	4.059871	-0.865704	-1.309533
O	0.443723	3.791850	-1.036554	O	-0.136667	3.871497	-1.001911
H	-2.615403	2.244485	-0.074995	H	-3.115656	1.571044	-0.336253
H	-2.266074	-1.639668	-1.849385	H	-1.662594	-2.025838	-2.162469
C	-0.547530	-3.457967	0.843127	C	0.181986	-3.505185	0.732035
H	0.286828	-3.436375	0.133627	H	1.044445	-3.285271	0.095465
H	-0.128645	-3.549629	1.852275	H	0.523990	-3.526641	1.776015
H	-1.160584	-4.347077	0.665506	H	-0.213632	-4.501071	0.495940
C	-2.687050	-2.163406	1.429492	C	-2.224030	-2.733792	1.187544
H	-2.613288	-2.616055	2.422632	H	-2.088437	-3.179607	2.180108
H	-3.064582	-1.144511	1.529532	H	-2.833279	-1.831898	1.282586
H	-3.423353	-2.755996	0.872696	H	-2.772585	-3.470445	0.584381
C	-1.362898	-2.214487	0.746220	C	-0.881047	-2.462143	0.575891
C	-2.420553	0.235781	-0.804879	C	-2.374498	-0.324716	-1.045501
N	-1.475197	0.027067	0.061889	N	-1.463774	-0.310329	-0.126253
N	-0.836421	-1.230270	0.086210	N	-0.549477	-1.375404	-0.043538
Fe	0.117092	1.278834	0.644349	Fe	-0.262300	1.280149	0.630317
Fe	1.094573	-0.848656	-0.541329	Fe	1.289976	-0.455408	-0.384898
C	1.804873	-0.683021	1.100049	C	1.822648	-0.157597	1.280139
O	2.399334	-0.617926	2.112894	O	2.340036	-0.000488	2.324427

Table S75. Optimized coordinates for the $(Me_2C{=}N)_2Fe_2(CO)_5$ structure **5S-8.**

	M06-L				B3PW91-D3		
	x	y	z		x	y	z
C	2.466963	-0.733095	1.499154	C	2.573259	-0.505680	1.524987
C	-3.787550	0.211263	-0.086361	C	-3.815736	0.235956	0.048441
O	2.991513	-1.030243	2.499976	O	3.142154	-0.625478	2.537374
O	-4.916745	0.229790	0.211341	O	-4.912368	0.246345	0.431110
C	-0.844878	-1.978522	1.407474	C	-0.897408	-2.180081	1.126650
C	-0.973131	0.325358	2.424096	C	-0.970371	0.004700	2.396849
H	-1.766416	-0.012061	3.095620	H	-1.788911	-0.399404	3.001493
H	-0.012823	0.201312	2.952841	H	-0.026951	-0.204066	2.925648
H	-1.729990	-2.370829	1.916445	H	-1.815118	-2.582715	1.569564
H	0.030910	-2.176712	2.047308	H	-0.051345	-2.475745	1.765935
C	2.318529	-1.709080	-0.916295	C	2.609986	-1.703461	-0.724505
C	-2.325833	-1.409080	-1.165130	C	-2.477296	-1.309721	-1.250650
O	2.534076	-2.717221	-1.457499	O	2.980967	-2.715815	-1.155614
O	-2.513060	-2.436531	-1.682290	O	-2.736543	-2.251211	-1.873367
H	-0.697409	-2.530944	0.476148	H	-0.739760	-2.632488	0.144284
H	-1.099660	1.398783	2.248620	H	-1.075955	1.093233	2.344142
C	0.896358	3.509406	-0.148054	C	0.893389	3.435213	-0.010169
H	1.889564	3.298497	0.251590	H	1.902779	3.182721	0.323877
H	1.013672	3.841922	-1.186989	H	0.961342	3.869134	-1.017634
H	0.458065	4.342886	0.409605	H	0.477056	4.205183	0.651746
C	-1.393057	2.302107	-0.436766	C	-1.423575	2.276380	-0.344747
H	-1.587733	1.739839	-1.401576	H	-1.656475	1.809789	-1.345217
H	-2.018128	1.830790	0.378699	H	-2.041854	1.787175	0.469530
H	-1.825617	3.293452	-0.575900	H	-1.827750	3.291281	-0.389342
C	0.039915	2.296664	-0.077903	C	0.031157	2.216034	-0.021576
C	-0.954239	-0.500195	1.169473	C	-0.942093	-0.673751	1.048833
N	-0.177689	-0.043428	0.076103	N	-0.236863	-0.101753	-0.015822
N	0.580653	1.135685	0.170933	N	0.546025	1.042903	0.166976
Fe	-2.055343	0.129128	-0.407477	Fe	-2.110912	0.152293	-0.385973
Fe	1.892451	-0.187501	-0.107340	Fe	1.957420	-0.189167	-0.096846
C	3.162919	0.715799	-0.975462	C	2.994849	0.746594	-1.175710
O	4.044720	1.308107	-1.461750	O	3.782372	1.367846	-1.771905

Table S76. Optimized coordinates for the $(Me_2C{=}N)_2Cr_2(CO)_7$ structure **7S-1.**

	M06-L				B3PW91-D3		
	x	y	z		x	y	z
C	2.518730	-1.168941	0.725409	C	2.522079	-1.124519	0.672128
C	2.365225	1.490092	0.835796	C	2.363940	1.477391	0.784354
C	-2.284512	-0.347164	-1.331593	C	-2.260952	-0.371012	-1.313080
C	-1.980346	-1.369880	1.294796	C	-1.922242	-1.354952	1.328255
O	3.292078	-1.947419	1.110255	O	3.321608	-1.883603	1.032263
O	3.043822	2.323267	1.279989	O	3.066728	2.298346	1.204878
O	-2.909150	-0.501095	-2.308357	O	-2.874917	-0.525565	-2.289297
O	-2.357725	-2.168724	2.050264	O	-2.277283	-2.148530	2.092401
C	0.864420	3.580191	-1.080411	C	0.798220	3.582168	-1.092118
C	-1.664256	3.385869	-0.819459	C	-1.721594	3.369462	-0.753266
H	-2.025021	3.725871	0.157258	H	-2.103872	3.605716	0.246969
H	-1.623224	4.255408	-1.479841	H	-1.678141	4.298571	-1.329735
H	0.971891	4.430431	-0.396306	H	0.862636	4.478047	-0.459748
H	0.700727	4.000875	-2.078001	H	0.619475	3.927511	-2.118726
C	0.587018	-0.009181	1.983890	C	0.672541	0.016319	1.979617
C	-2.726884	0.950365	0.754950	C	-2.732048	0.880781	0.749818
O	0.423330	-0.073751	3.135298	O	0.539160	-0.030994	3.131748
O	-3.678956	1.517725	1.108323	O	-3.700550	1.411588	1.100195
H	1.795284	3.012297	-1.083730	H	1.749159	3.050383	-1.058117
H	-2.396653	2.673251	-1.209306	H	-2.431753	2.685540	-1.229876
C	1.400743	-3.242130	-1.340583	C	1.408199	-3.216806	-1.341828
H	1.264765	-3.801217	-2.271273	H	1.244614	-3.868002	-2.207722
H	2.178170	-2.489389	-1.484509	H	2.141270	-2.450065	-1.601266
H	1.750545	-3.956111	-0.586091	H	1.827113	-3.834803	-0.537086
C	-1.120852	-3.472427	-1.124899	C	-1.109181	-3.488610	-1.090255
H	-0.978584	-4.466117	-0.686149	H	-0.921315	-4.486370	-0.673412
H	-2.014667	-3.013449	-0.699819	H	-2.004420	-3.065904	-0.631262
H	-1.282812	-3.620938	-2.198760	H	-1.292182	-3.615540	-2.165553
C	0.100007	-2.623027	-0.908055	C	0.100839	-2.608909	-0.890271
C	-0.311946	2.736347	-0.682563	C	-0.357291	2.722843	-0.648750
N	0.032938	-1.455580	-0.398272	N	0.024783	-1.442304	-0.384149
N	-0.186516	1.538548	-0.254002	N	-0.211709	1.527720	-0.223280
C	2.090858	0.270920	-1.548603	C	1.998328	0.291048	-1.564802
O	2.622684	0.379683	-2.573673	O	2.486177	0.409783	-2.605862
Cr	1.247236	0.117663	0.171907	Cr	1.233649	0.127752	0.167782
Cr	-1.238206	-0.061637	0.145326	Cr	-1.223504	-0.070353	0.155889

Table S77. Optimized coordinates for the $(Me_2C{=}N)_2Cr_2(CO)_7$ structure **7T-2.**

	M06-L				B3PW91-D3		
	x	y	z		x	y	z
C	2.269713	-1.378443	1.066335	C	-1.838192	1.731935	1.181515
C	2.269718	1.378452	1.066315	C	-2.506280	-0.935296	1.081327
C	-2.398729	-0.000005	-1.347463	C	2.524464	-0.334166	-1.124044
C	-2.280879	-1.289979	1.248589	C	2.506255	0.935271	1.081401
O	2.888406	-2.237930	1.549827	O	-2.152549	2.680744	1.758872
O	2.888413	2.237945	1.549793	O	-3.302332	-1.620926	1.565263
O	-2.999166	-0.000006	-2.339226	O	3.327818	-0.379286	-1.960437
O	-2.896075	-2.020754	1.904736	O	3.302296	1.620889	1.565371
C	1.210531	3.213113	-1.535819	C	-1.721286	-2.916667	-1.660001
C	-1.330629	3.147925	-1.629818	C	0.783459	-3.342291	-1.546306
H	-1.296051	4.221990	-1.419649	H	0.810564	-4.163339	-0.816624
H	-1.469011	3.041281	-2.712387	H	0.653638	-3.793094	-2.537459
H	1.293896	4.113673	-0.915977	H	-1.856632	-3.965014	-1.364673
H	1.199123	3.547592	-2.578754	H	-1.782388	-2.881585	-2.756136
C	0.339099	0.000011	2.004711	C	-0.000027	-0.000018	1.906306
C	-2.280881	1.289980	1.248581	C	1.838165	-1.731960	1.181519
O	0.136740	0.000019	3.158674	O	-0.000034	-0.000030	3.074775
O	-2.896074	2.020758	1.904727	O	2.152511	-2.680778	1.758866
H	2.094588	2.596738	-1.364213	H	-2.532879	-2.317822	-1.244348
H	-2.198992	2.712127	-1.128440	H	1.740099	-2.815930	-1.503791
C	1.210533	-3.213121	-1.535807	C	-0.783425	3.342327	-1.546246
H	1.199139	-3.547569	-2.578752	H	-0.653587	3.793149	-2.537388
H	2.094594	-2.596760	-1.364169	H	-1.740068	2.815969	-1.503755
H	1.293879	-4.113702	-0.915993	H	-0.810538	4.163362	-0.816549
C	-1.330627	-3.147925	-1.629817	C	1.721320	2.916696	-1.659912
H	-1.296046	-4.221995	-1.419678	H	1.856658	3.965042	-1.364579
H	-2.198987	-2.712141	-1.128421	H	2.532905	2.317850	-1.244243
H	-1.469018	-3.041249	-2.712382	H	1.782444	2.881615	-2.756046
C	-0.054886	-2.474048	-1.199466	C	0.366517	2.420161	-1.216803
C	-0.054890	2.474041	-1.199476	C	-0.366492	-2.420136	-1.216862
N	-0.053837	-1.355491	-0.590288	N	0.195400	1.318910	-0.598455
N	-0.053840	1.355481	-0.590304	N	-0.195388	-1.318898	-0.598488
C	2.421096	-0.000010	-1.224799	C	-2.524436	0.334191	-1.124092
O	3.146709	-0.000015	-2.129439	O	-3.327768	0.379333	-1.960505
Cr	1.238258	0.000000	0.293485	Cr	-1.238116	0.188932	0.216235
Cr	-1.262425	-0.000001	0.194758	Cr	1.238112	-0.188938	0.216254

Table S78. Optimized coordinates for the $(Me_2C{=}N)_2Cr_2(CO)_7$ structure **7S-3.**

	M06-L				B3PW91-D3		
	x	y	z		x	y	z
C	-0.780842	-0.505877	1.813764	C	-0.816965	-0.439070	1.818119
C	-1.771363	1.057685	-1.552769	C	-1.698215	1.043310	-1.559562
C	2.135440	-0.048008	1.572517	C	2.122815	-0.062243	1.567194
O	-0.676298	-0.946183	2.886664	O	-0.737377	-0.843474	2.902363
O	-2.136015	1.549969	-2.538106	O	-2.041387	1.536323	-2.547264
O	2.545483	0.209210	2.628118	O	2.542602	0.218365	2.607263
C	-1.302165	-3.189731	0.092881	C	-1.396123	-3.162791	0.147172
C	-1.908769	-2.168056	-2.176273	C	-1.918376	-2.156563	-2.156403
H	-1.733820	-3.126343	-2.679664	H	-1.738804	-3.133885	-2.625343
H	-2.974796	-2.124071	-1.930853	H	-2.991439	-2.082212	-1.947963
H	-1.013647	-4.151930	-0.347472	H	-1.110858	-4.129387	-0.290611
H	-2.361229	-3.261851	0.359232	H	-2.463890	-3.208745	0.386969
C	-2.905375	-0.476902	0.342696	C	-2.889990	-0.396325	0.315104
C	2.764346	-0.210097	-1.293385	C	2.700360	-0.291849	-1.314238
O	-3.964883	-0.942171	0.501207	O	-3.967010	-0.813009	0.459395
O	3.587103	-0.082909	-2.108135	O	3.490651	-0.192382	-2.156965
H	-0.704683	-3.028122	0.991832	H	-0.816123	-3.005726	1.058731
H	-1.657838	-1.360326	-2.864702	H	-1.621824	-1.370274	-2.853563
C	2.464296	2.931956	-0.368948	C	2.537032	2.865289	-0.377389
H	2.645869	3.820082	0.245057	H	2.724820	3.759111	0.230330
H	3.104293	2.117067	-0.023740	H	3.166956	2.047204	-0.021344
H	2.750676	3.191497	-1.395058	H	2.820966	3.108736	-1.410162
C	0.047467	3.696204	-0.442643	C	0.135414	3.681395	-0.456118
H	0.403845	4.410228	-1.191774	H	0.554198	4.417538	-1.151974
H	-0.957883	3.367386	-0.708003	H	-0.857945	3.388624	-0.798740
H	-0.011736	4.235417	0.509723	H	0.030397	4.173100	0.519736
C	1.009289	2.546760	-0.324652	C	1.070038	2.501681	-0.330826
C	-1.066133	-2.111000	-0.930867	C	-1.114962	-2.086428	-0.877997
N	0.647382	1.329721	-0.200217	N	0.682719	1.295432	-0.196300
N	-0.024454	-1.341650	-0.874485	N	-0.043121	-1.359840	-0.812081
O	2.811053	-3.125138	0.383697	O	2.752849	-3.143475	0.430075
C	2.241656	-2.127658	0.194110	C	2.198435	-2.145150	0.230058
C	-1.797255	1.739206	1.103132	C	-1.755849	1.765955	1.053623
O	-2.177242	2.620717	1.764454	O	-2.135877	2.656061	1.695570
Cr	-1.211950	0.274141	0.117783	Cr	-1.185598	0.285356	0.110227
Cr	1.355837	-0.485824	-0.107876	Cr	1.333476	-0.517179	-0.089948

Table S79. Optimized coordinates for the $(Me_2C{=}N)_2Cr_2(CO)_7$ structure **7S-4.**

	M06-L				B3PW91-D3		
	x	y	z		x	y	z
C	3.391408	-0.194976	0.000026	C	-3.342565	-0.245438	-0.010261
C	2.007392	1.221557	-1.458319	C	-2.013034	1.151391	1.470607
C	-1.962847	-0.108128	1.469033	C	1.914532	-0.093291	-1.490605
C	-1.962895	-0.107609	-1.468985	C	1.938436	-0.060345	1.472878
O	4.544208	-0.385628	0.000007	O	-4.486393	-0.463496	-0.013915
O	2.226769	1.837991	-2.422683	O	-2.247536	1.722531	2.453639
O	-2.536445	0.170039	2.445164	O	2.449102	0.162455	-2.488662
O	-2.536549	0.170913	-2.444985	O	2.490491	0.222973	2.453426
C	2.329191	-2.758498	1.279045	C	-2.258924	-2.759607	-1.287979
C	2.329240	-2.758064	-1.279881	C	-2.268169	-2.756714	1.278900
H	3.422794	-2.724374	-1.293557	H	-3.363465	-2.756281	1.295061
H	2.036307	-3.813064	-1.348421	H	-1.932815	-3.802138	1.331037
H	1.937078	-2.233622	2.151992	H	-1.873329	-2.225822	-2.160576
H	3.422744	-2.724806	1.292779	H	-3.354094	-2.760609	-1.311329
C	2.007355	1.221067	1.458815	C	-2.013283	1.203173	-1.442155
C	-2.040760	-2.122230	-0.000327	C	2.046103	-2.053680	0.011428
O	2.226707	1.837181	2.423390	O	-2.246398	1.811767	-2.402306
O	-2.784132	-3.016946	-0.000466	O	2.785394	-2.945680	0.015399
H	2.036262	-3.813523	1.347212	H	-1.921825	-3.804664	-1.336214
H	1.937167	-2.232886	-2.152664	H	-1.887768	-2.221842	2.153069
C	0.409146	3.614940	0.000624	C	-0.461512	3.621932	-0.022578
H	0.238556	4.245702	0.880191	H	-0.407503	4.122178	-0.998634
H	1.447438	3.281876	0.000560	H	-1.487684	3.291110	0.142825
H	0.238544	4.246007	-0.878721	H	-0.191071	4.363930	0.738580
C	-2.006561	2.850612	0.000472	C	1.968578	2.899054	0.030230
H	-2.230030	3.466061	-0.878500	H	2.195138	3.344137	1.008593
H	-2.665880	1.980565	0.000497	H	2.648062	2.061575	-0.134919
H	-2.229986	3.466094	0.879429	H	2.150140	3.672432	-0.726477
C	-0.554805	2.463118	0.000431	C	0.519925	2.476790	0.002457
C	1.784036	-2.179306	-0.000330	C	-1.746651	-2.142962	-0.003377
N	-0.186047	1.238223	0.000230	N	0.172881	1.247490	0.001280
N	0.633054	-1.528644	-0.000241	N	-0.587675	-1.498743	0.000201
C	-4.757375	0.370084	0.000014	C	4.804260	0.334343	-0.041789
O	-5.403356	-0.574677	-0.000931	O	5.369643	-0.655935	0.003490
Cr	-0.918371	-0.598818	-0.000081	Cr	0.917979	-0.558714	0.002695
Cr	1.566170	0.161423	0.000065	Cr	-1.547596	0.149962	-0.002131

Table S80. Optimized coordinates for the $(Me_2C{=}N)_2Cr_2(CO)_7$ structure **7S-5.**

	M06-L				B3PW91-D3		
	x	y	z		x	y	z
C	-2.469827	0.811214	-0.746115	C	-2.367518	0.704100	-0.955698
C	-0.721025	-0.673687	-1.642783	C	-0.654181	-1.006431	-1.492565
C	2.001943	-1.142626	-1.801386	C	2.046435	-1.081603	-1.729202
C	2.939237	-0.602808	0.498699	C	2.938223	-0.454130	0.501797
O	-3.112794	1.592524	-1.337352	O	-2.927155	1.429351	-1.679963
O	-0.748829	-0.865493	-2.797315	O	-0.698831	-1.439261	-2.576350
O	2.503445	-1.568410	-2.759487	O	2.593158	-1.434958	-2.686918
O	3.989204	-0.740470	0.996155	O	4.009141	-0.494019	0.960576
C	0.931213	3.251580	-1.981353	C	0.963610	3.131695	-2.075727
C	-0.597576	3.569843	0.066060	C	-0.641677	3.535421	-0.093080
H	-0.088116	4.442713	0.489961	H	-0.188054	4.503058	0.155011
H	-1.374549	3.949800	-0.603162	H	-1.480122	3.730589	-0.772482
H	1.324441	4.265720	-1.862414	H	1.403083	4.129793	-1.958809
H	0.126095	3.307538	-2.723071	H	0.174295	3.209290	-2.835133
C	-2.395721	-1.866298	-0.250244	C	-2.471232	-1.844998	-0.094851
C	0.862614	-2.133635	0.399670	C	1.024088	-2.140873	0.517609
O	-3.008624	-2.826382	-0.492826	O	-3.118366	-2.796673	-0.245544
O	0.634029	-3.200296	0.830855	O	0.891770	-3.197885	0.992247
H	1.713178	2.594470	-2.364664	H	1.725202	2.429213	-2.424324
H	-1.066522	3.003864	0.873653	H	-1.026402	3.065039	0.814800
C	0.571117	-0.219844	2.902452	C	0.467590	-0.140457	2.927355
H	-0.265687	-0.857682	2.601111	H	-0.354685	-0.803766	2.639270
H	1.380883	-0.871154	3.244651	H	1.277730	-0.763963	3.319158
H	0.256686	0.391484	3.758339	H	0.121095	0.515019	3.738795
C	2.129240	1.636546	2.126551	C	2.007398	1.724705	2.134735
H	2.953920	1.136837	2.640340	H	2.829150	1.261694	2.688581
H	2.528186	2.144315	1.246932	H	2.421503	2.228445	1.258713
H	1.723678	2.392288	2.814027	H	1.541113	2.475927	2.789453
C	1.048039	0.656476	1.786835	C	0.971481	0.690914	1.779120
C	0.378910	2.739416	-0.695712	C	0.361821	2.667909	-0.786820
N	0.772648	1.575478	-0.312396	N	0.722154	1.516304	-0.355342
N	0.147908	0.917731	0.791326	N	0.098415	0.895223	0.751332
O	-3.379191	0.111805	2.422794	O	-3.488629	0.520255	2.190952
C	-2.577922	-0.075427	1.600871	C	-2.661785	0.172449	1.455899
Cr	1.248304	-0.443665	-0.225231	Cr	1.238108	-0.454835	-0.172677
Cr	-1.384781	-0.351476	0.143894	Cr	-1.412363	-0.349653	0.157827

Table S81. Optimized coordinates for the $(Me_2C=N)_2Cr_2(CO)_7$ structure **7T-6.**

	M06-L				B3PW91-D3		
	x	y	z		x	y	z
C	-1.108582	0.000019	1.836006	C	-0.415531	0.000003	1.982523
C	-1.611303	-0.000009	-1.979513	C	-2.410596	-0.000010	-1.218945
C	3.133133	1.279073	-0.645961	C	2.251536	1.270510	1.276352
O	-1.070840	0.000036	2.998205	O	-0.239878	0.000001	3.138352
O	-1.818419	-0.000013	-3.120178	O	-3.161733	-0.000021	-2.098464
O	3.990149	2.017915	-0.893604	O	2.856796	1.988750	1.945268
C	-0.064099	-3.084034	1.218405	C	1.365838	-3.175295	-1.603054
C	-0.829485	-3.301843	-1.210934	C	-1.173636	-3.223951	-1.564679
H	-0.210273	-4.183210	-1.416862	H	-1.133956	-3.539883	-2.615114
H	-1.800904	-3.668911	-0.865058	H	-1.257121	-4.136006	-0.958229
H	0.500173	-4.022539	1.163553	H	1.500183	-3.104934	-2.691149
H	-1.061664	-3.337812	1.593496	H	1.317044	-4.243122	-1.355345
C	-2.683562	-1.255915	0.160114	C	-2.264642	-1.353338	1.021924
C	3.133124	-1.279091	-0.645962	C	2.416285	-0.000007	-1.312249
O	-3.549503	-2.026196	0.321236	O	-2.902861	-2.192310	1.509510
O	3.990132	-2.017940	-0.893610	O	3.049125	-0.000011	-2.276712
H	0.431979	-2.412167	1.922028	H	2.238795	-2.735219	-1.114678
H	-0.953865	-2.740581	-2.138236	H	-2.065397	-2.616809	-1.403912
C	-0.829472	3.301834	-1.210969	C	1.365838	3.175279	-1.603083
H	-0.210247	4.183188	-1.416917	H	2.238795	2.735212	-1.114697
H	-0.953865	2.740558	-2.138260	H	1.500190	3.104905	-2.691176
H	-1.800883	3.668923	-0.865093	H	1.317041	4.243109	-1.355387
C	-0.064099	3.084049	1.218375	C	-1.173636	3.223925	-1.564728
H	-1.061673	3.337780	1.593477	H	-1.133950	3.539845	-2.615166
H	0.432018	2.412208	1.921997	H	-2.065395	2.616780	-1.403960
H	0.500124	4.022582	1.163513	H	-1.257133	4.135987	-0.958289
C	-0.145855	2.472102	-0.156990	C	0.086766	2.478109	-1.193841
C	-0.145863	-2.472101	-0.156966	C	0.086765	-2.478124	-1.193813
N	0.458835	1.376789	-0.457118	N	0.076662	1.358890	-0.587255
N	0.458829	-1.376792	-0.457109	N	0.076663	-1.358894	-0.587245
O	2.263886	0.000016	2.747452	O	2.856886	-1.988685	1.945253
C	2.091310	0.000007	1.595347	C	2.251583	-1.270475	1.276343
C	-2.683562	1.255914	0.160106	C	-2.264645	1.353345	1.021913
O	-3.549504	2.026193	0.321227	O	-2.902864	2.192320	1.509494
Cr	-1.347276	-0.000001	-0.063578	Cr	-1.228482	0.000002	0.270068
Cr	1.704280	-0.000003	-0.230181	Cr	1.251495	-0.000003	0.195308

Table S82. Optimized coordinates for the $(Me_2C=N)_2Cr_2(CO)_7$ structure **7S-7.**

	M06-L				B3PW91-D3		
	x	y	z		x	y	z
C	1.240456	-2.135295	0.014290	C	1.360730	-2.118627	0.309776
C	2.611680	-0.745734	-1.646325	C	2.487298	-0.963166	-1.636971
C	-1.144470	2.407110	0.845474	C	-1.083321	2.463207	0.503240
C	0.997596	2.086027	-0.506344	C	0.937700	1.914099	-0.901873
O	1.052605	-3.269875	0.206303	O	1.304138	-3.219539	0.668481
O	3.406505	-1.055284	-2.431873	O	3.224897	-1.353287	-2.434896
O	-1.431258	3.285150	1.550993	O	-1.341604	3.461828	1.031092
O	1.812292	2.910297	-0.678092	O	1.679127	2.722894	-1.303188
C	-2.913801	-2.742240	-0.911480	C	-3.143355	-2.768760	-0.438138
C	-3.938944	-0.908233	0.550000	C	-4.027753	-0.670312	0.734778
H	-3.817594	0.141864	0.825760	H	-3.817987	0.392083	0.883232
H	-4.896287	-1.041038	0.034949	H	-4.976849	-0.782356	0.194391
H	-3.535971	-2.696610	-1.812445	H	-3.822491	-2.788648	-1.301787
H	-3.408703	-3.423454	-0.213008	H	-3.637334	-3.315198	0.374260
C	2.812243	0.155587	0.796513	C	2.844805	0.321072	0.571111
C	-1.326366	1.837043	-1.819609	C	-1.408104	1.465967	-1.959081
O	3.678406	0.501476	1.490617	O	3.758899	0.795404	1.100240
O	-1.786854	2.349651	-2.748801	O	-1.916857	1.833864	-2.926695
H	-1.943331	-3.137953	-1.209770	H	-2.219050	-3.266622	-0.732272
H	-3.992930	-1.514510	1.462158	H	-4.162148	-1.155366	1.711059
C	0.885668	-1.272130	3.260640	C	1.144240	-0.687968	3.434516
H	0.384171	-2.210226	3.524353	H	0.678593	-1.569573	3.894514
H	1.774421	-1.503834	2.670822	H	2.008688	-1.008433	2.850013
H	1.190631	-0.799355	4.200493	H	1.481449	-0.037848	4.252121
C	-1.269183	0.100554	3.252923	C	-1.033497	0.640833	3.322334
H	-0.980776	0.819823	4.027941	H	-0.695986	1.503568	3.912152
H	-1.983836	0.579559	2.580162	H	-1.815591	0.966890	2.634307
H	-1.757864	-0.736967	3.762941	H	-1.449100	-0.089343	4.028096
C	-0.054408	-0.376077	2.504466	C	0.135450	0.038991	2.577244
C	-2.810428	-1.374533	-0.312301	C	-2.918503	-1.339430	-0.026425
N	0.160138	-0.034025	1.299341	N	0.259505	0.144610	1.318266
N	-1.809404	-0.592294	-0.502244	N	-1.854348	-0.674990	-0.291129
C	-0.657083	-0.802153	-1.354225	C	-0.715813	-1.052663	-1.113673
O	-0.655015	-1.471910	-2.372963	O	-0.751953	-1.892357	-1.995377
Cr	1.332334	-0.300299	-0.288412	Cr	1.307419	-0.352101	-0.269236
Cr	-0.528096	0.989105	-0.278573	Cr	-0.538283	0.877668	-0.366944

Table S83. Optimized coordinates for the $(Me_2C{=}N)_2Cr_2(CO)_7$ structure **7S-8.**

	M06-L				B3PW91-D3		
	x	y	z		x	y	z
C	-2.922812	-1.091732	-0.963641	C	-2.627703	-1.392377	-0.967754
C	-0.256159	-0.864709	-1.551957	C	-0.250376	-1.121986	-1.613203
C	2.952642	0.530430	-0.756543	C	2.718771	0.691835	-0.957189
C	2.011749	0.526559	1.529791	C	2.206388	0.826776	1.460531
O	-3.919614	-1.144491	-1.571457	O	-3.573686	-1.599852	-1.617986
O	0.614324	-0.579438	-2.326159	O	0.186098	-1.154335	-2.705290
O	3.992248	0.897040	-1.134035	O	3.668183	1.034304	-1.531018
O	2.392897	0.991377	2.533168	O	2.739210	1.367762	2.344227
C	0.904309	2.826173	-2.055266	C	0.791486	3.040246	-1.822159
C	-1.593304	2.973852	-1.525303	C	-1.742598	2.852827	-1.618414
H	-1.802888	3.823453	-0.864111	H	-2.014372	3.811183	-1.154984
H	-1.624100	3.347772	-2.550206	H	-1.703870	3.025252	-2.699669
H	0.885721	3.912838	-2.183803	H	0.571242	4.092076	-2.039046
H	0.825517	2.383552	-3.054630	H	0.982727	2.543815	-2.783067
C	-1.146291	-2.875100	-0.155896	C	-0.815469	-2.908590	0.098662
C	2.296889	-1.763416	0.046640	C	2.381601	-1.510969	0.087143
O	-1.060491	-4.038354	-0.204143	O	-0.676387	-4.062980	0.159454
O	2.889241	-2.764555	0.107344	O	3.056687	-2.455245	0.081375
H	1.856574	2.523845	-1.620657	H	1.692552	2.975529	-1.210790
H	-2.387640	2.235929	-1.381538	H	-2.521869	2.119468	-1.398993
C	-2.742895	1.055700	2.191175	C	-3.081211	0.833980	1.983003
H	-3.180853	0.218639	1.635090	H	-3.367989	-0.064861	1.426170
H	-2.359283	0.675473	3.144752	H	-2.770519	0.540170	2.994721
H	-3.533414	1.774523	2.426332	H	-3.960804	1.480253	2.090214
C	-1.086458	2.994904	1.899338	C	-1.649428	2.953096	1.781512
H	-0.895069	2.935385	2.975197	H	-1.688988	2.971693	2.876929
H	-0.157396	3.264167	1.394374	H	-0.664358	3.295403	1.456981
H	-1.817641	3.799772	1.757729	H	-2.409720	3.662078	1.425943
C	-1.641011	1.698490	1.416646	C	-1.967535	1.570231	1.299495
C	-0.250405	2.389512	-1.225190	C	-0.387529	2.411158	-1.147211
N	-0.054967	1.500911	-0.300720	N	-0.191387	1.514950	-0.229868
N	-1.197456	1.035990	0.394812	N	-1.323239	0.935269	0.374141
C	0.237568	-1.059100	1.393243	C	0.493866	-0.909466	1.569352
O	0.103896	-1.407547	2.515955	O	0.384346	-1.321573	2.663464
Cr	-1.263709	-1.019630	-0.093348	Cr	-1.082775	-1.088023	-0.011005
Cr	1.358479	-0.161008	-0.038352	Cr	1.322824	0.000989	0.091391

Table S84. Optimized coordinates for the $(Me_2C{=}N)_2Cr_2(CO)_7$ structure **7T-9.**

	M06-L				B3PW91-D3		
	x	y	z		x	y	z
C	1.667892	-1.750687	-1.300111	C	1.915492	-1.681633	-1.107827
C	2.379899	1.199123	-1.496023	C	2.255249	1.159009	-1.607183
C	-1.437162	1.226333	2.112046	C	-1.558039	1.454765	1.916101
C	0.864967	1.867949	1.024216	C	0.669631	2.011938	0.854114
O	1.777504	-2.787018	-1.813027	O	2.221006	-2.713088	-1.540332
O	2.984882	1.985568	-2.084892	O	2.785459	1.891859	-2.321029
O	-1.897519	1.443649	3.157247	O	-2.028872	1.885058	2.882348
O	1.666643	2.611965	1.448075	O	1.368131	2.859600	1.240761
C	-2.986328	-1.545850	-2.514073	C	-2.814903	-2.072324	-2.367429
C	-4.068312	-0.986957	-0.263399	C	-3.968536	-1.401240	-0.179949
H	-3.941479	-0.356447	0.619575	H	-3.916768	-0.677204	0.637244
H	-4.991240	-0.705609	-0.781862	H	-4.903101	-1.258202	-0.737710
H	-3.586733	-0.925504	-3.189874	H	-3.527094	-1.614264	-3.068030
H	-3.506546	-2.503880	-2.418983	H	-3.180709	-3.084241	-2.154755
C	3.009215	-0.446370	0.553568	C	3.059537	-0.190767	0.520504
C	-1.292086	2.515628	-0.305424	C	-1.591687	2.268148	-0.601128
O	3.993559	-0.653364	1.133558	O	4.093646	-0.291959	1.035806
O	-1.668522	3.527174	-0.732983	O	-2.117891	3.143947	-1.138254
H	-2.010310	-1.681611	-2.977637	H	-1.840539	-2.107786	-2.855496
H	-4.199695	-2.027693	0.056691	H	-4.003003	-2.416429	0.238395
C	0.975757	-3.092594	1.707495	C	1.223370	-2.725683	2.150946
H	0.563032	-3.967341	1.190643	H	0.851047	-3.720029	1.869191
H	1.918828	-2.822616	1.227240	H	2.109991	-2.500559	1.555840
H	1.173746	-3.401415	2.739514	H	1.504556	-2.779771	3.210918
C	-1.233879	-2.120576	2.525617	C	-1.056820	-1.843626	2.857406
H	-0.978534	-2.005007	3.585619	H	-0.772872	-1.610037	3.892795
H	-1.993739	-1.378581	2.269617	H	-1.869966	-1.179934	2.555877
H	-1.655238	-3.125618	2.411114	H	-1.415040	-2.881338	2.853450
C	-0.006501	-1.954444	1.670622	C	0.141855	-1.690623	1.947863
C	-2.893795	-0.874732	-1.180443	C	-2.788842	-1.264277	-1.099515
N	0.173778	-0.911379	0.962594	N	0.223477	-0.757240	1.085342
N	-1.864681	-0.209344	-0.786751	N	-1.829639	-0.477783	-0.770196
C	-0.659407	0.106765	-1.495618	C	-0.634064	-0.120339	-1.493675
O	-0.480363	0.071475	-2.706211	O	-0.467718	-0.219317	-2.699219
Cr	1.379589	-0.108273	-0.391765	Cr	1.401211	-0.054854	-0.353341
Cr	-0.624873	0.870325	0.416451	Cr	-0.723583	0.830106	0.327267

Table S85. Optimized coordinates for the $(Me_2C{=}N)_2Cr_2(CO)_7$ structure **7S-10.**

	M06-L				B3PW91-D3		
	x	y	z		x	y	z
C	-2.294079	-0.627454	-1.101341	C	-1.973884	-1.384111	-1.098842
C	-2.490364	1.215612	0.614639	C	-2.614486	-0.199172	1.041841
C	2.557291	0.521124	1.567065	C	2.106480	1.316510	1.494326
C	2.609317	1.023275	-1.036458	C	1.536447	2.347731	-0.849470
O	-3.068220	-1.353564	-1.587641	O	-2.420394	-2.296597	-1.665720
O	-3.429123	1.662207	1.137998	O	-3.520747	-0.351062	1.748382
O	3.217635	0.789818	2.495167	O	2.727294	1.658842	2.420267
O	3.332209	1.556655	-1.784257	O	1.832667	3.319001	-1.417361
C	-0.675909	0.303952	2.906420	C	-0.080875	-0.762946	3.020822
C	-2.149689	-1.630799	2.167296	C	0.194741	-3.137009	2.140816
H	-1.854737	-2.432682	2.854699	H	1.211599	-3.548343	2.181677
H	-2.940254	-1.051898	2.650800	H	-0.303010	-3.339074	3.092845
H	-0.442435	-0.207272	3.850471	H	0.670276	-0.926303	3.807186
H	-1.546328	0.935814	3.101180	H	-1.050433	-1.038463	3.453188
C	-1.349699	1.888693	-1.510703	C	-2.407320	1.245136	-0.995603
C	0.833570	1.902212	0.483342	C	-0.181026	1.934767	0.879771
O	-1.549325	2.775572	-2.236261	O	-3.134247	2.007026	-1.486121
O	0.651717	3.027509	0.780337	O	-0.739920	2.783120	1.466011
H	0.177543	0.923609	2.636343	H	-0.081679	0.291630	2.749275
H	-2.523459	-2.106743	1.261269	H	-0.314630	-3.654256	1.321365
C	0.505789	-4.188593	-0.859803	C	2.217289	-3.067189	-2.011900
H	-0.510546	-4.543383	-0.690893	H	1.547648	-3.919704	-1.878850
H	0.687897	-4.123713	-1.938695	H	2.121073	-2.688671	-3.037942
H	1.218712	-4.919884	-0.463958	H	3.257436	-3.395027	-1.884961
C	2.049946	-2.199712	-0.411185	C	2.680321	-0.705716	-1.137008
H	2.061515	-1.605913	-1.335732	H	2.352391	-0.120265	-2.002895
H	2.339080	-1.581244	0.468421	H	2.703467	-0.113594	-0.184727
H	2.859488	-2.924346	-0.517430	H	3.742895	-0.921847	-1.300618
C	0.715235	-2.854154	-0.228134	C	1.889001	-1.980565	-1.031172
C	-0.971681	-0.753839	1.896312	C	0.241513	-1.662493	1.877160
N	-0.043866	-1.058524	0.982730	N	0.613357	-1.179087	0.713974
N	-0.286852	-2.339318	0.400536	N	0.945252	-2.215268	-0.191079
O	0.397564	-0.449479	-2.891438	O	-0.011328	0.395836	-2.974295
C	0.094228	-0.103039	-1.815199	C	-0.279983	0.333934	-1.837781
Cr	1.438415	0.198748	0.135841	Cr	1.049315	0.828317	0.074371
Cr	-1.031534	0.483298	-0.340178	Cr	-1.241115	0.060508	-0.226991

Table S86. Optimized coordinates for the $(Me_2C{=}N)_2Cr_2(CO)_7$ structure **7T-11.**

	M06-L				B3PW91-D3		
	x	y	z		x	y	z
C	-2.710346	0.876817	0.305238	C	-2.618877	1.059525	0.422049
C	-1.009605	0.557867	-1.649057	C	-1.136897	0.601364	-1.654713
C	1.929115	-0.597361	-2.089346	C	1.917602	-0.980661	-1.979686
C	2.790171	-1.370830	0.205172	C	2.625335	-1.651648	0.329766
O	-3.571558	1.646783	0.474486	O	-3.421016	1.867179	0.657976
O	-0.965408	1.087584	-2.689057	O	-1.165637	1.079730	-2.713653
O	2.311611	-0.796155	-3.168312	O	2.290266	-1.356465	-3.010052
O	3.671560	-2.046089	0.554596	O	3.432329	-2.401126	0.692535
C	0.922972	3.679659	-1.220879	C	1.366880	3.486019	-1.496868
C	-0.557465	3.526471	0.879669	C	-0.112397	3.668715	0.607599
H	-0.174636	4.488725	1.233532	H	0.443613	4.557089	0.932091
H	-1.494467	3.735693	0.350999	H	-0.990668	4.025708	0.054372
H	1.409738	4.599886	-0.882897	H	1.989696	4.334888	-1.187814
H	0.085424	3.978250	-1.860622	H	0.582376	3.881984	-2.154449
C	-2.400809	-1.474911	-0.971633	C	-2.584682	-1.254389	-0.849519
C	0.189623	-1.965917	-0.385169	C	-0.078657	-1.931494	-0.420731
O	-3.067061	-2.212164	-1.580313	O	-3.368934	-1.908893	-1.402650
O	0.105710	-3.136124	-0.463473	O	-0.218251	-3.084403	-0.580179
H	1.622516	3.089661	-1.815354	H	1.972820	2.770885	-2.058539
H	-0.780058	2.878383	1.728795	H	-0.452929	3.105638	1.478621
C	0.873266	-0.768854	2.815460	C	0.825415	-0.519729	2.912019
H	0.299444	-1.579947	2.365497	H	0.181466	-1.332584	2.576805
H	1.773702	-1.200498	3.260550	H	1.694748	-0.959983	3.410662
H	0.272344	-0.340535	3.628854	H	0.273925	0.069372	3.659567
C	2.405267	1.171347	2.244716	C	2.502873	1.206321	2.126678
H	3.255982	0.559991	2.556672	H	3.298224	0.561006	2.512169
H	2.741600	1.847415	1.456735	H	2.901690	1.768698	1.280122
H	2.107835	1.778496	3.112692	H	2.246068	1.919059	2.926934
C	1.264225	0.285044	1.823016	C	1.291012	0.365112	1.784345
C	0.412119	2.896880	-0.060040	C	0.742215	2.837073	-0.299023
N	0.819458	1.681368	0.051208	N	0.960908	1.585538	-0.123560
N	0.255354	0.781560	1.001234	N	0.331990	0.823204	0.891855
O	-2.447128	-1.935284	2.373042	O	-2.474856	-1.721949	2.422763
C	-1.945820	-1.315838	1.526263	C	-1.947674	-1.143536	1.566979
Cr	1.319139	-0.296793	-0.304519	Cr	1.313949	-0.438594	-0.265951
Cr	-1.315855	-0.325985	0.010737	Cr	-1.336258	-0.220221	0.031122

Table S87. Optimized coordinates for the $(Me_2C{=}N)_2Cr_2(CO)_7$ structure **7T-12.**

	M06-L				B3PW91-D3		
	x	y	z		x	y	z
C	-2.515240	1.637875	-0.982618	C	-2.586007	-0.497841	-1.531249
C	-0.560907	-0.387651	-1.630031	C	-0.235142	-1.382141	-1.382823
C	2.637201	-1.871135	-0.469966	C	2.461896	-0.742049	-1.102302
C	1.270129	-1.136398	1.564998	C	2.466213	0.035376	1.381235
O	-3.097778	2.499612	-1.507660	O	-3.282136	-0.535887	-2.466222
O	0.271915	-0.765941	-2.411682	O	-0.015710	-1.992876	-2.363115
O	3.682222	-2.374875	-0.576011	O	3.301533	-0.970891	-1.866925
O	1.485641	-1.110068	2.718780	O	3.291222	0.403448	2.114023
C	2.804404	1.391700	-2.024991	C	2.266107	2.339417	-1.595960
C	1.339002	3.317823	-1.185708	C	-0.040898	3.415019	-1.706941
H	2.047079	3.943647	-0.628604	H	0.155027	4.386411	-1.232774
H	1.372327	3.655341	-2.224524	H	0.202821	3.530542	-2.769326
H	3.676588	2.052742	-1.994333	H	2.622224	3.367639	-1.730615
H	2.431926	1.399128	-3.055190	H	2.329808	1.843866	-2.574153
C	-2.959860	-0.952359	-0.661751	C	-2.443646	-1.989770	0.476739
C	0.034481	-2.769890	-0.129941	C	1.515082	-2.338929	0.554249
O	-3.825525	-1.675654	-0.958618	O	-3.104903	-2.881834	0.814070
O	-0.530897	-3.777565	-0.015807	O	1.764500	-3.455056	0.758246
H	3.105708	0.370618	-1.780585	H	2.920465	1.814079	-0.897864
H	0.337914	3.488470	-0.784012	H	-1.102971	3.179826	-1.609335
C	-0.848572	2.306985	2.488522	C	-2.711957	2.247025	1.484133
H	-1.793354	2.176615	1.951935	H	-3.283467	1.542094	0.871892
H	-0.833843	1.596839	3.325066	H	-2.733201	1.904066	2.527090
H	-0.801184	3.312059	2.918265	H	-3.195286	3.231973	1.458423
C	1.668538	2.485822	2.077413	C	-0.445205	3.437053	1.606709
H	1.769467	2.265371	3.143913	H	-0.651295	3.505279	2.681029
H	2.480467	1.994341	1.538939	H	0.622895	3.263319	1.458334
H	1.777753	3.571903	1.968729	H	-0.713086	4.409509	1.171329
C	0.322359	2.051030	1.604304	C	-1.293550	2.354197	1.011814
C	1.742527	1.883188	-1.103402	C	0.847417	2.356299	-1.119517
N	1.205819	1.024277	-0.296834	N	0.458511	1.455338	-0.271218
N	0.101764	1.407327	0.498632	N	-0.882437	1.476690	0.150994
C	-1.581911	-0.752049	1.504693	C	0.095224	-0.692790	1.800850
O	-1.717253	-1.346160	2.497328	O	-0.323060	-0.820479	2.886472
Cr	-1.554958	0.184161	-0.188581	Cr	-1.412660	-0.513811	-0.102760
Cr	0.930648	-1.087193	-0.231084	Cr	1.136358	-0.557830	0.236656

Table S88. Optimized coordinates for the $(Me_2C{=}N)_2Cr_2(CO)_7$ structure **7T-13.**

	M06-L				B3PW91-D3		
	x	y	z		x	y	z
C	-2.458676	-0.556902	-0.931911	C	-1.892090	-1.607039	-1.048678
C	-2.178069	1.655670	0.510731	C	-2.650872	-0.179892	0.971711
C	2.680864	0.683942	1.362572	C	1.885571	1.918075	1.319380
C	2.815117	0.655605	-1.119138	C	1.772061	2.260506	-1.140077
O	-3.357753	-1.215527	-1.270438	O	-2.304978	-2.561924	-1.563199
O	-2.911399	2.384981	1.042683	O	-3.572086	-0.236786	1.673322
O	3.410284	1.174557	2.135382	O	2.413177	2.545699	2.147777
O	3.670236	1.013633	-1.826278	O	2.287892	3.039024	-1.829584
C	-0.517479	0.663983	2.858583	C	-0.312270	-0.585652	2.887029
C	-2.251108	-1.114815	2.341251	C	-0.499049	-2.865126	1.754765
H	-2.051072	-1.812691	3.163769	H	0.274446	-3.399687	2.325573
H	-2.969147	-0.375866	2.706201	H	-1.440411	-2.964818	2.303530
H	-0.424261	0.235397	3.865673	H	0.315463	-1.033709	3.672728
H	-1.248752	1.473102	2.932123	H	-1.344417	-0.620963	3.247248
C	-1.237191	1.730582	-1.817852	C	-2.438028	1.013431	-1.181197
C	0.628180	1.870838	0.220521	C	-0.589895	1.926843	0.690915
O	-1.381408	2.511842	-2.667422	O	-3.193861	1.687500	-1.746511
O	0.696281	3.022795	0.467676	O	-1.185752	2.786785	1.222529
H	0.451079	1.070103	2.568690	H	-0.005392	0.451093	2.748616
H	-2.676117	-1.691797	1.521568	H	-0.575817	-3.338048	0.774289
C	-0.003870	-4.318810	-0.394860	C	2.800500	-3.168109	-1.352353
H	-1.071076	-4.486921	-0.250706	H	1.939261	-3.597934	-1.869062
H	0.225256	-4.379544	-1.465445	H	3.477553	-2.716061	-2.089151
H	0.562710	-5.118653	0.094079	H	3.361668	-3.966304	-0.849107
C	1.838737	-2.577314	-0.005963	C	3.445480	-1.382612	0.371353
H	1.966295	-1.890398	-0.866597	H	3.830744	-0.580189	-0.273464
H	2.216280	-2.089836	0.902227	H	3.095725	-0.927107	1.300362
H	2.489715	-3.425543	-0.225145	H	4.285044	-2.053807	0.584418
C	0.402261	-2.983890	0.129725	C	2.362124	-2.133490	-0.355375
C	-0.977317	-0.435205	1.958700	C	-0.117612	-1.416744	1.650938
N	-0.110006	-0.960690	1.083184	N	0.668682	-0.963373	0.680146
N	-0.528557	-2.249097	0.639822	N	1.090445	-1.976332	-0.225382
O	0.296192	-1.119836	-2.701244	O	0.073270	0.137336	-3.011754
C	-0.021248	-0.498238	-1.759079	C	-0.228673	0.137814	-1.887105
Cr	1.469226	0.081234	0.077472	Cr	0.957848	0.997318	0.005781
Cr	-0.998461	0.507471	-0.434556	Cr	-1.245037	-0.054136	-0.267773

Table S89. Optimized coordinates for the $(Me_2C{=}N)_2Fe_2(CO)_4$ structure **4S-1.**

	M06-L				B3PW91-D3		
	x	y	z		x	y	z
C	1.219060	2.285451	1.218176	C	1.202693	2.265000	1.226722
C	-1.142471	-1.898982	-1.233158	C	-1.141479	-1.886365	-1.219622
O	1.453594	3.067978	2.051896	O	1.425004	3.035252	2.066660
O	-1.653274	-2.494428	-2.096067	O	-1.665025	-2.472105	-2.073879
C	-2.143450	2.936992	0.000000	C	-2.114269	2.935488	0.000000
C	-3.424376	0.723279	0.000000	C	-3.398046	0.712322	0.000000
H	-4.013168	1.008373	0.879111	H	-3.983241	0.998697	0.883698
H	-4.013168	1.008373	-0.879111	H	-3.983241	0.998697	-0.883698
H	-2.684028	3.304807	0.879311	H	-2.654508	3.298920	0.883879
H	-2.684028	3.304807	-0.879311	H	-2.654508	3.298920	-0.883879
C	1.219060	2.285451	-1.218176	C	1.202693	2.265000	-1.226722
C	-1.142471	-1.898982	1.233158	C	-1.141479	-1.886365	1.219622
O	1.453594	3.067978	-2.051896	O	1.425004	3.035252	-2.066660
O	-1.653274	-2.494428	2.096067	O	-1.665025	-2.472105	2.073879
H	-1.135758	3.357522	0.000000	H	-1.102666	3.347620	0.000000
H	-3.285839	-0.359983	0.000000	H	-3.248777	-0.369863	0.000000
C	2.027865	-2.612910	-1.278138	C	2.017702	-2.596384	-1.283633
H	1.576666	-3.608805	-1.330747	H	1.568452	-3.594684	-1.329903
H	1.721223	-2.041591	-2.157303	H	1.694540	-2.019823	-2.155290
H	3.117409	-2.743303	-1.314279	H	3.110384	-2.713318	-1.324133
C	2.027865	-2.612910	1.278138	C	2.017702	-2.596384	1.283633
H	3.117409	-2.743303	1.314279	H	3.110384	-2.713318	1.324133
H	1.721223	-2.041591	2.157303	H	1.694540	-2.019823	2.155290
H	1.576666	-3.608805	1.330747	H	1.568452	-3.594684	1.329903
Fe	-0.282401	-0.999726	0.000000	Fe	-0.276450	-0.991979	0.000000
Fe	0.876013	1.078147	0.000000	Fe	0.878398	1.067106	0.000000
C	1.624842	-1.915569	0.000000	C	1.613173	-1.898695	0.000000
C	-2.104105	1.437083	0.000000	C	-2.074009	1.430144	0.000000
N	1.539458	-0.569106	0.000000	N	1.537470	-0.553493	0.000000
N	-1.000108	0.794014	0.000000	N	-0.971497	0.790128	0.000000

Table S90. Optimized coordinates for the $(Me_2C{=}N)_2Fe_2(CO)_4$ structure **4S-2.**

	M06-L				B3PW91-D3		
	x	y	z		x	y	z
C	1.846060	1.299174	-1.184930	C	1.793225	1.287646	-1.226000
C	-1.846060	-1.299174	-1.184930	C	-1.793225	-1.287646	-1.226000
O	2.204716	2.163581	-1.879045	O	2.117556	2.133696	-1.948724
O	-2.204716	-2.163581	-1.879045	O	-2.117556	-2.133696	-1.948724
C	-1.277932	2.961556	1.860061	C	-1.285525	2.857749	1.958304
C	1.277932	2.961556	1.860061	C	1.285525	2.857749	1.958304
H	1.312079	3.991220	1.485868	H	1.313255	3.918816	1.677441
H	1.347678	3.021227	2.952181	H	1.364991	2.809341	3.052537
H	-1.312079	3.991220	1.485868	H	-1.313255	3.918816	1.677441
H	-1.347678	3.021227	2.952181	H	-1.364991	2.809341	3.052537
C	1.846060	-1.299174	-1.184930	C	1.793225	-1.287646	-1.226000
C	-1.846060	1.299174	-1.184930	C	-1.793225	1.287646	-1.226000
O	2.204716	-2.163581	-1.879045	O	2.117556	-2.133696	-1.948724
O	-2.204716	2.163581	-1.879045	O	-2.117556	2.133696	-1.948724
H	-2.149387	2.420935	1.480405	H	-2.147181	2.347492	1.515613
H	2.149387	2.420935	1.480405	H	2.147181	2.347492	1.515613
C	1.277932	-2.961556	1.860061	C	1.285525	-2.857749	1.958304
H	1.312079	-3.991220	1.485868	H	1.313255	-3.918816	1.677441
H	2.149387	-2.420935	1.480405	H	2.147181	-2.347492	1.515613
H	1.347678	-3.021227	2.952181	H	1.364991	-2.809341	3.052537
C	-1.277932	-2.961556	1.860061	C	-1.285525	-2.857749	1.958304
H	-1.347678	-3.021227	2.952181	H	-1.364991	-2.809341	3.052537
H	-2.149387	-2.420935	1.480405	H	-2.147181	-2.347492	1.515613
H	-1.312079	-3.991220	1.485868	H	-1.313255	-3.918816	1.677441
Fe	-1.207943	0.000000	-0.147735	Fe	-1.186753	0.000000	-0.163727
Fe	1.207943	0.000000	-0.147735	Fe	1.186753	0.000000	-0.163727
C	0.000000	-2.289452	1.438431	C	0.000000	-2.219015	1.490491
C	0.000000	2.289452	1.438431	C	0.000000	2.219015	1.490491
N	0.000000	-1.210293	0.762395	N	0.000000	-1.190787	0.744961
N	0.000000	1.210293	0.762395	N	0.000000	1.190787	0.744961

Table S91. Optimized coordinates for the $(Me_2C{=}N)_2Fe_2(CO)_4$ structure **4S-3.**

	M06-L				B3PW91-D3		
	x	y	z		x	y	z
C	-0.421253	0.418553	2.711137	C	-0.492379	0.386795	2.661437
C	1.365149	-1.345041	-1.727020	C	1.404063	-1.274504	-1.737816
C	-0.421253	0.418553	-2.711137	C	-0.492379	0.386795	-2.661437
O	-1.091951	0.513200	3.663624	O	-1.204891	0.442059	3.579022
O	1.965631	-2.288860	-2.054954	O	2.039997	-2.179279	-2.086797
O	-1.091951	0.513200	-3.663624	O	-1.204891	0.442059	-3.579022
C	-1.980306	-2.385338	-1.278743	C	-1.893240	-2.457383	-1.281260
C	-1.980306	-2.385338	1.278743	C	-1.893240	-2.457383	1.281260
H	-3.073841	-2.325589	1.303247	H	-2.982381	-2.331247	1.337016
H	-1.718467	-3.447879	1.344894	H	-1.687474	-3.535391	1.305716
H	-3.073841	-2.325589	-1.303247	H	-2.982381	-2.331247	-1.337016
H	-1.718467	-3.447879	-1.344894	H	-1.687474	-3.535391	-1.305716
C	1.365149	-1.345041	1.727020	C	1.404063	-1.274504	1.737816
O	1.965631	-2.288860	2.054954	O	2.039997	-2.179279	2.086797
H	-1.572951	-1.870674	-2.150256	H	-1.432063	-1.986409	-2.151017
H	-1.572951	-1.870674	2.150256	H	-1.432063	-1.986409	2.151017
C	-0.096928	3.632294	1.272109	C	-0.089941	3.628890	1.278103
H	-0.951307	4.317575	1.247671	H	-0.934070	4.330021	1.256112
H	-0.177220	2.975536	2.141360	H	-0.180347	2.963489	2.140876
H	0.796844	4.256563	1.388859	H	0.822588	4.230403	1.390412
C	-0.096928	3.632294	-1.272109	C	-0.089941	3.628890	-1.278103
H	0.796844	4.256563	-1.388859	H	0.822588	4.230403	-1.390412
H	-0.177220	2.975536	-2.141360	H	-0.180347	2.963489	-2.140876
H	-0.951307	4.317575	-1.247671	H	-0.934070	4.330021	-1.256112
Fe	0.478759	0.111791	-1.196563	Fe	0.454990	0.130274	-1.185497
Fe	0.478759	0.111791	1.196563	Fe	0.454990	0.130274	1.185497
C	-0.018254	2.838764	0.000000	C	-0.029750	2.832443	0.000000
C	-1.448929	-1.807503	0.000000	C	-1.368064	-1.864788	0.000000
N	0.127814	1.562959	0.000000	N	0.089269	1.556036	0.000000
N	-0.567426	-0.883935	0.000000	N	-0.516246	-0.917747	0.000000

Table S92. Optimized coordinates for the $(Me_2C{=}N)_2Fe_2(CO)_4$ structure **4T-4.**

	M06-L				B3PW91-D3		
	x	y	z		x	y	z
C	1.562786	-2.514455	-0.420172	C	2.947776	-0.511327	-0.266203
C	-1.776081	1.740955	-0.990131	C	-1.772408	0.278867	-1.694518
C	0.308700	2.939232	-0.344953	C	-2.200218	1.796794	0.246633
O	2.346521	-3.357450	-0.613541	O	4.074640	-0.436710	-0.529862
O	-2.732927	2.082577	-1.566367	O	-2.214550	0.103083	-2.755862
O	0.587271	4.072752	-0.381133	O	-2.992629	2.605372	0.513730
C	-3.269210	-0.701212	0.261048	C	-1.826379	-2.660067	-0.328995
C	-2.609983	-0.348164	2.692491	C	-2.635261	-1.718912	1.915408
H	-3.364965	0.434021	2.833040	H	-3.635793	-1.497875	1.524349
H	-3.084230	-1.292932	2.981842	H	-2.669985	-2.727399	2.351220
H	-4.083925	0.025404	0.359280	H	-2.854264	-2.612161	-0.705082
H	-3.702131	-1.687231	0.466811	H	-1.654948	-3.679664	0.044010
C	-0.899031	-2.429650	-0.796541	C	1.401892	-2.471390	-0.076853
O	-1.637247	-3.195106	-1.278175	O	1.531063	-3.617105	-0.196731
H	-2.894963	-0.683262	-0.764076	H	-1.131206	-2.456660	-1.147104
H	-1.759487	-0.151348	3.344688	H	-2.379195	-0.992984	2.690644
C	3.632470	-0.493567	0.822586	C	2.785574	2.438729	0.691461
H	4.256949	-0.119052	1.640243	H	2.922072	3.335451	1.307100
H	3.144528	-1.415905	1.137459	H	3.101368	1.559484	1.258356
H	4.310260	-0.722272	-0.009000	H	3.439530	2.539444	-0.185238
C	3.176011	1.963139	0.391380	C	0.620703	3.615433	0.034669
H	4.268559	1.971023	0.353404	H	1.270787	4.338964	-0.473592
H	2.786070	2.528733	-0.456225	H	-0.288449	3.457196	-0.550045
H	2.873060	2.487923	1.304504	H	0.346589	4.050635	1.005616
Fe	-0.237687	1.259691	-0.226100	Fe	-1.046965	0.526349	-0.092475
Fe	0.310287	-1.288428	-0.145038	Fe	1.224123	-0.707668	0.100863
C	2.639091	0.554846	0.405668	C	1.346215	2.308943	0.248540
C	-2.181404	-0.398703	1.251392	C	-1.619523	-1.675889	0.798972
N	1.422326	0.281364	0.113357	N	0.761710	1.187459	0.070069
N	-0.943237	-0.184882	0.963035	N	-0.554716	-0.936651	0.929471

Table S93. Optimized coordinates for the $(Me_2C{=}N)_2Fe_2(CO)_4$ structure **4T-5.**

	M06-L				B3PW91-D3		
	x	y	z		x	y	z
C	-2.309193	-1.862495	-0.241642	C	2.947761	-0.511212	0.266332
C	1.525763	0.946109	-1.659093	C	-1.772338	0.278843	1.694576
O	-3.335985	-2.377995	-0.439149	O	4.074590	-0.436511	0.530118
O	2.036580	0.884314	-2.708938	O	-2.214443	0.103053	2.755933
C	2.950623	-0.614647	2.334628	C	-2.635339	-1.718872	-1.915261
C	2.960492	-1.494641	-0.058041	C	-1.826260	-2.660114	0.329036
H	3.892732	-0.957837	-0.262512	H	-2.854119	-2.612251	0.705197
H	3.241772	-2.510584	0.246347	H	-1.654827	-3.679693	-0.044020
H	3.813956	0.036512	2.159460	H	-3.635846	-1.497889	-1.524108
H	3.347683	-1.567256	2.704697	H	-2.670065	-2.727334	-2.351132
C	0.035872	-2.725822	-0.379249	C	1.402003	-2.471378	0.076672
C	0.924147	2.768009	0.004471	C	-2.200303	1.796664	-0.246631
O	0.477520	-3.763930	-0.673622	O	1.531223	-3.617100	0.196431
O	1.132688	3.914260	0.098943	O	-2.992767	2.605185	-0.513747
H	2.310431	-0.168262	3.095400	H	-2.379358	-0.992890	-2.690475
H	2.370550	-1.549329	-0.974971	H	-1.131035	-2.456720	1.147104
C	-3.606409	0.790596	0.769608	C	2.785487	2.438798	-0.691563
H	-4.136395	1.470911	1.443777	H	2.921890	3.335427	-1.307360
H	-4.264688	0.621018	-0.091051	H	3.439451	2.539730	0.185106
H	-3.449803	-0.164834	1.272750	H	3.101349	1.559485	-1.258314
C	-2.337910	2.891133	0.123111	C	0.620583	3.615436	-0.034761
H	-2.226825	3.397271	1.089698	H	0.346366	4.050549	-1.005719
H	-1.531777	3.222844	-0.532570	H	-0.288517	3.457188	0.550032
H	-3.301010	3.207078	-0.290841	H	1.270663	4.339044	0.473396
Fe	0.680831	1.023172	-0.092828	Fe	-1.046962	0.526306	0.092497
Fe	-0.703890	-1.149799	0.036332	Fe	1.224152	-0.707648	-0.100890
C	-2.307389	1.399766	0.316248	C	1.346147	2.308969	-0.248595
C	2.194493	-0.833389	1.053857	C	-1.619509	-1.675885	-0.798906
N	-1.244569	0.715495	0.113638	N	0.761685	1.187473	-0.070064
N	0.939363	-0.513515	1.001714	N	-0.554700	-0.936656	-0.929474

Table S94. Optimized coordinates for the $(Me_2C{=}N)_2Fe_2(CO)_4$ structure **4T-6.**

	M06-L				B3PW91-D3		
	x	y	z		x	y	z
C	1.978052	1.375554	-1.126196	C	1.930920	1.353115	-1.193061
C	-1.978052	-1.375554	-1.126196	C	-1.930920	-1.353115	-1.193061
O	2.412743	2.306695	-1.673240	O	2.352125	2.246689	-1.797973
O	-2.412743	-2.306695	-1.673240	O	-2.352125	-2.246689	-1.797973
C	-1.276541	2.916508	1.841270	C	-1.279072	2.803862	1.986778
C	1.276541	2.916508	1.841270	C	1.279072	2.803862	1.986778
H	1.330954	3.948372	1.475071	H	1.310389	3.877634	1.758747
H	1.316329	2.968790	2.935056	H	1.328861	2.707512	3.079788
H	-1.330954	3.948372	1.475071	H	-1.310389	3.877634	1.758747
H	-1.316329	2.968790	2.935056	H	-1.328861	2.707512	3.079788
C	1.978052	-1.375554	-1.126196	C	1.930920	-1.353115	-1.193061
C	-1.978052	1.375554	-1.126196	C	-1.930920	1.353115	-1.193061
O	2.412743	-2.306695	-1.673240	O	2.352125	-2.246689	-1.797973
O	-2.412743	2.306695	-1.673240	O	-2.352125	2.246689	-1.797973
H	-2.149508	2.366559	1.481070	H	-2.155358	2.318167	1.548789
H	2.149508	2.366559	1.481070	H	2.155358	2.318167	1.548789
C	1.276541	-2.916508	1.841270	C	1.279072	-2.803862	1.986778
H	1.330954	-3.948372	1.475071	H	1.310389	-3.877634	1.758747
H	2.149508	-2.366559	1.481070	H	2.155358	-2.318167	1.548789
H	1.316329	-2.968790	2.935056	H	1.328861	-2.707512	3.079788
C	-1.276541	-2.916508	1.841270	C	-1.279072	-2.803862	1.986778
H	-1.316329	-2.968790	2.935056	H	-1.328861	-2.707512	3.079788
H	-2.149508	-2.366559	1.481070	H	-2.155358	-2.318167	1.548789
H	-1.330954	-3.948372	1.475071	H	-1.310389	-3.877634	1.758747
Fe	-1.207141	0.000000	-0.249578	Fe	-1.184061	0.000000	-0.264838
Fe	1.207141	0.000000	-0.249578	Fe	1.184061	0.000000	-0.264838
C	0.000000	-2.264813	1.384519	C	0.000000	-2.188840	1.464149
C	0.000000	2.264813	1.384519	C	0.000000	2.188840	1.464149
N	0.000000	-1.220213	0.655782	N	0.000000	-1.210184	0.652742
N	0.000000	1.220213	0.655782	N	0.000000	1.210184	0.652742

Table S95. Optimized coordinates for the $(Me_2C{=}N)_2Fe_2(CO)_4$ structure **4T-7.**

	M06-L				B3PW91-D3		
	x	y	z		x	y	z
C	-3.013275	0.551786	0.056042	C	3.032181	-0.391245	0.042070
O	-4.183927	0.513498	0.070887	O	4.193393	-0.290101	0.093451
C	-1.219360	-1.648063	-2.724810	C	1.160191	1.674451	-2.730315
C	0.589588	-3.020556	-1.507325	C	-0.749768	2.950903	-1.559882
H	0.050121	-3.960244	-1.327148	H	-0.267793	3.928329	-1.411748
H	1.080463	-3.125732	-2.479359	H	-1.234006	2.983271	-2.543217
H	-1.681061	-2.584131	-3.055322	H	1.524103	2.642551	-3.095964
H	-0.621103	-1.275166	-3.564720	H	0.596713	1.208789	-3.551248
C	1.308966	2.181933	-0.805713	C	-1.278171	-2.236946	-0.734989
C	-1.102130	2.290261	0.798236	C	1.277287	-2.237402	0.734926
O	1.350756	3.227051	-1.330182	O	-1.325747	-3.280228	-1.252017
O	-1.047248	3.336232	1.319896	O	1.324442	-3.280704	1.251952
H	-1.999791	-0.912991	-2.507892	H	2.010782	1.029356	-2.487782
H	1.354166	-2.910565	-0.735032	H	-1.516283	2.818733	-0.792099
C	1.060875	-1.736166	2.727451	C	-1.159532	1.674661	2.730366
H	1.904919	-1.075773	2.508329	H	-2.010384	1.029931	2.487777
H	0.497163	-1.305794	3.563767	H	-0.596233	1.208687	3.551244
H	1.435351	-2.708233	3.064321	H	-1.523052	2.642869	3.096118
C	-0.864472	-2.944531	1.514189	C	0.750893	2.950498	1.560024
H	-1.366277	-2.995883	2.485012	H	1.235144	2.982609	2.543361
H	-1.613131	-2.770307	0.738134	H	1.517360	2.818101	0.792231
H	-0.413651	-3.931558	1.343888	H	0.269283	3.928114	1.411966
C	0.187154	-1.888921	1.531866	C	-0.276101	1.859535	1.536497
C	-0.360269	-1.872695	-1.529922	C	0.276819	1.859557	-1.536438
N	-0.484516	-1.007345	-0.558174	N	0.442056	1.024346	-0.549643
N	0.391130	-1.044142	0.555099	N	-0.441657	1.024465	0.549633
Fe	-1.233802	0.690984	0.053945	Fe	1.275909	-0.629646	0.016149
Fe	1.292205	0.579246	-0.057208	Fe	-1.276141	-0.629197	-0.016197
O	4.214612	0.138684	-0.058784	O	-4.193495	-0.288522	-0.093435
C	3.052007	0.281469	-0.050375	C	-3.032322	-0.390120	-0.042077

Table S96. Optimized coordinates for the $(Me_2C=N)_2Fe_2(CO)_4$ structure **4S-8.**

	M06-L				B3PW91-D3		
	x	y	z		x	y	z
C	0.203332	0.882069	2.150631	C	-0.459282	-0.880171	2.132786
O	-0.001107	0.560029	3.259412	O	-0.396762	-0.620595	3.268066
C	-1.879516	2.876529	-0.884196	C	1.721580	-2.949653	-0.637666
C	-3.355725	0.879068	-1.590741	C	3.319875	-1.111337	-1.495784
H	-4.279955	1.113980	-1.046185	H	4.241341	-1.425161	-0.986040
H	-3.450381	1.348602	-2.576388	H	3.305078	-1.625443	-2.466412
H	-2.799545	3.466098	-0.841879	H	2.601847	-3.600665	-0.590074
H	-1.340187	3.170023	-1.795486	H	1.139618	-3.236575	-1.527460
C	2.214888	-0.156664	-1.499607	C	-1.982651	0.272056	-1.694407
C	2.260334	1.431410	0.676591	C	-2.338437	-1.319026	0.496477
O	3.235849	0.166205	-1.978462	O	-2.913682	0.004494	-2.349801
O	3.367363	1.723737	0.906509	O	-3.470428	-1.550654	0.621472
H	-1.250066	3.154307	-0.027263	H	1.096307	-3.141179	0.247737
H	-3.307992	-0.203047	-1.732940	H	3.371619	-0.035839	-1.682488
C	-1.964592	-2.886569	0.304630	C	2.198712	2.880702	0.126505
H	-1.181937	-3.210318	-0.383590	H	1.243340	3.187976	-0.309108
H	-1.711411	-3.238327	1.311435	H	2.328599	3.368365	1.100348
H	-2.914369	-3.359064	0.033259	H	3.013762	3.229482	-0.522350
C	-3.175017	-0.793203	1.144585	C	3.345691	0.812044	1.123867
H	-3.245009	-1.327715	2.095842	H	3.314034	1.296487	2.108029
H	-2.994843	0.265754	1.339316	H	3.248759	-0.266282	1.261028
H	-4.149951	-0.895083	0.652927	H	4.330414	1.035316	0.692814
C	-2.100574	-1.404507	0.310891	C	2.255337	1.391951	0.275001
C	-2.160439	1.411999	-0.880120	C	2.107009	-1.503825	-0.710572
N	-1.222126	0.678433	-0.337829	N	1.227216	-0.680483	-0.209462
N	-1.231052	-0.721652	-0.372408	N	1.315599	0.709060	-0.300391
Fe	0.548153	1.072697	0.434603	Fe	-0.607482	-1.025066	0.400235
Fe	0.730772	-0.842628	-0.866280	Fe	-0.634256	0.891242	-0.786075
O	1.898502	-3.158138	0.488146	O	-2.099206	2.908138	0.715918
C	1.380806	-2.289994	-0.105772	C	-1.452601	2.167566	0.088718

Table S97. Optimized coordinates for the $(Me_2C{=}N)_2Fe_2(CO)_4$ structure **4S-9.**

	M06-L				B3PW91-D3		
	x	y	z		x	y	z
C	-2.673269	1.534613	-0.279802	C	-2.818492	1.485167	-0.364894
C	3.470032	-0.658051	1.029710	C	3.421140	-0.694393	1.073726
O	-2.730379	2.707873	-0.299834	O	-3.011075	2.630997	-0.517334
O	4.294694	-0.826523	1.841173	O	4.207778	-0.884532	1.908319
C	1.250160	2.563648	0.010644	C	1.341458	2.613599	-0.079518
C	0.178178	1.071565	1.782494	C	0.231124	1.275986	1.789291
H	0.721338	1.669454	2.518244	H	0.905257	1.768815	2.498493
H	-0.863795	1.428913	1.749230	H	-0.716801	1.832611	1.779657
H	2.050958	2.944214	0.650967	H	2.147530	3.002343	0.551396
H	0.398287	3.252690	0.100009	H	0.505936	3.328937	-0.034663
C	-4.182263	-0.223378	0.375914	C	-4.135001	-0.311703	0.482162
C	3.303738	0.588289	-1.018281	C	3.370912	0.508552	-0.981793
O	-5.240998	-0.263889	0.878940	O	-5.181229	-0.393949	0.998295
O	4.072531	1.206022	-1.643644	O	4.175173	1.054927	-1.617570
H	1.587069	2.580337	-1.027556	H	1.684805	2.561818	-1.115560
H	0.136142	0.033347	2.132326	H	0.009393	0.265012	2.148238
C	-1.621506	-2.692925	-0.584522	C	-1.632460	-2.602880	-0.739106
H	-2.585084	-2.145168	-0.678360	H	-2.598417	-2.041636	-0.809645
H	-1.573140	-3.296984	-1.497202	H	-1.562638	-3.111040	-1.710185
H	-1.724430	-3.378140	0.263095	H	-1.740391	-3.368897	0.038010
C	0.941123	-2.255943	-0.218666	C	0.935113	-2.215754	-0.265270
H	1.680237	-1.948617	-1.026789	H	1.697922	-1.881695	-1.049258
H	1.307821	-1.805519	0.754902	H	1.280834	-1.828614	0.740673
H	1.056889	-3.333801	-0.098887	H	1.032967	-3.302707	-0.208268
C	-0.434887	-1.794788	-0.449117	C	-0.437717	-1.724111	-0.506947
C	0.818779	1.186941	0.430648	C	0.851699	1.273298	0.415458
N	0.444516	0.309273	-0.570273	N	0.479468	0.345232	-0.516588
N	-0.659046	-0.496964	-0.443561	N	-0.641292	-0.429223	-0.451856
Fe	2.164161	-0.341340	-0.105600	Fe	2.157159	-0.337706	-0.091253
Fe	-2.554849	-0.166144	-0.257106	Fe	-2.534292	-0.183183	-0.184213

Table S98. Optimized coordinates for the $(Me_2C{=}N)_2Cr_2(CO)_6$ structure **6S-1.**

	M06-L				B3PW91-D3		
	x	y	z		x	y	z
C	-0.063905	2.806038	1.239648	C	-0.063489	2.769948	1.222177
C	1.148379	2.381681	-0.897473	C	1.171170	2.335762	-0.870387
C	-1.148379	-2.381681	-0.897473	C	-1.171170	-2.335762	-0.870387
C	1.611272	-1.912715	-0.509686	C	1.596468	-1.903717	-0.510550
O	-0.136507	3.784852	1.871385	O	-0.137560	3.739326	1.860702
O	1.938438	3.019572	-1.470055	O	1.977117	2.954380	-1.433051
O	-1.938438	-3.019572	-1.470055	O	-1.977117	-2.954380	-1.433051
O	2.661805	-2.260207	-0.886897	O	2.645327	-2.251077	-0.878312
C	0.771610	-1.009721	3.638363	C	0.836268	-0.963061	3.620564
C	-0.771610	1.009721	3.638363	C	-0.836268	0.963061	3.620564
H	-1.528183	1.500917	3.024736	H	-1.627383	1.396113	3.004458
H	-1.233735	0.538236	4.511148	H	-1.260930	0.459989	4.497505
H	0.088841	-1.777569	4.022419	H	0.197867	-1.776781	3.992325
H	1.233735	-0.538236	4.511148	H	1.260930	-0.459989	4.497505
C	-1.611272	1.912715	-0.509686	C	-1.596468	1.903717	-0.510550
C	0.063905	-2.806038	1.239648	C	0.063489	-2.769948	1.222177
O	-2.661805	2.260207	-0.886897	O	-2.645327	2.251077	-0.878312
O	0.136507	-3.784852	1.871385	O	0.137560	-3.739326	1.860702
H	1.528183	-1.500917	3.024736	H	1.627383	-1.396113	3.004458
H	-0.088841	1.777569	4.022419	H	-0.197867	1.776781	3.992325
C	-0.097955	1.262944	-3.504424	C	-0.084507	1.265539	-3.503689
H	-0.765781	1.111231	-4.358695	H	-0.713006	1.096796	-4.386259
H	-0.461825	2.100192	-2.906462	H	-0.488800	2.098840	-2.926074
H	0.884469	1.528460	-3.912449	H	0.916339	1.541222	-3.861874
C	0.097955	-1.262944	-3.504424	C	0.084507	-1.265539	-3.503689
H	0.765781	-1.111231	-4.358695	H	0.713006	-1.096796	-4.386259
H	0.461825	-2.100192	-2.906462	H	0.488800	-2.098840	-2.926074
H	-0.884469	-1.528460	-3.912449	H	-0.916339	-1.541222	-3.861874
C	0.000000	0.000000	-2.695723	C	0.000000	0.000000	-2.685271
C	0.000000	0.000000	2.849655	C	0.000000	0.000000	2.829293
N	0.000000	0.000000	-1.415043	N	0.000000	0.000000	-1.406245
N	0.000000	0.000000	1.552514	N	0.000000	0.000000	1.533844
Cr	0.000000	1.296920	0.115097	Cr	0.000000	1.275857	0.110744
Cr	0.000000	-1.296920	0.115097	Cr	0.000000	-1.275857	0.110744

Table S99. Optimized coordinates for the $(Me_2C{=}N)_2Cr_2(CO)_6$ structure **6T-2.**

	M06-L				B3PW91-D3		
	x	y	z		x	y	z
C	-0.194208	2.650271	1.353678	C	-0.156758	2.622783	1.340376
C	-0.194208	2.650271	-1.353678	C	-0.156758	2.622783	-1.340376
C	0.747491	-2.556622	0.000000	C	0.732960	-2.531483	0.000000
C	-1.679963	-1.699490	1.302206	C	-1.697008	-1.667298	1.284888
O	-0.448776	3.429912	2.181337	O	-0.407291	3.403450	2.162998
O	-0.448776	3.429912	-2.181337	O	-0.407291	3.403450	-2.162998
O	1.528461	-3.416723	0.000000	O	1.519352	-3.377727	0.000000
O	-2.472506	-2.093129	2.051819	O	-2.491979	-2.050741	2.028914
C	1.477849	-1.542217	-3.095457	C	1.459108	-1.572134	-3.087321
C	2.066564	0.933871	-3.102632	C	2.074634	0.901299	-3.097857
H	3.098892	0.658595	-3.345268	H	3.093774	0.588823	-3.359854
H	1.572289	1.167661	-4.052937	H	1.563705	1.155169	-4.036464
H	2.469474	-1.975187	-2.917144	H	2.425739	-2.032911	-2.842342
H	1.367866	-1.441375	-4.180349	H	1.419256	-1.460843	-4.177813
C	-1.683884	1.126752	0.000000	C	-1.660544	1.179998	0.000000
C	-1.679963	-1.699490	-1.302206	C	-1.697008	-1.667298	-1.284888
O	-2.859268	1.166211	0.000000	O	-2.831672	1.251386	0.000000
O	-2.472506	-2.093129	-2.051819	O	-2.491979	-2.050741	-2.028914
H	0.726116	-2.241287	-2.719556	H	0.662283	-2.247026	-2.762850
H	2.074751	1.835283	-2.486080	H	2.124468	1.796571	-2.473588
C	2.066564	0.933871	3.102632	C	2.074634	0.901299	3.097857
H	3.098892	0.658595	3.345268	H	3.093774	0.588823	3.359854
H	2.074751	1.835283	2.486080	H	2.124468	1.796571	2.473588
H	1.572289	1.167661	4.052937	H	1.563705	1.155169	4.036464
C	1.477849	-1.542217	3.095457	C	1.459108	-1.572134	3.087321
H	1.367866	-1.441375	4.180349	H	1.419256	-1.460843	4.177813
H	0.726116	-2.241287	2.719556	H	0.662283	-2.247026	2.762850
H	2.469474	-1.975187	2.917144	H	2.425739	-2.032911	2.842342
C	1.360878	-0.204579	2.416394	C	1.343798	-0.227293	2.407235
C	1.360878	-0.204579	-2.416394	C	1.343798	-0.227293	-2.407235
N	0.710588	-0.049942	1.330371	N	0.684509	-0.062419	1.330387
N	0.710588	-0.049942	-1.330371	N	0.684509	-0.062419	-1.330387
Cr	0.147417	1.376267	0.000000	Cr	0.158675	1.353886	0.000000
Cr	-0.394714	-1.033254	0.000000	Cr	-0.408445	-1.017957	0.000000

Table S100. Optimized coordinates for the $(Me_2C{=}N)_2Cr_2(CO)_6$ structure **6T-3.**

	M06-L				B3PW91-D3		
	x	y	z		x	y	z
C	-1.088706	-2.828991	0.816379	C	-2.955571	-0.466875	0.802798
C	0.449424	-2.609248	-1.105575	C	-2.023395	-1.762752	-1.045190
C	1.088706	2.828991	0.816379	C	2.955571	0.466875	0.802798
C	-0.449424	2.609248	-1.105575	C	2.023395	1.762752	-1.045190
O	-1.796466	-3.707238	1.110959	O	-4.066684	-0.300980	1.096135
O	0.620149	-3.314692	-2.025974	O	-2.560571	-2.303661	-1.928395
O	1.796466	3.707238	1.110959	O	4.066684	0.300980	1.096135
O	-0.620149	3.314692	-2.025974	O	2.560571	2.303661	-1.928395
C	-3.345294	0.988403	-0.920429	C	-0.842484	3.400475	-0.936134
C	-3.054399	-1.500753	-1.271805	C	-2.804202	1.859157	-1.398255
H	-3.748232	-1.914527	-0.529871	H	-3.544962	2.231453	-0.676828
H	-3.636649	-1.322884	-2.181181	H	-2.907626	2.463503	-2.307394
H	-4.377854	0.760658	-0.635811	H	-1.613078	4.158656	-0.751130
H	-3.369499	1.277482	-1.978057	H	-0.485108	3.546781	-1.964859
C	1.293938	-2.258591	1.577692	C	-1.195816	-2.124804	1.647427
C	-1.293938	2.258591	1.577692	C	1.195816	2.124804	1.647427
O	1.952678	-2.715221	2.419651	O	-1.187202	-2.859991	2.541517
O	-1.952678	2.715221	2.419651	O	1.187202	2.859991	2.541517
H	-2.982326	1.840785	-0.342254	H	-0.001492	3.555134	-0.256612
H	-2.281011	-2.245453	-1.468922	H	-3.030913	0.814676	-1.621920
C	3.345294	-0.988403	-0.920429	C	0.842484	-3.400475	-0.936134
H	4.377854	-0.760658	-0.635811	H	1.613078	-4.158656	-0.751130
H	2.982326	-1.840785	-0.342254	H	0.001492	-3.555134	-0.256612
H	3.369499	-1.277482	-1.978057	H	0.485108	-3.546781	-1.964859
C	3.054399	1.500753	-1.271805	C	2.804202	-1.859157	-1.398255
H	3.636649	1.322884	-2.181181	H	2.907626	-2.463503	-2.307394
H	2.281011	2.245453	-1.468922	H	3.030913	-0.814676	-1.621920
H	3.748232	1.914527	-0.529871	H	3.544962	-2.231453	-0.676828
C	2.467188	0.218275	-0.753892	C	1.421087	-2.012811	-0.813319
C	-2.467188	-0.218275	-0.753892	C	-1.421087	2.012811	-0.813319
N	1.293938	0.151000	-0.230080	N	0.781343	-1.036072	-0.279178
N	-1.293938	-0.151000	-0.230080	N	-0.781343	1.036072	-0.279178
Cr	0.118295	-1.466605	0.277306	Cr	-1.187202	-0.815116	0.263124
Cr	-0.118295	1.466605	0.277306	Cr	1.187202	0.815116	0.263124

Table S101. Optimized coordinates for the $(Me_2C{=}N)_2Cr_2(CO)_6$ structure **6S-4.**

	M06-L				B3PW91-D3		
	x	y	z		x	y	z
C	-1.473620	2.115546	-0.266924	C	1.858456	-2.159565	-0.249429
C	0.371053	0.217591	-1.698084	C	-0.298377	-0.771928	-1.670539
C	1.473620	2.115547	0.266919	C	-1.858459	-2.159565	0.249425
C	-0.371053	0.217594	1.698084	C	0.298374	-0.771932	1.670538
O	-1.756092	3.230968	-0.112728	O	2.383828	-3.181611	-0.128696
O	1.070713	0.153035	-2.638798	O	-0.893235	-0.932484	-2.664681
O	1.756091	3.230969	0.112722	O	-2.383825	-3.181612	0.128686
O	-1.070713	0.153041	2.638798	O	0.893232	-0.932491	2.664679
C	3.157600	-3.111758	-0.228283	C	-2.097373	3.297589	-0.346388
C	4.328577	-1.177050	-1.450844	C	-3.781611	1.826935	-1.642436
H	5.306710	-1.506375	-1.083209	H	-4.648816	2.352119	-1.220432
H	4.188988	-1.624658	-2.441263	H	-3.532355	2.316757	-2.592822
H	4.098098	-3.458478	0.214005	H	-2.908741	3.913239	0.063278
H	3.016410	-3.676621	-1.156414	H	-1.734479	3.798320	-1.254185
C	-1.905024	0.440915	-2.187889	C	1.980911	-0.298420	-2.035611
C	1.905024	0.440921	2.187888	C	-1.980913	-0.298423	2.035611
O	-2.346372	0.561591	-3.257413	O	2.500232	-0.202711	-3.066000
O	2.346371	0.561599	3.257412	O	-2.500234	-0.202717	3.066001
H	2.336424	-3.332212	0.454265	H	-1.284000	3.230565	0.380614
H	4.322414	-0.091554	-1.552455	H	-4.042716	0.783138	-1.832517
C	-3.157599	-3.111757	0.228290	C	2.097381	3.297586	0.346392
H	-4.098098	-3.458478	-0.213998	H	2.908752	3.913232	-0.063275
H	-2.336423	-3.332213	-0.454258	H	1.284008	3.230564	-0.380611
H	-3.016410	-3.676618	1.156421	H	1.734488	3.798319	1.254188
C	-4.328577	-1.177047	1.450847	C	3.781615	1.826926	1.642439
H	-4.188988	-1.624654	2.441267	H	3.532360	2.316748	2.592826
H	-4.322414	-0.091551	1.552456	H	4.042717	0.783128	1.832520
H	-5.306710	-1.506372	1.083212	H	4.648821	2.352108	1.220435
C	-3.235729	-1.638733	0.524502	C	2.609935	1.918387	0.692407
C	3.235729	-1.638734	-0.524498	C	-2.609933	1.918392	-0.692402
N	-2.403087	-0.815053	0.043524	N	2.067039	0.878895	0.224894
N	2.403088	-0.815053	-0.043522	N	-2.067039	0.878898	-0.224891
Cr	1.105137	0.281307	0.506829	Cr	-1.089877	-0.453743	0.410685
Cr	-1.105137	0.281305	-0.506830	Cr	1.089875	-0.453743	-0.410684

Table S102. Optimized coordinates for the $(Me_2C{=}N)_2Cr_2(CO)_6$ structure **6S-5.**

	M06-L				B3PW91-D3		
	x	y	z		x	y	z
C	-0.216591	2.988475	-0.232505	C	-0.298710	2.957559	-0.223572
C	1.247613	1.081079	-0.869843	C	1.199910	1.180593	-0.961224
C	0.216591	-2.988475	-0.232505	C	0.298710	-2.957559	-0.223572
C	-1.247613	-1.081079	-0.869843	C	-1.199910	-1.180593	-0.961224
O	0.033480	4.108912	0.003092	O	-0.088391	4.086254	-0.007330
O	2.432398	1.010204	-0.942991	O	2.361013	1.291632	-1.146745
O	-0.033480	-4.108912	0.003092	O	0.088391	-4.086254	-0.007330
O	-2.432398	-1.010204	-0.942991	O	-2.361013	-1.291632	-1.146745
C	2.713387	-1.141590	2.039938	C	2.726511	-1.124034	2.076911
C	1.513029	0.744493	3.302438	C	1.519459	0.776597	3.312557
H	1.299438	0.269129	4.267877	H	1.317102	0.304635	4.283999
H	2.504231	1.197211	3.389302	H	2.516243	1.226996	3.379962
H	3.017347	-1.585896	2.992716	H	3.041023	-1.514515	3.052374
H	3.558050	-0.544572	1.678780	H	3.562184	-0.532684	1.679385
C	-0.761355	1.567158	-2.411978	C	-0.893543	1.558764	-2.355340
C	0.761355	-1.567158	-2.411978	C	0.893543	-1.558764	-2.355340
O	-0.881856	1.785785	-3.552883	O	-1.095369	1.817019	-3.472051
O	0.881856	-1.785785	-3.552883	O	1.095369	-1.817019	-3.472051
H	2.520986	-1.946325	1.321701	H	2.534823	-1.966781	1.403816
H	0.776114	1.531462	3.130621	H	0.783457	1.565589	3.141043
C	-2.713387	1.141590	2.039938	C	-2.726511	1.124034	2.076911
H	-2.520986	1.946325	1.321701	H	-2.534823	1.966781	1.403816
H	-3.558050	0.544572	1.678780	H	-3.562184	0.532684	1.679385
H	-3.017347	1.585896	2.992716	H	-3.041023	1.514515	3.052374
C	-1.513029	-0.744493	3.302438	C	-1.519459	-0.776597	3.312557
H	-2.504231	-1.197211	3.389302	H	-2.516243	-1.226996	3.379962
H	-0.776114	-1.531462	3.130621	H	-0.783457	-1.565589	3.141043
H	-1.299438	-0.269129	4.267877	H	-1.317102	-0.304635	4.283999
C	-1.513029	0.273223	2.213701	C	-1.513816	0.254915	2.226161
C	1.513029	-0.273223	2.213701	C	1.513816	-0.254915	2.226161
N	0.551775	-0.439457	1.358437	N	0.558202	-0.426021	1.369099
N	-0.551775	0.439457	1.358437	N	-0.558202	0.426021	1.369099
Cr	-0.528354	1.210533	-0.608263	Cr	-0.558202	1.182864	-0.582010
Cr	0.528354	-1.210533	-0.608263	Cr	0.558202	-1.182864	-0.582010

Table S103. Optimized coordinates for the $(Me_2C{=}N)_2Cr_2(CO)_6$ structure **6S-6.**

	M06-L				B3PW91-D3		
	x	y	z		x	y	z
C	3.710911	-0.463157	0.293983	C	-1.131866	-1.944511	-1.021285
C	2.440736	1.847288	0.384448	C	-1.662965	0.215634	-1.996172
C	-3.009380	0.496417	-1.163435	C	1.971208	0.797046	1.864288
C	-3.546769	-0.547102	1.087644	C	1.059060	-1.447560	1.020282
O	4.796643	-0.831419	0.509699	O	-1.347939	-2.998181	-1.450369
O	2.699126	2.957145	0.648255	O	-2.184306	0.474274	-3.007838
O	-3.651335	0.955341	-2.036082	O	2.096417	1.190693	2.944668
O	-4.548116	-0.775709	1.654063	O	0.714564	-2.371790	1.647161
C	-0.069219	2.156492	-1.577178	C	1.612459	3.395879	-0.373753
C	1.684690	0.422716	-2.363888	C	-0.904175	3.389708	-0.814500
H	1.972380	1.260752	-2.998151	H	-1.111650	4.108728	-0.009812
H	2.650964	0.158620	-1.815659	H	-0.752447	3.974022	-1.731694
H	-0.873618	2.143155	-2.323276	H	1.465847	4.303224	0.225160
H	0.673531	2.894930	-1.888153	H	1.933388	3.717820	-1.374101
C	1.588801	-0.088308	1.757319	C	1.684553	-0.869755	-1.537814
C	-2.071822	1.503886	0.944781	C	3.408434	-0.835436	0.351770
O	1.348419	-0.200325	2.895277	O	1.787801	-1.449969	-2.536335
O	-2.166235	2.564922	1.436142	O	4.442869	-1.347336	0.472998
H	-0.508350	2.473560	-0.629540	H	2.421609	2.804175	0.069934
H	1.478002	-0.469682	-2.960740	H	-1.767821	2.733995	-0.941206
C	0.895967	-3.692000	-0.353439	C	-4.096082	1.038204	1.705396
H	1.957232	-3.473719	-0.481413	H	-4.025361	1.232857	2.783885
H	0.743587	-4.118553	0.644346	H	-3.726550	1.909385	1.158879
H	0.590206	-4.459094	-1.072354	H	-5.158796	0.890785	1.470608
C	-1.397149	-2.498508	-0.345677	C	-3.714680	-1.500260	2.001448
H	-1.667703	-2.223571	0.698666	H	-4.769609	-1.718099	1.787390
H	-1.920763	-1.806058	-1.046413	H	-3.095491	-2.327823	1.647214
H	-1.834373	-3.485957	-0.493808	H	-3.613211	-1.420816	3.092075
C	0.078173	-2.461775	-0.516138	C	-3.309365	-0.200974	1.343785
C	0.558424	0.812662	-1.463633	C	0.334080	2.596822	-0.482580
N	-0.082268	-0.149801	-0.723572	N	-2.342494	-0.148338	0.524445
N	0.689215	-1.338442	-0.722601	N	0.318740	1.327811	-0.316733
Cr	-1.991423	-0.149258	0.182482	Cr	-0.889853	-0.181142	-0.420558
Cr	1.969843	0.117258	-0.035725	Cr	1.755984	0.003856	0.147235

Table S104. Optimized coordinates for the $(Me_2C{=}N)_2Cr_2(CO)_6$ structure **6T-7.**

	M06-L				B3PW91-D3		
	x	y	z		x	y	z
C	-0.153853	3.039922	-0.339018	C	-0.289927	2.992762	-0.393872
C	1.332358	0.890822	-0.776041	C	1.244200	1.013840	-0.847018
C	0.153853	-3.039922	-0.339018	C	0.289927	-2.992762	-0.393872
C	-1.332358	-0.890822	-0.776041	C	-1.244200	-1.013840	-0.847018
O	0.153853	4.156910	-0.195043	O	-0.013260	4.121666	-0.320944
O	2.498030	0.626584	-0.737188	O	2.430875	1.004363	-0.885741
O	-0.153853	-4.156910	-0.195043	O	0.013260	-4.121666	-0.320944
O	-2.498030	-0.626584	-0.737188	O	-2.430875	-1.004363	-0.885741
C	2.585631	-1.412976	2.079197	C	2.659498	-1.274701	2.125878
C	1.566890	0.565517	3.349680	C	1.553427	0.688899	3.352304
H	1.334687	0.093172	4.312334	H	1.324175	0.227238	4.322828
H	2.591858	0.938767	3.424377	H	2.572746	1.085203	3.421964
H	2.854026	-1.868494	3.037686	H	2.954029	-1.667365	3.106892
H	3.476772	-0.898047	1.703656	H	3.523059	-0.732859	1.718005
C	-0.710170	1.469483	-2.474483	C	-0.964977	1.449160	-2.390575
C	0.710170	-1.469483	-2.474483	C	0.964977	-1.449160	-2.390575
O	-0.865110	1.620533	-3.617908	O	-1.244200	1.640015	-3.500614
O	0.865110	-1.620533	-3.617908	O	1.244200	-1.640015	-3.500614
H	2.318494	-2.197592	1.363510	H	2.423757	-2.109085	1.456548
H	0.890563	1.409382	3.202830	H	0.860593	1.515939	3.180624
C	-2.585631	1.412976	2.079197	C	-2.659498	1.274701	2.125878
H	-2.318494	2.197592	1.363510	H	-2.423757	2.109085	1.456548
H	-3.476772	0.898047	1.703656	H	-3.523059	0.732859	1.718005
H	-2.854026	1.868494	3.037686	H	-2.954029	1.667365	3.106892
C	-1.566890	-0.565517	3.349680	C	-1.553427	-0.688899	3.352304
H	-2.591858	-0.938767	3.424377	H	-2.572746	-1.085203	3.421964
H	-0.890563	-1.409382	3.202830	H	-0.860593	-1.515939	3.180624
H	-1.334687	-0.093172	4.312334	H	-1.324175	-0.227238	4.322828
C	-1.473351	0.435088	2.248532	C	-1.495832	0.340630	2.264983
C	1.473351	-0.435088	2.248532	C	1.495832	-0.340630	2.264983
N	0.504086	-0.495026	1.386841	N	0.533396	-0.459407	1.405048
N	-0.504086	0.495026	1.386841	N	-0.533396	0.459407	1.405048
Cr	-0.448035	1.190516	-0.619932	Cr	-0.541546	1.153187	-0.585584
Cr	0.448035	-1.190516	-0.619932	Cr	0.541546	-1.153187	-0.585584

Table S105. Optimized coordinates for the $(Me_2C{=}N)_2Cr_2(CO)_6$ structure **6S-8.**

	M06-L				B3PW91-D3		
	x	y	z		x	y	z
C	3.131532	-0.017636	1.370013	C	3.742821	-0.525247	0.071753
C	2.791163	-1.482646	-0.641829	C	2.597438	1.802857	0.401453
C	-2.893287	-0.198865	1.438983	C	-3.029870	0.480005	-1.186751
C	-3.974711	1.225433	-0.418145	C	-3.649053	-0.595164	1.012369
O	3.423525	-0.257123	2.484417	O	4.824624	-0.942539	0.154568
O	2.852234	-2.650236	-0.787616	O	2.944214	2.876089	0.692956
O	-3.174175	-0.552300	2.520595	O	-3.634096	0.914789	-2.093201
O	-4.994164	1.784764	-0.525827	O	-4.664159	-0.860246	1.528329
C	-0.914688	-2.613575	-0.530282	C	-0.071949	2.279810	-1.352751
C	0.068494	-1.458333	1.508939	C	1.567157	0.521527	-2.306665
H	-0.560582	-2.099053	2.133860	H	1.837217	1.378360	-2.926284
H	1.055347	-1.935448	1.443824	H	2.569199	0.212482	-1.827706
H	-1.675109	-3.166176	0.027923	H	-0.880800	2.324433	-2.094911
H	-0.014076	-3.242918	-0.562136	H	0.695582	3.005175	-1.639428
C	4.419234	0.500993	-0.795858	C	1.838798	-0.165033	1.767110
C	-3.320586	-1.119592	-0.934413	C	-2.254061	1.489772	0.969147
O	5.548797	0.634014	-1.083503	O	1.756488	-0.332115	2.914724
O	-3.972033	-1.989523	-1.366469	O	-2.422092	2.533498	1.468486
H	-1.254860	-2.450057	-1.552513	H	-0.488539	2.563609	-0.384711
H	0.196986	-0.488608	1.995829	H	1.297350	-0.342597	-2.922313
C	1.429522	2.891893	0.060481	C	0.866219	-3.636912	-0.423540
H	2.428992	2.416960	0.072229	H	1.928197	-3.415608	-0.555451
H	1.436668	3.636834	-0.741856	H	0.724256	-4.128017	0.548659
H	1.350237	3.435248	1.007513	H	0.539870	-4.348499	-1.192951
C	-1.110279	2.341016	-0.083594	C	-1.428880	-2.435940	-0.292600
H	-1.613728	2.106406	-1.051935	H	-1.673583	-2.215031	0.769851
H	-1.661406	1.820849	0.751109	H	-1.978657	-1.730175	-0.973213
H	-1.273940	3.403855	0.091906	H	-1.859831	-3.422165	-0.482029
C	0.303585	1.925757	-0.100659	C	0.049582	-2.387985	-0.492592
C	-0.525136	-1.341597	0.145565	C	0.514231	0.905128	-1.315529
N	-0.424004	-0.215534	-0.581227	N	-0.103331	-0.066058	-0.574787
N	0.605289	0.662650	-0.227568	N	0.648358	-1.255903	-0.640852
Cr	-2.378412	0.283586	-0.240430	Cr	-2.070305	-0.143070	0.199940
Cr	2.645660	0.289682	-0.342993	Cr	2.011143	0.120530	-0.043496

Table S106. Harmonic vibrational frequencies (in cm^{-1}) and infrared intensities (in parentheses, in km/mol) for the $(Me_2C{=}N)_2Fe_2(CO)_8$ structure **2-8S-1**.

M06-L			B3PW91-D3		
30(2)	431(13)	1277(2)	35(3)	448(14)	1279(2)
46(0)	438(2)	1281(7)	45(0)	450(4)	1281(7)
53(0)	439(4)	1385(1)	50(0)	454(4)	1390(7)
58(0)	444(2)	1390(10)	61(0)	457(1)	1392(7)
64(1)	454(0)	1391(12)	70(1)	468(1)	1395(51)
69(0)	458(2)	1392(55)	71(0)	474(2)	1398(30)
70(0)	462(0)	1443(11)	79(1)	482(2)	1454(15)
80(0)	464(0)	1450(9)	80(0)	486(0)	1460(27)
83(1)	482(5)	1458(21)	90(0)	502(2)	1468(25)
86(1)	487(4)	1460(26)	92(0)	509(4)	1470(10)
87(0)	501(10)	1461(2)	92(0)	528(5)	1471(5)
91(1)	505(0)	1463(25)	103(0)	532(0)	1474(13)
99(0)	522(5)	1473(13)	103(1)	544(6)	1481(9)
102(1)	528(3)	1474(8)	108(1)	549(2)	1484(25)
106(0)	565(2)	1671(40)	109(0)	588(3)	1699(58)
107(0)	570(67)	1679(124)	114(1)	596(57)	1705(97)
112(2)	590(116)	1741(584)	122(3)	611(125)	1737(558)
136(1)	602(50)	1744(96)	139(0)	622(58)	1743(146)
145(1)	614(162)	2042(368)	147(0)	634(217)	2073(133)
167(0)	623(26)	2054(103)	150(1)	645(20)	2084(142)
171(1)	661(23)	2055(377)	168(1)	684(30)	2087(376)
190(0)	671(7)	2073(1398)	186(1)	697(8)	2099(1703)
195(0)	781(78)	2089(1586)	188(0)	802(66)	2115(1687)
203(0)	789(108)	2135(850)	203(1)	808(87)	2161(836)
208(1)	830(19)	3037(45)	206(0)	843(11)	3055(0)
224(0)	838(4)	3038(3)	225(1)	848(0)	3056(16)
288(0)	910(36)	3042(10)	297(1)	930(5)	3068(1)
297(0)	919(25)	3042(24)	306(1)	930(0)	3068(7)
300(2)	927(9)	3113(3)	314(2)	943(60)	3121(5)
304(4)	928(13)	3113(16)	318(3)	950(40)	3121(4)
305(0)	980(6)	3132(12)	323(3)	984(14)	3149(6)
332(0)	982(28)	3132(1)	341(0)	987(41)	3149(0)
390(4)	1080(5)	3156(8)	402(6)	1088(7)	3173(6)
396(1)	1081(3)	3157(46)	409(0)	1091(0)	3173(24)
419(2)	1098(6)	3196(0)	434(1)	1108(7)	3207(0)
420(1)	1100(1)	3196(6)	434(4)	1111(4)	3207(3)

Table S107. Harmonic vibrational frequencies (in cm^{-1}) and infrared intensities (in parentheses, in km/mol) for the $(Me_2C{=}N)_2Fe_2(CO)_8$ structure **2-8S-2**.

M06-L			B3PW91-D3		
36(2)	410(8)	1233(0)	35(2)	431(10)	1235(0)
39(1)	422(5)	1275(5)	42(0)	442(6)	1277(5)
50(0)	434(3)	1379(10)	51(0)	450(1)	1386(17)
54(1)	436(6)	1380(6)	56(1)	454(11)	1390(10)
60(1)	442(3)	1386(10)	62(1)	457(2)	1395(23)
65(1)	451(2)	1391(31)	69(1)	469(1)	1398(28)
68(1)	463(3)	1440(47)	72(0)	478(2)	1449(41)
73(0)	465(7)	1450(9)	77(0)	483(2)	1460(12)
79(0)	468(1)	1451(15)	83(0)	491(8)	1462(17)
83(0)	480(6)	1456(4)	90(0)	505(9)	1472(6)
88(0)	498(7)	1462(1)	96(1)	518(27)	1477(12)
90(0)	501(37)	1468(9)	98(0)	522(1)	1481(1)
92(0)	514(6)	1469(16)	99(0)	531(4)	1485(10)
102(1)	524(10)	1473(9)	105(1)	543(4)	1485(12)
107(0)	531(17)	1651(131)	115(0)	553(14)	1680(130)
119(2)	538(30)	1700(66)	128(3)	565(61)	1722(72)
123(1)	573(59)	1763(340)	135(0)	599(101)	1761(334)
132(0)	583(98)	1854(324)	142(0)	609(88)	1824(356)
157(0)	606(124)	2045(628)	164(0)	628(99)	2081(465)
163(0)	619(13)	2050(250)	173(0)	638(13)	2086(246)
173(2)	631(46)	2063(885)	179(0)	665(86)	2095(1053)
183(0)	645(63)	2067(921)	186(2)	667(49)	2100(844)
198(0)	675(79)	2095(1957)	208(1)	708(92)	2124(1988)
211(1)	708(28)	2128(313)	217(0)	721(73)	2156(274)
216(0)	792(138)	3038(26)	230(1)	809(114)	3055(10)
242(4)	804(4)	3044(34)	264(3)	828(2)	3063(1)
264(2)	846(4)	3045(11)	276(0)	844(3)	3066(11)
273(1)	898(40)	3052(26)	291(2)	921(34)	3070(9)
283(1)	920(0)	3113(11)	298(1)	924(1)	3122(5)
300(3)	934(39)	3129(11)	311(4)	946(74)	3137(6)
313(3)	958(4)	3137(14)	325(1)	957(3)	3145(8)
321(2)	977(4)	3138(13)	338(6)	985(18)	3150(9)
341(2)	1075(2)	3151(27)	361(4)	1082(4)	3164(14)
380(7)	1080(3)	3178(9)	407(8)	1086(3)	3191(3)
397(7)	1080(3)	3180(15)	418(3)	1090(3)	3195(8)
404(8)	1092(6)	3192(15)	419(9)	1103(9)	3200(11)

Table S107. Harmonic vibrational frequencies (in cm^{-1}) and infrared intensities (in parentheses, in km/mol) for the $(Me_2C{=}N)_2Fe_2(CO)_8$ structure **2-8S-3**.

M06-L			B3PW91-D3		
32(0)	418(0)	1296(1)	26(0)	437(6)	1298(1)
42(0)	425(5)	1298(0)	34(0)	438(0)	1300(1)
44(0)	429(0)	1381(12)	43(0)	444(0)	1393(15)
47(0)	434(7)	1386(7)	47(0)	447(4)	1396(15)
51(0)	436(2)	1397(27)	59(1)	448(1)	1402(36)
60(0)	452(0)	1399(29)	64(0)	462(1)	1406(30)
67(1)	464(13)	1440(17)	77(0)	484(8)	1449(10)
74(1)	472(18)	1443(5)	79(1)	495(20)	1454(23)
79(1)	476(13)	1446(9)	82(1)	499(16)	1456(13)
85(0)	480(10)	1456(6)	88(1)	503(1)	1466(2)
89(1)	482(1)	1458(4)	88(0)	505(4)	1472(4)
92(1)	488(2)	1462(7)	95(0)	506(4)	1475(2)
93(0)	488(1)	1473(10)	99(0)	509(8)	1484(9)
97(1)	495(1)	1477(13)	100(0)	514(4)	1488(14)
98(0)	508(4)	1657(8)	102(1)	522(2)	1684(19)
101(1)	538(3)	1662(39)	104(1)	561(3)	1690(34)
106(1)	564(1)	2006(559)	113(0)	580(1)	2029(327)
111(0)	584(2)	2009(677)	117(0)	599(1)	2031(1208)
118(0)	607(54)	2024(1590)	122(0)	627(60)	2046(1084)
127(0)	627(77)	2030(781)	127(0)	645(86)	2055(1230)
137(0)	628(117)	2046(1277)	133(0)	648(116)	2071(1525)
149(0)	632(69)	2051(201)	153(0)	649(72)	2075(76)
157(0)	645(60)	2111(477)	160(0)	669(65)	2134(386)
183(0)	671(85)	2124(300)	169(0)	685(84)	2146(308)
190(2)	737(3)	3035(16)	191(2)	745(4)	3055(2)
214(1)	834(0)	3038(22)	194(1)	843(0)	3059(5)
229(1)	862(2)	3040(17)	225(2)	862(1)	3060(13)
231(0)	936(5)	3049(16)	244(0)	942(5)	3069(5)
262(0)	939(1)	3117(5)	278(0)	949(0)	3128(3)
363(0)	957(0)	3119(9)	373(0)	973(1)	3130(3)
369(0)	978(3)	3126(8)	376(0)	984(3)	3138(3)
375(0)	989(3)	3137(11)	387(2)	1003(2)	3149(5)
380(2)	1076(10)	3170(14)	388(2)	1084(11)	3182(8)
386(2)	1082(1)	3171(1)	398(2)	1090(2)	3192(2)
397(6)	1085(1)	3173(14)	405(5)	1094(2)	3193(6)
413(0)	1112(11)	3196(2)	423(0)	1125(12)	3199(1)

Table S109. Harmonic vibrational frequencies (in cm^{-1}) and infrared intensities (in parentheses, in km/mol) for the $(Me_2C{=}N)_2Fe_2(CO)_8$ structure **2-8S-4**.

M06-L			B3PW91-D3		
26(1)	419(1)	1296(2)	5(1)	435(0)	1297(2)
37(0)	428(0)	1299(0)	29(0)	446(0)	1300(0)
44(1)	430(0)	1388(26)	37(1)	447(0)	1397(27)
59(0)	438(0)	1390(14)	52(0)	451(0)	1398(27)
62(0)	445(0)	1400(9)	56(0)	458(0)	1403(8)
66(0)	458(0)	1402(28)	59(0)	466(2)	1404(38)
73(0)	459(3)	1449(29)	72(0)	467(1)	1455(29)
79(1)	465(0)	1450(11)	78(0)	469(0)	1458(8)
83(0)	476(3)	1451(0)	80(1)	497(3)	1460(13)
85(0)	477(12)	1456(8)	83(0)	500(3)	1462(13)
92(1)	484(2)	1462(6)	91(1)	505(14)	1474(0)
94(0)	485(1)	1464(2)	97(0)	507(1)	1478(0)
97(0)	497(2)	1478(14)	99(0)	511(4)	1489(20)
105(1)	500(2)	1479(6)	103(0)	514(1)	1490(11)
108(2)	527(0)	1657(24)	105(0)	542(1)	1683(24)
113(1)	563(3)	1659(3)	111(2)	579(2)	1687(7)
123(0)	564(10)	2014(959)	118(0)	580(3)	2031(1611)
129(1)	584(2)	2020(28)	123(1)	598(1)	2040(178)
133(1)	601(13)	2026(1128)	126(1)	624(95)	2050(730)
139(0)	602(102)	2032(642)	136(0)	625(33)	2058(659)
144(0)	638(126)	2042(474)	146(1)	664(145)	2064(376)
155(0)	642(25)	2052(1892)	160(1)	668(33)	2079(1953)
168(0)	662(93)	2109(181)	162(0)	682(82)	2134(168)
186(1)	668(85)	2125(356)	189(0)	687(87)	2148(345)
202(1)	744(3)	3037(1)	200(1)	753(4)	3057(1)
215(0)	838(0)	3037(20)	224(0)	847(0)	3058(1)
225(0)	853(2)	3039(60)	229(1)	853(2)	3060(13)
231(0)	939(9)	3040(17)	246(0)	947(7)	3062(16)
242(1)	951(1)	3122(21)	262(1)	953(5)	3136(10)
365(1)	952(5)	3122(5)	372(2)	964(0)	3136(3)
367(1)	971(0)	3124(13)	373(0)	976(2)	3138(3)
371(1)	990(0)	3125(6)	382(2)	1000(1)	3139(10)
372(1)	1077(9)	3172(2)	385(1)	1083(14)	3179(0)
377(1)	1084(3)	3172(4)	395(1)	1090(0)	3181(0)
401(2)	1085(1)	3173(9)	404(2)	1094(0)	3185(3)
406(1)	1116(22)	3174(4)	405(2)	1127(21)	3192(3)

Table S110. Harmonic vibrational frequencies (in cm^{-1}) and infrared intensities (in parentheses, in km/mol) for the $(Me_2C{=}N)_2Fe_2(CO)_8$ structure **2-8S-5**.

M06-L			B3PW91-D3		
30(0)	374(4)	1285(6)	10(0)	385(3)	1280(9)
32(0)	378(0)	1294(2)	18(0)	396(3)	1288(0)
41(0)	390(2)	1386(18)	25(0)	402(2)	1388(21)
51(0)	393(8)	1386(21)	27(0)	410(3)	1388(37)
53(0)	397(6)	1404(31)	35(0)	427(17)	1404(54)
64(0)	412(10)	1409(19)	37(0)	439(14)	1406(0)
65(0)	438(3)	1448(4)	37(0)	442(1)	1460(9)
68(0)	441(3)	1454(4)	43(0)	456(1)	1460(1)
70(0)	447(6)	1457(20)	55(0)	461(0)	1462(26)
75(1)	448(6)	1460(17)	57(8)	465(7)	1467(11)
75(0)	456(6)	1461(28)	65(0)	465(6)	1469(12)
80(0)	462(0)	1466(18)	78(0)	479(5)	1470(18)
86(0)	484(0)	1475(2)	80(0)	496(0)	1481(10)
87(1)	495(0)	1478(4)	84(0)	509(3)	1483(15)
92(1)	513(3)	1681(27)	86(0)	512(1)	1717(76)
95(0)	517(1)	1699(65)	89(1)	539(0)	1718(12)
97(2)	544(100)	1892(707)	91(0)	551(2)	1894(851)
99(0)	549(7)	1921(66)	96(0)	563(2)	1936(118)
116(3)	552(2)	2048(292)	99(0)	573(110)	2082(199)
121(0)	559(93)	2051(303)	99(0)	581(114)	2083(167)
143(1)	581(42)	2058(1336)	109(2)	596(17)	2092(1338)
145(2)	586(28)	2065(1323)	122(0)	599(47)	2092(1571)
182(3)	592(42)	2089(1766)	133(4)	608(47)	2117(1825)
187(0)	611(483)	2132(67)	147(0)	644(568)	2161(17)
199(0)	671(4)	3033(33)	180(0)	672(7)	3052(20)
204(0)	833(1)	3033(8)	183(0)	825(0)	3052(0)
207(1)	835(1)	3035(33)	194(0)	829(1)	3057(18)
239(4)	929(5)	3036(32)	218(0)	931(4)	3058(15)
248(0)	948(1)	3110(12)	246(4)	945(1)	3118(3)
319(0)	962(0)	3111(20)	317(9)	959(0)	3118(17)
325(7)	970(4)	3119(12)	321(0)	966(3)	3131(10)
349(1)	988(4)	3121(15)	348(0)	993(4)	3132(7)
356(1)	1070(5)	3153(17)	358(4)	1070(10)	3169(12)
362(2)	1084(3)	3155(11)	363(1)	1091(1)	3170(0)
366(2)	1091(3)	3169(14)	372(0)	1092(10)	3180(4)
371(1)	1139(16)	3170(12)	374(2)	1149(23)	3180(11)

Table S111. Harmonic vibrational frequencies (in cm^{-1}) and infrared intensities (in parentheses, in km/mol) for the $(Me_2C{=}N)_2Fe_2(CO)_8$ structure **2-8S-6**.

M06-L				B3PW91-D3		
28(0)	386(1)	1288(3)		6(0)	379(5)	1285(8)
36(0)	403(20)	1298(1)		7(0)	408(3)	1295(2)
39(1)	406(1)	1383(13)		11(1)	424(30)	1393(21)
49(0)	411(19)	1386(10)		13(0)	426(3)	1395(17)
54(1)	416(1)	1399(4)		15(0)	435(2)	1402(26)
57(0)	424(1)	1400(49)		17(0)	440(0)	1403(34)
60(0)	425(6)	1442(29)		33(0)	443(4)	1453(22)
61(0)	427(5)	1446(5)		53(0)	450(7)	1457(6)
66(0)	465(11)	1447(1)		56(1)	467(1)	1459(11)
70(0)	470(24)	1454(7)		59(0)	486(16)	1463(13)
77(1)	473(15)	1455(5)		70(3)	498(7)	1468(10)
81(1)	479(7)	1460(3)		74(0)	499(31)	1472(5)
83(1)	487(0)	1472(3)		79(0)	504(15)	1480(2)
84(1)	493(6)	1474(15)		81(0)	506(3)	1482(24)
88(1)	496(19)	1660(16)		83(2)	511(7)	1686(19)
90(1)	505(35)	1698(62)		84(0)	514(46)	1727(66)
95(1)	509(2)	2003(432)		87(0)	521(0)	2025(1472)
97(1)	517(22)	2005(1175)		90(0)	534(2)	2032(551)
114(1)	525(9)	2025(1364)		92(0)	541(19)	2059(686)
121(1)	528(18)	2032(1371)		99(0)	547(47)	2061(989)
131(0)	578(1)	2042(486)		106(0)	587(0)	2079(973)
132(1)	627(66)	2054(878)		122(0)	645(72)	2085(1658)
147(0)	629(90)	2109(418)		130(0)	648(84)	2127(466)
153(0)	632(75)	2120(145)		143(0)	650(80)	2158(2)
172(1)	722(5)	3034(20)		161(1)	719(9)	3055(7)
184(1)	831(3)	3037(18)		173(0)	826(2)	3057(9)
192(1)	855(2)	3040(17)		190(1)	850(1)	3062(11)
206(1)	938(5)	3043(23)		196(0)	941(1)	3066(6)
239(1)	942(3)	3115(8)		232(1)	945(3)	3120(5)
314(1)	959(1)	3123(7)		315(0)	960(0)	3125(6)
315(1)	973(3)	3129(15)		321(0)	972(4)	3136(5)
321(0)	990(9)	3131(9)		321(0)	996(8)	3142(11)
338(5)	1075(7)	3151(20)		351(3)	1076(12)	3176(9)
358(1)	1082(1)	3165(16)		361(0)	1091(2)	3180(8)
361(1)	1086(1)	3173(9)		368(7)	1094(2)	3185(6)
377(1)	1126(12)	3189(2)		372(1)	1136(13)	3195(1)

Table S112. Harmonic vibrational frequencies (in cm^{-1}) and infrared intensities (in parentheses, in km/mol) for the $(Me_2C{=}N)_2Fe_2(CO)_8$ structure **2-8S-7**.

M06-L			B3PW91-D3		
30(1)	360(2)	1210(8)	4(0)	390(19)	1206(2)
31(0)	361(1)	1215(2)	6(0)	390(22)	1206(29)
35(1)	412(3)	1368(29)	6(0)	427(0)	1373(57)
39(0)	413(10)	1368(5)	10(0)	428(0)	1373(1)
39(0)	422(7)	1375(0)	11(0)	432(2)	1382(1)
54(0)	423(0)	1375(1)	14(0)	432(1)	1382(1)
59(0)	427(21)	1453(26)	27(1)	448(23)	1458(13)
59(1)	429(0)	1453(0)	28(0)	448(4)	1458(1)
65(0)	436(1)	1463(1)	42(0)	460(11)	1460(0)
69(0)	438(0)	1463(8)	43(0)	461(2)	1460(0)
73(0)	444(4)	1465(1)	65(0)	475(11)	1468(4)
77(0)	447(16)	1465(2)	66(0)	475(9)	1468(4)
79(0)	480(11)	1471(0)	70(0)	483(16)	1480(19)
83(0)	483(4)	1471(21)	70(0)	484(17)	1480(12)
90(1)	495(12)	1717(195)	71(1)	522(3)	1777(72)
92(0)	496(7)	1718(12)	72(1)	522(2)	1777(63)
96(0)	503(3)	2043(2)	85(0)	541(11)	2066(1960)
98(0)	505(10)	2043(1605)	85(0)	541(11)	2072(68)
101(1)	519(58)	2061(371)	88(0)	547(77)	2090(270)
108(1)	535(0)	2062(134)	88(0)	547(2)	2094(135)
112(0)	570(12)	2072(833)	95(0)	596(56)	2106(1514)
112(0)	571(45)	2087(1845)	95(0)	596(56)	2106(1851)
132(0)	592(163)	2120(455)	98(0)	630(99)	2153(459)
134(1)	593(39)	2133(24)	98(0)	633(20)	2156(303)
174(0)	609(117)	3028(84)	142(1)	638(89)	3050(0)
186(6)	616(0)	3028(12)	142(0)	638(128)	3050(14)
197(1)	829(6)	3034(88)	149(1)	836(9)	3055(50)
197(3)	829(0)	3034(1)	149(1)	837(8)	3055(45)
243(0)	919(0)	3111(3)	243(2)	921(0)	3122(0)
243(1)	919(0)	3111(32)	244(1)	921(1)	3122(0)
280(1)	947(1)	3129(7)	280(3)	922(0)	3126(23)
280(4)	947(7)	3129(26)	280(1)	922(0)	3126(21)
323(1)	1070(2)	3143(64)	347(6)	1085(54)	3176(5)
326(1)	1070(23)	3143(8)	348(0)	1085(46)	3176(18)
345(1)	1076(0)	3164(1)	350(0)	1085(4)	3182(17)
347(2)	1076(3)	3164(67)	350(0)	1085(12)	3182(7)

Table S113. Harmonic vibrational frequencies (in cm^{-1}) and infrared intensities (in parentheses, in km/mol) for the $(Me_2C{=}N)_2Fe_2(CO)_8$ structure **2-8S-8**.

M06-L			B3PW91-D3		
19(0)	370(8)	1206(3)	3(0)	389(22)	1206(13)
22(0)	381(13)	1213(28)	5(0)	391(25)	1208(18)
24(0)	417(4)	1357(28)	8(0)	428(0)	1374(31)
29(0)	421(1)	1365(2)	10(0)	428(0)	1374(21)
36(0)	422(2)	1370(15)	11(0)	432(2)	1382(1)
38(1)	424(2)	1374(18)	12(0)	433(1)	1384(1)
50(0)	435(10)	1444(2)	24(0)	447(16)	1458(7)
51(0)	439(8)	1447(5)	28(0)	449(14)	1459(3)
58(0)	444(2)	1451(10)	44(1)	461(7)	1460(4)
67(0)	446(10)	1453(3)	48(1)	462(6)	1462(2)
72(0)	455(24)	1455(4)	65(0)	475(12)	1468(7)
74(1)	458(13)	1461(7)	65(0)	477(11)	1470(4)
78(1)	469(19)	1467(12)	70(1)	483(19)	1479(16)
79(0)	471(17)	1475(27)	71(1)	484(17)	1481(11)
86(0)	500(10)	1739(37)	71(0)	521(3)	1772(50)
89(0)	503(2)	1750(33)	73(2)	522(3)	1777(76)
91(0)	515(12)	2040(727)	84(0)	540(12)	2069(997)
93(0)	524(17)	2046(739)	86(0)	543(11)	2072(997)
99(0)	538(39)	2061(1779)	88(0)	546(29)	2092(891)
104(0)	541(40)	2069(611)	95(0)	549(47)	2095(328)
108(1)	576(55)	2073(1146)	95(0)	596(65)	2103(1510)
117(0)	577(46)	2081(892)	98(0)	596(66)	2106(1451)
122(0)	611(119)	2128(399)	98(0)	632(56)	2152(347)
152(1)	619(90)	2133(311)	112(0)	633(54)	2155(320)
162(4)	626(43)	3020(26)	141(1)	638(154)	3047(5)
183(0)	627(51)	3026(47)	142(1)	640(87)	3052(8)
191(3)	841(3)	3032(25)	150(1)	837(8)	3054(33)
199(2)	842(11)	3035(65)	161(1)	837(10)	3056(38)
238(3)	918(0)	3112(5)	243(2)	921(1)	3120(2)
248(0)	923(0)	3113(41)	244(2)	922(0)	3124(0)
276(3)	929(1)	3115(15)	280(2)	922(0)	3126(16)
289(2)	930(1)	3119(22)	281(3)	926(1)	3128(19)
335(1)	1075(3)	3148(15)	348(3)	1084(38)	3174(9)
340(2)	1078(3)	3159(18)	348(3)	1085(0)	3178(12)
343(1)	1080(37)	3168(16)	350(0)	1087(64)	3180(12)
344(2)	1083(36)	3173(14)	352(0)	1088(20)	3182(15)

Table S114. Harmonic vibrational frequencies (in cm^{-1}) and infrared intensities (in parentheses, in km/mol) for the $(Me_2C{=}N)_2Fe_2(CO)_8$ structure **2-8S-9**.

M06-L			B3PW91-D3		
24(1)	399(29)	1215(6)	14(1)	392(67)	1208(9)
32(1)	410(4)	1246(1)	29(1)	409(17)	1239(0)
34(1)	416(18)	1366(12)	33(1)	417(2)	1375(19)
48(1)	424(9)	1377(5)	38(0)	431(3)	1384(3)
54(1)	431(6)	1382(7)	55(1)	434(3)	1388(21)
60(0)	440(1)	1387(9)	57(1)	443(3)	1394(10)
64(1)	456(5)	1445(3)	60(0)	461(7)	1457(3)
72(0)	460(15)	1448(5)	66(1)	475(7)	1458(9)
73(1)	466(12)	1449(7)	69(1)	483(8)	1458(8)
77(0)	479(6)	1452(6)	74(0)	489(2)	1459(6)
78(0)	481(6)	1460(14)	78(0)	495(7)	1469(13)
84(1)	489(29)	1462(7)	81(1)	496(33)	1470(7)
86(0)	511(6)	1466(10)	87(0)	504(7)	1479(9)
89(0)	517(7)	1472(9)	91(0)	526(8)	1482(11)
95(0)	527(35)	1677(599)	95(2)	538(13)	1732(616)
100(1)	537(7)	1723(83)	100(0)	542(30)	1735(151)
103(1)	555(12)	1747(39)	100(1)	568(63)	1761(75)
109(1)	562(49)	1855(530)	109(0)	574(43)	1842(577)
114(0)	576(40)	1899(339)	109(1)	590(44)	1886(322)
122(0)	596(126)	2047(468)	113(1)	599(120)	2073(581)
128(2)	625(92)	2051(791)	127(0)	632(93)	2081(819)
134(1)	632(55)	2069(648)	131(2)	648(31)	2105(1422)
139(2)	665(8)	2072(1480)	140(2)	668(3)	2106(546)
149(3)	681(14)	2120(751)	145(5)	697(7)	2144(797)
185(1)	781(6)	3027(23)	159(2)	806(14)	3052(7)
202(2)	845(8)	3036(46)	202(5)	841(5)	3057(4)
229(1)	856(2)	3037(18)	235(1)	846(1)	3060(25)
245(1)	918(0)	3038(59)	252(0)	923(0)	3061(34)
253(5)	925(1)	3118(3)	256(3)	926(0)	3123(1)
266(7)	934(4)	3119(24)	270(9)	927(1)	3127(11)
274(1)	937(1)	3126(8)	279(0)	930(1)	3130(2)
283(3)	939(0)	3131(12)	300(0)	936(4)	3134(9)
304(5)	1077(2)	3139(24)	304(6)	1083(26)	3168(8)
328(3)	1082(0)	3161(16)	336(8)	1085(3)	3183(8)
355(1)	1084(44)	3168(12)	352(1)	1087(39)	3185(10)
361(2)	1089(20)	3171(19)	364(1)	1090(1)	3190(9)

Table S115. Harmonic vibrational frequencies (in cm^{-1}) and infrared intensities (in parentheses, in km/mol) for the $(Me_2C{=}N)_2Fe_2(CO)_8$ structure **2-8S-10**.

M06-L			B3PW91-D3		
29(2)	431(13)	1277(2)	15(0)	365(2)	1233(1)
45(0)	438(2)	1281(7)	18(0)	430(23)	1241(0)
50(0)	439(4)	1385(1)	27(1)	433(25)	1386(9)
58(0)	444(2)	1389(10)	34(0)	450(4)	1387(34)
64(1)	454(0)	1391(13)	45(1)	455(1)	1391(4)
69(0)	458(2)	1391(54)	47(0)	458(7)	1392(11)
70(0)	462(0)	1443(12)	55(1)	463(1)	1455(8)
78(0)	464(0)	1451(8)	62(1)	467(8)	1461(18)
82(1)	482(5)	1458(23)	68(0)	485(4)	1461(5)
87(0)	487(4)	1461(25)	72(1)	499(3)	1464(13)
87(1)	501(9)	1461(2)	72(1)	506(8)	1470(3)
91(1)	505(0)	1464(23)	75(0)	516(9)	1475(4)
98(0)	522(5)	1473(13)	80(1)	530(10)	1480(10)
102(1)	528(3)	1473(8)	84(0)	541(3)	1489(12)
106(0)	565(2)	1671(40)	90(0)	551(3)	1745(96)
107(0)	570(67)	1679(124)	91(0)	564(18)	1755(46)
112(2)	590(115)	1741(584)	98(0)	566(18)	1763(269)
136(1)	602(51)	1744(96)	101(0)	596(51)	1904(954)
146(1)	614(162)	2042(363)	109(0)	599(43)	2069(79)
163(0)	623(26)	2054(93)	112(1)	611(68)	2082(785)
171(1)	661(23)	2055(388)	120(2)	623(57)	2097(733)
189(0)	671(8)	2073(1398)	127(1)	638(76)	2103(1564)
193(0)	781(78)	2089(1591)	135(2)	649(24)	2149(922)
203(0)	789(108)	2135(849)	151(1)	678(12)	2204(109)
207(1)	830(19)	3037(46)	162(2)	718(108)	3057(11)
224(0)	838(4)	3038(3)	169(3)	846(8)	3058(4)
289(0)	910(35)	3042(10)	181(0)	859(21)	3062(26)
297(0)	918(24)	3042(23)	207(3)	867(55)	3068(13)
300(2)	926(10)	3112(3)	256(2)	928(1)	3124(5)
304(4)	928(13)	3114(16)	268(2)	940(3)	3128(6)
305(0)	980(6)	3131(13)	276(0)	946(1)	3132(9)
332(0)	982(28)	3131(0)	294(0)	954(3)	3147(10)
390(4)	1080(5)	3157(7)	300(0)	1085(7)	3178(7)
396(1)	1081(3)	3157(47)	312(3)	1087(12)	3189(9)
419(2)	1098(6)	3196(0)	328(8)	1089(3)	3194(10)
420(1)	1100(1)	3197(6)	337(6)	1094(17)	3209(2)

Table S116. Harmonic vibrational frequencies (in cm^{-1}) and infrared intensities (in parentheses, in km/mol) for the $(Me_2C{=}N)_2Cr_2(CO)_{10}$ structure **2-10S-1**.

M06-L			B3PW91-D3		
19(1)	378(6)	1090(54)	12(1)	408(0)	1096(2)
35(0)	395(38)	1099(62)	32(0)	414(0)	1122(17)
42(0)	397(32)	1212(1)	43(0)	417(1)	1287(2)
56(0)	404(14)	1232(2)	47(1)	420(0)	1288(0)
58(0)	416(4)	1366(7)	58(1)	430(1)	1399(51)
60(1)	420(28)	1366(13)	60(0)	435(1)	1399(32)
68(0)	421(3)	1372(10)	69(1)	444(1)	1405(0)
69(0)	426(7)	1374(5)	71(1)	470(26)	1406(51)
74(0)	434(23)	1446(7)	72(0)	471(24)	1451(8)
78(0)	456(23)	1446(5)	73(1)	475(7)	1454(5)
80(1)	462(0)	1448(1)	78(0)	477(49)	1456(8)
84(0)	467(13)	1448(3)	85(0)	484(1)	1463(1)
86(1)	474(8)	1456(12)	89(1)	492(3)	1469(12)
90(0)	487(10)	1461(5)	94(0)	493(1)	1470(12)
94(2)	496(3)	1468(7)	94(1)	538(4)	1491(4)
100(0)	510(29)	1468(10)	97(0)	539(0)	1498(17)
100(1)	514(20)	1629(488)	101(1)	542(1)	1687(30)
102(1)	519(14)	1737(106)	105(1)	551(18)	1688(9)
108(0)	537(17)	1743(41)	108(0)	555(3)	2019(741)
113(0)	540(15)	1764(57)	113(1)	571(9)	2019(101)
113(2)	545(4)	1829(536)	118(0)	572(0)	2030(2619)
122(0)	553(19)	1872(332)	128(1)	575(3)	2035(1774)
114(1)	578(15)	1999(344)	138(0)	577(2)	2038(1426)
121(2)	588(21)	2063(1080)	151(0)	590(1)	2050(611)
146(0)	599(35)	2069(899)	161(0)	685(91)	2065(0)
155(0)	624(75)	2071(697)	165(0)	690(6)	2080(886)
161(0)	641(96)	2083(1700)	167(1)	691(213)	2141(249)
174(1)	651(174)	2133(640)	186(0)	692(132)	2151(259)
189(0)	654(30)	3035(18)	190(1)	717(209)	3062(1)
211(2)	662(21)	3037(98)	198(1)	722(51)	3062(2)
229(6)	793(7)	3040(45)	212(0)	744(10)	3066(6)
251(1)	857(11)	3044(64)	242(0)	832(1)	3067(14)
260(3)	872(3)	3126(9)	255(1)	852(0)	3130(2)
263(2)	928(1)	3127(19)	370(0)	931(4)	3130(1)
266(6)	932(0)	3128(9)	372(0)	946(2)	3147(6)
281(2)	941(0)	3130(16)	374(1)	960(4)	3148(0)
304(7)	943(1)	3166(18)	396(3)	961(2)	3201(7)
326(2)	989(6)	3168(18)	402(0)	988(1)	3201(0)
351(3)	1074(0)	3171(16)	404(7)	1079(10)	3224(4)
365(4)	1075(0)	3198(7)	406(1)	1091(2)	3224(2)

Table S117. Harmonic vibrational frequencies (in cm^{-1}) and infrared intensities (in parentheses, in km/mol) for the $(Me_2C{=}N)_2Cr_2(CO)_{10}$ structure **2-10S-2**.

M06-L			B3PW91-D3		
15(0)	373(0)	1085(49)	12(1)	408(0)	1096(2)
22(0)	380(47)	1086(21)	32(0)	414(0)	1122(17)
31(0)	381(0)	1208(2)	43(0)	417(1)	1287(2)
32(0)	382(0)	1209(15)	47(1)	420(0)	1288(0)
37(0)	404(2)	1368(9)	58(1)	430(1)	1399(51)
40(1)	414(3)	1371(14)	60(0)	435(1)	1399(32)
47(0)	417(20)	1375(5)	69(1)	444(1)	1405(0)
55(1)	427(11)	1378(13)	71(1)	470(26)	1406(51)
58(0)	429(32)	1454(4)	72(0)	471(24)	1451(8)
62(0)	430(19)	1454(5)	73(1)	475(7)	1454(5)
65(0)	430(28)	1459(10)	78(0)	477(49)	1456(8)
68(0)	440(4)	1462(2)	85(0)	484(1)	1463(1)
71(0)	447(13)	1466(4)	89(1)	492(3)	1469(12)
74(1)	466(10)	1476(12)	94(0)	493(1)	1470(12)
78(0)	494(1)	1477(9)	94(1)	538(4)	1491(4)
85(0)	509(2)	1490(16)	97(0)	539(0)	1498(17)
86(0)	512(0)	1749(129)	101(1)	542(1)	1687(30)
89(0)	518(0)	1755(8)	105(1)	551(18)	1688(9)
92(1)	519(0)	2028(2451)	108(0)	555(3)	2019(741)
94(0)	520(0)	2041(499)	113(1)	571(9)	2019(101)
97(0)	526(1)	2045(142)	118(0)	572(0)	2030(2619)
98(1)	527(1)	2050(1653)	128(1)	575(3)	2035(1774)
103(1)	529(1)	2055(2247)	138(0)	577(2)	2038(1426)
113(0)	536(2)	2057(1263)	151(0)	590(1)	2050(611)
121(0)	545(1)	2074(87)	161(0)	685(91)	2065(0)
130(1)	578(28)	2079(220)	165(0)	690(6)	2080(886)
136(0)	618(81)	2117(253)	167(1)	691(213)	2141(249)
150(1)	638(85)	2168(2)	186(0)	692(132)	2151(259)
156(0)	642(34)	3024(70)	190(1)	717(209)	3062(1)
191(1)	681(194)	3029(64)	198(1)	722(51)	3062(2)
220(1)	682(156)	3035(82)	212(0)	744(10)	3066(6)
229(2)	683(174)	3037(38)	242(0)	832(1)	3067(14)
252(5)	841(4)	3106(33)	255(1)	852(0)	3130(2)
272(0)	841(4)	3110(23)	370(0)	931(4)	3130(1)
285(7)	921(0)	3124(22)	372(0)	946(2)	3147(6)
295(3)	925(0)	3128(28)	374(1)	960(4)	3148(0)
355(0)	942(3)	3156(25)	396(3)	961(2)	3201(7)
356(0)	949(4)	3161(16)	402(0)	988(1)	3201(0)
358(0)	1074(1)	3165(26)	404(7)	1079(10)	3224(4)
365(1)	1077(1)	3179(17)	406(1)	1091(2)	3224(2)

Table S118. Harmonic vibrational frequencies (in cm^{-1}) and infrared intensities (in parentheses, in km/mol) for the $(Me_2C=N)_2Cr_2(CO)_{10}$ structure **2-10S-3**.

M06-L			B3PW91-D3		
15(0)	373(0)	1085(49)	9(0)	393(2)	1090(2)
22(0)	380(47)	1086(21)	19(0)	400(1)	1129(8)
31(0)	381(0)	1208(2)	23(1)	410(1)	1291(2)
32(0)	382(0)	1209(15)	34(1)	412(3)	1302(1)
37(0)	404(2)	1368(9)	39(1)	415(2)	1376(6)
40(1)	414(3)	1371(14)	45(0)	421(1)	1395(22)
47(0)	417(20)	1375(5)	51(0)	427(1)	1400(22)
55(1)	427(11)	1378(13)	60(1)	440(0)	1401(14)
58(0)	429(32)	1454(4)	62(0)	453(1)	1423(31)
62(0)	430(19)	1454(5)	71(0)	463(4)	1455(22)
65(0)	430(28)	1459(10)	73(0)	466(30)	1459(6)
68(0)	440(4)	1462(2)	74(0)	471(31)	1464(16)
71(0)	447(13)	1466(4)	77(0)	478(44)	1470(13)
74(1)	466(10)	1476(12)	77(0)	489(2)	1474(9)
78(0)	494(1)	1477(9)	81(0)	494(6)	1482(25)
85(0)	509(2)	1490(16)	84(0)	501(6)	1530(12)
86(0)	512(0)	1749(129)	86(2)	535(3)	1683(25)
89(0)	518(0)	1755(8)	91(0)	539(0)	1717(16)
92(1)	519(0)	2028(2451)	96(0)	549(3)	1998(1247)
94(0)	520(0)	2041(499)	99(1)	568(4)	2000(1087)
97(0)	526(1)	2045(142)	101(1)	571(4)	2017(919)
98(1)	527(1)	2050(1653)	105(2)	578(10)	2029(1340)
103(1)	529(1)	2055(2247)	107(0)	590(0)	2035(1889)
113(0)	536(2)	2057(1263)	112(1)	596(3)	2039(722)
121(0)	545(1)	2074(87)	116(0)	649(62)	2072(269)
130(1)	578(28)	2079(220)	123(0)	665(53)	2108(421)
136(0)	618(81)	2117(253)	128(0)	689(98)	2146(283)
150(1)	638(85)	2168(2)	131(0)	690(107)	2212(115)
156(0)	642(34)	3024(70)	135(0)	716(138)	2902(8)
191(1)	681(194)	3029(64)	137(1)	725(51)	3060(5)
220(1)	682(156)	3035(82)	153(0)	745(6)	3064(4)
229(2)	683(174)	3037(38)	174(1)	842(2)	3075(5)
252(5)	841(4)	3106(33)	179(1)	851(2)	3101(6)
272(0)	841(4)	3110(23)	188(0)	937(3)	3127(2)
285(7)	921(0)	3124(22)	202(1)	940(2)	3130(2)
295(3)	925(0)	3128(28)	250(0)	960(4)	3149(1)
355(0)	942(3)	3156(25)	362(3)	964(3)	3153(9)
356(0)	949(4)	3161(16)	368(0)	992(0)	3191(4)
358(0)	1074(1)	3165(26)	382(0)	1074(2)	3198(2)
365(1)	1077(1)	3179(17)	389(1)	1079(3)	3224(4)

Table S119. Harmonic vibrational frequencies (in cm^{-1}) and infrared intensities (in parentheses, in km/mol) for the $(Me_2C{=}N)_2Cr_2(CO)_{10}$ structure **2-10S-4**.

M06-L			B3PW91-D3		
17(1)	373(1)	1077(36)	7(1)	397(0)	1082(148)
26(1)	374(3)	1080(73)	8(0)	399(1)	1083(9)
31(1)	390(0)	1187(23)	10(0)	400(7)	1166(11)
36(0)	395(1)	1194(5)	11(0)	400(0)	1166(6)
39(0)	402(5)	1358(5)	15(0)	422(21)	1360(15)
42(0)	407(2)	1360(9)	18(0)	424(3)	1361(15)
47(1)	408(14)	1367(18)	21(0)	429(0)	1370(41)
52(1)	409(5)	1368(5)	25(0)	429(0)	1372(13)
58(0)	414(6)	1447(9)	29(0)	438(22)	1456(8)
60(2)	418(43)	1449(8)	30(0)	439(34)	1456(7)
65(0)	421(3)	1451(12)	49(0)	447(29)	1459(1)
67(0)	427(18)	1454(0)	51(1)	448(7)	1460(0)
73(0)	429(112)	1457(3)	52(0)	458(219)	1466(8)
74(0)	432(2)	1459(2)	53(0)	466(2)	1467(13)
78(1)	483(0)	1468(11)	70(0)	513(0)	1478(15)
81(0)	488(4)	1474(8)	71(0)	513(0)	1479(9)
84(1)	500(23)	1743(190)	76(0)	520(68)	1791(941)
87(0)	505(1)	1748(33)	76(0)	523(2)	1802(1)
89(0)	508(1)	2014(645)	87(0)	525(5)	2036(2508)
90(0)	510(53)	2016(1285)	88(0)	527(1)	2046(781)
92(1)	515(1)	2029(173)	89(1)	528(1)	2051(1071)
96(0)	520(16)	2031(714)	90(0)	529(2)	2058(660)
99(2)	536(149)	2037(392)	91(1)	536(1)	2066(734)
111(0)	542(0)	2051(1814)	91(0)	537(1)	2073(2043)
115(1)	543(20)	2061(365)	92(0)	545(2)	2089(156)
122(1)	569(3)	2067(1027)	94(0)	547(0)	2093(98)
134(3)	634(328)	2096(2535)	96(0)	667(36)	2120(4593)
142(1)	635(40)	2132(154)	96(0)	668(150)	2154(102)
144(3)	643(93)	3029(37)	148(0)	675(399)	3054(19)
166(29)	645(141)	3033(125)	150(0)	677(372)	3054(5)
176(1)	649(237)	3034(25)	152(0)	680(141)	3057(97)
182(1)	656(8)	3040(117)	154(0)	686(19)	3058(34)
230(1)	832(6)	3117(22)	243(139)	821(45)	3131(2)
240(2)	835(10)	3119(15)	248(3)	821(10)	3132(1)
254(59)	917(0)	3123(18)	249(3)	913(0)	3135(14)
265(2)	918(1)	3123(14)	255(0)	915(0)	3136(11)
344(0)	929(1)	3162(16)	354(0)	917(1)	3181(8)
345(0)	935(1)	3163(23)	355(0)	920(1)	3181(8)
364(29)	1070(7)	3171(20)	381(68)	1075(0)	3185(7)
369(2)	1073(0)	3207(6)	385(7)	1075(1)	3187(11)

Table S120. Harmonic vibrational frequencies (in cm^{-1}) and infrared intensities (in parentheses, in km/mol) for the $(Me_2C{=}N)_2Cr_2(CO)_{10}$ structure **2-10S-5**.

M06-L			B3PW91-D3		
5(0)	376(1)	1086(20)	4(0)	393(4)	1092(2)
5(0)	380(16)	1131(29)	6(0)	395(4)	1131(52)
11(0)	395(25)	1143(18)	12(0)	412(29)	1141(33)
20(1)	402(3)	1281(14)	21(1)	421(98)	1276(17)
27(2)	404(9)	1311(9)	32(3)	423(3)	1319(25)
37(0)	405(143)	1367(3)	36(3)	425(47)	1373(4)
39(1)	406(39)	1385(12)	41(0)	427(83)	1389(24)
39(2)	409(13)	1397(49)	42(6)	439(32)	1397(44)
46(0)	417(6)	1423(2)	57(1)	440(103)	1439(5)
52(0)	418(31)	1436(0)	59(0)	444(45)	1447(2)
55(1)	426(20)	1446(14)	60(1)	455(16)	1452(11)
58(1)	441(135)	1447(7)	64(0)	459(186)	1459(24)
58(0)	458(32)	1448(1)	65(0)	478(39)	1463(20)
67(0)	476(7)	1455(49)	71(0)	489(2)	1468(8)
69(0)	479(3)	1458(8)	75(0)	498(7)	1473(12)
70(0)	496(1)	1474(14)	76(0)	507(2)	1485(16)
73(0)	504(26)	1680(422)	80(1)	513(78)	1708(485)
78(0)	505(47)	1762(285)	82(7)	520(3)	1755(269)
80(4)	508(2)	1802(502)	86(1)	526(6)	1811(989)
81(3)	517(2)	2000(326)	86(2)	527(1)	2007(690)
82(0)	519(2)	2007(1513)	89(1)	535(1)	2025(1561)
88(1)	533(5)	2016(1364)	91(2)	554(4)	2036(1717)
89(1)	542(72)	2042(2577)	95(0)	562(30)	2066(5049)
93(4)	574(0)	2054(1904)	98(2)	592(0)	2082(617)
99(1)	590(68)	2065(411)	99(2)	627(87)	2084(1748)
110(2)	619(242)	2069(3184)	104(0)	647(505)	2090(1968)
118(1)	625(176)	2083(51)	117(1)	655(94)	2110(44)
127(0)	642(119)	2141(1497)	133(0)	670(144)	2161(1656)
165(14)	644(85)	3021(35)	164(28)	674(315)	3028(35)
184(1)	653(418)	3036(10)	175(2)	675(415)	3052(1)
214(0)	734(658)	3039(133)	188(1)	743(749)	3057(24)
229(0)	816(20)	3042(23)	238(0)	818(49)	3057(44)
241(1)	843(51)	3112(1)	253(15)	832(23)	3118(4)
264(45)	923(1)	3115(25)	268(43)	918(0)	3123(12)
287(17)	926(1)	3131(6)	277(59)	926(92)	3127(3)
292(25)	929(91)	3138(7)	295(20)	931(57)	3141(1)
343(0)	948(13)	3174(12)	353(0)	943(7)	3185(7)
364(52)	971(284)	3176(12)	379(97)	963(365)	3190(5)
365(0)	1066(3)	3181(7)	380(0)	1073(6)	3196(5)
373(0)	1085(1)	3194(1)	386(0)	1090(25)	3197(1)

Table S121. Harmonic vibrational frequencies (in cm^{-1}) and infrared intensities (in parentheses, in km/mol) for the $(Me_2C{=}N)_2Cr_2(CO)_{10}$ structure **2-10S-6**.

M06-L			B3PW91-D3		
24(1)	383(1)	1112(6)	23(0)	401(1)	1118(6)
42(0)	392(5)	1114(1)	38(1)	405(4)	1122(0)
54(1)	397(2)	1280(1)	43(0)	407(1)	1277(0)
60(0)	399(2)	1286(8)	54(1)	416(3)	1279(8)
67(0)	404(3)	1387(4)	56(1)	420(1)	1397(9)
73(0)	418(3)	1396(14)	62(1)	428(2)	1400(5)
79(0)	423(4)	1397(12)	64(0)	434(3)	1401(58)
82(0)	436(7)	1400(60)	76(0)	446(3)	1402(51)
87(0)	438(9)	1443(6)	80(1)	451(2)	1448(5)
88(0)	440(11)	1447(16)	86(0)	460(13)	1454(20)
92(0)	451(4)	1454(7)	93(0)	467(23)	1457(8)
95(0)	457(4)	1455(4)	97(0)	477(2)	1459(13)
97(0)	462(12)	1463(31)	99(0)	478(28)	1468(9)
101(0)	465(10)	1463(16)	102(0)	485(4)	1472(21)
103(0)	473(13)	1470(11)	104(1)	490(10)	1477(11)
107(0)	491(42)	1472(6)	110(0)	510(24)	1482(14)
114(0)	508(57)	1674(43)	112(0)	523(64)	1686(331)
122(0)	522(6)	1683(91)	115(0)	534(2)	1698(49)
126(0)	523(5)	1712(323)	120(1)	540(13)	1708(202)
129(1)	574(16)	1730(167)	127(2)	589(8)	1711(162)
137(1)	585(39)	1903(196)	129(0)	595(47)	1948(176)
145(0)	586(62)	2021(342)	132(1)	599(31)	2043(368)
147(1)	605(46)	2028(284)	137(1)	618(90)	2050(225)
150(1)	608(32)	2041(1031)	142(2)	626(26)	2062(1238)
158(1)	617(54)	2042(965)	148(1)	637(78)	2064(946)
163(0)	627(49)	2055(458)	155(1)	643(73)	2076(376)
169(1)	636(63)	2072(1761)	161(1)	657(77)	2089(1968)
171(1)	658(71)	2118(798)	164(4)	678(94)	2138(802)
183(1)	754(93)	3035(22)	172(1)	770(87)	3059(0)
192(1)	757(54)	3038(20)	182(2)	775(83)	3060(4)
210(3)	825(18)	3039(13)	192(9)	828(13)	3065(16)
219(8)	831(12)	3043(55)	206(4)	834(4)	3066(19)
251(2)	920(2)	3114(5)	257(3)	923(4)	3123(1)
260(4)	928(3)	3117(11)	266(3)	927(3)	3125(4)
261(3)	942(21)	3119(12)	282(3)	949(11)	3128(8)
280(2)	946(23)	3125(16)	296(1)	950(27)	3134(8)
299(2)	980(18)	3174(5)	302(1)	982(57)	3194(3)
306(1)	987(27)	3175(13)	313(2)	989(53)	3206(4)
354(13)	1077(4)	3184(4)	338(17)	1086(5)	3207(5)
373(22)	1079(3)	3189(7)	387(34)	1089(4)	3211(1)

Table S122. Harmonic vibrational frequencies (in cm^{-1}) and infrared intensities (in parentheses, in km/mol) for the $(Me_2C{=}N)_2Cr_2(CO)_{10}$ structure **2-10S-7**.

M06-L			B3PW91-D3		
20(1)	372(3)	1098(46)	6(1)	391(4)	1101(69)
30(1)	375(2)	1195(51)	11(1)	397(1)	1181(22)
36(1)	387(22)	1226(4)	19(1)	400(5)	1226(4)
41(1)	399(0)	1280(19)	29(0)	415(5)	1288(10)
43(1)	405(2)	1371(14)	34(2)	423(8)	1378(20)
52(0)	415(13)	1380(1)	43(2)	432(2)	1387(1)
54(1)	428(18)	1390(18)	46(0)	448(8)	1390(38)
65(1)	434(19)	1401(47)	54(0)	457(3)	1397(18)
68(1)	444(12)	1443(9)	62(0)	465(13)	1451(29)
74(1)	450(15)	1448(7)	64(1)	468(40)	1456(26)
80(1)	454(17)	1451(1)	67(0)	476(8)	1459(4)
82(1)	460(10)	1452(11)	78(0)	479(11)	1461(9)
86(1)	469(35)	1459(6)	82(0)	483(9)	1464(2)
89(0)	478(9)	1464(49)	87(0)	494(10)	1470(6)
91(0)	487(4)	1470(8)	89(0)	516(13)	1478(11)
93(0)	494(1)	1471(15)	93(1)	522(21)	1481(15)
98(1)	514(8)	1743(626)	99(1)	537(4)	1674(98)
101(1)	519(2)	1753(78)	105(1)	542(7)	1782(37)
102(1)	531(11)	1787(180)	108(0)	552(7)	1805(367)
106(1)	542(5)	1810(308)	109(0)	565(12)	1849(801)
116(0)	558(1)	1929(327)	110(0)	574(8)	1984(1938)
116(1)	567(7)	1997(400)	120(1)	592(4)	1998(592)
121(1)	586(8)	2010(606)	126(1)	612(56)	2010(704)
129(4)	607(89)	2027(444)	129(1)	637(65)	2037(23)
131(3)	623(24)	2034(955)	136(1)	645(49)	2040(952)
133(0)	630(31)	2042(1175)	144(2)	664(113)	2053(1783)
148(2)	639(36)	2072(1148)	151(0)	668(56)	2089(1293)
152(5)	655(69)	2106(1006)	168(1)	716(68)	2131(284)
155(1)	666(89)	3032(39)	170(1)	718(35)	3053(5)
158(0)	704(51)	3036(77)	174(2)	769(59)	3057(45)
164(1)	822(29)	3043(8)	183(7)	837(12)	3058(3)
180(3)	837(71)	3046(39)	189(6)	865(3)	3070(3)
198(2)	864(2)	3116(9)	198(3)	910(17)	3124(1)
217(2)	930(1)	3118(21)	251(1)	915(1)	3125(0)
258(2)	935(9)	3124(8)	273(1)	934(1)	3128(15)
271(4)	938(1)	3131(18)	278(4)	937(0)	3148(8)
272(0)	940(6)	3169(14)	291(4)	977(9)	3179(10)
287(0)	1033(119)	3173(13)	313(8)	1052(21)	3185(6)
308(3)	1077(0)	3175(19)	319(6)	1084(2)	3185(6)
351(3)	1089(1)	3183(15)	385(1)	1090(10)	3196(7)

Table S123. Harmonic vibrational frequencies (in cm^{-1}) and infrared intensities (in parentheses, in km/mol) for the $(Me_2C{=}N)_2Cr_2(CO)_{10}$ structure **2-10S-8**.

M06-L			B3PW91-D3		
24(1)	378(6)	1090(54)	20(1)	390(16)	1098(62)
30(0)	395(38)	1099(62)	25(1)	414(31)	1101(73)
33(1)	397(32)	1212(1)	30(0)	415(38)	1214(2)
41(1)	404(14)	1232(2)	40(1)	424(6)	1224(2)
42(1)	416(4)	1366(7)	42(1)	430(15)	1374(17)
50(1)	420(28)	1366(13)	47(1)	434(7)	1375(13)
57(1)	421(3)	1372(10)	52(2)	439(5)	1383(4)
64(1)	426(7)	1374(5)	62(1)	446(14)	1385(2)
66(0)	434(23)	1446(7)	66(0)	460(6)	1455(9)
69(0)	456(23)	1446(5)	68(0)	467(28)	1456(8)
71(0)	462(0)	1448(1)	71(0)	481(17)	1458(2)
75(0)	467(13)	1448(3)	72(0)	487(7)	1459(1)
78(0)	474(8)	1456(12)	80(0)	497(2)	1467(7)
81(0)	487(10)	1461(5)	80(0)	501(5)	1470(5)
87(1)	496(3)	1468(7)	81(0)	508(2)	1478(11)
88(0)	510(29)	1468(10)	85(0)	526(2)	1478(10)
93(1)	514(20)	1629(488)	89(0)	533(12)	1672(664)
95(1)	519(14)	1737(106)	90(0)	535(35)	1725(86)
95(0)	537(17)	1743(41)	94(1)	539(6)	1771(16)
101(1)	540(15)	1764(57)	97(1)	550(59)	1791(60)
103(0)	545(4)	1829(536)	99(0)	554(0)	1817(588)
113(0)	553(19)	1872(332)	106(0)	570(21)	1859(369)
114(1)	578(15)	1999(344)	112(0)	594(22)	2029(466)
121(2)	588(21)	2063(1080)	123(1)	610(33)	2091(1124)
146(0)	599(35)	2069(899)	139(0)	612(39)	2097(670)
155(0)	624(75)	2071(697)	141(0)	650(98)	2099(891)
161(0)	641(96)	2083(1700)	157(0)	662(52)	2113(1569)
174(1)	651(174)	2133(640)	181(1)	676(80)	2157(531)
189(0)	654(30)	3035(18)	195(1)	678(145)	3056(5)
211(2)	662(21)	3037(98)	211(7)	686(39)	3060(52)
229(6)	793(7)	3040(45)	240(9)	794(6)	3060(8)
251(1)	857(11)	3044(64)	254(0)	861(13)	3066(37)
260(3)	872(3)	3126(9)	264(4)	866(3)	3132(1)
263(2)	928(1)	3127(19)	267(2)	932(2)	3135(2)
266(6)	932(0)	3128(9)	272(7)	936(0)	3135(12)
281(2)	941(0)	3130(16)	286(2)	939(0)	3139(10)
304(7)	943(1)	3166(18)	299(12)	941(0)	3184(7)
326(2)	989(6)	3168(18)	327(1)	991(4)	3186(10)
351(3)	1074(0)	3171(16)	364(4)	1082(0)	3186(7)
365(4)	1075(0)	3198(7)	380(8)	1083(0)	3212(3)

Table S124. Harmonic vibrational frequencies (in cm^{-1}) and infrared intensities (in parentheses, in km/mol) for the $(Me_2C{=}N)_2Fe_2(CO)_7$ structure **2-7S-1**.

M06-L			B3PW91-D3		
24(2)	436(4)	1280(5)	17(2)	449(6)	1278(4)
37(0)	446(3)	1382(1)	38(0)	460(3)	1389(3)
42(0)	453(2)	1383(10)	39(1)	466(2)	1390(17)
54(1)	459(1)	1385(6)	51(0)	473(0)	1392(18)
57(0)	460(1)	1389(31)	56(0)	476(2)	1394(29)
64(0)	468(3)	1445(36)	67(0)	485(7)	1451(43)
69(0)	477(3)	1448(6)	73(0)	497(3)	1457(5)
78(0)	483(2)	1451(8)	78(0)	505(0)	1459(11)
82(0)	486(7)	1456(8)	84(0)	510(5)	1462(10)
86(0)	501(6)	1457(6)	88(0)	521(8)	1468(5)
87(0)	503(14)	1460(6)	91(0)	534(7)	1472(6)
89(0)	519(1)	1468(7)	93(0)	538(6)	1480(11)
91(0)	539(3)	1475(18)	96(1)	558(5)	1484(19)
94(0)	575(19)	1672(105)	99(0)	598(27)	1694(98)
103(0)	582(77)	1729(34)	100(0)	603(82)	1747(427)
107(1)	593(97)	1746(421)	107(0)	615(90)	1758(42)
114(1)	603(63)	2028(622)	113(1)	623(86)	2057(564)
122(1)	620(75)	2043(239)	123(1)	644(94)	2072(232)
140(1)	637(55)	2048(346)	132(2)	656(45)	2077(340)
159(0)	661(26)	2069(1277)	172(1)	686(34)	2097(1329)
168(0)	691(44)	2081(1527)	176(1)	713(62)	2103(1737)
169(1)	791(116)	2128(770)	180(1)	806(96)	2153(783)
202(1)	832(9)	3034(12)	205(1)	846(4)	3052(5)
222(0)	866(4)	3037(78)	229(1)	863(4)	3056(34)
271(1)	908(22)	3041(10)	278(1)	923(6)	3061(1)
281(1)	928(1)	3047(27)	292(1)	937(1)	3064(9)
301(0)	934(40)	3111(11)	314(0)	941(1)	3121(5)
313(1)	942(0)	3115(21)	324(1)	948(77)	3124(14)
319(2)	987(13)	3125(12)	332(4)	989(26)	3133(8)
342(1)	1081(2)	3134(19)	351(1)	1088(3)	3146(8)
397(2)	1083(1)	3173(18)	410(2)	1090(3)	3182(10)
415(3)	1090(25)	3175(11)	428(3)	1092(23)	3192(6)
419(2)	1100(8)	3181(11)	433(3)	1108(10)	3200(7)
432(3)	1242(1)	3195(6)	445(4)	1243(2)	3202(2)

Table S125. Harmonic vibrational frequencies (in cm^{-1}) and infrared intensities (in parentheses, in km/mol) for the $(Me_2C{=}N)_2Fe_2(CO)_7$ structure **2-7S-2**.

M06-L			B3PW91-D3		
33(0)	423(0)	1301(1)	17(2)	449(6)	1278(4)
38(0)	428(15)	1389(11)	38(0)	460(3)	1389(3)
49(0)	434(6)	1392(12)	39(1)	466(2)	1390(17)
53(1)	446(9)	1397(16)	51(0)	473(0)	1392(18)
60(1)	452(1)	1403(27)	56(0)	476(2)	1394(29)
66(0)	463(8)	1444(37)	67(0)	485(7)	1451(43)
70(0)	470(40)	1450(15)	73(0)	497(3)	1457(5)
76(0)	475(8)	1451(10)	78(0)	505(0)	1459(11)
82(1)	476(46)	1455(1)	84(0)	510(5)	1462(10)
85(0)	485(10)	1458(18)	88(0)	521(8)	1468(5)
91(1)	501(6)	1462(3)	91(0)	534(7)	1472(6)
94(0)	512(6)	1475(21)	93(0)	538(6)	1480(11)
98(0)	520(14)	1477(8)	96(1)	558(5)	1484(19)
100(2)	535(52)	1655(11)	99(0)	598(27)	1694(98)
116(1)	564(75)	1673(27)	100(0)	603(82)	1747(427)
125(0)	580(6)	1880(477)	107(0)	615(90)	1758(42)
137(0)	606(75)	2003(194)	113(1)	623(86)	2057(564)
143(0)	609(67)	2021(1125)	123(1)	644(94)	2072(232)
153(1)	619(91)	2026(749)	132(2)	656(45)	2077(340)
177(2)	633(65)	2044(894)	172(1)	686(34)	2097(1329)
203(0)	643(111)	2062(1265)	176(1)	713(62)	2103(1737)
213(1)	765(4)	2111(768)	180(1)	806(96)	2153(783)
217(1)	842(1)	3036(10)	205(1)	846(4)	3052(5)
224(1)	867(5)	3038(54)	229(1)	863(4)	3056(34)
234(1)	944(2)	3042(5)	278(1)	923(6)	3061(1)
260(2)	952(4)	3045(16)	292(1)	937(1)	3064(9)
266(0)	960(2)	3120(10)	314(0)	941(1)	3121(5)
368(1)	972(1)	3121(12)	324(1)	948(77)	3124(14)
373(1)	998(1)	3125(7)	332(4)	989(26)	3133(8)
379(1)	1079(10)	3135(17)	351(1)	1088(3)	3146(8)
385(20)	1082(2)	3171(6)	410(2)	1090(3)	3182(10)
391(8)	1084(5)	3173(10)	428(3)	1092(23)	3192(6)
410(3)	1133(22)	3174(5)	433(3)	1108(10)	3200(7)
422(7)	1299(3)	3180(8)	445(4)	1243(2)	3202(2)

Table S126. Harmonic vibrational frequencies (in cm^{-1}) and infrared intensities (in parentheses, in km/mol) for the $(Me_2C{=}N)_2Fe_2(CO)_7$ structure **2-7T-3**.

M06-L			B3PW91-D3		
30(2)	368(15)	1231(0)	28(0)	429(2)	1294(2)
38(0)	373(9)	1374(10)	32(1)	434(1)	1386(1)
41(0)	394(5)	1378(3)	37(0)	440(1)	1394(9)
55(1)	405(7)	1378(9)	50(0)	450(4)	1397(41)
58(0)	418(7)	1383(11)	53(0)	476(4)	1405(31)
63(0)	426(13)	1450(11)	61(0)	479(5)	1414(19)
67(0)	433(2)	1451(3)	70(0)	484(1)	1446(8)
70(0)	443(2)	1451(11)	75(1)	489(10)	1452(25)
71(1)	451(3)	1454(8)	82(0)	496(21)	1453(8)
79(1)	457(9)	1462(6)	86(1)	504(2)	1465(2)
84(0)	469(1)	1463(7)	93(1)	508(7)	1469(12)
86(1)	474(6)	1471(4)	94(0)	518(2)	1483(16)
89(1)	476(6)	1473(4)	97(2)	526(3)	1559(12)
95(0)	494(1)	1706(124)	98(2)	569(1)	1681(36)
100(0)	512(1)	1715(40)	103(1)	577(14)	1694(27)
102(0)	529(14)	2021(148)	104(1)	590(8)	2006(1048)
112(3)	556(112)	2036(370)	116(0)	612(34)	2026(275)
117(1)	564(62)	2040(957)	124(0)	641(70)	2030(1209)
130(0)	576(89)	2045(1165)	126(2)	645(100)	2051(1428)
145(1)	582(27)	2060(827)	135(1)	646(72)	2070(481)
156(0)	606(44)	2084(1695)	147(0)	648(18)	2091(692)
160(0)	626(3)	2114(231)	160(0)	705(1)	2135(444)
177(0)	685(23)	3031(17)	167(1)	842(2)	2808(6)
186(1)	837(4)	3034(85)	203(2)	864(1)	3057(5)
197(0)	848(2)	3036(17)	213(1)	930(2)	3059(5)
234(2)	922(1)	3039(51)	258(1)	938(1)	3069(8)
239(2)	925(1)	3113(4)	303(1)	954(2)	3074(3)
271(1)	927(1)	3114(30)	352(2)	974(3)	3126(2)
288(4)	941(1)	3117(9)	365(2)	998(2)	3127(5)
290(4)	1075(19)	3118(23)	371(1)	1065(11)	3148(3)
314(1)	1079(1)	3170(13)	376(0)	1089(0)	3163(0)
331(9)	1079(0)	3171(26)	383(1)	1093(4)	3180(9)
349(1)	1080(6)	3179(12)	407(2)	1112(5)	3193(2)
364(14)	1216(1)	3183(18)	416(0)	1280(4)	3200(1)

Table S127. Harmonic vibrational frequencies (in cm^{-1}) and infrared intensities (in parentheses, in km/mol) for the $(Me_2C=N)_2Fe_2(CO)_7$ structure **2-7T-4**.

M06-L			B3PW91-D3		
24(0)	392(20)	1300(1)	20(0)	399(7)	1296(1)
28(0)	397(30)	1387(23)	21(0)	408(44)	1394(25)
35(0)	412(0)	1389(10)	31(0)	421(37)	1394(24)
51(0)	415(6)	1402(15)	44(0)	426(0)	1405(21)
63(1)	425(3)	1406(43)	50(1)	438(3)	1406(42)
64(0)	443(5)	1445(2)	64(1)	460(8)	1457(11)
69(0)	453(6)	1450(20)	68(1)	461(2)	1457(12)
73(0)	459(2)	1452(4)	73(0)	466(8)	1460(3)
76(0)	462(4)	1453(7)	75(1)	474(0)	1464(3)
80(0)	482(3)	1457(17)	77(1)	514(4)	1466(13)
84(1)	504(23)	1461(18)	81(0)	518(12)	1469(29)
89(1)	510(63)	1469(21)	87(0)	525(4)	1479(0)
91(0)	514(3)	1474(1)	89(1)	530(42)	1481(23)
94(0)	516(0)	1665(12)	93(1)	535(8)	1687(14)
103(2)	532(28)	1706(69)	96(0)	543(0)	1728(69)
107(1)	554(102)	1998(173)	100(1)	562(112)	2021(423)
120(0)	565(25)	2016(346)	110(0)	578(28)	2029(256)
121(3)	573(80)	2023(589)	114(0)	580(128)	2051(284)
131(0)	575(17)	2030(1114)	115(3)	584(24)	2053(1532)
139(2)	577(137)	2046(1242)	125(0)	600(75)	2065(1269)
141(1)	590(33)	2062(1656)	139(4)	610(123)	2084(1722)
149(0)	722(10)	2114(337)	142(2)	723(11)	2135(289)
162(0)	832(1)	3036(14)	164(0)	826(2)	3057(3)
178(0)	853(3)	3038(11)	169(0)	849(2)	3058(6)
185(1)	934(5)	3039(28)	184(1)	935(2)	3062(12)
236(0)	944(1)	3041(40)	199(0)	944(1)	3063(14)
243(1)	954(2)	3114(10)	235(1)	954(2)	3122(4)
338(1)	965(2)	3120(14)	340(1)	967(2)	3127(5)
348(3)	992(8)	3120(10)	344(1)	996(7)	3133(8)
353(1)	1075(6)	3125(13)	349(3)	1076(9)	3134(5)
356(1)	1081(0)	3164(18)	366(0)	1087(1)	3172(8)
366(0)	1091(1)	3165(11)	371(2)	1096(2)	3185(6)
370(2)	1125(15)	3174(13)	374(3)	1135(19)	3186(5)
376(5)	1290(9)	3179(10)	380(3)	1285(10)	3195(6)

Table S128. Harmonic vibrational frequencies (in cm^{-1}) and infrared intensities (in parentheses, in km/mol) for the $(Me_2C{=}N)_2Fe_2(CO)_7$ structure **2-7T-5**.

M06-L			B3PW91-D3		
18(0)	406(1)	1301(1)	15(0)	415(1)	1299(1)
28(0)	413(1)	1380(10)	29(1)	429(2)	1390(15)
34(1)	416(0)	1385(9)	35(0)	432(3)	1395(12)
47(0)	425(3)	1394(26)	40(0)	437(3)	1400(34)
49(0)	431(4)	1400(38)	47(0)	439(2)	1403(40)
51(0)	441(16)	1440(10)	53(0)	468(20)	1449(13)
59(0)	445(25)	1441(5)	60(0)	471(22)	1453(9)
65(1)	463(15)	1446(4)	65(1)	474(4)	1455(10)
69(1)	470(15)	1448(8)	72(1)	485(16)	1459(4)
73(1)	473(12)	1455(2)	74(0)	497(23)	1467(2)
76(0)	485(0)	1460(6)	79(2)	503(1)	1473(8)
82(1)	491(2)	1470(5)	84(2)	511(9)	1477(2)
84(2)	503(3)	1472(11)	87(1)	515(0)	1481(13)
93(0)	521(14)	1656(15)	89(0)	535(9)	1677(21)
98(0)	525(2)	1663(42)	97(1)	536(2)	1683(55)
105(1)	534(6)	1986(484)	101(0)	547(3)	2012(935)
107(1)	539(8)	1999(901)	104(1)	551(6)	2021(379)
114(0)	580(2)	2010(1412)	114(0)	590(1)	2031(1409)
121(1)	625(72)	2023(1186)	124(1)	642(68)	2050(1523)
138(1)	628(99)	2049(487)	130(0)	645(96)	2071(530)
139(0)	631(67)	2078(581)	139(1)	646(72)	2102(532)
147(0)	743(3)	2114(499)	143(0)	739(2)	2134(457)
156(1)	844(1)	3034(15)	157(0)	844(0)	3049(9)
188(0)	855(4)	3035(7)	187(1)	849(2)	3052(4)
194(1)	931(4)	3036(24)	195(1)	936(4)	3057(8)
230(0)	939(6)	3037(23)	224(0)	941(4)	3066(4)
252(0)	961(1)	3116(2)	250(1)	969(1)	3122(2)
316(0)	980(5)	3117(9)	312(0)	978(6)	3124(2)
352(1)	998(3)	3124(5)	340(1)	1005(2)	3130(1)
355(0)	1078(7)	3126(11)	343(0)	1078(7)	3147(5)
359(1)	1080(3)	3155(5)	363(2)	1089(4)	3160(2)
368(3)	1085(1)	3169(12)	370(2)	1093(0)	3177(6)
381(1)	1115(11)	3171(16)	376(1)	1123(12)	3183(7)
388(2)	1299(1)	3190(2)	387(4)	1296(2)	3199(1)

Table S129. Harmonic vibrational frequencies (in cm^{-1}) and infrared intensities (in parentheses, in km/mol) for the $(Me_2C{=}N)_2Fe_2(CO)_7$ structure **2-7S-6**.

M06-L			B3PW91-D3		
41(1)	433(6)	1245(0)	40(0)	444(8)	1240(0)
44(0)	439(4)	1377(9)	43(0)	451(3)	1384(1)
54(0)	440(0)	1380(8)	58(0)	454(4)	1385(35)
56(1)	447(6)	1383(7)	61(0)	456(3)	1390(8)
61(1)	453(4)	1385(11)	65(2)	468(10)	1392(9)
68(0)	458(5)	1449(5)	72(0)	471(3)	1458(9)
70(0)	472(2)	1451(6)	73(0)	487(1)	1460(3)
77(0)	480(4)	1452(9)	81(0)	497(7)	1464(18)
80(1)	488(0)	1456(10)	86(0)	510(1)	1468(9)
85(0)	501(8)	1459(5)	90(0)	517(3)	1470(10)
89(0)	506(21)	1461(8)	94(0)	531(12)	1471(3)
92(0)	520(4)	1469(6)	100(0)	543(1)	1480(10)
96(0)	535(20)	1470(5)	102(0)	567(35)	1484(8)
99(0)	556(34)	1720(147)	104(0)	574(34)	1717(333)
103(0)	575(4)	1725(113)	111(0)	608(64)	1742(105)
112(0)	591(106)	1733(177)	120(1)	617(30)	1750(65)
122(1)	604(41)	1837(634)	122(1)	623(63)	1829(558)
137(1)	610(82)	2038(658)	131(0)	631(81)	2064(722)
158(1)	637(16)	2068(678)	160(0)	660(17)	2104(605)
166(4)	661(8)	2079(436)	172(1)	669(19)	2110(992)
173(2)	674(3)	2083(1619)	178(1)	704(1)	2114(985)
179(0)	712(52)	2129(515)	187(0)	729(87)	2157(545)
221(0)	795(21)	3031(35)	215(0)	812(17)	3053(13)
227(1)	857(6)	3036(26)	238(5)	850(4)	3056(9)
241(3)	864(2)	3038(49)	251(1)	856(2)	3061(23)
263(0)	930(1)	3041(36)	262(1)	927(1)	3067(12)
277(4)	931(3)	3117(6)	283(1)	932(1)	3123(3)
281(8)	939(0)	3118(8)	297(7)	936(0)	3124(5)
291(0)	958(3)	3119(27)	298(2)	959(4)	3128(10)
302(1)	1080(0)	3134(15)	311(0)	1086(2)	3150(9)
337(7)	1080(0)	3158(18)	346(9)	1087(18)	3172(9)
359(3)	1086(24)	3165(9)	376(2)	1088(6)	3182(8)
379(8)	1092(16)	3167(24)	391(2)	1092(14)	3186(15)
429(3)	1240(0)	3172(20)	439(7)	1233(1)	3187(13)

Table S130. Harmonic vibrational frequencies (in cm^{-1}) and infrared intensities (in parentheses, in km/mol) for the $(Me_2C{=}N)_2Fe_2(CO)_7$ structure **2-7S-7**.

M06-L			B3PW91-D3		
30(0)	419(2)	1216(13)	19(0)	430(3)	1212(12)
41(0)	424(1)	1371(17)	31(0)	434(3)	1374(26)
45(0)	425(2)	1376(4)	35(1)	436(2)	1382(3)
46(1)	429(4)	1380(21)	46(1)	442(3)	1383(27)
57(0)	445(14)	1385(17)	57(0)	455(17)	1393(20)
64(0)	458(5)	1423(70)	59(0)	470(11)	1439(110)
75(0)	469(7)	1450(1)	73(0)	481(0)	1458(6)
77(0)	470(2)	1453(5)	76(0)	489(5)	1461(2)
83(0)	480(5)	1458(3)	81(1)	498(5)	1462(2)
86(0)	483(3)	1458(7)	82(0)	502(13)	1470(16)
95(0)	502(16)	1467(15)	88(0)	517(14)	1470(8)
99(1)	509(9)	1469(4)	92(0)	522(7)	1481(15)
100(1)	517(3)	1479(10)	97(1)	530(2)	1483(5)
105(0)	524(11)	1497(17)	100(0)	537(18)	1508(32)
108(0)	548(8)	1745(48)	104(1)	555(7)	1761(79)
115(2)	558(18)	2024(596)	108(0)	580(9)	2050(697)
122(1)	582(28)	2040(215)	115(1)	596(32)	2070(267)
137(6)	598(75)	2056(691)	118(1)	615(116)	2082(510)
150(0)	605(131)	2068(1386)	142(8)	615(90)	2094(1713)
159(0)	611(20)	2071(866)	152(2)	623(24)	2102(690)
172(6)	636(94)	2110(1318)	164(1)	654(123)	2132(1322)
190(0)	641(122)	2132(51)	171(3)	661(120)	2154(80)
208(1)	676(23)	3011(59)	190(1)	691(44)	3037(29)
212(22)	830(7)	3022(50)	209(1)	828(7)	3048(30)
229(1)	848(1)	3033(20)	227(11)	843(1)	3053(9)
251(10)	928(0)	3036(74)	252(21)	923(0)	3058(40)
259(6)	929(1)	3103(20)	268(5)	923(0)	3120(7)
273(4)	934(0)	3108(19)	272(7)	931(0)	3126(0)
306(1)	966(1)	3118(13)	311(2)	970(1)	3127(15)
356(16)	1057(35)	3124(16)	374(17)	1063(4)	3130(16)
377(1)	1062(14)	3165(12)	388(1)	1068(59)	3179(8)
391(1)	1081(0)	3168(19)	405(3)	1086(1)	3182(9)
396(5)	1088(27)	3169(14)	406(6)	1089(29)	3182(13)
416(9)	1203(6)	3170(19)	423(11)	1193(6)	3185(10)

Table S131. Harmonic vibrational frequencies (in cm^{-1}) and infrared intensities (in parentheses, in km/mol) for the $(Me_2C{=}N)_2Fe_2(CO)_7$ structure **2-7T-8**.

M06-L			B3PW91-D3		
-1(0)	282(1)	1241(0)	10(0)	352(0)	1234(0)
26(0)	295(7)	1380(3)	13(0)	379(1)	1386(0)
35(0)	324(2)	1380(5)	31(0)	402(0)	1387(36)
41(2)	331(6)	1381(0)	31(0)	422(5)	1389(21)
46(1)	343(11)	1381(34)	34(1)	424(8)	1389(5)
50(0)	405(2)	1446(2)	43(0)	437(11)	1456(10)
52(0)	421(2)	1448(3)	48(0)	455(6)	1457(6)
55(0)	431(7)	1450(4)	50(0)	457(7)	1460(5)
62(1)	445(3)	1452(5)	60(1)	463(1)	1460(12)
63(0)	446(0)	1459(29)	63(1)	487(1)	1467(19)
67(0)	459(0)	1461(12)	66(0)	490(4)	1468(6)
68(0)	463(5)	1468(4)	69(0)	490(6)	1482(2)
76(0)	469(3)	1469(10)	71(0)	494(2)	1482(6)
79(0)	479(13)	1703(182)	80(0)	523(3)	1707(256)
81(0)	481(3)	1714(57)	83(0)	539(1)	1715(53)
84(0)	482(10)	2002(777)	89(0)	553(0)	2053(87)
87(0)	516(4)	2032(670)	93(0)	570(61)	2059(1492)
89(1)	516(3)	2042(140)	96(0)	579(41)	2060(214)
100(1)	556(43)	2063(1262)	97(0)	589(15)	2068(1493)
102(1)	587(58)	2074(1240)	109(0)	617(35)	2112(1241)
104(1)	621(62)	2110(551)	132(0)	630(32)	2131(263)
122(1)	645(0)	2173(183)	133(0)	671(0)	2203(121)
127(3)	669(52)	3029(33)	142(0)	702(4)	3054(2)
132(2)	854(1)	3029(29)	150(1)	843(1)	3054(6)
172(1)	862(1)	3036(73)	152(0)	850(0)	3059(45)
185(0)	918(0)	3036(41)	173(0)	923(0)	3060(12)
192(2)	919(7)	3115(1)	184(0)	923(0)	3121(3)
202(3)	938(0)	3115(1)	253(0)	927(2)	3121(2)
222(4)	938(0)	3116(20)	268(2)	928(1)	3125(5)
228(3)	1083(1)	3117(39)	277(0)	1080(14)	3125(19)
234(0)	1084(2)	3155(18)	291(0)	1086(4)	3192(7)
263(3)	1085(22)	3155(17)	315(4)	1088(1)	3192(16)
264(1)	1090(8)	3174(14)	327(10)	1088(0)	3193(9)
279(2)	1238(0)	3174(14)	350(0)	1232(0)	3193(9)

Table S132. Harmonic vibrational frequencies (in cm^{-1}) and infrared intensities (in parentheses, in km/mol) for the $(Me_2C{=}N)_2Fe_2(CO)_7$ structure **2-7S-9**.

M06-L			B3PW91-D3		
30(0)	419(2)	1216(13)	28(0)	429(2)	1294(2)
41(0)	424(1)	1371(17)	32(1)	434(1)	1386(1)
45(0)	425(2)	1376(4)	37(0)	440(1)	1394(9)
46(1)	429(4)	1380(21)	50(0)	450(4)	1397(41)
57(0)	445(14)	1385(17)	53(0)	476(4)	1405(31)
64(0)	458(5)	1423(70)	61(0)	479(5)	1414(19)
75(0)	469(7)	1450(1)	70(0)	484(1)	1446(8)
77(0)	470(2)	1453(5)	75(1)	489(10)	1452(25)
83(0)	480(5)	1458(3)	82(0)	496(21)	1453(8)
86(0)	483(3)	1458(7)	86(1)	504(2)	1465(2)
95(0)	502(16)	1467(15)	93(1)	508(7)	1469(12)
99(1)	509(9)	1469(4)	94(0)	518(2)	1483(16)
100(1)	517(3)	1479(10)	97(2)	526(3)	1559(12)
105(0)	524(11)	1497(17)	98(2)	569(1)	1681(36)
108(0)	548(8)	1745(48)	103(1)	577(14)	1694(27)
115(2)	558(18)	2024(596)	104(1)	590(8)	2006(1048)
122(1)	582(28)	2040(215)	116(0)	612(34)	2026(275)
137(6)	598(75)	2056(691)	124(0)	641(70)	2030(1209)
150(0)	605(131)	2068(1386)	126(2)	645(100)	2051(1428)
159(0)	611(20)	2071(866)	135(1)	646(72)	2070(481)
172(6)	636(94)	2110(1318)	147(0)	648(18)	2091(692)
190(0)	641(122)	2132(51)	160(0)	705(1)	2135(444)
208(1)	676(23)	3011(59)	167(1)	842(2)	2808(6)
212(22)	830(7)	3022(50)	203(2)	864(1)	3057(5)
229(1)	848(1)	3033(20)	213(1)	930(2)	3059(5)
251(10)	928(0)	3036(74)	258(1)	938(1)	3069(8)
259(6)	929(1)	3103(20)	303(1)	954(2)	3074(3)
273(4)	934(0)	3108(19)	352(2)	974(3)	3126(2)
306(1)	966(1)	3118(13)	365(2)	998(2)	3127(5)
356(16)	1057(35)	3124(16)	371(1)	1065(11)	3148(3)
377(1)	1062(14)	3165(12)	376(0)	1089(0)	3163(0)
391(1)	1081(0)	3168(19)	383(1)	1093(4)	3180(9)
396(5)	1088(27)	3169(14)	407(2)	1112(5)	3193(2)
416(9)	1203(6)	3170(19)	416(0)	1280(4)	3200(1)

Table S133. Harmonic vibrational frequencies (in cm^{-1}) and infrared intensities (in parentheses, in km/mol) for the $(Me_2C{=}N)_2Cr_2(CO)_9$ structure **2-9S-1**.

M06-L			B3PW91-D3		
19(1)	390(5)	1123(7)	17(1)	417(3)	1142(8)
36(1)	403(2)	1295(2)	33(0)	421(2)	1295(2)
43(1)	416(0)	1307(2)	44(1)	428(0)	1305(1)
54(0)	420(3)	1386(7)	56(0)	432(1)	1379(6)
59(0)	427(32)	1387(2)	63(0)	446(0)	1396(18)
63(1)	431(35)	1392(12)	64(1)	460(0)	1399(14)
69(1)	434(45)	1398(8)	71(1)	470(27)	1404(16)
75(0)	437(10)	1418(27)	73(0)	471(14)	1428(27)
78(0)	448(1)	1448(35)	80(0)	476(28)	1458(25)
81(0)	468(3)	1456(16)	83(0)	482(26)	1462(29)
84(1)	472(2)	1459(7)	87(1)	491(6)	1471(8)
89(1)	473(1)	1464(18)	90(0)	493(7)	1473(9)
92(1)	488(4)	1465(18)	93(1)	503(6)	1474(23)
92(0)	528(5)	1470(21)	95(1)	539(4)	1487(26)
97(2)	530(0)	1506(13)	98(1)	544(0)	1529(10)
103(1)	531(6)	1661(16)	106(0)	550(6)	1687(21)
108(0)	549(2)	1693(12)	111(1)	564(4)	1723(10)
109(2)	557(3)	1976(1025)	112(0)	571(3)	2000(1301)
113(1)	568(13)	1982(689)	116(1)	581(11)	2004(533)
117(0)	580(1)	1997(2024)	118(0)	593(1)	2018(1735)
122(0)	585(1)	2010(1418)	120(1)	597(0)	2032(1335)
132(1)	633(68)	2014(1213)	128(0)	652(72)	2036(1651)
139(1)	644(44)	2021(440)	129(0)	666(61)	2041(537)
158(0)	661(92)	2050(381)	147(0)	692(104)	2075(326)
163(0)	662(80)	2088(474)	163(0)	693(110)	2111(446)
179(1)	692(123)	2125(294)	171(1)	719(150)	2148(277)
191(1)	702(40)	2941(13)	180(2)	725(51)	2892(11)
206(0)	742(5)	3040(17)	186(1)	754(9)	3062(5)
213(0)	846(2)	3045(10)	209(0)	848(1)	3065(2)
215(0)	860(4)	3051(12)	214(1)	859(3)	3076(4)
260(1)	930(1)	3087(22)	273(1)	936(1)	3099(7)
355(0)	942(2)	3122(11)	366(2)	939(1)	3129(3)
360(2)	956(3)	3125(13)	367(0)	969(3)	3131(4)
372(1)	971(4)	3136(3)	383(0)	976(6)	3151(2)
376(3)	994(1)	3143(6)	393(1)	1000(1)	3160(3)
382(1)	1071(3)	3167(22)	398(2)	1076(3)	3175(11)
385(0)	1074(1)	3174(9)	404(2)	1083(1)	3201(3)
386(1)	1082(3)	3194(10)	414(1)	1090(4)	3218(5)

Table S134. Harmonic vibrational frequencies (in cm^{-1}) and infrared intensities (in parentheses, in km/mol) for the $(Me_2C=N)_2Cr_2(CO)_9$ structure **2-9S-2**.

M06-L			B3PW91-D3		
30(0)	406(2)	1107(15)	33(0)	432(1)	1118(21)
36(1)	423(0)	1237(2)	38(1)	442(2)	1238(1)
46(1)	424(3)	1285(5)	48(1)	450(2)	1282(6)
51(0)	445(19)	1376(5)	57(0)	462(1)	1383(9)
56(0)	446(0)	1378(7)	64(1)	464(4)	1388(11)
68(0)	450(6)	1383(9)	71(0)	467(6)	1393(17)
75(1)	451(10)	1392(36)	77(1)	476(1)	1398(37)
77(0)	455(1)	1446(17)	84(0)	479(25)	1452(34)
84(0)	456(3)	1448(14)	91(0)	481(5)	1458(19)
87(0)	470(11)	1454(15)	95(0)	490(5)	1463(8)
90(1)	474(11)	1459(17)	96(1)	498(14)	1475(8)
91(0)	478(12)	1459(2)	98(0)	509(3)	1476(12)
95(0)	491(6)	1466(13)	102(0)	516(8)	1480(15)
101(1)	504(15)	1468(17)	107(1)	526(11)	1481(8)
105(2)	510(13)	1475(10)	109(0)	531(12)	1486(15)
108(0)	529(5)	1662(133)	112(1)	548(1)	1687(145)
110(0)	540(14)	1701(43)	121(1)	561(29)	1724(43)
116(0)	556(37)	1766(318)	127(0)	589(20)	1757(330)
123(0)	590(4)	1977(401)	131(1)	607(5)	1977(495)
125(1)	604(49)	2004(204)	136(0)	614(44)	2026(242)
136(1)	612(10)	2009(1268)	149(1)	637(3)	2031(1229)
145(0)	622(84)	2023(391)	153(0)	647(65)	2043(525)
155(2)	630(88)	2031(992)	168(2)	656(93)	2056(1101)
164(2)	633(54)	2064(1367)	175(1)	659(135)	2086(1450)
170(1)	650(50)	2072(1391)	184(2)	672(32)	2102(1113)
179(2)	672(124)	2119(626)	192(1)	695(133)	2144(655)
189(0)	677(34)	3032(35)	194(2)	706(64)	3052(11)
199(1)	761(113)	3042(22)	206(1)	782(101)	3062(2)
258(2)	833(9)	3045(9)	274(0)	837(4)	3065(11)
264(0)	849(9)	3047(30)	280(1)	847(9)	3067(4)
272(1)	919(1)	3110(11)	288(0)	927(0)	3120(4)
287(3)	923(2)	3123(6)	303(0)	929(3)	3132(4)
294(1)	943(41)	3134(22)	311(3)	961(4)	3149(5)
316(2)	960(6)	3137(7)	334(3)	964(38)	3150(9)
366(20)	990(29)	3152(26)	393(4)	998(50)	3163(17)
374(5)	1076(1)	3169(19)	397(18)	1083(3)	3182(8)
386(3)	1079(7)	3176(12)	407(2)	1086(7)	3188(8)
401(0)	1080(2)	3197(5)	420(1)	1089(4)	3207(2)

Table S135. Harmonic vibrational frequencies (in cm^{-1}) and infrared intensities (in parentheses, in km/mol) for the $(Me_2C{=}N)_2Cr_2(CO)_9$ structure **2-9S-3**.

M06-L			B3PW91-D3		
24(1)	388(2)	1095(49)	20(0)	411(5)	1104(51)
35(1)	396(2)	1213(0)	28(0)	420(11)	1211(0)
38(1)	399(17)	1221(4)	42(0)	423(6)	1224(5)
51(0)	406(6)	1369(12)	48(0)	425(2)	1378(16)
56(0)	415(1)	1376(6)	52(1)	431(0)	1383(18)
58(0)	423(22)	1376(9)	56(1)	441(5)	1386(3)
63(1)	428(6)	1379(7)	64(0)	449(15)	1388(11)
74(1)	434(2)	1449(1)	70(0)	463(18)	1456(13)
75(0)	438(48)	1450(8)	73(0)	472(4)	1459(2)
80(0)	457(7)	1452(9)	78(1)	480(7)	1464(23)
81(0)	458(2)	1458(32)	83(0)	484(25)	1464(25)
86(1)	473(4)	1460(8)	86(0)	501(1)	1469(7)
88(0)	481(2)	1465(2)	91(0)	502(2)	1478(1)
93(0)	500(1)	1469(12)	94(0)	510(6)	1480(15)
98(1)	513(2)	1483(5)	99(1)	527(2)	1490(6)
99(1)	518(0)	1656(56)	102(1)	534(0)	1670(65)
104(0)	532(5)	1764(54)	105(1)	545(2)	1802(48)
107(0)	534(2)	1985(1188)	109(1)	546(5)	2003(1357)
110(2)	541(1)	2008(727)	109(1)	555(0)	2022(771)
113(0)	548(3)	2010(806)	115(2)	558(1)	2034(868)
114(0)	563(13)	2029(432)	123(0)	576(18)	2049(431)
122(1)	582(16)	2045(516)	126(0)	605(20)	2074(609)
131(1)	605(21)	2053(1823)	140(1)	619(31)	2086(1600)
139(0)	613(27)	2061(651)	145(0)	633(38)	2093(785)
163(0)	629(77)	2099(864)	165(1)	650(61)	2124(497)
188(0)	647(97)	2127(203)	167(0)	668(93)	2153(212)
192(2)	660(70)	3035(33)	194(2)	686(101)	3055(8)
206(0)	661(78)	3038(67)	205(0)	695(104)	3057(6)
231(2)	685(102)	3040(20)	233(0)	709(120)	3059(34)
256(1)	832(10)	3050(40)	235(3)	824(10)	3070(14)
272(2)	860(5)	3116(8)	276(2)	866(3)	3126(4)
279(3)	913(2)	3123(13)	287(2)	918(2)	3132(3)
288(1)	930(1)	3128(15)	295(2)	935(0)	3139(10)
306(3)	941(0)	3131(19)	311(5)	944(0)	3143(13)
348(2)	946(2)	3162(16)	367(0)	947(2)	3178(8)
363(1)	1067(8)	3165(31)	373(0)	1070(8)	3183(7)
375(3)	1075(0)	3191(17)	398(14)	1084(0)	3193(14)
377(22)	1079(0)	3196(11)	407(1)	1085(1)	3196(8)

Table S136. Harmonic vibrational frequencies (in cm^{-1}) and infrared intensities (in parentheses, in km/mol) for the $(Me_2C{=}N)_2Cr_2(CO)_9$ structure **2-9S-4**.

M06-L			B3PW91-D3		
26(0)	392(1)	1100(15)	22(0)	404(1)	1109(16)
38(1)	393(1)	1275(1)	36(1)	404(16)	1265(2)
48(1)	399(16)	1287(8)	46(1)	413(11)	1286(7)
57(3)	409(1)	1386(22)	60(2)	420(1)	1397(30)
62(0)	414(7)	1390(12)	64(0)	430(7)	1402(1)
67(0)	418(19)	1397(14)	71(0)	457(28)	1405(59)
75(0)	433(39)	1402(41)	78(1)	469(23)	1411(21)
77(2)	436(27)	1430(25)	80(1)	471(28)	1434(21)
83(1)	457(10)	1444(6)	81(1)	478(9)	1457(9)
84(2)	460(0)	1448(4)	85(1)	481(16)	1460(4)
89(1)	467(1)	1450(4)	92(1)	487(6)	1463(5)
93(0)	482(11)	1452(4)	98(0)	499(9)	1468(17)
96(0)	494(17)	1456(31)	100(0)	508(26)	1474(10)
102(1)	494(13)	1466(2)	105(1)	516(1)	1483(10)
106(0)	505(12)	1475(13)	109(2)	525(4)	1491(11)
110(1)	507(7)	1521(6)	111(0)	530(20)	1519(7)
116(0)	545(3)	1693(65)	121(1)	560(4)	1720(69)
118(1)	556(6)	1969(327)	131(1)	562(16)	1982(417)
124(5)	559(5)	1994(587)	135(2)	569(1)	2019(909)
129(2)	565(14)	2000(321)	138(1)	579(13)	2027(21)
137(8)	574(3)	2027(790)	148(2)	589(2)	2052(557)
156(1)	632(65)	2030(1666)	153(6)	654(54)	2056(1714)
168(2)	637(57)	2034(953)	173(0)	662(89)	2061(1294)
171(9)	643(76)	2048(1328)	183(8)	667(99)	2072(1227)
187(12)	655(44)	2087(1151)	197(12)	681(75)	2111(1165)
197(0)	665(210)	2126(359)	214(2)	693(274)	2149(345)
204(3)	681(35)	3018(36)	232(3)	702(11)	3039(20)
221(1)	730(7)	3029(50)	237(1)	739(19)	3054(10)
229(2)	811(3)	3035(27)	247(2)	820(6)	3061(21)
247(0)	846(8)	3041(17)	273(1)	845(10)	3065(7)
283(0)	916(6)	3115(8)	310(0)	930(3)	3124(5)
343(66)	939(1)	3116(12)	357(7)	946(1)	3136(3)
352(6)	946(1)	3123(6)	366(20)	954(1)	3137(4)
357(9)	954(3)	3124(11)	381(3)	963(4)	3144(5)
369(33)	983(1)	3168(12)	386(6)	996(0)	3182(4)
370(2)	1056(42)	3169(13)	393(23)	1065(31)	3188(7)
373(11)	1068(17)	3172(8)	395(32)	1079(36)	3189(4)
379(13)	1088(1)	3218(2)	402(2)	1098(3)	3232(1)

Table S137. Harmonic vibrational frequencies (in cm^{-1}) and infrared intensities (in parentheses, in km/mol) for the $(Me_2C{=}N)_2Cr_2(CO)_9$ structure **2-9S-5**.

M06-L			B3PW91-D3		
25(0)	387(2)	1120(13)	15(0)	405(3)	1132(19)
31(1)	395(4)	1289(5)	23(1)	413(0)	1288(7)
34(1)	399(9)	1294(1)	34(1)	418(2)	1293(1)
46(0)	403(6)	1388(23)	42(1)	424(7)	1395(26)
49(0)	416(23)	1391(16)	49(0)	435(1)	1397(25)
51(0)	418(69)	1401(20)	55(0)	453(39)	1405(5)
60(1)	422(4)	1406(36)	60(1)	461(6)	1405(47)
61(0)	444(92)	1448(21)	62(0)	468(90)	1454(27)
70(0)	458(3)	1452(3)	70(0)	470(57)	1458(10)
74(0)	465(3)	1453(6)	75(0)	479(36)	1460(9)
77(0)	471(28)	1458(8)	77(0)	487(0)	1467(7)
80(0)	482(12)	1459(12)	80(1)	489(9)	1470(15)
82(0)	483(5)	1464(20)	86(1)	501(10)	1478(3)
85(0)	497(9)	1473(8)	91(1)	509(0)	1481(7)
89(1)	516(1)	1480(2)	91(0)	523(1)	1485(18)
92(0)	526(0)	1671(12)	95(1)	548(3)	1690(17)
99(3)	548(9)	1701(60)	98(1)	563(9)	1732(43)
101(1)	557(27)	1975(968)	102(2)	565(8)	1989(1244)
102(1)	560(5)	1982(1049)	108(0)	576(3)	2002(1247)
115(1)	573(0)	1989(513)	117(1)	583(0)	2013(821)
126(1)	583(1)	1998(1204)	122(1)	599(0)	2033(886)
133(1)	636(51)	2012(710)	130(0)	660(68)	2044(1558)
138(1)	643(68)	2038(2042)	135(2)	673(15)	2061(2179)
155(1)	645(13)	2056(1561)	145(6)	673(111)	2082(515)
163(2)	646(71)	2068(1151)	156(0)	675(121)	2095(680)
172(1)	666(100)	2128(307)	171(1)	690(92)	2155(146)
180(8)	696(55)	3036(17)	178(1)	723(73)	3055(7)
200(2)	724(8)	3037(32)	186(1)	727(13)	3057(3)
208(7)	828(3)	3039(26)	198(0)	831(4)	3060(14)
210(1)	849(2)	3040(26)	216(2)	845(2)	3060(14)
236(1)	927(9)	3115(13)	219(0)	937(9)	3129(6)
350(1)	950(3)	3121(11)	359(0)	950(2)	3129(9)
351(3)	960(2)	3122(16)	360(2)	956(2)	3134(7)
352(0)	966(2)	3126(10)	362(3)	972(2)	3137(5)
363(20)	989(4)	3161(19)	367(0)	1000(7)	3173(4)
375(1)	1073(5)	3162(3)	375(4)	1078(7)	3182(4)
376(7)	1086(0)	3176(11)	388(3)	1089(0)	3186(8)
379(3)	1091(1)	3184(5)	402(4)	1096(2)	3200(3)

Table S138. Harmonic vibrational frequencies (in cm^{-1}) and infrared intensities (in parentheses, in km/mol) for the $(Me_2C{=}N)_2Cr_2(CO)_9$ structure **2-9T-6**.

M06-L			B3PW91-D3		
25(0)	375(2)	1096(75)	21(1)	399(21)	1085(6)
26(0)	375(1)	1200(5)	31(1)	410(25)	1203(7)
36(1)	390(1)	1221(19)	46(0)	414(3)	1205(1)
38(1)	399(4)	1366(18)	47(2)	415(1)	1373(23)
40(1)	400(3)	1367(4)	51(1)	425(20)	1381(4)
42(0)	404(19)	1375(5)	55(2)	427(15)	1384(23)
45(0)	410(14)	1376(14)	69(0)	435(4)	1389(16)
58(0)	416(26)	1449(6)	72(1)	445(41)	1451(7)
60(0)	417(31)	1450(6)	76(0)	453(11)	1459(2)
61(1)	429(17)	1451(1)	80(1)	462(16)	1462(29)
65(0)	432(24)	1456(10)	85(7)	467(2)	1466(13)
68(0)	446(7)	1459(1)	85(4)	478(33)	1467(6)
73(0)	461(2)	1459(4)	90(5)	484(27)	1482(13)
74(0)	485(4)	1475(12)	95(4)	492(3)	1483(2)
78(0)	488(8)	1484(8)	100(0)	496(1)	1487(7)
81(0)	513(1)	1749(9)	102(6)	516(4)	1688(49)
83(1)	516(1)	1765(14)	107(5)	536(3)	1772(14)
89(0)	521(3)	1996(976)	110(0)	543(2)	2016(520)
91(2)	523(1)	2019(456)	114(0)	549(4)	2026(993)
100(0)	526(2)	2029(1850)	116(1)	551(6)	2034(953)
106(1)	536(5)	2032(863)	121(0)	559(14)	2056(1300)
112(1)	540(4)	2041(1983)	130(0)	583(29)	2061(2138)
118(1)	559(33)	2044(871)	144(0)	590(27)	2068(863)
122(0)	595(40)	2060(13)	149(0)	597(16)	2071(1111)
134(0)	628(35)	2103(740)	160(0)	620(39)	2082(1350)
154(0)	640(91)	2128(439)	172(3)	629(170)	2139(583)
161(0)	643(78)	3026(42)	185(0)	682(151)	3057(11)
168(5)	650(81)	3031(25)	208(0)	684(96)	3058(4)
209(3)	664(142)	3032(68)	231(1)	700(177)	3062(18)
230(1)	828(18)	3042(38)	244(4)	823(18)	3064(22)
247(1)	862(16)	3114(11)	252(2)	831(15)	3126(6)
264(2)	916(0)	3117(19)	262(4)	920(1)	3131(8)
270(0)	930(1)	3120(16)	285(2)	922(1)	3133(7)
289(1)	934(0)	3133(10)	294(13)	939(1)	3146(3)
347(0)	943(0)	3156(18)	363(14)	943(0)	3183(9)
366(3)	1073(3)	3160(14)	373(1)	1067(17)	3192(4)
367(1)	1077(6)	3165(19)	386(30)	1082(12)	3199(7)
370(8)	1078(54)	3171(12)	393(6)	1084(53)	3202(7)

Table S139. Harmonic vibrational frequencies (in cm^{-1}) and infrared intensities (in parentheses, in km/mol) for the $(Me_2C{=}N)_2Cr_2(CO)_9$ structure **2-9T-7**.

M06-L			B3PW91-D3		
7(0)	382(3)	1082(91)	15(0)	397(8)	1087(69)
20(0)	383(2)	1198(5)	28(1)	404(3)	1196(9)
22(0)	383(5)	1199(12)	32(1)	410(4)	1200(10)
32(0)	387(3)	1365(4)	35(0)	414(2)	1371(14)
34(0)	395(4)	1366(28)	36(0)	421(7)	1373(25)
41(0)	401(20)	1375(2)	46(0)	422(1)	1381(2)
47(0)	409(18)	1377(0)	52(0)	439(12)	1383(7)
50(0)	415(26)	1446(3)	56(0)	442(37)	1457(8)
55(0)	421(42)	1451(8)	59(0)	462(30)	1459(1)
56(0)	422(38)	1453(3)	64(0)	463(35)	1459(10)
60(1)	452(2)	1456(4)	66(0)	464(12)	1463(2)
67(0)	476(4)	1460(3)	71(0)	482(21)	1467(7)
71(1)	487(5)	1462(3)	74(0)	495(1)	1470(3)
76(0)	494(0)	1468(21)	77(1)	500(5)	1479(22)
78(0)	496(1)	1477(14)	81(0)	507(1)	1484(24)
80(0)	518(1)	1766(5)	82(1)	526(2)	1783(5)
82(0)	519(3)	1777(3)	87(1)	529(8)	1793(0)
85(0)	525(16)	1896(1666)	93(0)	536(7)	1937(1618)
93(0)	529(1)	2004(1397)	99(1)	545(6)	2027(1450)
98(0)	534(10)	2009(697)	106(1)	547(1)	2037(501)
101(1)	542(5)	2021(1183)	109(1)	550(4)	2042(1163)
113(2)	556(7)	2040(1261)	113(1)	568(5)	2051(1689)
121(0)	557(4)	2053(2176)	125(0)	572(6)	2069(1747)
129(0)	612(44)	2055(250)	128(0)	637(128)	2078(431)
133(0)	619(149)	2102(573)	136(2)	650(80)	2120(793)
156(1)	657(76)	2134(163)	145(1)	679(86)	2157(53)
168(1)	658(50)	3034(18)	152(1)	686(105)	3056(12)
177(0)	665(137)	3036(26)	177(0)	688(149)	3056(13)
202(2)	685(72)	3036(43)	178(0)	709(83)	3060(40)
212(0)	827(20)	3038(91)	193(15)	827(24)	3061(15)
218(15)	831(22)	3120(21)	239(3)	831(17)	3131(9)
236(3)	914(0)	3124(6)	246(1)	919(0)	3135(4)
253(9)	919(0)	3126(26)	254(3)	924(0)	3139(10)
307(1)	932(0)	3127(16)	281(2)	936(0)	3141(12)
354(0)	937(1)	3164(17)	366(0)	938(0)	3179(8)
358(9)	1075(54)	3168(17)	373(26)	1080(100)	3183(11)
375(2)	1077(2)	3169(29)	391(2)	1083(2)	3188(6)
378(0)	1079(23)	3180(11)	393(2)	1085(9)	3192(6)

Table S140. Harmonic vibrational frequencies (in cm^{-1}) and infrared intensities (in parentheses, in km/mol) for the $(Me_2C{=}N)_2Cr_2(CO)_9$ structure **2-9T-8**.

M06-L			B3PW91-D3		
18(1)	381(0)	1111(14)	11(0)	396(1)	1120(13)
33(0)	384(4)	1280(3)	28(1)	399(3)	1264(1)
44(2)	389(3)	1288(12)	48(0)	400(3)	1281(10)
53(0)	397(6)	1384(24)	53(1)	407(11)	1392(31)
60(0)	399(2)	1396(7)	61(0)	411(1)	1400(24)
64(1)	402(2)	1399(19)	64(0)	415(1)	1401(18)
69(1)	416(4)	1404(29)	71(1)	424(1)	1408(64)
71(1)	420(17)	1419(49)	73(1)	435(3)	1413(21)
73(1)	422(16)	1443(4)	78(1)	438(30)	1454(3)
78(1)	425(7)	1452(6)	82(2)	463(29)	1461(10)
81(0)	430(49)	1455(16)	83(0)	464(0)	1462(3)
84(2)	457(15)	1459(15)	89(3)	470(32)	1466(29)
88(0)	465(2)	1465(1)	92(0)	491(6)	1471(2)
92(1)	467(5)	1470(4)	94(1)	495(17)	1481(8)
95(1)	483(11)	1477(16)	101(1)	502(4)	1489(22)
99(2)	519(6)	1510(26)	103(1)	531(1)	1507(23)
104(1)	520(0)	1688(66)	105(1)	537(2)	1712(78)
105(1)	525(8)	1983(945)	112(1)	545(4)	2007(751)
111(1)	531(5)	2001(546)	112(1)	548(4)	2022(552)
115(0)	546(29)	2010(389)	114(1)	555(23)	2031(891)
121(3)	548(5)	2020(670)	119(2)	566(3)	2034(1066)
131(1)	565(8)	2023(1663)	127(1)	580(9)	2050(615)
153(8)	573(22)	2039(891)	138(0)	591(19)	2051(1579)
161(0)	583(47)	2048(1039)	157(0)	595(57)	2072(702)
167(4)	651(71)	2086(1066)	174(1)	682(89)	2104(1138)
184(4)	655(77)	2123(334)	187(10)	685(91)	2143(305)
195(0)	679(139)	3014(28)	193(6)	710(127)	3047(4)
213(0)	721(2)	3026(55)	205(0)	715(27)	3051(35)
220(0)	797(4)	3038(15)	223(1)	794(11)	3059(7)
290(2)	841(11)	3040(27)	234(0)	830(12)	3068(10)
321(19)	926(6)	3109(13)	267(0)	929(2)	3129(3)
338(10)	945(1)	3119(12)	334(33)	940(1)	3133(1)
351(5)	951(2)	3121(8)	350(6)	952(2)	3136(14)
356(2)	959(1)	3128(9)	353(5)	956(2)	3137(2)
360(2)	998(1)	3158(12)	367(0)	998(2)	3186(5)
364(7)	1053(76)	3168(8)	375(1)	1054(47)	3191(4)
373(1)	1070(8)	3169(13)	379(5)	1072(52)	3198(6)
375(4)	1089(3)	3170(8)	388(2)	1095(3)	3205(4)

Table S141. Harmonic vibrational frequencies (in cm^{-1}) and infrared intensities (in parentheses, in km/mol) for the $(Me_2C{=}N)_2Cr_2(CO)_9$ structure **2-9T-9**.

M06-L			B3PW91-D3		
22(1)	375(0)	1118(15)	3(0)	378(5)	1132(27)
28(3)	386(5)	1288(1)	13(1)	396(5)	1285(5)
34(1)	387(1)	1293(2)	15(0)	400(2)	1291(2)
40(2)	391(12)	1385(28)	28(0)	406(2)	1393(15)
45(0)	393(45)	1386(13)	34(0)	408(0)	1394(37)
48(1)	399(11)	1399(29)	41(2)	411(0)	1404(5)
60(1)	409(42)	1408(34)	47(0)	422(6)	1405(61)
65(0)	416(26)	1445(29)	54(0)	425(9)	1456(23)
66(3)	420(1)	1450(5)	60(4)	452(12)	1458(10)
68(1)	440(76)	1451(14)	66(1)	460(14)	1462(5)
74(0)	444(6)	1456(7)	69(1)	462(42)	1466(8)
76(0)	451(19)	1458(13)	73(1)	474(53)	1468(18)
77(1)	464(4)	1462(22)	75(0)	475(0)	1471(15)
81(1)	471(9)	1473(4)	79(1)	488(26)	1480(3)
87(1)	501(15)	1477(12)	86(2)	494(16)	1483(23)
90(1)	513(1)	1668(16)	87(0)	517(1)	1686(21)
92(1)	516(1)	1698(47)	93(1)	531(0)	1726(49)
96(0)	526(1)	1859(1774)	97(1)	535(1)	1945(1071)
102(2)	544(45)	1991(987)	102(1)	567(58)	1997(1336)
108(0)	550(7)	1992(764)	108(1)	571(3)	2008(990)
118(1)	563(0)	2011(1399)	110(0)	576(0)	2020(787)
129(0)	575(0)	2018(2480)	120(0)	592(0)	2047(2535)
133(0)	608(59)	2025(1040)	128(0)	624(74)	2065(441)
147(1)	619(127)	2041(732)	145(5)	626(194)	2073(1091)
155(2)	633(47)	2059(3182)	151(9)	656(60)	2085(2101)
170(0)	635(64)	2112(153)	165(1)	668(57)	2136(393)
175(10)	671(92)	3039(11)	172(2)	712(113)	3056(5)
199(1)	722(13)	3040(17)	178(0)	720(14)	3058(3)
206(7)	829(4)	3040(27)	189(3)	824(5)	3061(17)
217(8)	850(1)	3042(40)	194(1)	842(1)	3062(14)
257(1)	927(12)	3121(10)	210(5)	933(6)	3124(8)
324(17)	944(3)	3121(17)	327(1)	946(3)	3126(6)
351(5)	960(0)	3124(14)	351(5)	956(2)	3134(9)
353(54)	965(2)	3126(10)	356(0)	965(1)	3135(4)
354(8)	989(7)	3164(18)	361(0)	992(8)	3171(7)
356(96)	1072(6)	3164(4)	365(3)	1073(10)	3181(7)
362(9)	1082(0)	3171(11)	367(31)	1088(0)	3184(4)
372(21)	1093(2)	3184(7)	373(33)	1095(2)	3206(3)

Table S142. Harmonic vibrational frequencies (in cm^{-1}) and infrared intensities (in parentheses, in km/mol) for the $(Me_2C{=}N)_2Cr_2(CO)_9$ structure **2-9S-10**.

M06-L			B3PW91-D3		
13(0)	382(1)	1097(30)	21(0)	406(12)	1179(24)
21(0)	385(3)	1214(15)	24(0)	422(4)	1269(37)
30(0)	389(4)	1218(18)	34(1)	425(2)	1284(8)
40(0)	397(3)	1368(10)	43(1)	442(9)	1322(33)
44(1)	411(7)	1372(34)	49(1)	448(28)	1396(14)
49(1)	419(12)	1376(0)	57(1)	450(27)	1401(45)
54(0)	420(25)	1379(3)	58(1)	455(29)	1408(59)
59(0)	422(38)	1449(2)	62(0)	463(19)	1412(15)
64(0)	431(1)	1451(2)	65(0)	475(5)	1454(10)
69(0)	453(1)	1455(7)	67(0)	481(24)	1457(7)
73(0)	465(2)	1459(4)	72(1)	489(15)	1461(3)
76(0)	471(1)	1463(9)	74(0)	503(10)	1463(60)
77(1)	476(3)	1469(18)	78(0)	513(206)	1466(28)
77(0)	489(4)	1474(9)	83(0)	513(5)	1469(104)
86(1)	503(26)	1483(15)	88(1)	531(18)	1478(11)
89(1)	512(17)	1736(115)	89(1)	544(13)	1485(16)
93(0)	518(0)	1772(34)	93(1)	566(24)	1514(201)
96(1)	529(1)	1972(1317)	97(1)	576(5)	1577(384)
103(0)	549(11)	2012(1805)	101(1)	592(48)	1715(175)
107(3)	555(5)	2021(664)	105(0)	605(54)	2019(783)
121(0)	556(5)	2028(1502)	119(0)	621(54)	2033(765)
127(1)	564(25)	2040(908)	126(1)	646(87)	2047(940)
130(1)	575(30)	2057(146)	149(4)	657(146)	2058(1078)
138(0)	599(54)	2065(1068)	157(1)	669(56)	2083(805)
147(1)	609(11)	2113(733)	168(2)	688(32)	2101(668)
151(0)	639(30)	2136(163)	183(0)	691(76)	2139(1124)
174(0)	658(94)	3022(22)	223(2)	802(83)	3052(1)
187(1)	659(88)	3029(82)	240(4)	813(51)	3058(28)
193(1)	685(105)	3030(21)	252(0)	830(38)	3072(3)
220(2)	840(2)	3032(68)	275(1)	874(9)	3077(17)
249(4)	857(2)	3109(6)	281(7)	933(1)	3118(2)
262(2)	922(0)	3111(32)	351(2)	939(1)	3122(9)
292(0)	926(1)	3114(21)	366(6)	946(7)	3154(0)
325(4)	934(0)	3120(20)	379(34)	986(1)	3159(15)
353(0)	934(1)	3140(34)	387(5)	1025(131)	3185(5)
356(3)	1078(2)	3159(21)	394(7)	1042(26)	3187(9)
374(1)	1079(2)	3170(21)	399(2)	1089(2)	3188(4)
382(5)	1085(25)	3173(18)	405(5)	1145(48)	3195(1)

Table S143. Harmonic vibrational frequencies (in cm^{-1}) and infrared intensities (in parentheses, in km/mol) for the $(Me_2C{=}N)_2Cr_2(CO)_9$ structure **2-9T-11**.

M06-L			B3PW91-D3		
17(0)	374(4)	1127(8)	6(0)	382(1)	1136(10)
26(1)	376(6)	1302(1)	21(0)	386(10)	1299(1)
38(0)	379(3)	1306(2)	25(1)	388(2)	1302(4)
40(1)	385(2)	1383(2)	28(1)	392(6)	1382(2)
44(1)	385(2)	1386(3)	34(1)	404(0)	1397(7)
50(0)	397(1)	1396(7)	39(1)	411(2)	1402(12)
56(1)	411(4)	1404(25)	48(0)	420(9)	1405(27)
62(0)	417(19)	1423(26)	49(1)	424(0)	1422(32)
63(1)	418(25)	1437(12)	56(0)	432(0)	1454(12)
66(0)	432(41)	1449(16)	62(2)	443(18)	1456(4)
72(0)	434(2)	1451(10)	64(0)	451(0)	1459(19)
73(0)	443(4)	1453(10)	67(0)	457(6)	1462(11)
76(1)	446(3)	1455(20)	72(1)	467(10)	1470(21)
83(1)	465(2)	1473(17)	75(1)	478(41)	1480(20)
85(1)	477(3)	1516(11)	79(1)	493(3)	1528(12)
88(2)	489(5)	1667(29)	80(0)	501(5)	1689(28)
95(0)	520(22)	1679(5)	82(0)	533(6)	1715(11)
95(2)	524(8)	1964(1553)	88(1)	537(3)	1985(1320)
100(1)	529(2)	1980(666)	90(2)	554(45)	1998(911)
103(1)	559(4)	1987(1584)	92(0)	570(3)	2005(1591)
106(0)	575(0)	2006(809)	97(1)	582(2)	2021(1369)
114(0)	583(4)	2008(832)	100(2)	594(3)	2029(339)
119(0)	609(41)	2021(1008)	103(1)	616(39)	2036(1414)
125(0)	617(80)	2081(402)	107(0)	632(69)	2104(260)
130(1)	633(39)	2094(405)	115(0)	649(47)	2115(437)
137(0)	644(42)	2187(138)	123(1)	665(51)	2212(96)
144(0)	701(42)	2919(10)	129(0)	724(51)	2905(9)
152(0)	743(3)	3035(18)	139(0)	740(4)	3050(6)
161(0)	846(3)	3040(18)	141(1)	845(1)	3065(3)
182(0)	859(5)	3042(15)	149(1)	853(3)	3071(3)
190(1)	930(2)	3081(14)	163(0)	938(2)	3097(7)
195(1)	943(4)	3120(13)	180(1)	939(1)	3130(2)
223(1)	963(2)	3124(7)	213(0)	965(2)	3132(5)
253(0)	972(3)	3130(17)	248(0)	968(1)	3143(0)
312(1)	990(1)	3140(9)	312(1)	998(1)	3147(7)
350(0)	1068(4)	3162(7)	348(3)	1075(1)	3154(5)
356(1)	1078(6)	3172(15)	355(1)	1080(8)	3191(7)
368(1)	1080(1)	3179(5)	365(2)	1090(6)	3199(3)

Table S144. Harmonic vibrational frequencies (in cm^{-1}) and infrared intensities (in parentheses, in km/mol) for the $(Me_2C{=}N)_2Cr_2(CO)_9$ structure **2-9S-12**.

M06-L			B3PW91-D3		
16(0)	380(5)	1202(141)	6(0)	382(1)	1136(10)
25(0)	389(1)	1263(6)	21(0)	386(10)	1299(1)
28(0)	398(8)	1309(2)	25(1)	388(2)	1302(4)
39(1)	408(2)	1354(64)	28(1)	392(6)	1382(2)
46(0)	417(2)	1399(11)	34(1)	404(0)	1397(7)
51(0)	422(50)	1401(22)	39(1)	411(2)	1402(12)
56(1)	426(56)	1405(7)	48(0)	420(9)	1405(27)
56(0)	433(96)	1410(10)	49(1)	424(0)	1422(32)
65(0)	463(4)	1446(3)	56(0)	432(0)	1454(12)
68(0)	469(6)	1455(7)	62(2)	443(18)	1456(4)
73(0)	477(2)	1462(8)	64(0)	451(0)	1459(19)
79(0)	485(27)	1463(2)	67(0)	457(6)	1462(11)
85(1)	489(6)	1467(4)	72(1)	467(10)	1470(21)
85(2)	498(2)	1476(19)	75(1)	478(41)	1480(20)
91(0)	527(4)	1476(19)	79(1)	493(3)	1528(12)
95(1)	547(9)	1495(6)	80(0)	501(5)	1689(28)
96(1)	553(16)	1781(76)	82(0)	533(6)	1715(11)
104(1)	555(1)	1974(1123)	88(1)	537(3)	1985(1320)
110(2)	557(9)	1982(131)	90(2)	554(45)	1998(911)
114(1)	570(12)	1984(1219)	92(0)	570(3)	2005(1591)
131(0)	577(0)	2000(638)	97(1)	582(2)	2021(1369)
140(1)	628(86)	2014(1439)	100(2)	594(3)	2029(339)
149(7)	641(60)	2027(1416)	103(1)	616(39)	2036(1414)
152(1)	644(40)	2054(2446)	107(0)	632(69)	2104(260)
169(0)	647(69)	2067(1172)	115(0)	649(47)	2115(437)
173(2)	667(96)	2124(438)	123(1)	665(51)	2212(96)
180(0)	675(5)	3027(45)	129(0)	724(51)	2905(9)
195(8)	700(79)	3032(27)	139(0)	740(4)	3050(6)
211(2)	777(2)	3040(15)	141(1)	845(1)	3065(3)
249(0)	921(2)	3042(47)	149(1)	853(3)	3071(3)
255(0)	944(0)	3103(15)	163(0)	938(2)	3097(7)
299(0)	963(3)	3114(5)	180(1)	939(1)	3130(2)
350(1)	975(0)	3130(7)	213(0)	965(2)	3132(5)
354(0)	990(1)	3133(33)	248(0)	968(1)	3143(0)
356(6)	1014(5)	3155(13)	312(1)	998(1)	3147(7)
360(1)	1069(2)	3156(20)	348(3)	1075(1)	3154(5)
369(0)	1071(2)	3162(8)	355(1)	1080(8)	3191(7)
378(9)	1137(5)	3164(32)	365(2)	1090(6)	3199(3)

Table S145. Harmonic vibrational frequencies (in cm^{-1}) and infrared intensities (in parentheses, in km/mol) for the $(Me_2C{=}N)_2Cr_2(CO)_9$ structure **2-9T-13**.

M06-L			B3PW91-D3		
27(2)	374(33)	1218(162)	6(1)	381(11)	1198(245)
28(1)	380(15)	1266(4)	7(2)	398(23)	1255(4)
36(2)	383(18)	1311(10)	14(2)	400(19)	1308(21)
41(0)	395(1)	1350(156)	17(2)	402(14)	1358(295)
45(1)	398(9)	1396(12)	28(30)	412(5)	1405(27)
52(1)	401(24)	1402(20)	34(7)	417(2)	1407(27)
56(3)	410(2)	1404(8)	42(0)	426(12)	1410(17)
60(1)	412(48)	1406(5)	49(1)	439(8)	1413(4)
60(1)	425(10)	1446(6)	57(11)	448(251)	1456(6)
70(0)	438(11)	1450(9)	60(6)	452(85)	1462(40)
74(0)	443(15)	1455(18)	67(0)	457(45)	1468(7)
75(0)	447(9)	1464(11)	72(1)	472(8)	1472(1)
78(0)	476(26)	1467(1)	76(1)	474(10)	1475(6)
82(0)	489(4)	1471(11)	78(1)	481(16)	1483(23)
85(2)	512(81)	1475(31)	84(0)	514(48)	1485(37)
98(1)	520(6)	1493(27)	85(3)	534(1)	1500(18)
100(3)	523(4)	1727(825)	88(1)	544(3)	1768(7290)
104(2)	528(2)	1766(63)	99(14)	550(9)	1787(1387)
109(0)	547(3)	1963(558)	103(1)	557(25)	2013(2069)
114(1)	548(2)	1995(2245)	110(5)	564(2)	2020(352)
128(2)	573(7)	2004(973)	123(9)	576(5)	2029(1867)
136(1)	606(62)	2012(418)	135(0)	590(65)	2038(3282)
144(1)	611(34)	2028(2018)	143(0)	631(68)	2045(974)
153(4)	623(34)	2029(1622)	147(3)	642(173)	2058(711)
164(0)	628(59)	2053(2610)	159(58)	648(10)	2070(3133)
169(4)	640(22)	2107(432)	163(2)	652(44)	2129(287)
172(1)	652(140)	3033(50)	172(0)	676(19)	3051(24)
206(1)	677(5)	3041(11)	173(1)	689(192)	3059(13)
220(7)	782(4)	3043(35)	180(12)	770(1)	3064(5)
242(1)	918(1)	3047(33)	212(63)	912(1)	3069(20)
253(22)	948(0)	3111(10)	228(13)	941(0)	3116(5)
299(1)	957(1)	3134(7)	293(6)	963(4)	3130(1)
346(6)	975(1)	3134(11)	338(19)	975(1)	3144(1)
353(13)	988(0)	3138(25)	349(421)	988(1)	3148(24)
357(192)	1016(7)	3155(12)	356(354)	1013(13)	3169(10)
362(15)	1063(24)	3159(18)	362(0)	1070(9)	3172(7)
367(4)	1078(4)	3167(14)	365(7)	1075(1)	3174(6)
369(1)	1136(6)	3170(11)	368(0)	1138(5)	3177(14)

Table S146. Harmonic vibrational frequencies (in cm^{-1}) and infrared intensities (in parentheses, in km/mol) for the $(Me_2C{=}N)_2Cr_2(CO)_9$ structure **2-9S-14**.

M06-L			B3PW91-D3		
19(1)	396(6)	1172(17)	8(0)	398(1)	1098(48)
30(1)	401(0)	1273(40)	12(0)	404(3)	1206(19)
36(0)	406(2)	1290(8)	18(0)	407(0)	1210(8)
43(1)	419(4)	1326(80)	22(1)	411(2)	1373(11)
48(0)	426(25)	1390(8)	29(1)	418(3)	1374(38)
49(0)	433(6)	1400(75)	38(1)	431(1)	1380(3)
61(1)	437(10)	1401(40)	50(0)	438(5)	1383(1)
64(0)	446(6)	1406(59)	54(1)	455(35)	1457(4)
66(1)	456(11)	1428(190)	57(0)	456(33)	1458(5)
70(1)	457(6)	1444(1)	59(0)	467(3)	1459(4)
76(1)	465(4)	1449(16)	66(1)	481(3)	1461(8)
78(0)	480(19)	1454(6)	73(0)	485(10)	1470(2)
82(0)	493(17)	1459(37)	74(0)	486(1)	1471(19)
84(0)	501(40)	1461(4)	77(0)	498(2)	1479(21)
84(0)	508(4)	1467(11)	79(0)	524(31)	1482(7)
91(0)	521(46)	1477(11)	87(0)	528(0)	1758(77)
98(0)	531(12)	1491(57)	89(0)	531(14)	1804(104)
107(1)	554(16)	1577(292)	94(0)	546(0)	2016(2011)
113(1)	560(10)	1689(153)	96(0)	554(16)	2037(625)
119(0)	581(95)	1996(674)	99(0)	559(3)	2047(973)
121(0)	603(43)	2011(784)	100(1)	566(5)	2050(1438)
126(0)	618(79)	2028(1250)	101(1)	568(4)	2069(1482)
141(19)	636(96)	2034(738)	104(1)	597(2)	2080(414)
171(6)	659(45)	2062(916)	128(0)	616(33)	2082(376)
179(4)	666(69)	2085(520)	132(3)	639(42)	2128(933)
182(6)	694(30)	2126(1011)	147(1)	647(68)	2157(123)
217(142)	793(70)	3035(13)	156(4)	688(108)	3050(15)
235(5)	804(16)	3039(64)	161(4)	688(108)	3051(8)
247(8)	839(52)	3052(10)	176(4)	708(141)	3055(49)
253(6)	868(10)	3055(41)	181(0)	833(1)	3058(20)
267(8)	931(1)	3113(1)	214(5)	852(2)	3123(5)
280(5)	939(1)	3115(21)	243(10)	919(1)	3123(1)
318(11)	946(7)	3148(4)	283(0)	923(0)	3127(19)
361(1)	983(0)	3152(26)	335(16)	925(0)	3130(15)
366(4)	1023(114)	3166(23)	364(0)	928(0)	3174(11)
380(5)	1039(9)	3168(13)	372(5)	1082(1)	3177(12)
382(11)	1081(0)	3178(18)	396(1)	1082(10)	3180(12)
390(3)	1136(52)	3183(5)	397(1)	1086(10)	3181(9)

Table S147. Harmonic vibrational frequencies (in cm^{-1}) and infrared intensities (in parentheses, in km/mol) for the $(Me_2C{=}N)_2Fe_2(CO)_6$ structure **2-6S-1**.

M06-L			B3PW91-D3		
40(0)	447(5)	1384(4)	-39(0)	458(8)	1390(0)
43(1)	454(0)	1384(0)	-34(0)	471(0)	1392(43)
53(0)	457(0)	1384(0)	40(1)	472(0)	1393(10)
61(1)	462(1)	1385(29)	49(0)	479(1)	1393(1)
62(0)	468(0)	1448(0)	50(0)	485(2)	1456(0)
64(0)	472(0)	1448(2)	61(1)	492(0)	1456(9)
65(0)	485(1)	1452(0)	65(0)	502(2)	1461(0)
75(0)	489(0)	1452(11)	67(0)	515(0)	1461(10)
77(0)	494(21)	1461(22)	71(0)	517(3)	1467(20)
77(0)	501(0)	1462(1)	78(0)	521(17)	1469(1)
81(0)	536(8)	1468(2)	83(0)	554(8)	1481(2)
86(0)	549(2)	1469(14)	90(0)	569(1)	1482(13)
90(0)	573(0)	1729(76)	92(0)	598(0)	1758(76)
91(0)	586(165)	1739(9)	94(0)	607(185)	1767(10)
92(0)	590(87)	2029(227)	95(0)	616(106)	2059(185)
107(0)	613(33)	2039(0)	112(0)	635(54)	2067(1148)
116(2)	627(40)	2039(1118)	125(3)	641(44)	2072(0)
133(0)	639(64)	2061(1364)	129(0)	659(55)	2090(1373)
142(1)	680(0)	2078(1622)	140(1)	698(0)	2100(1854)
161(2)	720(86)	2120(478)	165(3)	743(111)	2146(518)
166(0)	864(4)	3034(0)	179(0)	863(3)	3053(0)
230(0)	872(3)	3034(28)	240(0)	871(3)	3053(10)
252(0)	926(0)	3036(127)	259(0)	935(0)	3057(57)
277(0)	927(4)	3037(36)	289(0)	935(1)	3057(21)
287(0)	946(0)	3115(0)	299(0)	941(0)	3121(0)
294(0)	947(0)	3115(1)	307(0)	942(2)	3121(0)
326(2)	1081(2)	3116(3)	335(3)	1090(0)	3125(6)
353(3)	1081(1)	3116(60)	363(4)	1090(0)	3125(24)
410(3)	1089(35)	3171(0)	424(3)	1093(39)	3190(0)
422(0)	1096(15)	3171(22)	434(0)	1100(20)	3190(17)
430(3)	1244(0)	3172(28)	449(5)	1244(0)	3190(10)
439(2)	1246(0)	3172(10)	453(6)	1246(1)	3190(5)

Table S148. Harmonic vibrational frequencies (in cm^{-1}) and infrared intensities (in parentheses, in km/mol) for the $(Me_2C{=}N)_2Fe_2(CO)_6$ structure **2-6T-2**.

M06-L			B3PW91-D3		
-30(0)	398(4)	1379(13)	-39(0)	458(8)	1390(0)
30(0)	404(8)	1379(1)	-34(0)	471(0)	1392(43)
36(1)	431(7)	1381(0)	40(1)	472(0)	1393(10)
44(0)	444(7)	1382(23)	49(0)	479(1)	1393(1)
52(0)	447(0)	1444(0)	50(0)	485(2)	1456(0)
52(2)	454(1)	1444(9)	61(1)	492(0)	1456(9)
55(0)	467(5)	1450(0)	65(0)	502(2)	1461(0)
63(0)	472(0)	1450(0)	67(0)	515(0)	1461(10)
63(0)	475(7)	1454(40)	71(0)	517(3)	1467(20)
66(0)	475(1)	1455(3)	78(0)	521(17)	1469(1)
84(0)	509(3)	1472(0)	83(0)	554(8)	1481(2)
85(0)	524(0)	1472(7)	90(0)	569(1)	1482(13)
91(0)	531(0)	1690(217)	92(0)	598(0)	1758(76)
91(1)	556(40)	1699(35)	94(0)	607(185)	1767(10)
107(0)	562(56)	2029(71)	95(0)	616(106)	2059(185)
131(0)	579(18)	2035(1718)	112(0)	635(54)	2067(1148)
140(0)	608(34)	2035(0)	125(3)	641(44)	2072(0)
145(0)	620(32)	2042(1529)	129(0)	659(55)	2090(1373)
155(1)	667(0)	2090(1233)	140(1)	698(0)	2100(1854)
156(0)	695(9)	2109(210)	165(3)	743(111)	2146(518)
164(0)	848(1)	3035(0)	179(0)	863(3)	3053(0)
177(0)	857(0)	3035(23)	240(0)	871(3)	3053(10)
252(0)	913(0)	3039(123)	259(0)	935(0)	3057(57)
260(2)	913(0)	3039(20)	289(0)	935(1)	3057(21)
271(0)	935(2)	3115(0)	299(0)	941(0)	3121(0)
282(0)	935(0)	3115(0)	307(0)	942(2)	3121(0)
310(3)	1078(14)	3117(1)	335(3)	1090(0)	3125(6)
321(7)	1082(0)	3117(57)	363(4)	1090(0)	3125(24)
356(0)	1082(5)	3179(0)	424(3)	1093(39)	3190(0)
356(1)	1085(5)	3179(25)	434(0)	1100(20)	3190(17)
381(0)	1234(0)	3180(23)	449(5)	1244(0)	3190(10)
398(8)	1235(0)	3180(4)	453(6)	1246(1)	3190(5)

Table S149. Harmonic vibrational frequencies (in cm^{-1}) and infrared intensities (in parentheses, in km/mol) for the $(Me_2C{=}N)_2Fe_2(CO)_6$ structure **2-6T-3**.

M06-L			B3PW91-D3		
25(0)	416(2)	1368(12)	21(0)	425(3)	1372(21)
32(0)	417(8)	1376(11)	32(0)	428(5)	1383(3)
36(1)	430(3)	1378(0)	36(0)	448(2)	1384(17)
40(0)	440(8)	1380(7)	41(1)	457(2)	1387(7)
51(0)	445(2)	1450(1)	53(1)	464(3)	1459(3)
53(0)	451(7)	1451(14)	58(0)	466(19)	1459(13)
62(1)	462(1)	1455(4)	60(0)	479(2)	1460(9)
70(0)	466(9)	1459(0)	65(0)	486(7)	1461(5)
73(0)	480(7)	1460(7)	72(0)	495(5)	1468(2)
76(0)	487(27)	1465(9)	80(0)	510(16)	1472(2)
76(0)	502(13)	1475(12)	81(0)	513(26)	1480(16)
79(0)	517(3)	1476(4)	84(0)	533(1)	1483(6)
83(0)	532(19)	1721(51)	88(0)	545(27)	1743(63)
95(1)	549(8)	1767(41)	88(0)	560(8)	1789(40)
101(0)	563(14)	2018(480)	97(0)	582(20)	2044(185)
114(1)	574(84)	2025(545)	106(1)	593(103)	2055(477)
125(0)	576(87)	2039(630)	112(0)	597(91)	2060(839)
134(1)	600(11)	2055(1152)	121(0)	613(2)	2077(1294)
144(1)	617(77)	2074(1385)	141(2)	634(72)	2099(1492)
154(1)	682(61)	2113(837)	149(1)	695(85)	2132(870)
173(1)	840(11)	3030(35)	156(0)	834(12)	3053(11)
177(0)	859(6)	3032(33)	167(1)	857(3)	3054(6)
196(2)	921(0)	3036(72)	207(2)	922(0)	3058(47)
229(1)	926(1)	3041(28)	237(2)	925(0)	3061(19)
262(0)	929(1)	3113(14)	270(0)	929(1)	3122(7)
277(1)	964(6)	3119(2)	279(1)	950(3)	3127(0)
296(3)	1078(0)	3120(31)	305(3)	1082(68)	3130(17)
329(3)	1079(1)	3135(11)	333(4)	1084(13)	3135(11)
333(0)	1082(54)	3154(22)	345(1)	1088(2)	3179(11)
384(10)	1089(16)	3158(23)	391(21)	1091(24)	3185(8)
405(2)	1208(5)	3160(21)	414(2)	1200(6)	3185(10)
410(4)	1246(2)	3173(20)	418(5)	1241(1)	3187(10)

Table S150. Harmonic vibrational frequencies (in cm^{-1}) and infrared intensities (in parentheses, in km/mol) for the $(Me_2C=N)_2Fe_2(CO)_6$ structure **2-6S-4**.

M06-L			B3PW91-D3		
20(1)	433(2)	1367(29)	13(0)	448(6)	1375(28)
35(0)	438(4)	1372(1)	19(1)	455(2)	1377(10)
49(0)	450(0)	1377(4)	38(0)	461(4)	1385(1)
49(0)	453(1)	1381(1)	49(0)	467(6)	1386(1)
62(0)	469(18)	1447(4)	54(0)	488(15)	1455(2)
62(2)	474(0)	1449(3)	59(0)	493(0)	1460(4)
68(0)	489(0)	1454(1)	64(4)	512(0)	1460(13)
81(0)	494(41)	1456(3)	67(0)	515(61)	1465(0)
84(0)	510(14)	1458(2)	73(2)	533(4)	1470(3)
86(1)	520(37)	1468(8)	90(0)	533(59)	1470(7)
88(0)	524(1)	1470(15)	90(0)	545(21)	1477(33)
91(0)	534(15)	1474(18)	93(1)	555(35)	1485(2)
109(0)	551(184)	1755(10)	100(1)	558(9)	1796(20)
111(3)	564(1)	1756(16)	100(0)	574(49)	1797(23)
117(0)	584(123)	2005(67)	105(0)	600(187)	2004(1024)
130(5)	593(4)	2007(997)	118(0)	616(1)	2015(15)
132(0)	612(46)	2045(483)	123(0)	641(41)	2071(284)
147(0)	617(84)	2059(358)	124(0)	646(50)	2087(656)
152(0)	648(17)	2078(1897)	145(12)	647(39)	2107(2070)
167(0)	664(0)	2118(1113)	146(0)	661(25)	2137(908)
168(0)	866(1)	3021(47)	172(0)	870(0)	3052(6)
176(0)	867(0)	3022(65)	172(0)	870(1)	3052(4)
189(1)	931(2)	3029(72)	187(0)	935(0)	3055(40)
227(0)	933(1)	3029(41)	187(0)	936(0)	3056(63)
261(0)	945(1)	3106(9)	265(1)	939(0)	3123(3)
270(1)	946(0)	3107(10)	275(1)	940(0)	3123(0)
288(1)	1074(3)	3111(19)	288(1)	1083(2)	3126(38)
296(0)	1075(3)	3111(33)	299(0)	1084(0)	3126(0)
359(1)	1097(17)	3151(23)	373(2)	1107(43)	3184(19)
396(71)	1099(63)	3151(23)	395(119)	1109(59)	3184(2)
416(7)	1228(12)	3167(17)	425(0)	1224(7)	3185(2)
418(0)	1228(4)	3167(13)	428(3)	1225(4)	3185(13)

Table S151. Harmonic vibrational frequencies (in cm^{-1}) and infrared intensities (in parentheses, in km/mol) for the $(Me_2C{=}N)_2Fe_2(CO)_6$ structure **2-6S-5**.

M06-L			B3PW91-D3		
27(2)	419(1)	1388(10)	-37(18)	423(2)	1398(24)
33(0)	446(0)	1389(12)	41(0)	461(0)	1399(16)
47(4)	458(0)	1402(3)	41(1)	467(1)	1405(1)
48(1)	465(0)	1405(72)	52(1)	473(0)	1406(87)
60(1)	470(3)	1440(14)	60(1)	487(7)	1449(21)
64(1)	478(0)	1443(0)	72(0)	495(0)	1452(0)
76(0)	494(0)	1445(20)	85(0)	509(6)	1455(23)
79(1)	497(10)	1447(7)	91(1)	514(0)	1458(8)
86(2)	502(2)	1453(3)	94(2)	517(3)	1464(3)
88(0)	511(1)	1458(16)	98(0)	531(1)	1470(12)
90(0)	513(9)	1471(1)	99(0)	533(14)	1480(6)
92(0)	549(27)	1475(19)	103(0)	566(0)	1486(19)
104(0)	554(1)	1663(42)	105(1)	574(24)	1688(51)
109(0)	570(53)	1674(3)	109(0)	597(62)	1699(11)
111(1)	608(12)	1933(7)	115(0)	621(8)	1939(26)
121(1)	623(28)	1941(1020)	119(2)	635(25)	1951(1103)
140(0)	639(96)	2007(1392)	144(1)	658(88)	2030(1555)
151(0)	662(11)	2012(609)	148(0)	676(13)	2036(664)
158(1)	766(3)	2044(1576)	149(4)	757(5)	2064(1648)
166(2)	832(0)	2090(941)	161(0)	832(0)	2110(922)
176(1)	870(4)	3038(0)	171(0)	863(3)	3057(4)
179(0)	947(4)	3038(31)	190(1)	947(1)	3057(7)
220(0)	947(2)	3040(62)	226(0)	949(1)	3065(4)
247(8)	956(1)	3043(14)	239(19)	957(2)	3065(17)
272(0)	966(1)	3121(9)	263(0)	962(1)	3127(5)
280(5)	988(0)	3121(8)	269(4)	996(0)	3127(4)
309(0)	1080(4)	3127(1)	321(0)	1084(22)	3137(8)
363(1)	1083(1)	3127(20)	361(2)	1089(0)	3138(1)
373(1)	1084(14)	3172(15)	379(2)	1092(0)	3186(5)
386(0)	1132(28)	3173(3)	394(1)	1141(28)	3186(0)
401(1)	1299(0)	3174(5)	408(1)	1296(1)	3187(1)
402(1)	1300(3)	3174(2)	411(0)	1298(3)	3187(1)

Table S152. Harmonic vibrational frequencies (in cm^{-1}) and infrared intensities (in parentheses, in km/mol) for the $(Me_2C{=}N)_2Fe_2(CO)_6$ structure **2-6T-6**.

M06-L			B3PW91-D3		
25(0)	416(2)	1368(12)	21(1)	411(3)	1398(16)
32(0)	417(8)	1376(11)	40(0)	429(29)	1398(10)
36(1)	430(3)	1378(0)	47(0)	444(0)	1404(4)
40(0)	440(8)	1380(7)	52(1)	453(12)	1406(64)
51(0)	445(2)	1450(1)	65(0)	456(0)	1452(15)
53(0)	451(7)	1451(14)	68(2)	472(0)	1457(38)
62(1)	462(1)	1455(4)	71(1)	492(5)	1457(4)
70(0)	466(9)	1459(0)	80(0)	497(4)	1459(1)
73(0)	480(7)	1460(7)	85(1)	502(4)	1467(4)
76(0)	487(27)	1465(9)	86(0)	518(0)	1469(6)
76(0)	502(13)	1475(12)	92(1)	530(12)	1479(7)
79(0)	517(3)	1476(4)	92(0)	551(131)	1482(14)
83(0)	532(19)	1721(51)	99(0)	555(27)	1665(25)
95(1)	549(8)	1767(41)	108(0)	565(5)	1681(1)
101(0)	563(14)	2018(480)	109(2)	566(84)	2019(634)
114(1)	574(84)	2025(545)	114(3)	589(7)	2020(29)
125(0)	576(87)	2039(630)	117(1)	600(72)	2034(1434)
134(1)	600(11)	2055(1152)	120(1)	606(32)	2040(593)
144(1)	617(77)	2074(1385)	150(0)	758(0)	2057(2150)
154(1)	682(61)	2113(837)	160(0)	840(4)	2113(846)
173(1)	840(11)	3030(35)	162(0)	857(5)	3055(3)
177(0)	859(6)	3032(33)	179(1)	952(5)	3055(2)
196(2)	921(0)	3036(72)	181(4)	953(5)	3059(27)
229(1)	926(1)	3041(28)	256(3)	957(1)	3059(14)
262(0)	929(1)	3113(14)	263(2)	964(0)	3127(6)
277(1)	964(6)	3119(2)	346(6)	1005(3)	3127(7)
296(3)	1078(0)	3120(31)	350(0)	1076(23)	3130(9)
329(3)	1079(1)	3135(11)	362(1)	1085(0)	3130(3)
333(0)	1082(54)	3154(22)	377(2)	1086(1)	3183(6)
384(10)	1089(16)	3158(23)	387(2)	1147(47)	3183(2)
405(2)	1208(5)	3160(21)	394(0)	1297(0)	3183(3)
410(4)	1246(2)	3173(20)	400(4)	1299(4)	3184(1)

Table S153. Harmonic vibrational frequencies (in cm^{-1}) and infrared intensities (in parentheses, in km/mol) for the $(Me_2C{=}N)_2Fe_2(CO)_6$ structure **2-6T-7**.

M06-L			B3PW91-D3		
17(0)	400(2)	1364(16)	4(1)	409(0)	1378(8)
26(1)	404(0)	1368(3)	7(1)	409(0)	1378(38)
29(0)	405(3)	1374(20)	7(0)	423(1)	1385(2)
33(0)	414(0)	1378(1)	10(0)	423(0)	1385(2)
39(0)	439(9)	1442(7)	17(0)	459(4)	1457(9)
42(0)	440(19)	1447(3)	18(2)	459(31)	1457(2)
43(1)	462(5)	1447(4)	34(1)	485(6)	1459(0)
52(0)	463(8)	1452(6)	34(0)	486(20)	1460(2)
60(0)	472(3)	1454(2)	38(0)	489(9)	1467(16)
66(0)	483(2)	1459(9)	41(1)	489(0)	1467(2)
73(0)	492(8)	1467(21)	56(0)	510(0)	1479(30)
74(1)	506(21)	1473(15)	56(4)	511(37)	1480(3)
80(0)	542(13)	1711(57)	78(0)	551(8)	1739(10)
82(0)	543(44)	1719(12)	78(0)	552(51)	1740(124)
88(0)	565(64)	2022(616)	83(0)	586(55)	2047(0)
89(0)	569(7)	2034(1482)	83(0)	586(4)	2047(2737)
105(0)	578(31)	2040(308)	91(0)	601(3)	2063(191)
108(0)	601(31)	2047(1389)	92(0)	601(68)	2064(1869)
112(0)	632(8)	2099(165)	101(0)	651(4)	2122(0)
121(0)	650(1)	2109(1053)	102(0)	651(11)	2125(1260)
142(1)	863(1)	3025(40)	148(5)	869(1)	3056(3)
172(1)	871(2)	3027(36)	149(0)	869(0)	3056(6)
173(0)	919(0)	3030(60)	174(0)	925(2)	3060(49)
179(2)	923(2)	3034(31)	174(0)	925(0)	3060(4)
224(2)	937(1)	3110(16)	216(5)	935(0)	3130(4)
235(9)	940(1)	3112(23)	216(0)	935(0)	3130(0)
272(1)	1073(2)	3116(23)	282(2)	1087(4)	3134(22)
284(1)	1077(3)	3121(10)	282(0)	1087(0)	3134(2)
341(0)	1092(28)	3159(18)	335(0)	1102(1)	3182(9)
343(1)	1098(51)	3165(23)	335(0)	1102(90)	3182(7)
388(3)	1232(4)	3169(12)	396(3)	1232(6)	3185(6)
395(3)	1234(8)	3173(14)	396(5)	1232(9)	3185(8)

Table S154. Harmonic vibrational frequencies (in cm^{-1}) and infrared intensities (in parentheses, in km/mol) for the $(Me_2C{=}N)_2Fe_2(CO)_6$ structure **2-6S-8**.

M06-L			B3PW91-D3		
25(1)	441(1)	1332(37)	21(1)	445(1)	1356(48)
29(0)	456(4)	1387(33)	27(0)	461(0)	1377(18)
45(0)	461(11)	1388(15)	43(0)	467(5)	1394(22)
48(1)	475(3)	1392(6)	48(0)	484(4)	1400(16)
57(0)	481(2)	1394(16)	57(0)	487(2)	1404(16)
67(0)	483(6)	1431(26)	68(0)	494(2)	1436(24)
71(1)	492(1)	1448(16)	72(1)	504(3)	1455(8)
78(1)	505(5)	1452(1)	78(1)	515(5)	1463(2)
81(2)	513(17)	1456(1)	83(1)	521(4)	1469(6)
82(1)	519(9)	1465(40)	87(0)	527(19)	1471(9)
90(1)	540(13)	1470(7)	92(2)	548(21)	1482(7)
93(3)	560(20)	1478(16)	95(1)	569(20)	1485(35)
94(0)	570(21)	1481(4)	98(0)	580(21)	1492(4)
107(6)	593(35)	1593(60)	105(0)	606(46)	1654(21)
121(1)	602(51)	1987(1055)	120(1)	612(59)	2002(1239)
135(3)	624(52)	2008(543)	130(1)	640(34)	2027(684)
144(1)	626(39)	2049(909)	140(0)	645(55)	2079(925)
159(3)	655(48)	2054(814)	167(1)	666(38)	2086(743)
181(2)	746(8)	2074(1246)	170(2)	759(7)	2094(975)
196(0)	835(7)	2113(703)	191(1)	828(8)	2137(783)
212(1)	877(6)	2852(31)	198(5)	873(4)	2822(6)
225(1)	934(2)	3013(21)	222(1)	928(1)	3039(15)
237(1)	945(0)	3017(35)	240(1)	944(0)	3048(15)
262(1)	980(4)	3031(12)	261(0)	974(4)	3057(8)
300(0)	984(2)	3033(26)	301(0)	986(1)	3089(6)
302(1)	1026(2)	3100(18)	312(2)	1027(5)	3117(4)
335(12)	1050(5)	3105(10)	345(7)	1060(5)	3120(15)
370(1)	1072(16)	3113(12)	371(1)	1079(13)	3127(7)
393(2)	1088(34)	3132(23)	401(1)	1093(35)	3145(9)
397(1)	1186(18)	3142(21)	407(1)	1193(39)	3151(15)
420(0)	1233(6)	3150(20)	427(0)	1226(10)	3164(11)
431(0)	1306(21)	3152(14)	442(1)	1305(3)	3175(4)

Table S155. Harmonic vibrational frequencies (in cm^{-1}) and infrared intensities (in parentheses, in km/mol) for the $(Me_2C{=}N)_2Fe_2(CO)_6$ structure **2-6T-9**.

M06-L			B3PW91-D3		
17(0)	409(12)	1340(39)	11(0)	420(26)	1357(8)
27(1)	418(2)	1361(2)	21(1)	436(3)	1387(49)
33(1)	430(5)	1393(17)	33(0)	445(6)	1398(27)
38(2)	435(14)	1396(20)	42(0)	455(1)	1404(40)
49(1)	441(20)	1398(10)	51(0)	465(1)	1407(15)
52(2)	450(23)	1429(17)	58(0)	470(28)	1435(18)
59(0)	455(9)	1445(4)	64(0)	474(22)	1455(5)
67(1)	461(6)	1454(2)	71(1)	483(14)	1466(1)
68(1)	478(3)	1456(3)	73(1)	486(7)	1468(4)
79(1)	495(6)	1460(0)	80(0)	508(4)	1471(1)
80(1)	522(13)	1474(8)	83(1)	534(9)	1486(10)
88(0)	527(13)	1488(4)	94(0)	539(5)	1502(5)
90(1)	533(8)	1535(17)	99(2)	550(22)	1568(16)
113(0)	538(46)	1662(1)	109(1)	552(18)	1688(4)
116(1)	551(48)	1992(1049)	119(0)	572(23)	2010(1129)
125(1)	565(19)	2002(1050)	131(1)	581(15)	2018(1130)
140(1)	593(45)	2040(992)	138(1)	609(57)	2064(1072)
172(1)	624(109)	2052(834)	164(1)	643(68)	2080(664)
177(1)	693(12)	2068(1406)	173(0)	703(4)	2093(772)
196(1)	806(4)	2107(759)	185(1)	802(23)	2129(770)
209(0)	852(1)	2871(30)	200(2)	853(1)	2787(49)
229(0)	932(0)	3009(34)	226(8)	929(2)	3043(13)
261(25)	947(1)	3010(24)	276(3)	947(0)	3049(19)
288(1)	955(2)	3030(32)	291(2)	963(0)	3051(12)
315(0)	965(3)	3070(34)	309(0)	970(3)	3076(9)
326(25)	984(2)	3103(13)	337(0)	986(1)	3122(4)
350(1)	1042(7)	3111(15)	344(1)	1051(17)	3125(10)
356(15)	1071(18)	3114(11)	347(1)	1078(73)	3135(9)
365(19)	1076(9)	3138(5)	356(2)	1083(4)	3160(1)
375(1)	1104(12)	3153(19)	375(40)	1115(9)	3169(0)
382(5)	1249(7)	3158(1)	395(15)	1243(7)	3174(13)
399(1)	1277(1)	3160(19)	408(1)	1272(1)	3179(10)

Table S156. Harmonic vibrational frequencies (in cm^{-1}) and infrared intensities (in parentheses, in km/mol) for the $(Me_2C{=}N)_2Cr_2(CO)_8$ structure **2-8S-1**.

M06-L			B3PW91-D3		
13(2)	400(48)	1232(0)	7(1)	424(2)	1233(0)
37(0)	404(3)	1233(4)	39(0)	433(7)	1234(6)
43(0)	409(17)	1376(1)	40(1)	439(7)	1383(1)
52(0)	428(17)	1376(18)	56(0)	450(27)	1383(24)
57(1)	431(93)	1377(0)	62(1)	452(51)	1389(11)
64(3)	431(14)	1378(0)	64(4)	457(12)	1389(1)
76(0)	436(6)	1449(50)	76(0)	465(82)	1458(42)
77(0)	442(3)	1449(0)	79(0)	471(1)	1459(0)
77(0)	456(0)	1455(16)	81(0)	473(12)	1469(19)
77(0)	458(4)	1455(0)	84(0)	486(10)	1469(2)
85(0)	467(0)	1469(5)	88(0)	496(3)	1480(11)
86(0)	498(1)	1469(2)	89(1)	501(1)	1480(6)
100(0)	503(1)	1478(14)	95(0)	528(4)	1486(8)
114(0)	519(6)	1479(5)	108(1)	535(5)	1486(9)
115(0)	525(8)	1705(33)	119(0)	538(9)	1726(31)
118(0)	535(0)	1706(0)	121(0)	552(0)	1729(3)
121(0)	560(0)	2002(3)	122(0)	574(3)	2028(38)
123(0)	561(2)	2012(195)	128(1)	575(5)	2036(235)
127(1)	574(0)	2016(1849)	133(1)	586(1)	2041(1745)
135(2)	616(15)	2018(1102)	138(1)	632(23)	2043(1570)
147(0)	623(103)	2025(0)	158(0)	644(18)	2050(34)
179(0)	624(5)	2042(2605)	185(3)	644(137)	2065(2560)
194(2)	642(114)	2073(1462)	191(1)	666(173)	2089(1452)
200(0)	648(376)	2115(0)	201(1)	673(421)	2137(26)
206(9)	663(0)	3038(23)	207(0)	681(0)	3057(12)
229(0)	665(0)	3038(1)	218(0)	692(0)	3057(1)
243(0)	849(0)	3040(105)	258(0)	849(1)	3062(27)
273(1)	851(35)	3040(0)	258(1)	850(32)	3062(2)
281(0)	926(0)	3127(11)	281(73)	931(4)	3129(1)
285(54)	927(8)	3127(21)	283(0)	931(0)	3129(9)
291(1)	962(0)	3128(15)	301(1)	966(6)	3146(18)
304(2)	962(2)	3128(16)	314(8)	966(0)	3146(4)
362(0)	1077(7)	3168(10)	385(3)	1083(7)	3172(16)
362(0)	1077(0)	3168(11)	390(0)	1083(0)	3172(6)
393(3)	1079(21)	3170(42)	417(1)	1086(23)	3191(15)
399(7)	1080(0)	3170(1)	419(1)	1087(2)	3191(0)

Table S157. Harmonic vibrational frequencies (in cm^{-1}) and infrared intensities (in parentheses, in km/mol) for the $(Me_2C{=}N)_2Cr_2(CO)_8$ structure **2-8T-2**.

M06-L			B3PW91-D3		
24(1)	394(2)	1230(8)	13(1)	408(2)	1227(5)
31(1)	396(2)	1231(0)	30(0)	414(0)	1228(1)
33(0)	405(1)	1375(29)	39(0)	418(2)	1381(1)
54(0)	408(15)	1375(0)	52(0)	429(1)	1381(32)
58(0)	409(9)	1379(2)	59(1)	431(5)	1386(14)
60(0)	411(50)	1379(9)	64(0)	436(11)	1386(2)
65(0)	416(0)	1450(51)	68(0)	440(12)	1458(68)
66(0)	423(2)	1450(1)	70(0)	449(7)	1460(0)
73(0)	425(7)	1462(13)	77(0)	455(53)	1469(16)
75(1)	435(25)	1462(4)	79(0)	464(4)	1469(4)
77(0)	457(0)	1471(6)	79(0)	471(0)	1482(4)
81(0)	459(0)	1472(2)	82(0)	490(4)	1482(19)
94(0)	476(1)	1472(15)	93(1)	492(0)	1490(8)
97(0)	487(1)	1472(18)	102(0)	522(4)	1490(9)
103(0)	515(0)	1683(119)	111(0)	526(1)	1708(136)
104(0)	517(3)	1691(10)	112(0)	544(0)	1728(4)
109(1)	533(1)	2001(449)	119(0)	547(1)	2030(1260)
118(1)	538(0)	2009(1067)	125(1)	565(6)	2037(668)
134(1)	567(7)	2018(49)	126(0)	576(2)	2039(161)
135(0)	573(70)	2020(1188)	138(2)	595(71)	2043(868)
147(0)	596(19)	2020(117)	146(0)	610(10)	2045(0)
160(0)	606(49)	2041(2648)	184(0)	626(33)	2074(2691)
166(0)	612(74)	2076(1702)	196(0)	633(163)	2114(1043)
168(1)	613(82)	2117(53)	197(0)	635(114)	2144(15)
207(0)	615(51)	3034(0)	201(0)	772(0)	3052(1)
207(0)	632(1)	3034(28)	208(1)	819(16)	3052(15)
240(4)	833(10)	3036(79)	235(0)	824(17)	3055(25)
245(0)	839(1)	3037(8)	243(6)	913(0)	3056(1)
260(1)	917(4)	3118(1)	263(5)	922(3)	3126(2)
262(15)	917(1)	3118(25)	266(1)	941(0)	3126(13)
287(0)	961(0)	3130(11)	356(21)	954(0)	3138(18)
294(19)	961(4)	3130(24)	359(0)	958(2)	3138(5)
350(3)	1069(3)	3151(38)	387(0)	1073(5)	3163(9)
357(0)	1073(0)	3151(14)	389(1)	1082(0)	3163(20)
377(23)	1078(0)	3169(39)	396(0)	1082(0)	3175(32)
380(2)	1078(0)	3169(0)	399(5)	1122(1)	3175(0)

Table S158. Harmonic vibrational frequencies (in cm^{-1}) and infrared intensities (in parentheses, in km/mol) for the $(Me_2C{=}N)_2Cr_2(CO)_8$ structure **2-8S-3**.

M06-L			B3PW91-D3		
7(1)	424(2)	1233(0)	7(1)	424(2)	1233(0)
39(0)	433(7)	1234(6)	39(0)	433(7)	1234(6)
40(1)	439(7)	1383(1)	40(1)	439(7)	1383(1)
56(0)	450(27)	1383(24)	56(0)	450(27)	1383(24)
62(1)	452(51)	1389(11)	62(1)	452(51)	1389(11)
64(4)	457(12)	1389(1)	64(4)	457(12)	1389(1)
76(0)	465(82)	1458(42)	76(0)	465(82)	1458(42)
79(0)	471(1)	1459(0)	79(0)	471(1)	1459(0)
81(0)	473(12)	1469(19)	81(0)	473(12)	1469(19)
84(0)	486(10)	1469(2)	84(0)	486(10)	1469(2)
88(0)	496(3)	1480(11)	88(0)	496(3)	1480(11)
89(1)	501(1)	1480(6)	89(1)	501(1)	1480(6)
95(0)	528(4)	1486(8)	95(0)	528(4)	1486(8)
108(1)	535(5)	1486(9)	108(1)	535(5)	1486(9)
119(0)	538(9)	1726(31)	119(0)	538(9)	1726(31)
121(0)	552(0)	1729(3)	121(0)	552(0)	1729(3)
122(0)	574(3)	2028(38)	122(0)	574(3)	2028(38)
128(1)	575(5)	2036(235)	128(1)	575(5)	2036(235)
133(1)	586(1)	2041(1745)	133(1)	586(1)	2041(1745)
138(1)	632(23)	2043(1570)	138(1)	632(23)	2043(1570)
158(0)	644(18)	2050(34)	158(0)	644(18)	2050(34)
185(3)	644(137)	2065(2560)	185(3)	644(137)	2065(2560)
191(1)	666(173)	2089(1452)	191(1)	666(173)	2089(1452)
201(1)	673(421)	2137(26)	201(1)	673(421)	2137(26)
207(0)	681(0)	3057(12)	207(0)	681(0)	3057(12)
218(0)	692(0)	3057(1)	218(0)	692(0)	3057(1)
258(0)	849(1)	3062(27)	258(0)	849(1)	3062(27)
258(1)	850(32)	3062(2)	258(1)	850(32)	3062(2)
281(73)	931(4)	3129(1)	281(73)	931(4)	3129(1)
283(0)	931(0)	3129(9)	283(0)	931(0)	3129(9)
301(1)	966(6)	3146(18)	301(1)	966(6)	3146(18)
314(8)	966(0)	3146(4)	314(8)	966(0)	3146(4)
385(3)	1083(7)	3172(16)	385(3)	1083(7)	3172(16)
390(0)	1083(0)	3172(6)	390(0)	1083(0)	3172(6)
417(1)	1086(23)	3191(15)	417(1)	1086(23)	3191(15)
419(1)	1087(2)	3191(0)	419(1)	1087(2)	3191(0)

Table S159. Harmonic vibrational frequencies (in cm^{-1}) and infrared intensities (in parentheses, in km/mol) for the $(Me_2C{=}N)_2Cr_2(CO)_8$ structure **2-8S-4**.

M06-L			B3PW91-D3		
36(1)	401(5)	1286(1)	27(1)	422(10)	1277(1)
45(0)	411(8)	1290(11)	46(0)	427(2)	1288(12)
53(0)	419(1)	1384(23)	54(0)	433(0)	1395(33)
60(0)	429(38)	1391(16)	58(0)	461(30)	1403(12)
62(1)	440(48)	1398(13)	68(0)	465(7)	1405(52)
68(0)	450(7)	1401(56)	70(0)	469(11)	1412(33)
74(0)	457(1)	1438(33)	76(0)	471(3)	1450(31)
79(0)	459(1)	1445(3)	81(0)	483(39)	1455(6)
81(0)	466(1)	1449(7)	85(0)	487(2)	1460(2)
82(1)	472(0)	1451(4)	86(1)	491(8)	1461(9)
87(0)	477(11)	1451(10)	91(0)	498(15)	1463(6)
87(0)	495(7)	1457(16)	95(0)	512(11)	1466(24)
93(1)	516(3)	1469(5)	96(0)	529(2)	1483(9)
96(1)	528(2)	1472(15)	103(1)	542(6)	1485(15)
99(1)	541(35)	1548(9)	107(0)	553(30)	1544(6)
106(8)	559(6)	1696(79)	111(10)	569(2)	1726(84)
111(0)	573(7)	1972(136)	117(1)	583(5)	1995(148)
118(0)	576(3)	1983(726)	124(1)	591(2)	2004(842)
129(5)	606(72)	1996(801)	143(9)	633(88)	2019(924)
154(1)	612(38)	2008(1595)	155(2)	634(56)	2031(1500)
161(0)	631(48)	2018(470)	163(0)	651(64)	2042(518)
168(1)	638(7)	2036(985)	169(1)	659(12)	2064(1014)
176(1)	667(125)	2064(1874)	175(1)	684(137)	2087(1836)
183(1)	687(29)	2109(397)	189(2)	709(41)	2133(414)
199(1)	720(6)	3025(28)	211(1)	732(11)	3047(8)
210(2)	832(2)	3031(33)	214(2)	838(7)	3055(17)
218(0)	840(5)	3039(13)	232(1)	841(3)	3057(8)
236(0)	926(6)	3041(34)	242(2)	937(1)	3066(10)
259(1)	944(1)	3116(7)	255(0)	948(0)	3129(5)
346(27)	949(3)	3121(12)	353(18)	955(3)	3136(1)
348(5)	959(2)	3122(10)	360(7)	971(3)	3138(4)
361(7)	985(2)	3128(8)	370(7)	995(1)	3139(6)
372(6)	1063(48)	3166(11)	380(10)	1070(36)	3182(4)
378(5)	1071(10)	3173(6)	384(4)	1080(33)	3190(6)
383(3)	1089(2)	3175(11)	409(3)	1098(3)	3191(2)
395(2)	1109(9)	3184(5)	414(3)	1119(9)	3201(3)

Table S160. Harmonic vibrational frequencies (in cm^{-1}) and infrared intensities (in parentheses, in km/mol) for the $(Me_2C{=}N)_2Cr_2(CO)_8$ structure **2-8S-5**.

M06-L			B3PW91-D3		
25(0)	416(3)	1251(0)	29(0)	432(5)	1251(1)
36(2)	427(10)	1288(6)	40(2)	445(2)	1285(6)
47(0)	434(3)	1374(10)	49(1)	450(10)	1383(18)
56(1)	438(42)	1378(17)	59(1)	457(1)	1389(21)
58(0)	445(17)	1386(7)	62(0)	466(8)	1394(15)
65(1)	455(4)	1392(35)	71(0)	473(3)	1397(33)
73(0)	461(0)	1437(58)	78(0)	483(27)	1445(58)
76(0)	466(1)	1437(29)	80(0)	486(7)	1448(30)
78(1)	468(11)	1444(12)	83(1)	489(12)	1455(10)
80(0)	481(1)	1449(7)	85(0)	503(0)	1458(10)
83(0)	488(3)	1460(1)	89(0)	507(18)	1475(1)
90(1)	495(3)	1463(1)	99(0)	515(5)	1480(4)
98(1)	503(13)	1472(13)	105(1)	518(15)	1484(15)
100(1)	532(12)	1474(15)	112(2)	549(13)	1486(15)
111(0)	545(17)	1631(169)	118(0)	568(4)	1658(215)
117(1)	562(19)	1648(82)	123(0)	577(24)	1669(77)
122(0)	582(5)	1799(332)	128(2)	602(3)	1792(354)
130(1)	604(47)	1941(363)	141(0)	627(32)	1954(513)
133(1)	615(44)	1988(216)	151(1)	639(58)	2007(517)
152(1)	622(10)	1993(1509)	161(1)	645(31)	2016(1300)
167(1)	637(69)	2010(730)	173(1)	657(94)	2031(792)
179(1)	657(15)	2029(984)	184(1)	678(4)	2051(1070)
181(1)	668(48)	2051(2040)	189(1)	694(47)	2078(1607)
193(2)	683(65)	2091(697)	199(2)	710(91)	2117(775)
208(0)	756(129)	3037(14)	210(0)	771(138)	3055(1)
222(0)	830(7)	3041(10)	215(0)	830(4)	3060(1)
247(0)	860(12)	3042(29)	256(2)	858(10)	3061(9)
261(2)	911(14)	3049(21)	271(3)	916(14)	3065(5)
271(1)	921(3)	3125(12)	282(0)	928(3)	3133(12)
284(1)	937(45)	3127(12)	292(1)	951(65)	3135(4)
293(6)	954(2)	3129(12)	309(5)	960(1)	3138(7)
339(5)	988(12)	3130(14)	350(3)	992(26)	3140(9)
376(14)	1079(1)	3172(13)	401(11)	1086(0)	3187(5)
399(12)	1080(0)	3172(11)	412(3)	1087(3)	3191(4)
405(4)	1085(8)	3186(4)	421(5)	1091(8)	3196(2)
410(3)	1100(7)	3188(7)	428(6)	1109(13)	3202(1)

Table S161. Harmonic vibrational frequencies (in cm^{-1}) and infrared intensities (in parentheses, in km/mol) for the $(Me_2C=N)_2Cr_2(CO)_8$ structure **2-8T-6**.

M06-L			B3PW91-D3		
24(1)	390(2)	1240(0)	25(1)	410(2)	1274(6)
26(1)	397(7)	1280(6)	37(0)	412(12)	1380(18)
36(1)	401(12)	1375(8)	54(0)	427(2)	1384(8)
46(0)	409(3)	1377(11)	57(0)	436(0)	1394(25)
53(0)	423(0)	1386(5)	58(2)	445(4)	1395(25)
55(1)	431(14)	1391(36)	60(1)	448(7)	1446(61)
60(0)	444(2)	1440(62)	68(1)	460(4)	1453(29)
66(1)	449(11)	1447(33)	70(1)	468(6)	1458(9)
67(1)	456(4)	1451(8)	79(0)	475(3)	1466(8)
75(0)	459(22)	1455(8)	82(0)	484(12)	1468(1)
78(0)	461(3)	1457(2)	86(0)	486(2)	1477(12)
84(0)	463(12)	1466(4)	91(1)	492(36)	1478(4)
87(1)	472(4)	1470(13)	93(1)	507(4)	1484(14)
97(1)	495(10)	1479(10)	103(1)	512(7)	1649(171)
101(1)	498(4)	1635(154)	106(1)	532(8)	1674(111)
111(1)	505(11)	1657(94)	115(1)	547(8)	1736(293)
116(1)	520(4)	1734(290)	121(0)	556(48)	1938(557)
120(2)	547(33)	1931(325)	132(1)	602(4)	2005(815)
135(1)	580(9)	1990(660)	160(0)	618(21)	2031(582)
158(2)	608(56)	2014(469)	171(2)	633(58)	2048(1106)
171(4)	612(26)	2024(1161)	173(2)	654(54)	2074(901)
174(3)	632(41)	2035(1618)	177(2)	678(28)	2076(1921)
177(1)	662(6)	2048(2144)	184(1)	708(85)	2136(872)
197(1)	681(73)	2103(837)	193(2)	796(79)	3053(5)
205(0)	783(102)	3033(18)	194(1)	824(3)	3059(13)
211(2)	822(3)	3036(35)	240(5)	845(6)	3062(1)
233(6)	850(13)	3043(12)	254(8)	914(8)	3068(10)
246(6)	911(9)	3049(28)	262(14)	923(1)	3123(8)
271(11)	914(4)	3119(17)	281(13)	950(66)	3133(10)
281(6)	939(57)	3126(15)	288(5)	954(2)	3138(11)
286(12)	961(1)	3127(18)	299(7)	989(50)	3139(12)
300(12)	984(10)	3131(16)	332(10)	1078(4)	3179(8)
322(14)	1077(0)	3166(7)	345(6)	1083(0)	3196(3)
347(12)	1079(3)	3169(23)	365(9)	1086(8)	3198(7)
352(2)	1081(4)	3182(8)	392(7)	1104(19)	3206(5)
377(24)	1099(8)	3185(11)	25(1)	410(2)	1274(6)

Table S162. Harmonic vibrational frequencies (in cm^{-1}) and infrared intensities (in parentheses, in km/mol) for the $(Me_2C{=}N)_2Cr_2(CO)_8$ structure **2-8S-7**.

M06-L			B3PW91-D3		
37(1)	409(0)	1307(4)	27(2)	421(1)	1303(0)
37(0)	414(1)	1307(0)	33(1)	424(1)	1305(6)
50(1)	432(49)	1382(10)	36(0)	429(0)	1386(0)
55(0)	433(49)	1382(1)	48(1)	448(0)	1387(2)
62(1)	436(5)	1394(11)	57(0)	450(0)	1400(17)
67(1)	442(0)	1394(0)	65(1)	469(21)	1401(1)
73(0)	446(0)	1419(0)	67(1)	473(43)	1419(5)
76(1)	469(4)	1421(42)	74(1)	482(39)	1420(55)
76(2)	469(7)	1450(36)	74(0)	489(7)	1457(37)
83(2)	471(1)	1452(3)	75(1)	489(0)	1460(2)
86(0)	489(6)	1465(1)	81(3)	500(9)	1470(2)
87(0)	490(1)	1466(45)	86(1)	501(1)	1471(40)
89(1)	542(1)	1504(21)	88(0)	548(3)	1515(3)
95(3)	555(5)	1505(1)	91(1)	568(4)	1517(19)
95(0)	558(2)	1685(32)	93(0)	571(3)	1715(37)
108(0)	568(0)	1693(0)	98(1)	574(2)	1725(0)
113(0)	579(5)	1962(0)	102(2)	591(4)	1992(9)
117(0)	582(2)	1967(1774)	103(0)	593(0)	1992(1460)
121(0)	626(93)	1988(1084)	105(0)	647(105)	2006(1295)
127(2)	630(25)	1991(1927)	124(0)	650(6)	2008(1980)
130(1)	641(55)	2011(1513)	124(1)	663(41)	2029(1732)
139(1)	643(34)	2016(72)	134(1)	664(68)	2034(220)
154(1)	702(17)	2084(379)	134(0)	723(72)	2107(291)
162(2)	702(60)	2098(446)	141(0)	724(31)	2119(454)
163(2)	746(7)	2944(29)	147(0)	744(7)	2924(16)
174(0)	856(5)	2944(5)	155(0)	852(4)	2924(15)
187(1)	862(1)	3042(16)	165(0)	854(1)	3065(3)
233(0)	924(1)	3043(14)	220(0)	937(0)	3065(1)
270(0)	929(4)	3091(26)	269(1)	937(6)	3098(8)
343(0)	971(1)	3091(11)	332(2)	970(2)	3098(7)
356(1)	974(1)	3127(4)	356(2)	970(1)	3137(3)
368(4)	1004(1)	3127(23)	381(0)	1012(0)	3137(9)
369(1)	1069(13)	3142(7)	381(0)	1074(1)	3144(0)
383(1)	1072(3)	3142(9)	403(0)	1077(0)	3144(1)
383(5)	1080(1)	3175(0)	409(5)	1079(6)	3193(7)
397(0)	1142(4)	3176(17)	410(1)	1151(5)	3193(0)

Table S163. Harmonic vibrational frequencies (in cm^{-1}) and infrared intensities (in parentheses, in km/mol) for the $(Me_2C{=}N)_2Cr_2(CO)_8$ structure **2-8T-8**.

M06-L			B3PW91-D3		
37(0)	386(1)	1299(3)	17(1)	413(8)	1293(1)
40(3)	400(1)	1300(0)	42(1)	419(5)	1296(3)
46(1)	403(0)	1393(18)	50(0)	426(5)	1397(20)
59(2)	416(1)	1393(6)	53(1)	429(2)	1398(25)
63(0)	417(7)	1404(9)	56(1)	430(2)	1401(11)
67(1)	419(2)	1407(63)	68(0)	441(4)	1405(32)
69(0)	427(0)	1446(28)	71(0)	452(5)	1451(44)
75(0)	434(10)	1449(7)	72(1)	464(5)	1456(11)
76(5)	438(15)	1452(42)	81(0)	468(9)	1459(18)
83(1)	441(0)	1454(1)	83(2)	471(8)	1464(9)
84(0)	447(26)	1460(6)	85(2)	486(41)	1470(10)
90(1)	461(1)	1462(12)	90(1)	490(38)	1473(7)
90(0)	467(0)	1473(8)	94(3)	498(41)	1482(13)
91(1)	487(1)	1476(15)	97(1)	507(1)	1483(26)
95(1)	509(2)	1657(19)	100(2)	523(9)	1677(24)
110(1)	536(1)	1670(1)	105(1)	525(3)	1697(19)
115(0)	538(5)	1959(78)	109(0)	560(34)	1910(291)
117(0)	561(1)	1977(2849)	120(0)	569(4)	1976(294)
125(2)	566(68)	1984(1695)	125(1)	574(6)	2008(1150)
131(0)	593(73)	1998(0)	128(1)	596(26)	2018(1548)
133(0)	594(23)	2006(1531)	132(3)	609(75)	2026(400)
162(0)	603(5)	2015(2165)	152(1)	641(62)	2036(1158)
165(0)	613(63)	2016(1123)	170(15)	661(53)	2064(2405)
185(1)	629(56)	2097(563)	180(0)	689(125)	2114(469)
190(0)	752(4)	3037(3)	181(4)	748(1)	3061(2)
200(0)	836(1)	3037(22)	189(5)	835(0)	3061(1)
200(0)	856(5)	3038(60)	200(13)	853(2)	3065(5)
231(0)	944(2)	3040(17)	224(1)	945(3)	3065(15)
241(1)	952(3)	3120(14)	234(0)	949(3)	3131(3)
358(1)	955(2)	3120(18)	298(27)	954(1)	3133(1)
360(20)	962(0)	3122(11)	357(3)	967(1)	3137(7)
362(0)	989(2)	3123(5)	361(1)	997(1)	3139(11)
366(109)	1074(15)	3172(14)	375(8)	1076(10)	3190(6)
375(42)	1084(0)	3173(1)	386(5)	1087(1)	3190(0)
382(2)	1084(5)	3185(4)	395(13)	1090(0)	3193(4)
383(32)	1127(28)	3185(4)	408(2)	1136(22)	3199(2)

Table S164. Harmonic vibrational frequencies (in cm^{-1}) and infrared intensities (in parentheses, in km/mol) for the $(Me_2C{=}N)_2Cr_2(CO)_8$ structure **2-8S-9**.

M06-L			B3PW91-D3		
38(2)	386(2)	1277(1)	41(0)	408(6)	1266(1)
44(0)	400(1)	1288(9)	47(1)	412(13)	1283(9)
47(0)	407(16)	1384(27)	53(0)	422(8)	1392(34)
49(1)	412(9)	1393(4)	55(0)	428(6)	1399(9)
57(1)	415(10)	1398(23)	61(0)	432(7)	1403(53)
62(0)	416(4)	1400(45)	74(0)	437(20)	1407(22)
69(1)	423(7)	1417(29)	75(1)	443(9)	1426(36)
70(1)	429(4)	1447(4)	77(0)	460(15)	1457(5)
74(0)	442(32)	1450(6)	79(0)	466(5)	1460(1)
80(0)	448(5)	1451(15)	82(1)	472(15)	1461(8)
83(0)	454(12)	1455(5)	86(1)	480(11)	1463(9)
85(1)	467(6)	1459(7)	89(1)	487(65)	1467(18)
86(0)	472(13)	1467(5)	92(1)	497(21)	1481(6)
93(2)	502(19)	1475(9)	97(2)	516(8)	1483(17)
103(0)	515(3)	1505(7)	102(1)	524(12)	1510(7)
108(5)	517(8)	1695(79)	112(1)	528(13)	1722(87)
115(0)	525(9)	1948(188)	115(6)	538(9)	1949(354)
122(1)	539(5)	1953(365)	127(0)	552(7)	1973(367)
140(17)	553(16)	1996(1279)	145(3)	565(4)	2019(1168)
148(3)	575(23)	2013(1408)	161(10)	590(24)	2037(1174)
157(4)	591(134)	2019(750)	164(5)	610(131)	2040(754)
166(0)	609(45)	2033(807)	170(2)	643(60)	2059(876)
177(0)	611(37)	2047(2748)	177(12)	649(62)	2078(2401)
188(1)	643(67)	2106(450)	191(3)	674(119)	2127(445)
213(3)	719(5)	3017(37)	217(4)	717(16)	3042(12)
222(1)	829(5)	3025(54)	220(3)	830(10)	3051(28)
234(0)	834(9)	3038(15)	232(1)	834(2)	3058(7)
241(3)	918(5)	3040(33)	242(2)	928(1)	3064(12)
264(4)	944(4)	3115(9)	263(4)	940(1)	3127(3)
312(6)	949(2)	3117(8)	328(23)	953(2)	3133(6)
344(27)	962(1)	3119(12)	352(15)	961(3)	3134(0)
352(2)	993(1)	3126(8)	357(3)	997(1)	3137(7)
359(16)	1054(62)	3159(13)	368(7)	1058(42)	3185(4)
368(5)	1068(24)	3172(8)	378(16)	1075(51)	3186(7)
377(4)	1088(2)	3175(11)	389(19)	1096(2)	3193(3)
380(12)	1103(7)	3200(4)	397(5)	1111(9)	3205(3)

Table S165. Harmonic vibrational frequencies (in cm^{-1}) and infrared intensities (in parentheses, in km/mol) for the $(Me_2C{=}N)_2Cr_2(CO)_8$ structure **2-8T-10**.

M06-L			B3PW91-D3		
29(1)	384(3)	1303(1)	24(1)	392(5)	1298(0)
37(0)	388(0)	1307(2)	30(1)	404(0)	1302(3)
45(1)	395(1)	1386(2)	38(1)	411(2)	1382(2)
47(1)	409(6)	1391(4)	42(0)	419(9)	1397(9)
59(0)	416(2)	1398(8)	49(1)	424(0)	1402(11)
61(1)	425(26)	1401(18)	54(0)	430(0)	1405(31)
63(1)	428(1)	1418(30)	61(2)	443(18)	1422(32)
69(1)	432(48)	1445(4)	66(1)	450(0)	1452(9)
72(1)	442(3)	1452(11)	71(1)	457(6)	1455(5)
74(0)	446(2)	1453(23)	73(1)	467(9)	1458(21)
77(0)	469(2)	1457(6)	78(1)	478(40)	1462(10)
84(0)	473(3)	1465(17)	80(0)	492(3)	1470(21)
88(1)	489(4)	1471(23)	84(0)	500(6)	1480(20)
91(1)	517(18)	1505(15)	90(2)	533(6)	1528(12)
94(1)	528(10)	1667(25)	91(2)	536(4)	1690(27)
99(1)	530(17)	1687(10)	98(2)	554(45)	1715(11)
101(1)	557(3)	1966(1368)	102(1)	570(3)	1986(1307)
106(1)	572(0)	1977(560)	105(1)	581(2)	2001(903)
110(0)	582(5)	1986(1641)	107(0)	594(3)	2004(1622)
118(1)	600(34)	2005(1088)	113(1)	615(38)	2022(1224)
137(1)	618(74)	2012(991)	121(0)	632(70)	2030(332)
142(1)	633(45)	2021(823)	129(0)	649(47)	2036(1469)
156(0)	644(41)	2082(373)	138(0)	665(52)	2104(258)
159(1)	702(40)	2095(431)	138(1)	723(52)	2116(465)
196(1)	742(4)	2940(12)	148(0)	739(4)	2903(9)
198(0)	850(2)	3031(19)	163(0)	844(1)	3051(7)
217(0)	859(4)	3040(15)	179(1)	853(3)	3065(3)
223(0)	932(2)	3043(17)	212(0)	938(3)	3070(3)
255(0)	941(2)	3087(21)	248(0)	940(1)	3097(6)
316(1)	961(2)	3120(11)	312(1)	965(2)	3128(2)
345(1)	970(1)	3122(8)	346(3)	966(2)	3132(5)
350(1)	991(1)	3131(15)	355(1)	997(1)	3144(0)
364(1)	1072(0)	3135(2)	365(2)	1075(1)	3147(8)
371(7)	1077(4)	3147(11)	381(1)	1079(8)	3156(4)
374(3)	1081(11)	3171(14)	385(10)	1087(3)	3190(7)
379(2)	1128(11)	3176(6)	388(3)	1136(10)	3198(3)

Table S166. Harmonic vibrational frequencies (in cm^{-1}) and infrared intensities (in parentheses, in km/mol) for the $(Me_2C{=}N)_2Cr_2(CO)_8$ structure **2-8T-11**.

M06-L			B3PW91-D3		
34(1)	391(4)	1280(4)	28(4)	419(10)	1278(7)
39(1)	394(7)	1288(8)	35(1)	428(3)	1286(7)
45(0)	402(11)	1388(2)	46(1)	434(7)	1396(15)
53(0)	408(14)	1395(15)	48(1)	438(8)	1396(24)
60(1)	413(12)	1399(25)	55(1)	443(14)	1397(29)
62(0)	419(1)	1400(62)	59(1)	456(4)	1400(50)
66(1)	425(2)	1440(4)	64(1)	457(7)	1451(30)
70(0)	439(6)	1445(6)	66(1)	466(15)	1453(10)
72(1)	441(7)	1449(10)	71(0)	470(9)	1456(10)
75(0)	452(4)	1451(13)	77(1)	478(12)	1457(12)
79(0)	463(12)	1456(13)	81(0)	481(14)	1464(32)
83(0)	470(19)	1460(36)	86(0)	494(9)	1465(12)
84(0)	476(4)	1465(9)	88(0)	502(25)	1476(10)
93(0)	485(7)	1472(10)	93(0)	522(31)	1477(13)
106(1)	509(4)	1559(183)	95(0)	551(20)	1593(352)
109(1)	524(19)	1644(34)	100(1)	556(58)	1670(167)
114(0)	551(41)	1677(72)	106(1)	587(19)	1696(182)
120(0)	555(66)	1739(289)	109(1)	595(13)	1742(297)
120(1)	565(13)	1991(322)	118(0)	607(41)	1996(904)
130(2)	582(66)	2013(118)	134(1)	620(44)	2010(179)
142(0)	609(12)	2021(2040)	150(0)	652(12)	2036(1033)
161(0)	616(14)	2035(1346)	159(2)	653(47)	2051(1033)
166(1)	741(54)	2040(2332)	171(2)	788(155)	2077(1538)
182(0)	791(16)	2103(1151)	172(1)	798(53)	2111(827)
199(1)	795(54)	3026(8)	179(1)	819(16)	3051(3)
213(4)	841(6)	3028(91)	221(0)	835(7)	3058(23)
254(9)	888(7)	3032(23)	247(13)	909(51)	3058(2)
257(2)	934(1)	3038(51)	259(0)	933(2)	3070(10)
267(0)	939(11)	3098(12)	273(3)	940(71)	3120(4)
283(7)	947(1)	3103(17)	286(9)	947(6)	3124(9)
299(2)	962(0)	3114(13)	304(1)	972(14)	3127(6)
350(32)	1005(55)	3119(10)	368(10)	1018(89)	3142(10)
366(15)	1074(3)	3157(16)	389(2)	1086(2)	3181(6)
373(23)	1081(0)	3158(14)	398(4)	1086(5)	3193(5)
377(30)	1098(4)	3166(8)	401(3)	1102(7)	3195(3)
387(2)	1149(4)	3185(2)	405(8)	1172(6)	3199(1)

Table S167. Harmonic vibrational frequencies (in cm^{-1}) and infrared intensities (in parentheses, in km/mol) for the $(Me_2C{=}N)_2Cr_2(CO)_8$ structure **2-8S-12**.

M06-L			B3PW91-D3		
16(1)	386(13)	1216(6)	7(1)	399(12)	1207(4)
20(1)	395(36)	1253(11)	11(4)	406(11)	1237(53)
28(0)	398(81)	1322(4)	19(1)	416(57)	1267(11)
36(0)	406(5)	1363(1)	25(1)	426(13)	1371(6)
42(1)	412(2)	1366(11)	34(1)	428(19)	1377(6)
44(1)	421(18)	1376(9)	39(1)	436(9)	1382(15)
51(0)	424(10)	1377(25)	43(4)	447(32)	1383(16)
58(0)	442(51)	1438(14)	45(1)	467(50)	1447(8)
62(3)	445(80)	1441(2)	53(1)	472(64)	1458(3)
63(1)	453(9)	1445(20)	55(0)	476(30)	1459(6)
68(1)	472(0)	1450(7)	62(1)	488(2)	1460(1)
70(1)	484(13)	1459(10)	69(6)	495(13)	1470(16)
72(2)	494(10)	1460(11)	70(0)	496(3)	1471(15)
77(0)	497(10)	1465(12)	73(3)	514(27)	1478(7)
80(1)	512(6)	1619(215)	79(0)	522(14)	1637(222)
83(0)	516(5)	1766(51)	81(10)	533(5)	1788(161)
85(0)	523(1)	1987(1021)	87(1)	543(0)	2016(1165)
89(0)	547(15)	2000(572)	89(1)	558(18)	2040(2724)
94(1)	555(2)	2023(533)	91(0)	569(17)	2047(1078)
102(1)	562(13)	2030(1580)	92(2)	573(3)	2050(217)
108(0)	577(21)	2038(1703)	94(1)	608(38)	2059(2870)
135(7)	606(55)	2045(1056)	96(0)	634(88)	2063(1872)
144(0)	627(40)	2067(3769)	104(2)	653(69)	2085(4000)
155(1)	638(143)	2112(212)	120(75)	655(70)	2131(87)
164(2)	642(250)	2632(10)	145(3)	666(549)	2525(78)
172(0)	650(27)	3013(28)	148(2)	680(91)	3051(13)
176(9)	860(4)	3031(18)	178(15)	861(27)	3055(1)
252(1)	864(16)	3039(51)	246(3)	884(1)	3059(36)
259(3)	899(1)	3081(18)	263(1)	923(63)	3115(3)
268(4)	928(0)	3099(16)	271(7)	931(0)	3121(6)
291(3)	943(2)	3126(9)	279(1)	940(0)	3131(0)
327(2)	944(1)	3128(13)	314(6)	951(4)	3135(11)
366(1)	1049(9)	3156(16)	380(3)	1066(6)	3181(8)
369(7)	1072(0)	3171(11)	381(64)	1081(0)	3184(3)
372(4)	1092(43)	3173(8)	393(94)	1095(50)	3185(2)
379(4)	1102(8)	3179(6)	397(2)	1097(20)	3206(4)

Table S168. Harmonic vibrational frequencies (in cm^{-1}) and infrared intensities (in parentheses, in km/mol) for the $(Me_2C{=}N)_2Cr_2(CO)_8$ structure **2-8S-13**.

M06-L			B3PW91-D3		
33(0)	404(2)	1284(3)	26(0)	423(0)	1275(1)
34(4)	415(0)	1284(17)	32(4)	426(8)	1276(13)
45(1)	420(0)	1388(1)	36(0)	431(0)	1393(18)
51(0)	433(8)	1391(10)	48(1)	446(2)	1394(14)
55(1)	434(0)	1395(2)	54(0)	448(3)	1398(82)
61(0)	435(6)	1396(70)	62(0)	450(0)	1398(2)
68(0)	450(2)	1447(10)	65(1)	458(1)	1449(7)
73(1)	451(0)	1448(0)	73(3)	459(2)	1449(11)
76(1)	459(4)	1450(49)	73(0)	479(3)	1457(10)
78(1)	460(1)	1451(24)	79(0)	480(0)	1458(42)
79(1)	479(10)	1464(22)	80(2)	501(4)	1462(7)
86(1)	488(19)	1468(2)	87(1)	511(8)	1463(21)
92(0)	500(9)	1480(8)	89(0)	522(21)	1477(21)
95(1)	502(10)	1484(11)	95(0)	522(12)	1477(9)
109(2)	527(55)	1663(116)	97(1)	548(44)	1693(189)
109(0)	547(17)	1663(101)	101(0)	567(2)	1694(120)
116(0)	557(97)	1717(359)	105(0)	579(91)	1729(414)
122(0)	570(10)	1727(115)	107(0)	597(26)	1738(165)
128(0)	575(33)	2004(65)	113(1)	600(79)	2030(24)
142(1)	581(13)	2014(439)	113(2)	606(12)	2033(279)
148(5)	623(18)	2024(1415)	119(0)	648(20)	2044(1674)
166(2)	623(61)	2050(902)	150(1)	649(68)	2071(1127)
177(3)	718(61)	2065(2045)	150(3)	726(97)	2088(2086)
192(4)	740(157)	2101(932)	155(1)	745(183)	2123(734)
193(2)	835(11)	3033(1)	180(5)	835(9)	3055(1)
238(4)	836(8)	3034(20)	222(6)	835(3)	3055(0)
242(1)	918(0)	3038(88)	246(1)	915(2)	3059(15)
249(1)	920(2)	3038(8)	255(0)	918(13)	3060(27)
252(0)	936(19)	3111(8)	265(3)	938(35)	3123(7)
276(0)	937(15)	3114(9)	286(1)	939(38)	3123(5)
286(9)	972(15)	3121(1)	288(12)	959(16)	3126(5)
296(9)	974(36)	3121(40)	301(6)	961(31)	3126(12)
364(80)	1075(1)	3167(2)	391(82)	1086(6)	3192(4)
382(2)	1081(3)	3167(26)	396(16)	1086(0)	3192(5)
387(2)	1123(0)	3181(2)	398(2)	1123(0)	3202(1)
390(15)	1123(1)	3182(7)	408(27)	1124(0)	3202(0)

Table S169. Harmonic vibrational frequencies (in cm^{-1}) and infrared intensities (in parentheses, in km/mol) for the $(Me_2C{=}N)_2Fe_2(CO)_5$ structure **2-5S-1**.

M06-L			B3PW91-D3		
40(2)	453(2)	1383(1)	36(2)	461(3)	1390(27)
42(0)	459(1)	1384(2)	39(1)	471(1)	1391(19)
47(0)	464(1)	1385(25)	49(0)	481(1)	1391(7)
58(0)	471(4)	1446(2)	55(0)	491(4)	1455(8)
63(0)	475(3)	1449(5)	63(0)	493(4)	1458(7)
67(0)	481(11)	1452(5)	68(0)	499(9)	1461(5)
71(1)	499(2)	1455(8)	73(1)	521(7)	1462(13)
79(0)	510(5)	1459(16)	82(0)	530(1)	1466(12)
81(0)	525(8)	1460(3)	87(0)	543(10)	1469(1)
84(0)	555(22)	1467(8)	88(0)	573(22)	1481(9)
87(0)	564(34)	1471(5)	90(0)	585(35)	1483(8)
88(0)	583(91)	1707(89)	93(1)	600(86)	1735(100)
97(1)	596(41)	1730(5)	97(1)	613(69)	1761(7)
102(1)	609(18)	2008(611)	116(0)	628(32)	2038(645)
125(1)	634(63)	2029(852)	128(0)	652(67)	2056(885)
141(0)	680(4)	2039(701)	132(1)	700(3)	2067(716)
146(1)	721(60)	2052(1767)	140(1)	744(76)	2078(1949)
159(2)	867(5)	2099(596)	166(2)	865(3)	2124(576)
180(0)	872(3)	3032(20)	190(0)	870(4)	3050(8)
228(1)	923(2)	3033(38)	236(1)	933(4)	3052(13)
244(0)	939(3)	3035(83)	248(0)	941(0)	3055(34)
279(0)	944(0)	3038(46)	291(1)	943(3)	3058(25)
286(1)	952(1)	3113(1)	297(2)	952(1)	3121(3)
309(1)	1078(0)	3114(20)	320(1)	1086(0)	3123(7)
328(3)	1082(1)	3115(28)	337(4)	1088(1)	3124(15)
335(5)	1090(41)	3121(17)	343(6)	1094(40)	3132(10)
407(5)	1096(13)	3165(16)	420(4)	1099(16)	3178(8)
431(4)	1247(0)	3169(13)	442(4)	1247(0)	3185(7)
431(3)	1249(0)	3170(14)	446(6)	1250(1)	3186(7)
444(3)	1383(7)	3171(14)	455(6)	1389(3)	3188(7)

Table S170. Harmonic vibrational frequencies (in cm^{-1}) and infrared intensities (in parentheses, in km/mol) for the $(Me_2C{=}N)_2Fe_2(CO)_5$ structure **2-5T-2**.

M06-L			B3PW91-D3		
36(2)	426(7)	1382(3)	36(2)	461(3)	1390(27)
38(0)	429(1)	1383(1)	39(1)	471(1)	1391(19)
45(0)	450(0)	1384(36)	49(0)	481(1)	1391(7)
52(0)	456(0)	1446(0)	55(0)	491(4)	1455(8)
58(1)	458(17)	1447(2)	63(0)	493(4)	1458(7)
61(0)	463(2)	1450(13)	68(0)	499(9)	1461(5)
66(0)	475(13)	1450(1)	73(1)	521(7)	1462(13)
69(0)	476(0)	1458(25)	82(0)	530(1)	1466(12)
76(0)	478(22)	1459(2)	87(0)	543(10)	1469(1)
77(0)	501(64)	1467(2)	88(0)	573(22)	1481(9)
82(0)	513(11)	1467(15)	90(0)	585(35)	1483(8)
84(0)	514(0)	1716(163)	93(1)	600(86)	1735(100)
85(0)	555(42)	1724(27)	97(1)	613(69)	1761(7)
100(1)	593(55)	1981(1173)	116(0)	628(32)	2038(645)
102(0)	620(57)	2032(599)	128(0)	652(67)	2056(885)
133(0)	656(1)	2047(729)	132(1)	700(3)	2067(716)
135(2)	685(28)	2050(1635)	140(1)	744(76)	2078(1949)
145(2)	860(1)	2106(688)	166(2)	865(3)	2124(576)
160(1)	867(1)	3029(39)	190(0)	870(4)	3050(8)
195(1)	922(0)	3030(34)	236(1)	933(4)	3052(13)
228(0)	923(3)	3035(92)	248(0)	941(0)	3055(34)
260(0)	938(0)	3036(27)	291(1)	943(3)	3058(25)
261(2)	938(0)	3114(0)	297(2)	952(1)	3121(3)
286(4)	1083(2)	3114(1)	320(1)	1086(0)	3123(7)
293(2)	1083(0)	3115(5)	337(4)	1088(1)	3124(15)
321(1)	1089(29)	3116(59)	343(6)	1094(40)	3132(10)
361(3)	1094(7)	3154(19)	420(4)	1099(16)	3178(8)
362(7)	1241(0)	3154(19)	442(4)	1247(0)	3185(7)
405(17)	1243(0)	3171(16)	446(6)	1250(1)	3186(7)
407(6)	1381(2)	3171(15)	455(6)	1389(3)	3188(7)

Table S171. Harmonic vibrational frequencies (in cm^{-1}) and infrared intensities (in parentheses, in km/mol) for the $(Me_2C{=}N)_2Fe_2(CO)_5$ structure **2-5T-3**.

M06-L			B3PW91-D3		
35(0)	441(2)	1382(7)	14(0)	446(9)	1389(32)
40(1)	444(5)	1385(3)	39(1)	461(9)	1391(9)
42(0)	452(0)	1385(29)	43(0)	465(0)	1391(23)
51(1)	467(0)	1449(14)	53(1)	476(1)	1457(23)
57(0)	469(5)	1449(3)	62(0)	490(4)	1457(7)
64(0)	477(2)	1452(0)	66(0)	491(2)	1460(1)
76(0)	482(14)	1452(18)	70(0)	498(15)	1461(23)
79(0)	496(5)	1460(16)	83(0)	510(2)	1472(5)
84(0)	505(2)	1461(6)	86(1)	521(3)	1472(3)
87(0)	537(3)	1469(2)	88(0)	551(1)	1482(4)
91(1)	537(8)	1470(11)	90(0)	559(5)	1483(14)
100(0)	571(35)	1704(169)	97(0)	590(56)	1732(179)
105(0)	578(59)	1714(53)	103(0)	593(46)	1741(71)
132(0)	611(42)	2015(664)	127(0)	625(46)	2041(691)
134(0)	643(16)	2034(818)	131(0)	655(20)	2060(855)
146(0)	665(0)	2045(996)	142(0)	680(0)	2071(1045)
153(0)	693(8)	2064(1578)	148(0)	714(3)	2092(1515)
158(0)	854(4)	2105(500)	161(1)	852(3)	2128(486)
169(0)	862(2)	3028(27)	172(0)	860(1)	3048(5)
181(0)	927(0)	3028(35)	177(0)	931(2)	3048(14)
231(0)	927(1)	3035(62)	234(0)	932(1)	3054(28)
262(1)	937(0)	3035(33)	280(2)	940(0)	3055(14)
277(0)	937(2)	3113(2)	293(0)	940(1)	3122(2)
285(0)	1081(0)	3113(8)	297(0)	1087(0)	3122(5)
322(3)	1081(0)	3114(17)	334(5)	1088(0)	3126(11)
333(3)	1085(17)	3115(38)	341(4)	1091(18)	3126(15)
370(1)	1091(9)	3155(30)	372(1)	1096(10)	3168(15)
403(1)	1243(0)	3155(16)	408(1)	1244(1)	3168(12)
427(6)	1243(1)	3173(13)	419(1)	1244(1)	3183(6)
430(6)	1381(3)	3173(21)	443(1)	1389(3)	3183(16)

Table S172. Harmonic vibrational frequencies (in cm^{-1}) and infrared intensities (in parentheses, in km/mol) for the $(Me_2C{=}N)_2Fe_2(CO)_5$ structure **2-5T-4**.

M06-L			B3PW91-D3		
24(0)	430(6)	1374(6)	23(1)	439(5)	1383(15)
30(1)	447(4)	1380(5)	30(0)	454(2)	1387(16)
35(1)	449(20)	1382(16)	37(1)	456(11)	1388(15)
56(0)	454(8)	1444(12)	54(0)	464(11)	1451(7)
62(0)	460(10)	1445(10)	65(0)	470(14)	1455(33)
68(1)	469(7)	1450(15)	68(0)	483(4)	1456(14)
74(1)	479(13)	1453(7)	73(1)	493(4)	1459(9)
78(0)	488(6)	1454(11)	83(0)	502(16)	1462(7)
86(0)	503(27)	1460(7)	89(0)	525(9)	1471(4)
92(1)	519(3)	1468(6)	92(0)	527(14)	1482(7)
97(0)	532(17)	1473(8)	94(1)	564(13)	1483(11)
100(1)	556(37)	1605(65)	96(0)	575(41)	1628(91)
112(0)	572(15)	1705(86)	116(0)	583(10)	1723(108)
121(1)	592(71)	2000(654)	125(1)	608(71)	2026(734)
130(0)	605(53)	2034(1615)	132(1)	616(47)	2061(803)
144(0)	620(16)	2037(922)	139(0)	641(5)	2067(1606)
159(0)	696(30)	2040(1091)	155(0)	717(26)	2072(992)
177(1)	833(9)	2097(550)	180(1)	829(5)	2121(527)
184(0)	862(4)	3029(12)	183(0)	857(3)	3048(9)
192(0)	909(1)	3032(70)	186(1)	918(2)	3053(25)
240(0)	925(3)	3037(14)	237(1)	927(4)	3054(4)
252(0)	936(3)	3040(59)	249(1)	937(3)	3058(20)
273(1)	946(1)	3112(12)	273(2)	946(1)	3122(6)
297(2)	1069(6)	3117(16)	299(3)	1076(29)	3125(9)
313(3)	1071(31)	3117(11)	322(4)	1078(8)	3133(5)
351(5)	1081(0)	3124(18)	348(3)	1087(2)	3133(10)
362(4)	1088(11)	3166(16)	368(1)	1090(8)	3179(8)
395(1)	1228(9)	3172(16)	400(0)	1231(9)	3183(8)
411(3)	1243(0)	3173(17)	417(4)	1243(0)	3186(9)
425(2)	1372(27)	3186(11)	431(4)	1379(30)	3196(6)

Table S173. Harmonic vibrational frequencies (in cm^{-1}) and infrared intensities (in parentheses, in km/mol) for the $(Me_2C{=}N)_2Fe_2(CO)_5$ structure **2-5S-5**.

M06-L			B3PW91-D3		
26(1)	456(0)	1405(20)	27(0)	463(2)	1398(27)
40(1)	457(3)	1406(22)	41(1)	468(2)	1400(4)
49(1)	465(5)	1408(34)	51(1)	472(2)	1405(55)
58(0)	468(1)	1458(38)	60(0)	479(6)	1451(29)
64(1)	488(16)	1460(19)	67(1)	500(9)	1452(28)
87(2)	497(10)	1463(19)	88(1)	508(10)	1456(14)
89(1)	510(12)	1468(3)	90(2)	525(14)	1459(4)
92(1)	523(6)	1475(17)	95(1)	539(7)	1468(21)
99(0)	543(13)	1479(5)	101(0)	553(18)	1471(5)
103(1)	555(21)	1488(7)	105(1)	574(13)	1481(6)
107(0)	566(30)	1490(33)	108(0)	579(43)	1484(35)
117(1)	586(2)	1644(42)	115(0)	598(3)	1658(42)
130(0)	614(15)	1653(8)	131(0)	627(15)	1670(8)
133(1)	640(23)	1869(684)	134(1)	652(26)	1886(678)
141(0)	643(57)	1980(1121)	142(0)	659(58)	2000(1080)
159(0)	748(1)	1992(853)	158(0)	756(2)	2010(817)
179(1)	817(0)	2021(1081)	182(1)	830(0)	2041(1040)
190(0)	854(1)	2076(1349)	196(0)	864(1)	2095(1384)
198(0)	943(1)	3045(3)	198(1)	939(0)	3056(4)
209(0)	951(2)	3048(0)	207(0)	946(1)	3061(1)
228(3)	961(1)	3052(4)	234(1)	960(1)	3064(13)
234(2)	968(2)	3053(20)	244(3)	966(2)	3065(14)
257(1)	982(0)	3104(4)	266(2)	993(1)	3124(2)
312(36)	1082(11)	3111(5)	332(36)	1078(13)	3130(4)
362(1)	1092(3)	3116(5)	362(1)	1086(4)	3133(4)
381(4)	1095(2)	3119(12)	381(4)	1089(2)	3139(10)
387(1)	1124(26)	3166(4)	391(3)	1135(30)	3183(3)
398(7)	1286(2)	3172(5)	407(5)	1294(2)	3186(3)
424(6)	1291(4)	3176(3)	426(4)	1298(1)	3188(4)
438(2)	1401(19)	3182(2)	448(2)	1396(20)	3195(2)

Table S174. Harmonic vibrational frequencies (in cm^{-1}) and infrared intensities (in parentheses, in km/mol) for the $(Me_2C{=}N)_2Fe_2(CO)_5$ structure **2-5T-6**.

M06-L			B3PW91-D3		
31(0)	422(1)	1391(5)	24(0)	437(0)	1398(12)
38(1)	435(8)	1403(7)	38(1)	444(17)	1404(5)
45(0)	453(0)	1406(51)	49(1)	460(4)	1406(61)
48(1)	457(1)	1440(14)	57(0)	463(3)	1453(15)
66(1)	481(0)	1445(10)	60(1)	488(2)	1456(29)
69(1)	486(6)	1448(20)	67(1)	496(2)	1459(8)
75(1)	492(9)	1451(13)	74(2)	509(14)	1459(9)
77(1)	499(1)	1453(0)	79(1)	515(16)	1467(3)
83(0)	504(7)	1459(8)	83(1)	525(2)	1470(10)
86(1)	524(59)	1469(6)	86(0)	556(35)	1479(8)
91(1)	558(68)	1474(17)	88(1)	570(51)	1483(15)
97(1)	564(59)	1638(20)	108(0)	580(114)	1676(34)
112(1)	578(51)	1664(21)	110(2)	589(94)	1689(5)
117(0)	605(44)	1925(872)	115(1)	610(78)	1963(1265)
125(0)	614(11)	1990(660)	122(2)	628(9)	2011(972)
129(2)	762(1)	2003(715)	130(1)	753(2)	2025(594)
157(0)	828(6)	2030(1340)	149(0)	829(2)	2049(1760)
171(0)	864(3)	2076(1136)	164(0)	857(2)	2096(732)
181(1)	947(5)	3026(25)	172(0)	950(1)	3054(4)
190(0)	948(3)	3033(64)	175(1)	952(4)	3056(3)
198(0)	956(1)	3039(4)	194(0)	956(1)	3059(22)
231(1)	966(0)	3041(37)	219(1)	962(1)	3061(13)
253(2)	983(3)	3113(9)	255(0)	994(2)	3124(7)
354(8)	1075(14)	3115(15)	357(2)	1078(20)	3128(8)
360(1)	1080(7)	3124(9)	365(2)	1088(1)	3130(6)
370(5)	1083(3)	3125(13)	381(7)	1089(1)	3135(5)
383(1)	1126(47)	3142(14)	388(14)	1141(36)	3172(4)
412(11)	1299(1)	3167(11)	403(1)	1293(1)	3180(5)
417(6)	1302(2)	3171(5)	408(1)	1298(2)	3183(2)
421(1)	1388(10)	3173(7)	415(4)	1397(16)	3184(2)

Table S175. Harmonic vibrational frequencies (in cm^{-1}) and infrared intensities (in parentheses, in km/mol) for the $(Me_2C{=}N)_2Fe_2(CO)_5$ structure **2-5T-7**.

M06-L			B3PW91-D3		
28(1)	389(32)	1391(5)	25(0)	382(9)	1397(11)
32(1)	405(45)	1405(10)	29(0)	385(3)	1405(7)
35(0)	408(4)	1408(59)	31(0)	393(12)	1407(57)
53(5)	418(1)	1440(30)	41(1)	409(2)	1451(17)
56(2)	423(7)	1444(14)	53(0)	418(1)	1455(16)
58(2)	449(3)	1449(20)	56(1)	448(2)	1456(17)
60(1)	458(0)	1452(9)	62(0)	454(7)	1461(3)
72(0)	479(16)	1454(14)	67(0)	468(5)	1466(13)
78(2)	484(1)	1458(8)	70(1)	491(5)	1469(13)
93(1)	490(8)	1469(6)	85(2)	503(1)	1477(8)
97(1)	496(4)	1472(28)	90(0)	524(41)	1481(19)
106(1)	554(50)	1652(27)	98(1)	550(3)	1681(43)
112(0)	558(13)	1669(5)	102(1)	560(10)	1694(6)
115(1)	580(40)	1937(360)	103(0)	576(54)	1943(841)
120(1)	616(50)	1958(410)	109(2)	596(68)	1968(100)
131(1)	759(5)	1991(2277)	115(0)	749(2)	2039(1616)
140(3)	831(3)	2018(2651)	128(1)	826(2)	2043(2200)
149(3)	859(2)	2068(827)	137(0)	851(1)	2118(750)
158(2)	943(5)	3026(30)	147(0)	942(3)	3050(8)
167(5)	951(2)	3032(16)	173(0)	949(2)	3055(3)
187(0)	956(0)	3037(60)	180(2)	957(0)	3058(21)
208(2)	966(1)	3039(14)	204(5)	965(1)	3061(10)
242(6)	984(2)	3116(8)	221(1)	987(1)	3124(5)
318(9)	1077(11)	3118(17)	298(4)	1079(18)	3128(8)
331(6)	1080(5)	3119(12)	324(21)	1086(1)	3129(4)
346(68)	1083(3)	3125(8)	337(3)	1090(0)	3138(4)
359(2)	1128(46)	3139(15)	353(2)	1136(38)	3162(4)
362(56)	1302(1)	3154(14)	359(40)	1296(0)	3168(8)
364(11)	1303(3)	3169(8)	362(5)	1298(4)	3179(3)
384(3)	1390(15)	3172(8)	374(6)	1396(20)	3181(4)

Table S176. Harmonic vibrational frequencies (in cm^{-1}) and infrared intensities (in parentheses, in km/mol) for the $(Me_2C{=}N)_2Fe_2(CO)_5$ structure **2-5S-8**.

M06-L			B3PW91-D3		
26(1)	462(7)	1359(66)	17(1)	469(0)	1348(85)
34(1)	466(1)	1390(12)	34(1)	475(9)	1392(24)
45(0)	475(11)	1393(15)	46(1)	477(9)	1401(17)
59(0)	492(4)	1405(8)	63(0)	498(26)	1409(12)
69(1)	498(4)	1445(3)	71(0)	510(19)	1454(3)
74(0)	504(34)	1451(3)	77(1)	527(2)	1460(34)
81(1)	523(61)	1454(8)	82(0)	540(34)	1464(3)
82(1)	554(41)	1461(16)	87(1)	561(32)	1467(35)
87(1)	558(36)	1467(9)	92(1)	568(12)	1469(6)
93(1)	583(25)	1470(34)	100(1)	578(8)	1477(10)
103(0)	592(52)	1480(3)	115(2)	602(34)	1493(2)
119(8)	617(32)	1552(172)	127(2)	621(32)	1557(116)
124(1)	631(58)	1600(13)	136(1)	643(17)	1651(84)
146(1)	665(35)	2002(1142)	138(1)	654(63)	2001(1301)
161(1)	690(11)	2013(1213)	158(1)	673(25)	2022(963)
174(3)	705(4)	2021(844)	170(2)	708(25)	2050(1055)
184(0)	814(17)	2049(2330)	182(0)	808(50)	2074(1895)
220(3)	875(14)	2081(643)	202(1)	869(6)	2101(697)
229(1)	948(2)	2675(126)	232(6)	940(11)	2684(54)
257(5)	954(10)	2732(4)	246(5)	945(3)	2766(57)
290(3)	960(10)	2987(33)	286(4)	961(14)	3036(6)
317(3)	975(5)	2994(36)	315(4)	971(4)	3041(16)
348(1)	989(15)	3031(43)	344(13)	986(10)	3052(13)
352(37)	1050(29)	3097(17)	355(6)	1057(62)	3119(6)
368(4)	1061(19)	3103(10)	367(0)	1067(6)	3122(6)
403(19)	1068(27)	3110(16)	417(5)	1071(44)	3126(4)
410(18)	1125(9)	3148(19)	423(0)	1136(10)	3158(2)
421(26)	1258(12)	3152(8)	439(4)	1254(5)	3161(11)
440(32)	1282(29)	3155(19)	443(22)	1276(8)	3175(9)
451(4)	1298(28)	3166(6)	450(6)	1341(83)	3180(1)

Table S177. Harmonic vibrational frequencies (in cm^{-1}) and infrared intensities (in parentheses, in km/mol) for the $(Me_2C=N)_2Cr_2(CO)_7$ structure **2-7S-1**.

M06-L			B3PW91-D3		
22(0)	417(31)	1239(1)	29(1)	440(12)	1238(1)
41(0)	419(59)	1376(16)	42(0)	449(14)	1384(21)
44(1)	425(6)	1379(1)	44(1)	455(11)	1388(3)
51(1)	425(7)	1380(12)	52(1)	460(5)	1389(23)
59(1)	436(2)	1381(5)	62(1)	462(37)	1392(3)
65(1)	443(6)	1447(7)	71(2)	468(12)	1458(19)
66(0)	453(3)	1447(13)	73(0)	471(24)	1459(11)
72(0)	469(2)	1452(10)	76(0)	488(3)	1462(13)
75(0)	474(3)	1457(4)	79(0)	498(2)	1470(10)
76(1)	483(2)	1458(8)	85(0)	504(1)	1471(2)
83(0)	495(8)	1459(7)	92(0)	512(6)	1475(9)
85(1)	508(6)	1471(6)	95(0)	526(8)	1484(7)
89(0)	519(4)	1472(4)	102(1)	540(2)	1485(6)
96(1)	529(5)	1693(46)	107(1)	549(6)	1711(61)
97(0)	544(1)	1715(18)	113(0)	565(3)	1736(20)
102(1)	563(61)	1984(345)	120(1)	580(69)	2011(234)
110(0)	595(24)	2005(1395)	128(0)	612(22)	2028(1568)
126(0)	613(60)	2021(84)	136(3)	636(21)	2040(536)
134(2)	620(80)	2026(1153)	145(0)	644(91)	2050(1170)
145(0)	635(57)	2029(1407)	152(1)	660(72)	2052(1189)
152(0)	651(128)	2056(2064)	163(1)	675(271)	2079(2052)
171(4)	658(5)	2098(232)	179(3)	680(26)	2121(170)
197(0)	672(68)	3034(35)	204(0)	693(11)	3054(12)
208(1)	861(5)	3036(27)	217(3)	858(3)	3054(10)
236(2)	866(11)	3040(56)	247(2)	864(13)	3063(19)
269(4)	918(1)	3042(40)	275(17)	923(1)	3064(14)
277(7)	930(2)	3115(12)	284(5)	934(3)	3121(5)
281(0)	949(3)	3116(13)	286(2)	956(3)	3124(5)
304(16)	959(4)	3125(16)	313(17)	962(5)	3138(10)
319(1)	1077(1)	3134(14)	332(1)	1083(4)	3148(6)
375(7)	1078(5)	3157(22)	396(4)	1084(3)	3166(15)
382(2)	1085(16)	3169(15)	408(1)	1091(14)	3184(8)
396(0)	1089(24)	3175(13)	411(1)	1094(22)	3190(7)
405(6)	1239(0)	3180(13)	419(2)	1237(1)	3203(6)

Table S178. Harmonic vibrational frequencies (in cm^{-1}) and infrared intensities (in parentheses, in km/mol) for the $(Me_2C{=}N)_2Cr_2(CO)_7$ structure **2-7T-2**.

M06-L			B3PW91-D3		
38(0)	389(13)	1236(1)	29(0)	412(0)	1237(1)
40(1)	403(0)	1378(1)	37(8)	425(5)	1388(0)
45(0)	404(20)	1378(23)	43(2)	427(5)	1388(50)
47(0)	408(0)	1382(2)	51(6)	441(3)	1390(3)
53(2)	420(7)	1382(37)	52(2)	448(7)	1390(21)
59(0)	429(19)	1449(6)	55(2)	452(2)	1457(5)
60(0)	433(12)	1449(3)	61(2)	458(6)	1457(19)
63(0)	437(4)	1450(3)	65(0)	459(0)	1459(19)
75(1)	446(30)	1450(13)	71(0)	464(9)	1459(2)
75(1)	460(9)	1458(10)	76(0)	471(6)	1469(16)
77(2)	464(2)	1459(4)	81(0)	477(20)	1469(0)
80(0)	480(8)	1468(1)	87(0)	494(3)	1480(5)
89(0)	494(0)	1468(8)	93(0)	507(8)	1480(9)
90(0)	507(5)	1716(61)	96(1)	509(7)	1718(215)
98(0)	508(2)	1724(25)	103(0)	530(14)	1728(31)
105(0)	531(32)	1965(463)	104(0)	578(1)	1968(522)
120(1)	545(59)	2010(679)	119(0)	581(35)	2026(206)
133(0)	549(2)	2013(1637)	126(0)	587(79)	2028(1503)
139(1)	596(63)	2021(1007)	137(5)	592(74)	2061(35)
142(0)	609(36)	2037(1634)	145(0)	603(66)	2065(1616)
155(0)	637(4)	2047(2511)	147(6)	637(1)	2077(2600)
159(13)	644(52)	2103(194)	170(41)	659(6)	2125(342)
174(0)	655(100)	3028(30)	178(0)	679(61)	3052(7)
199(5)	858(8)	3029(30)	213(5)	855(4)	3052(0)
240(0)	861(4)	3035(82)	244(2)	858(3)	3056(38)
261(0)	926(7)	3035(27)	283(2)	924(1)	3056(11)
265(7)	927(3)	3114(3)	285(1)	924(6)	3122(9)
288(2)	939(0)	3114(5)	293(0)	941(1)	3122(1)
291(7)	939(1)	3115(15)	296(26)	941(1)	3127(1)
296(3)	1078(0)	3115(39)	304(5)	1086(6)	3127(21)
362(50)	1078(0)	3150(26)	322(15)	1087(0)	3177(15)
363(4)	1084(31)	3150(17)	362(1)	1089(14)	3177(1)
374(25)	1087(12)	3174(10)	405(18)	1092(10)	3195(3)
389(4)	1236(0)	3174(19)	411(16)	1236(0)	3195(13)

Table S179. Harmonic vibrational frequencies (in cm^{-1}) and infrared intensities (in parentheses, in km/mol) for the $(Me_2C{=}N)_2Cr_2(CO)_7$ structure **2-7S-3**.

M06-L			B3PW91-D3		
22(0)	417(31)	1239(1)	26(1)	440(12)	1238(1)
41(0)	419(59)	1376(16)	44(0)	449(14)	1384(21)
44(1)	425(6)	1379(1)	51(1)	455(11)	1388(3)
51(1)	425(7)	1380(12)	58(1)	460(5)	1389(23)
59(1)	436(2)	1381(5)	71(1)	462(37)	1392(3)
65(1)	443(6)	1447(7)	74(2)	468(12)	1458(19)
66(0)	453(3)	1447(13)	75(1)	471(24)	1459(11)
72(0)	469(2)	1452(10)	80(1)	488(3)	1462(13)
75(0)	474(3)	1457(4)	85(0)	498(2)	1470(10)
76(1)	483(2)	1458(8)	88(1)	504(1)	1471(2)
83(0)	495(8)	1459(7)	88(0)	512(6)	1475(9)
85(1)	508(6)	1471(6)	95(0)	526(8)	1484(7)
89(0)	519(4)	1472(4)	105(1)	540(2)	1485(6)
96(1)	529(5)	1693(46)	109(0)	549(6)	1711(61)
97(0)	544(1)	1715(18)	114(3)	565(3)	1736(20)
102(1)	563(61)	1984(345)	125(2)	580(69)	2011(234)
110(0)	595(24)	2005(1395)	130(0)	612(22)	2028(1568)
126(0)	613(60)	2021(84)	137(4)	636(21)	2040(536)
134(2)	620(80)	2026(1153)	156(2)	644(91)	2050(1170)
145(0)	635(57)	2029(1407)	171(3)	660(72)	2052(1189)
152(0)	651(128)	2056(2064)	184(0)	675(271)	2079(2052)
171(4)	658(5)	2098(232)	187(0)	680(26)	2121(170)
197(0)	672(68)	3034(35)	204(0)	693(11)	3054(12)
208(1)	861(5)	3036(27)	217(3)	858(3)	3054(10)
236(2)	866(11)	3040(56)	247(2)	864(13)	3063(19)
269(4)	918(1)	3042(40)	275(17)	923(1)	3064(14)
277(7)	930(2)	3115(12)	284(5)	934(3)	3121(5)
281(0)	949(3)	3116(13)	286(2)	956(3)	3124(5)
304(16)	959(4)	3125(16)	313(17)	962(5)	3138(10)
319(1)	1077(1)	3134(14)	332(1)	1083(4)	3148(6)
375(7)	1078(5)	3157(22)	396(4)	1084(3)	3166(15)
382(2)	1085(16)	3169(15)	408(1)	1091(14)	3184(8)
396(0)	1089(24)	3175(13)	411(1)	1094(22)	3190(7)
405(6)	1239(0)	3180(13)	419(2)	1237(1)	3203(6)

Table S180. Harmonic vibrational frequencies (in cm^{-1}) and infrared intensities (in parentheses, in km/mol) for the $(Me_2C{=}N)_2Cr_2(CO)_7$ structure **2-7S-4**.

M06-L			B3PW91-D3		
9(0)	408(0)	1243(2)	21(0)	416(0)	1242(2)
27(1)	418(2)	1381(9)	37(1)	427(10)	1391(19)
37(0)	425(4)	1383(6)	38(0)	438(2)	1392(31)
40(0)	439(11)	1385(25)	39(0)	450(12)	1394(6)
48(0)	443(15)	1389(6)	44(0)	466(8)	1400(9)
54(0)	456(0)	1448(7)	53(0)	466(1)	1458(12)
55(1)	461(0)	1449(1)	62(2)	472(0)	1460(1)
67(0)	464(0)	1450(28)	69(0)	480(4)	1461(29)
75(1)	469(13)	1455(3)	71(2)	484(9)	1463(4)
77(1)	489(8)	1456(0)	79(0)	513(36)	1466(5)
81(0)	492(42)	1458(11)	85(0)	514(9)	1470(4)
88(1)	516(3)	1470(10)	86(1)	535(1)	1482(12)
89(1)	528(35)	1476(1)	90(1)	549(14)	1487(2)
92(0)	533(14)	1544(14)	92(0)	558(20)	1546(11)
99(1)	560(85)	1700(11)	94(0)	575(9)	1725(16)
101(0)	561(2)	1990(199)	97(0)	577(122)	2016(38)
106(0)	574(74)	2000(596)	109(0)	595(95)	2022(726)
113(0)	615(40)	2005(2087)	118(0)	635(58)	2030(2340)
126(0)	625(11)	2014(531)	122(0)	641(12)	2043(473)
133(0)	626(10)	2050(2238)	136(0)	651(17)	2069(2313)
138(4)	641(35)	2080(30)	145(1)	659(17)	2103(85)
171(0)	671(60)	2180(145)	154(1)	692(77)	2201(122)
178(0)	712(43)	3030(14)	160(0)	743(55)	3049(5)
181(0)	865(14)	3032(70)	179(0)	867(5)	3052(31)
194(8)	870(16)	3034(16)	208(3)	877(22)	3053(4)
210(5)	930(1)	3039(64)	221(9)	938(0)	3059(26)
210(0)	937(0)	3114(7)	225(0)	941(1)	3124(4)
221(0)	938(2)	3117(15)	233(0)	947(1)	3129(8)
276(4)	973(0)	3119(1)	283(14)	982(0)	3132(1)
290(15)	1065(65)	3120(18)	299(14)	1076(73)	3134(7)
302(0)	1080(0)	3163(9)	305(0)	1088(0)	3175(3)
305(9)	1086(25)	3164(26)	317(9)	1092(23)	3177(13)
374(14)	1099(12)	3180(10)	394(13)	1110(11)	3192(7)
401(0)	1243(5)	3182(6)	409(0)	1237(3)	3194(2)

Table S181. Harmonic vibrational frequencies (in cm^{-1}) and infrared intensities (in parentheses, in km/mol) for the $(Me_2C{=}N)_2Cr_2(CO)_7$ structure **2-7S-5**.

M06-L			B3PW91-D3		
20(1)	426(7)	1299(9)	21(1)	434(1)	1298(9)
42(1)	436(28)	1388(19)	42(0)	456(7)	1399(26)
49(0)	439(8)	1392(10)	50(0)	466(3)	1403(10)
53(1)	452(5)	1397(21)	57(1)	470(1)	1408(40)
58(1)	457(12)	1404(45)	63(0)	473(8)	1411(42)
64(0)	461(6)	1411(25)	67(2)	480(54)	1423(16)
77(0)	465(2)	1441(1)	74(1)	484(3)	1452(3)
81(1)	481(4)	1445(1)	81(0)	496(9)	1454(6)
85(0)	499(6)	1452(6)	86(0)	511(11)	1457(3)
87(0)	513(16)	1455(4)	90(0)	530(26)	1467(24)
89(1)	527(15)	1462(18)	91(0)	540(15)	1467(3)
96(1)	530(18)	1468(11)	93(2)	546(6)	1479(22)
101(1)	543(4)	1470(11)	98(1)	561(7)	1485(2)
108(0)	576(24)	1499(2)	103(1)	589(19)	1512(3)
121(2)	583(9)	1713(20)	111(0)	599(15)	1751(23)
129(0)	605(32)	1964(255)	124(2)	624(44)	1975(471)
146(2)	618(10)	1965(430)	131(0)	639(25)	1992(264)
155(0)	625(48)	1986(1155)	144(0)	649(67)	2003(1292)
166(1)	636(12)	2003(1227)	156(0)	655(7)	2025(1246)
170(2)	680(80)	2019(631)	170(11)	701(62)	2039(703)
173(3)	688(32)	2048(2145)	184(1)	710(78)	2070(2173)
196(8)	725(2)	2091(588)	197(1)	747(4)	2110(551)
202(6)	839(13)	3021(10)	205(7)	843(9)	3043(3)
213(2)	855(10)	3023(61)	213(6)	861(10)	3047(25)
228(2)	938(2)	3039(14)	246(1)	947(0)	3058(4)
263(2)	950(1)	3043(28)	271(3)	954(1)	3065(9)
271(2)	950(1)	3115(14)	284(1)	958(0)	3128(12)
341(7)	965(1)	3123(6)	354(1)	975(2)	3132(3)
355(16)	997(1)	3123(9)	365(15)	1012(1)	3139(4)
360(18)	1053(42)	3129(9)	377(0)	1064(32)	3140(2)
373(5)	1067(29)	3142(10)	386(31)	1077(41)	3155(4)
394(1)	1087(1)	3169(10)	414(1)	1095(3)	3179(4)
404(9)	1120(31)	3169(11)	420(19)	1139(30)	3183(5)
410(3)	1289(3)	3172(8)	421(1)	1283(3)	3186(4)

Table S182. Harmonic vibrational frequencies (in cm^{-1}) and infrared intensities (in parentheses, in km/mol) for the $(Me_2C{=}N)_2Cr_2(CO)_7$ structure **2-7T-6**.

M06-L			B3PW91-D3		
19(0)	391(45)	1228(21)	31(0)	408(17)	1235(1)
36(2)	391(29)	1370(3)	37(11)	423(8)	1386(0)
40(0)	406(15)	1371(54)	46(1)	424(6)	1386(59)
50(1)	416(3)	1379(15)	51(6)	437(1)	1388(13)
51(0)	430(16)	1379(34)	52(3)	442(8)	1388(3)
59(0)	442(1)	1440(5)	60(1)	449(5)	1456(6)
66(1)	449(13)	1440(0)	62(0)	456(6)	1457(12)
68(2)	466(27)	1445(2)	68(0)	456(1)	1459(0)
68(0)	468(1)	1446(4)	74(0)	460(9)	1459(17)
72(0)	473(7)	1451(59)	79(0)	466(7)	1467(25)
74(2)	479(6)	1452(18)	84(0)	471(19)	1468(1)
81(0)	481(4)	1464(15)	89(0)	492(2)	1479(3)
83(2)	499(0)	1464(5)	91(0)	506(9)	1479(8)
96(1)	510(0)	1626(190)	98(1)	509(6)	1712(220)
98(0)	532(29)	1648(6)	99(0)	530(13)	1721(32)
106(1)	537(10)	1979(661)	105(0)	575(1)	1965(496)
114(3)	555(1)	1988(509)	119(1)	579(29)	2023(214)
117(0)	560(58)	1998(1272)	128(0)	583(75)	2025(1516)
120(1)	564(3)	2023(2250)	133(1)	588(72)	2059(35)
128(0)	619(37)	2040(694)	141(1)	599(61)	2062(1588)
135(0)	623(11)	2062(2235)	145(3)	632(0)	2076(2617)
143(0)	627(36)	2103(71)	167(47)	653(7)	2123(367)
148(3)	674(85)	3030(19)	171(0)	672(66)	3053(7)
169(0)	844(6)	3030(16)	208(5)	851(3)	3053(0)
177(2)	851(0)	3035(85)	245(2)	855(3)	3058(42)
182(0)	903(0)	3036(5)	272(2)	923(0)	3058(13)
255(7)	903(4)	3118(4)	276(2)	923(6)	3122(1)
264(4)	933(0)	3118(5)	284(0)	931(1)	3122(6)
289(1)	933(1)	3122(3)	292(18)	932(1)	3126(2)
340(1)	1068(41)	3122(14)	305(4)	1085(20)	3126(22)
353(0)	1071(1)	3162(13)	319(28)	1087(3)	3181(14)
372(8)	1082(17)	3162(11)	357(1)	1087(0)	3181(2)
377(0)	1088(3)	3171(17)	405(18)	1089(8)	3196(3)
381(2)	1227(2)	3171(12)	407(0)	1234(0)	3196(13)

Table S183. Harmonic vibrational frequencies (in cm^{-1}) and infrared intensities (in parentheses, in km/mol) for the $(Me_2C=N)_2Cr_2(CO)_7$ structure **2-7S-7**.

M06-L			B3PW91-D3		
34(1)	406(6)	1277(8)	37(1)	424(17)	1272(7)
40(0)	412(9)	1377(7)	41(1)	436(19)	1383(14)
43(0)	421(10)	1380(8)	43(0)	443(2)	1389(8)
53(0)	436(5)	1389(8)	55(1)	449(8)	1394(20)
57(1)	441(2)	1395(41)	57(1)	452(13)	1396(42)
61(0)	442(2)	1444(10)	68(0)	455(3)	1451(11)
67(1)	449(2)	1448(4)	71(1)	462(1)	1456(4)
73(1)	457(1)	1451(7)	77(0)	473(2)	1458(17)
75(0)	463(3)	1452(7)	79(0)	482(2)	1461(9)
76(0)	476(8)	1455(16)	82(1)	490(7)	1462(17)
81(0)	488(18)	1460(22)	84(0)	508(16)	1470(9)
86(1)	501(6)	1466(7)	88(1)	519(13)	1475(12)
93(1)	515(14)	1472(4)	90(1)	534(17)	1482(7)
99(0)	539(19)	1683(129)	92(0)	554(19)	1702(145)
107(0)	554(71)	1735(197)	94(0)	574(29)	1746(374)
119(1)	563(5)	1737(156)	113(0)	584(68)	1757(8)
133(0)	585(41)	1968(229)	124(0)	602(45)	1985(381)
138(0)	594(58)	2001(1128)	136(0)	617(60)	2036(1011)
157(1)	617(13)	2027(436)	142(1)	636(36)	2052(1250)
167(0)	627(22)	2035(1552)	150(1)	641(39)	2062(1036)
173(0)	639(27)	2062(1966)	160(0)	657(6)	2081(1926)
182(0)	729(160)	2096(688)	173(0)	740(168)	2120(716)
196(0)	800(9)	3030(31)	193(0)	807(7)	3054(4)
225(1)	869(9)	3032(13)	235(2)	864(9)	3055(5)
239(4)	901(9)	3035(67)	253(8)	906(25)	3060(32)
260(15)	932(1)	3046(32)	259(20)	934(4)	3062(17)
271(2)	936(30)	3115(13)	279(2)	935(37)	3123(4)
280(1)	943(2)	3116(5)	285(3)	942(2)	3124(2)
285(4)	961(1)	3117(20)	292(3)	952(2)	3128(10)
311(0)	1078(1)	3126(15)	318(0)	1086(2)	3130(12)
379(20)	1084(3)	3153(21)	392(6)	1091(6)	3176(9)
390(15)	1091(39)	3165(10)	404(6)	1094(40)	3188(7)
396(14)	1113(0)	3167(20)	413(25)	1117(0)	3189(8)
398(14)	1239(1)	3188(7)	420(18)	1235(1)	3206(1)

Table S184. Harmonic vibrational frequencies (in cm^{-1}) and infrared intensities (in parentheses, in km/mol) for the $(Me_2C{=}N)_2Cr_2(CO)_7$ structure **2-7S-8**.

M06-L			B3PW91-D3		
30(1)	434(3)	1302(3)	19(1)	441(1)	1299(3)
49(0)	443(7)	1386(11)	40(0)	451(7)	1397(20)
51(1)	451(0)	1393(11)	46(1)	453(13)	1399(17)
55(1)	452(6)	1399(17)	51(1)	463(8)	1402(8)
55(0)	454(17)	1406(35)	56(0)	467(6)	1406(49)
71(0)	460(6)	1445(24)	72(0)	473(33)	1452(36)
73(1)	466(6)	1450(11)	79(1)	482(5)	1456(9)
76(0)	476(8)	1451(9)	79(0)	486(6)	1459(15)
81(0)	487(6)	1455(4)	86(1)	494(11)	1464(4)
87(1)	505(2)	1460(5)	90(3)	512(4)	1470(11)
89(3)	523(10)	1461(22)	92(0)	530(2)	1473(4)
97(1)	525(9)	1473(5)	95(0)	542(13)	1481(10)
103(0)	538(50)	1478(12)	100(1)	543(4)	1483(25)
114(0)	562(35)	1665(43)	107(1)	575(5)	1671(41)
115(0)	571(10)	1677(2)	110(0)	586(20)	1694(8)
124(0)	605(11)	1810(273)	117(0)	622(15)	1926(11)
128(3)	620(9)	1889(611)	118(0)	628(17)	1947(1286)
132(0)	632(61)	1985(918)	128(0)	644(30)	1996(999)
138(0)	647(21)	1997(960)	136(1)	659(16)	2013(953)
157(0)	690(66)	2010(871)	150(1)	702(50)	2030(682)
187(1)	710(22)	2029(1995)	166(1)	712(41)	2045(1946)
193(2)	757(5)	2081(775)	169(0)	755(2)	2102(816)
199(2)	832(1)	3026(18)	175(1)	832(0)	3051(5)
211(1)	859(5)	3037(43)	185(7)	857(3)	3060(10)
212(4)	940(6)	3040(9)	209(0)	946(6)	3060(2)
234(2)	947(0)	3046(25)	229(0)	951(1)	3065(10)
247(1)	954(1)	3119(5)	251(1)	956(2)	3129(2)
301(175)	966(1)	3121(11)	346(57)	965(0)	3131(6)
354(1)	988(2)	3125(9)	356(2)	995(1)	3135(4)
377(2)	1076(15)	3127(11)	373(4)	1077(16)	3136(6)
385(9)	1083(1)	3134(21)	391(3)	1088(1)	3151(7)
391(26)	1085(1)	3165(11)	399(31)	1090(1)	3185(4)
416(2)	1124(28)	3174(8)	417(1)	1137(34)	3185(1)
425(8)	1298(1)	3193(4)	432(2)	1296(1)	3198(3)

Table S185. Harmonic vibrational frequencies (in cm^{-1}) and infrared intensities (in parentheses, in km/mol) for the $(Me_2C{=}N)_2Cr_2(CO)_7$ structure **2-7T-9**.

M06-L			B3PW91-D3		
23(1)	395(25)	1277(8)	33(1)	414(6)	1274(8)
35(1)	404(14)	1377(7)	39(1)	424(6)	1384(22)
36(0)	405(9)	1382(17)	41(1)	428(4)	1389(12)
45(0)	412(6)	1386(6)	50(1)	431(26)	1394(14)
51(1)	415(7)	1394(47)	59(2)	433(6)	1396(52)
54(1)	429(9)	1442(9)	60(0)	438(7)	1450(9)
58(0)	437(5)	1447(7)	62(1)	453(17)	1456(4)
61(1)	444(3)	1449(9)	69(2)	460(2)	1458(24)
65(1)	450(5)	1450(19)	72(1)	466(9)	1460(3)
69(0)	456(1)	1452(13)	77(1)	467(2)	1461(21)
71(1)	468(6)	1459(11)	79(0)	473(24)	1469(15)
75(0)	477(19)	1465(12)	81(0)	497(12)	1476(13)
82(1)	489(8)	1469(6)	82(1)	513(15)	1481(7)
84(0)	515(20)	1669(114)	89(1)	531(19)	1695(126)
93(0)	521(35)	1712(314)	93(1)	539(21)	1733(143)
102(0)	558(29)	1720(76)	106(0)	567(18)	1734(304)
112(1)	559(10)	1954(461)	110(0)	577(24)	1991(330)
131(2)	584(29)	2015(804)	129(1)	601(39)	2017(1038)
137(1)	591(9)	2021(1194)	133(0)	619(11)	2048(1470)
145(1)	621(38)	2030(3171)	139(1)	634(26)	2053(1298)
156(2)	651(43)	2045(498)	152(2)	660(44)	2070(1845)
157(1)	741(153)	2104(1010)	158(2)	745(156)	2122(833)
171(1)	812(11)	3028(37)	165(3)	811(6)	3051(5)
213(2)	861(5)	3029(15)	206(11)	855(6)	3054(4)
222(0)	909(6)	3032(61)	222(1)	912(20)	3056(32)
254(2)	925(2)	3043(42)	262(6)	925(1)	3060(20)
277(5)	937(1)	3111(7)	277(3)	928(1)	3118(2)
286(6)	939(43)	3112(13)	279(4)	937(45)	3122(15)
289(6)	958(5)	3113(22)	290(2)	952(12)	3123(3)
299(1)	1078(1)	3121(17)	314(0)	1087(1)	3128(10)
342(98)	1081(3)	3155(16)	376(25)	1090(14)	3177(9)
368(3)	1089(20)	3156(21)	385(7)	1091(10)	3180(12)
384(5)	1117(1)	3162(18)	388(24)	1120(0)	3190(7)
392(1)	1238(0)	3192(4)	397(10)	1235(1)	3206(0)

Table S186. Harmonic vibrational frequencies (in cm^{-1}) and infrared intensities (in parentheses, in km/mol) for the $(Me_2C{=}N)_2Cr_2(CO)_7$ structure **2-7S-10**.

M06-L			B3PW91-D3		
26(1)	440(5)	1295(2)	33(1)	445(0)	1297(5)
41(1)	446(27)	1367(15)	35(1)	456(8)	1362(13)
47(1)	449(4)	1390(19)	51(0)	463(2)	1388(26)
54(0)	460(3)	1398(53)	57(0)	477(19)	1400(51)
67(0)	465(9)	1399(4)	63(0)	480(27)	1403(15)
76(2)	469(3)	1433(72)	76(1)	483(2)	1428(50)
78(3)	478(8)	1440(6)	78(0)	495(5)	1447(26)
81(2)	486(2)	1444(2)	84(0)	498(2)	1455(10)
84(1)	493(5)	1449(5)	87(1)	505(9)	1460(18)
88(2)	505(7)	1454(14)	91(1)	517(13)	1464(7)
91(4)	522(15)	1460(9)	96(1)	533(21)	1470(11)
98(1)	528(17)	1466(6)	102(1)	546(22)	1474(2)
109(1)	551(2)	1543(15)	106(1)	552(0)	1596(24)
111(3)	570(16)	1557(33)	114(0)	574(3)	1623(46)
125(6)	582(7)	1691(35)	123(0)	591(5)	1730(40)
144(4)	610(25)	1938(201)	131(2)	620(11)	1954(143)
145(1)	615(6)	1966(469)	140(5)	626(25)	1975(957)
159(0)	621(19)	1985(1275)	147(1)	629(21)	2002(1104)
164(3)	630(20)	2004(993)	155(2)	649(28)	2020(1018)
165(1)	637(33)	2022(796)	162(1)	654(21)	2038(762)
178(1)	678(79)	2033(1712)	173(1)	693(55)	2052(1961)
182(3)	689(17)	2092(684)	177(1)	709(40)	2111(524)
193(2)	837(5)	2871(7)	188(4)	832(1)	2778(6)
203(2)	876(8)	3032(24)	209(0)	867(3)	3057(2)
216(2)	923(0)	3038(26)	213(1)	917(0)	3058(7)
248(2)	930(1)	3046(26)	227(3)	927(0)	3069(5)
267(1)	951(4)	3069(3)	303(5)	962(1)	3102(3)
346(37)	968(2)	3117(13)	363(1)	970(6)	3123(1)
356(13)	997(1)	3123(5)	371(11)	998(4)	3127(6)
363(7)	1058(8)	3133(10)	384(1)	1059(4)	3144(3)
376(2)	1067(63)	3143(8)	392(2)	1086(10)	3146(2)
390(8)	1090(1)	3179(11)	419(0)	1102(2)	3183(9)
419(1)	1104(3)	3190(4)	424(5)	1115(1)	3189(3)
436(1)	1275(16)	3205(2)	434(1)	1267(16)	3214(2)

Table S187. Harmonic vibrational frequencies (in cm^{-1}) and infrared intensities (in parentheses, in km/mol) for the $(Me_2C{=}N)_2Cr_2(CO)_7$ structure **2-7T-11**.

M06-L			B3PW91-D3		
30(1)	393(9)	1296(8)	19(0)	410(2)	1293(7)
43(0)	401(5)	1343(18)	45(0)	415(5)	1362(20)
49(2)	407(4)	1390(14)	53(2)	424(8)	1397(12)
51(1)	421(23)	1392(8)	55(0)	435(18)	1398(18)
56(0)	423(9)	1400(10)	59(1)	438(27)	1407(49)
63(2)	437(3)	1408(37)	62(2)	455(9)	1408(4)
64(1)	451(22)	1445(9)	68(0)	470(2)	1454(8)
75(0)	457(13)	1448(8)	78(1)	474(18)	1456(1)
79(1)	462(13)	1453(5)	79(1)	484(2)	1463(10)
82(0)	470(2)	1458(22)	81(1)	501(20)	1466(22)
88(1)	487(10)	1460(8)	88(0)	508(16)	1470(6)
92(1)	504(25)	1469(22)	90(0)	519(6)	1477(23)
96(0)	513(9)	1475(4)	100(1)	527(42)	1489(8)
108(0)	531(4)	1481(6)	102(1)	550(5)	1492(7)
115(1)	542(15)	1709(35)	107(1)	557(17)	1738(47)
120(0)	554(32)	1921(273)	111(0)	571(31)	1931(307)
137(1)	562(5)	1979(486)	120(1)	582(6)	2009(615)
140(1)	621(12)	2001(925)	135(1)	640(14)	2024(914)
147(0)	633(44)	2005(1731)	143(1)	661(56)	2026(1753)
175(5)	634(54)	2017(436)	156(0)	665(71)	2036(431)
186(5)	667(112)	2037(2614)	178(7)	696(160)	2059(2442)
191(1)	703(2)	2092(636)	191(8)	713(3)	2113(645)
203(1)	828(18)	3010(60)	198(18)	826(20)	3030(30)
211(5)	851(6)	3027(44)	204(7)	847(6)	3051(24)
223(1)	935(1)	3041(6)	219(4)	940(0)	3061(2)
249(1)	944(1)	3043(36)	250(0)	947(1)	3067(11)
260(1)	954(0)	3115(10)	258(6)	954(0)	3131(0)
315(21)	961(1)	3124(10)	330(40)	969(2)	3132(3)
336(19)	998(1)	3125(5)	347(10)	1002(1)	3136(8)
347(22)	1037(25)	3127(15)	358(16)	1043(32)	3140(7)
360(18)	1067(64)	3166(12)	373(26)	1073(71)	3183(7)
384(1)	1087(2)	3167(13)	389(12)	1094(2)	3183(4)
387(12)	1120(24)	3174(9)	393(6)	1131(24)	3193(3)
388(3)	1276(1)	3176(10)	398(29)	1266(3)	3199(5)

Table S188. Harmonic vibrational frequencies (in cm^{-1}) and infrared intensities (in parentheses, in km/mol) for the $(Me_2C{=}N)_2Cr_2(CO)_7$ structure **2-7T-12**.

M06-L			B3PW91-D3		
26(1)	411(2)	1302(2)	24(0)	414(1)	1299(2)
38(1)	412(7)	1391(11)	32(0)	417(4)	1398(19)
45(0)	414(1)	1392(11)	39(1)	425(5)	1398(15)
49(0)	425(5)	1403(7)	52(1)	435(0)	1404(5)
54(1)	435(3)	1406(57)	56(0)	444(1)	1406(60)
56(1)	445(3)	1441(13)	65(1)	459(20)	1451(28)
65(0)	448(15)	1444(7)	71(1)	465(2)	1456(24)
66(0)	458(5)	1448(24)	75(1)	472(3)	1459(15)
74(1)	464(6)	1450(10)	81(1)	474(16)	1461(1)
80(1)	465(2)	1459(5)	86(1)	491(21)	1467(14)
84(0)	474(11)	1463(20)	94(1)	502(6)	1471(7)
88(1)	489(10)	1469(6)	95(1)	506(22)	1479(10)
100(0)	507(20)	1471(10)	102(0)	521(3)	1484(22)
106(0)	517(4)	1662(21)	108(0)	547(0)	1668(29)
111(1)	529(6)	1679(14)	113(1)	561(21)	1684(9)
120(0)	570(9)	1763(371)	118(1)	569(8)	1911(246)
123(1)	575(40)	1951(294)	121(1)	585(6)	1965(819)
131(0)	593(15)	1994(623)	135(0)	614(26)	1999(1096)
143(0)	600(52)	2001(821)	140(0)	645(57)	2021(1029)
156(0)	634(31)	2013(727)	160(0)	660(69)	2037(933)
163(3)	642(63)	2014(3481)	174(1)	698(102)	2047(2103)
182(0)	757(3)	2082(819)	176(0)	753(0)	2103(850)
186(3)	836(0)	3028(18)	179(1)	829(0)	3052(7)
204(1)	862(4)	3037(45)	193(7)	858(2)	3059(2)
212(0)	944(4)	3040(12)	210(1)	948(2)	3061(10)
242(1)	950(2)	3041(33)	225(1)	950(3)	3063(15)
244(2)	954(1)	3116(11)	240(0)	956(1)	3127(5)
341(10)	966(1)	3122(8)	328(2)	963(0)	3131(5)
353(3)	988(1)	3124(8)	357(1)	995(1)	3133(4)
358(4)	1078(11)	3127(11)	370(2)	1077(18)	3134(7)
370(20)	1082(6)	3146(13)	382(3)	1087(1)	3158(3)
377(13)	1084(0)	3166(8)	393(5)	1090(1)	3185(4)
382(10)	1128(31)	3168(9)	395(3)	1138(36)	3185(1)
393(3)	1301(0)	3173(7)	406(5)	1295(1)	3190(3)

Table S189. Harmonic vibrational frequencies (in cm^{-1}) and infrared intensities (in parentheses, in km/mol) for the $(Me_2C{=}N)_2Cr_2(CO)_7$ structure **2-7T-13**.

M06-L			B3PW91-D3		
38(1)	406(21)	1296(2)	27(0)	406(4)	1288(5)
48(0)	414(6)	1373(14)	33(0)	413(2)	1394(22)
51(0)	417(1)	1389(25)	35(4)	426(7)	1397(30)
58(1)	420(0)	1399(20)	51(3)	432(4)	1402(40)
66(1)	436(22)	1403(35)	56(1)	446(7)	1407(21)
71(1)	443(6)	1432(53)	59(5)	464(12)	1449(44)
73(1)	449(35)	1441(3)	67(1)	465(23)	1454(20)
78(1)	452(9)	1450(1)	72(1)	474(16)	1455(11)
81(1)	464(6)	1452(18)	76(2)	477(3)	1457(1)
84(1)	481(10)	1458(6)	80(1)	486(14)	1459(14)
88(6)	488(17)	1464(30)	83(2)	498(6)	1466(14)
92(0)	494(7)	1466(11)	87(1)	511(21)	1477(6)
95(2)	509(10)	1479(32)	93(0)	537(4)	1480(32)
109(2)	530(29)	1538(8)	102(1)	539(10)	1567(13)
116(1)	544(2)	1687(27)	106(0)	552(1)	1717(69)
127(2)	557(4)	1900(254)	116(0)	566(17)	1928(391)
153(1)	569(4)	1955(371)	121(1)	582(9)	1994(218)
159(3)	604(74)	1970(1268)	151(0)	617(38)	2007(1509)
171(1)	612(10)	2014(1005)	155(1)	631(47)	2035(1052)
186(1)	625(23)	2022(1842)	165(1)	652(60)	2043(746)
187(2)	630(51)	2027(1173)	171(1)	676(12)	2048(2333)
196(5)	659(118)	2088(763)	173(1)	689(112)	2110(689)
206(1)	833(4)	2936(1)	187(1)	828(4)	3047(7)
213(1)	873(7)	3032(26)	190(3)	848(5)	3056(2)
215(2)	929(1)	3035(27)	207(1)	930(0)	3057(13)
232(0)	937(0)	3043(31)	222(0)	938(0)	3060(11)
253(1)	950(3)	3067(3)	236(2)	953(2)	3126(4)
326(1)	967(5)	3114(15)	328(3)	964(2)	3133(12)
345(9)	993(2)	3124(3)	345(1)	989(1)	3138(1)
358(11)	1060(6)	3129(10)	356(3)	1066(22)	3141(4)
369(17)	1070(66)	3149(19)	366(4)	1076(55)	3183(8)
381(6)	1081(2)	3175(11)	371(15)	1094(2)	3186(4)
388(1)	1104(6)	3196(3)	394(4)	1115(10)	3198(1)
398(4)	1279(16)	3197(2)	398(13)	1280(10)	3206(2)

Table S190. Harmonic vibrational frequencies (in cm^{-1}) and infrared intensities (in parentheses, in km/mol) for the $(Me_2C{=}N)_2Fe_2(CO)_4$ structure **2-4S-1**.

M06-L			B3PW91-D3		
21(1)	454(0)	1383(15)	11(1)	455(0)	1391(3)
32(0)	456(2)	1385(5)	24(0)	469(6)	1393(5)
45(1)	482(2)	1419(17)	46(1)	495(7)	1434(23)
52(0)	497(0)	1448(0)	52(0)	510(0)	1457(9)
64(0)	499(1)	1449(6)	60(0)	513(2)	1460(0)
70(1)	543(16)	1453(1)	75(1)	555(14)	1463(0)
74(0)	551(33)	1458(7)	78(0)	564(27)	1466(8)
80(0)	570(7)	1460(4)	82(0)	585(86)	1467(6)
85(0)	573(81)	1468(6)	89(0)	591(13)	1481(7)
91(1)	592(26)	1470(4)	94(1)	611(35)	1482(6)
111(0)	646(26)	1497(1)	114(0)	663(15)	1506(1)
123(0)	662(4)	1709(16)	129(1)	678(1)	1739(22)
123(1)	692(42)	2017(213)	134(0)	713(53)	2044(244)
144(0)	728(27)	2023(1558)	146(0)	743(29)	2051(1606)
197(0)	865(15)	2049(2148)	189(0)	874(9)	2072(2312)
210(1)	877(2)	2072(19)	213(1)	879(0)	2097(28)
223(0)	937(0)	3020(24)	227(0)	940(0)	3041(11)
233(4)	943(0)	3021(125)	240(5)	944(0)	3043(66)
237(1)	946(0)	3030(19)	248(2)	950(0)	3051(6)
276(1)	979(0)	3033(73)	286(2)	982(0)	3055(36)
292(0)	1066(65)	3110(1)	298(0)	1075(60)	3122(0)
301(1)	1079(0)	3110(36)	315(5)	1087(25)	3125(1)
308(10)	1079(22)	3113(3)	324(8)	1088(0)	3126(14)
398(26)	1096(26)	3114(29)	401(1)	1102(25)	3127(18)
399(1)	1228(3)	3151(14)	414(31)	1229(2)	3165(6)
424(3)	1251(0)	3152(36)	426(3)	1253(0)	3167(20)
438(0)	1381(14)	3158(18)	438(0)	1386(21)	3181(8)
439(12)	1382(3)	3162(15)	447(8)	1389(24)	3182(6)

Table S191. Harmonic vibrational frequencies (in cm^{-1}) and infrared intensities (in parentheses, in km/mol) for the $(Me_2C{=}N)_2Fe_2(CO)_4$ structure **2-4S-2**.

M06-L			B3PW91-D3		
11(1)	455(0)	1391(3)	44(0)	479(51)	1391(81)
24(0)	469(6)	1393(5)	44(1)	482(0)	1391(2)
46(1)	495(7)	1434(23)	52(0)	490(7)	1455(0)
52(0)	510(0)	1457(9)	56(0)	510(0)	1456(0)
60(0)	513(2)	1460(0)	62(0)	512(4)	1460(0)
75(1)	555(14)	1463(0)	63(0)	533(1)	1460(20)
78(0)	564(27)	1466(8)	77(1)	564(9)	1468(13)
82(0)	585(86)	1467(6)	85(0)	567(0)	1469(7)
89(0)	591(13)	1481(7)	87(0)	603(41)	1476(2)
94(1)	611(35)	1482(6)	87(0)	612(0)	1477(18)
114(0)	663(15)	1506(1)	91(0)	650(14)	1763(155)
129(1)	678(1)	1739(22)	110(0)	659(28)	1772(87)
134(0)	713(53)	2044(244)	111(0)	691(0)	2040(0)
146(0)	743(29)	2051(1606)	145(0)	729(3)	2057(1639)
189(0)	874(9)	2072(2312)	152(0)	860(0)	2058(1790)
213(1)	879(0)	2097(28)	153(1)	868(0)	2117(1020)
227(0)	940(0)	3041(11)	160(1)	932(0)	3043(0)
240(5)	944(0)	3043(66)	230(4)	932(0)	3043(20)
248(2)	950(0)	3051(6)	246(1)	938(0)	3047(35)
286(2)	982(0)	3055(36)	266(2)	938(1)	3047(25)
298(0)	1075(60)	3122(0)	300(0)	1089(0)	3119(0)
315(5)	1087(25)	3125(1)	302(0)	1089(0)	3119(0)
324(8)	1088(0)	3126(14)	334(0)	1098(27)	3122(11)
401(1)	1102(25)	3127(18)	366(7)	1104(24)	3123(24)
414(31)	1229(2)	3165(6)	445(8)	1250(0)	3152(0)
426(3)	1253(0)	3167(20)	458(2)	1252(4)	3152(17)
438(0)	1386(21)	3181(8)	459(6)	1390(0)	3153(38)
447(8)	1389(24)	3182(6)	470(0)	1391(9)	3154(16)

Table S192. Harmonic vibrational frequencies (in cm^{-1}) and infrared intensities (in parentheses, in km/mol) for the $(Me_2C{=}N)_2Fe_2(CO)_4$ structure **2-4S-3**.

M06-L			B3PW91-D3		
32(2)	459(2)	1385(1)	9(0)	469(2)	1391(34)
33(0)	464(2)	1386(40)	34(2)	477(4)	1394(3)
47(0)	479(9)	1443(5)	53(0)	491(1)	1452(9)
52(0)	479(10)	1449(1)	57(0)	495(15)	1457(7)
59(0)	483(5)	1451(6)	58(1)	502(4)	1458(3)
63(1)	516(12)	1451(5)	65(0)	542(14)	1462(5)
65(0)	524(6)	1456(22)	67(1)	546(3)	1463(20)
74(1)	546(15)	1460(2)	77(1)	563(22)	1468(4)
81(1)	564(14)	1465(10)	81(0)	570(21)	1477(12)
84(0)	573(48)	1468(8)	86(1)	589(58)	1483(7)
85(0)	599(1)	1675(134)	89(0)	612(1)	1697(145)
97(0)	604(12)	1718(1)	90(1)	620(18)	1751(3)
110(1)	684(12)	1994(402)	118(1)	703(5)	2023(391)
119(0)	730(44)	2005(1267)	130(0)	748(63)	2032(1359)
146(2)	873(3)	2028(1941)	148(2)	869(1)	2056(2027)
151(1)	878(2)	2065(552)	153(1)	877(1)	2095(566)
187(0)	913(3)	3029(15)	199(0)	915(4)	3046(5)
226(2)	939(4)	3032(109)	229(2)	944(0)	3051(42)
242(1)	941(0)	3032(13)	246(0)	945(2)	3053(5)
286(0)	951(0)	3035(75)	288(2)	948(3)	3057(42)
304(1)	1077(1)	3111(0)	314(5)	1088(0)	3117(0)
309(3)	1082(2)	3112(0)	322(0)	1089(5)	3121(0)
317(5)	1094(51)	3112(21)	329(3)	1098(43)	3121(17)
330(4)	1099(11)	3112(46)	339(8)	1103(16)	3125(16)
377(3)	1250(1)	3160(18)	396(4)	1251(0)	3171(8)
428(7)	1254(0)	3160(11)	438(9)	1255(1)	3171(7)
436(0)	1383(2)	3175(11)	443(0)	1389(23)	3192(4)
441(2)	1384(2)	3175(9)	449(4)	1389(7)	3192(6)

Table S193. Harmonic vibrational frequencies (in cm^{-1}) and infrared intensities (in parentheses, in km/mol) for the $(Me_2C=N)_2Fe_2(CO)_4$ structure **2-4T-4**.

M06-L			B3PW91-D3		
-7(0)	459(0)	1376(10)	20(0)	451(3)	1384(3)
8(1)	469(4)	1378(7)	24(2)	468(3)	1387(16)
40(1)	471(1)	1440(10)	40(0)	470(6)	1451(5)
41(3)	481(1)	1442(0)	46(2)	482(3)	1454(54)
55(0)	498(4)	1445(42)	62(1)	496(9)	1455(7)
64(0)	503(2)	1447(32)	67(1)	506(16)	1459(9)
72(1)	510(2)	1458(7)	80(1)	529(2)	1461(5)
77(1)	528(4)	1467(14)	89(0)	547(30)	1474(1)
94(1)	540(6)	1472(1)	90(0)	561(23)	1479(8)
100(1)	562(29)	1475(18)	100(0)	599(13)	1483(15)
100(1)	593(7)	1632(155)	106(1)	608(24)	1604(58)
113(2)	627(44)	1690(108)	122(1)	641(37)	1719(135)
125(0)	640(6)	2000(212)	139(1)	657(5)	2024(866)
139(0)	693(13)	2008(1395)	144(0)	687(17)	2045(914)
154(0)	843(3)	2047(2003)	164(0)	831(9)	2063(1623)
170(0)	855(6)	2070(281)	185(1)	853(2)	2101(650)
184(1)	900(7)	3030(11)	190(3)	922(2)	3047(7)
192(0)	914(3)	3034(69)	193(1)	927(1)	3048(11)
199(0)	917(2)	3035(22)	218(1)	946(1)	3052(26)
252(2)	960(3)	3045(33)	245(6)	949(1)	3055(18)
268(1)	1075(16)	3112(8)	259(0)	1075(51)	3122(10)
277(5)	1079(0)	3116(18)	298(1)	1077(1)	3124(5)
301(1)	1081(0)	3117(15)	316(3)	1085(2)	3132(12)
329(0)	1084(2)	3133(24)	357(7)	1089(7)	3132(6)
404(5)	1237(11)	3171(17)	396(6)	1240(6)	3175(10)
416(3)	1240(1)	3173(15)	408(3)	1241(6)	3178(9)
417(5)	1371(9)	3177(17)	420(1)	1383(17)	3179(10)
451(3)	1374(30)	3184(15)	426(2)	1383(39)	3182(8)

Table S194. Harmonic vibrational frequencies (in cm^{-1}) and infrared intensities (in parentheses, in km/mol) for the $(Me_2C{=}N)_2Fe_2(CO)_4$ structure **2-4T-5**.

M06-L			B3PW91-D3		
23(2)	448(2)	1379(14)	37(1)	455(1)	1391(14)
35(0)	460(1)	1379(23)	40(0)	461(28)	1391(3)
44(0)	465(8)	1444(4)	46(1)	462(0)	1456(0)
51(1)	474(5)	1446(24)	52(0)	488(4)	1456(9)
63(1)	480(12)	1448(22)	63(0)	497(2)	1460(0)
68(1)	494(7)	1453(8)	64(0)	506(3)	1461(13)
81(2)	517(9)	1454(15)	81(0)	535(0)	1467(13)
86(0)	530(7)	1464(2)	84(0)	560(0)	1468(12)
87(0)	539(14)	1470(11)	84(0)	576(34)	1478(3)
105(0)	562(13)	1477(13)	85(0)	621(156)	1480(14)
112(2)	596(19)	1612(70)	101(2)	625(36)	1760(150)
118(0)	626(38)	1690(96)	119(0)	661(99)	1771(79)
126(1)	634(13)	1996(715)	120(0)	666(0)	2014(2317)
138(0)	679(14)	2016(915)	147(0)	712(7)	2039(0)
169(0)	841(6)	2037(1725)	152(1)	862(0)	2094(1009)
182(0)	858(5)	2072(601)	161(5)	869(1)	2115(745)
191(1)	918(3)	3030(11)	162(1)	934(0)	3045(0)
208(0)	920(2)	3033(77)	226(1)	934(2)	3045(17)
239(3)	932(3)	3033(14)	233(3)	934(0)	3049(37)
242(1)	950(1)	3036(53)	274(4)	934(0)	3050(26)
246(1)	1074(36)	3114(2)	289(0)	1090(0)	3119(0)
295(0)	1077(1)	3115(20)	297(0)	1090(0)	3119(0)
312(5)	1079(0)	3117(19)	334(0)	1098(23)	3123(10)
356(4)	1085(7)	3119(22)	339(54)	1104(26)	3123(23)
386(7)	1240(10)	3170(18)	349(7)	1247(0)	3162(0)
410(1)	1242(3)	3171(15)	426(0)	1250(2)	3162(16)
426(3)	1376(4)	3174(16)	437(13)	1390(0)	3164(27)
432(2)	1377(10)	3175(17)	445(2)	1391(75)	3164(13)

Table S195. Harmonic vibrational frequencies (in cm^{-1}) and infrared intensities (in parentheses, in km/mol) for the $(Me_2C{=}N)_2Fe_2(CO)_4$ structure **2-4T-6**.

M06-L			B3PW91-D3		
-40(18)	437(1)	1386(0)	29(3)	450(1)	1389(12)
38(0)	448(0)	1386(66)	38(0)	457(0)	1390(3)
47(1)	452(0)	1445(0)	48(1)	462(29)	1456(0)
50(1)	479(9)	1446(0)	55(1)	488(4)	1457(7)
54(10)	481(2)	1452(0)	63(0)	496(1)	1460(0)
61(0)	493(2)	1453(19)	66(0)	509(4)	1461(11)
64(0)	517(0)	1459(21)	79(0)	535(0)	1469(13)
78(0)	555(1)	1459(13)	84(0)	560(0)	1470(14)
84(0)	557(35)	1468(2)	86(0)	576(37)	1477(4)
93(0)	565(0)	1470(11)	87(0)	623(34)	1479(13)
96(0)	619(34)	1729(159)	102(2)	623(127)	1755(153)
124(0)	655(0)	1740(59)	106(0)	654(0)	1766(88)
127(1)	669(15)	1939(3064)	111(0)	666(141)	2003(2347)
137(0)	740(129)	2000(0)	143(0)	706(49)	2038(0)
151(2)	864(1)	2032(2245)	151(1)	854(0)	2099(873)
154(0)	872(2)	2087(694)	157(6)	862(1)	2114(785)
161(1)	928(0)	3026(0)	160(1)	926(0)	3048(0)
206(92)	929(3)	3026(38)	223(2)	927(0)	3048(16)
214(1)	935(0)	3029(86)	232(6)	933(0)	3052(38)
258(37)	935(2)	3030(50)	267(8)	933(3)	3053(29)
269(59)	1082(0)	3112(0)	282(0)	1089(1)	3118(0)
278(0)	1082(1)	3112(0)	291(0)	1089(0)	3118(0)
284(0)	1093(14)	3113(12)	303(64)	1093(20)	3122(13)
328(1)	1100(24)	3114(53)	327(0)	1099(26)	3123(21)
341(5)	1246(0)	3147(0)	351(8)	1240(0)	3166(0)
403(114)	1249(3)	3148(21)	423(0)	1242(2)	3167(18)
420(0)	1384(12)	3150(60)	432(12)	1387(0)	3168(25)
429(11)	1385(0)	3150(20)	443(3)	1388(72)	3168(13)

Table S196. Harmonic vibrational frequencies (in cm^{-1}) and infrared intensities (in parentheses, in km/mol) for the $(Me_2C{=}N)_2Fe_2(CO)_4$ structure **2-4T-7**.

M06-L			B3PW91-D3		
26(1)	436(2)	1402(10)	31(1)	446(2)	1403(10)
29(1)	466(0)	1405(53)	32(1)	475(1)	1405(61)
42(0)	484(2)	1441(5)	37(1)	484(4)	1451(9)
44(0)	489(5)	1446(1)	38(0)	491(5)	1458(1)
48(4)	492(7)	1449(19)	55(5)	508(3)	1459(25)
65(1)	509(0)	1451(2)	67(0)	530(14)	1460(0)
77(0)	512(8)	1455(6)	84(0)	530(1)	1468(4)
83(1)	542(116)	1459(4)	85(2)	561(11)	1469(5)
92(0)	543(33)	1467(12)	91(0)	566(165)	1477(5)
96(1)	554(21)	1468(3)	102(2)	574(46)	1477(12)
104(0)	588(86)	1608(3)	108(0)	606(91)	1631(2)
110(0)	604(40)	1635(28)	112(0)	617(50)	1660(30)
115(1)	749(6)	1979(892)	119(1)	749(7)	1995(723)
137(1)	839(17)	1996(491)	141(2)	844(18)	2013(605)
147(1)	863(19)	2016(2065)	154(1)	856(20)	2034(2441)
150(0)	951(7)	2059(1079)	158(0)	955(10)	2079(1099)
159(3)	952(3)	3021(69)	167(4)	955(1)	3046(9)
171(0)	957(4)	3024(8)	169(0)	959(5)	3047(3)
192(1)	967(1)	3027(80)	189(2)	965(0)	3050(30)
259(3)	987(8)	3027(32)	265(3)	996(17)	3050(32)
267(3)	1068(13)	3106(1)	272(4)	1073(41)	3119(0)
344(0)	1070(3)	3107(24)	348(0)	1078(2)	3119(7)
356(1)	1077(31)	3111(10)	355(1)	1080(8)	3121(7)
363(3)	1131(64)	3111(12)	363(3)	1145(57)	3121(9)
402(5)	1303(4)	3143(14)	386(6)	1300(1)	3161(4)
409(3)	1305(8)	3144(16)	407(2)	1301(11)	3161(6)
426(10)	1391(2)	3160(17)	416(1)	1399(3)	3176(8)
435(2)	1393(1)	3161(5)	438(19)	1400(5)	3177(1)

Table S197. Harmonic vibrational frequencies (in cm^{-1}) and infrared intensities (in parentheses, in km/mol) for the $(Me_2C{=}N)_2Fe_2(CO)_4$ structure **2-4S-8.**

M06-L			B3PW91-D3		
20(1)	430(2)	1400(14)	25(0)	438(5)	1401(18)
32(0)	460(9)	1406(48)	34(0)	465(10)	1405(66)
35(0)	474(1)	1440(13)	43(0)	479(0)	1451(14)
43(0)	488(3)	1444(18)	48(0)	484(2)	1454(17)
55(1)	497(4)	1445(4)	56(3)	504(12)	1457(3)
70(1)	518(10)	1453(9)	63(1)	526(4)	1461(12)
78(0)	525(9)	1455(15)	82(2)	532(83)	1464(15)
83(2)	539(112)	1461(9)	82(4)	554(90)	1471(4)
92(3)	549(11)	1469(6)	90(1)	564(39)	1476(25)
103(0)	553(21)	1470(23)	101(0)	570(31)	1481(12)
109(0)	585(31)	1610(84)	109(0)	599(28)	1629(67)
112(2)	611(29)	1659(23)	110(3)	630(33)	1682(23)
119(0)	763(1)	1968(147)	114(0)	761(3)	1986(344)
121(0)	832(6)	1990(1707)	126(1)	827(2)	2008(1673)
132(1)	870(8)	1994(1115)	134(1)	871(7)	2019(1192)
143(0)	940(1)	2057(1338)	143(0)	938(2)	2081(1531)
152(1)	955(0)	2997(54)	153(1)	955(3)	3015(24)
167(1)	958(7)	3019(87)	167(1)	959(2)	3048(30)
230(1)	966(2)	3037(19)	225(3)	966(4)	3053(13)
231(5)	993(18)	3042(31)	238(10)	994(16)	3063(19)
282(3)	1059(37)	3073(37)	291(2)	1066(53)	3081(19)
346(3)	1077(15)	3092(19)	340(5)	1078(16)	3109(8)
351(5)	1082(1)	3119(9)	351(7)	1088(1)	3125(1)
357(3)	1145(40)	3126(9)	356(2)	1158(42)	3129(7)
367(3)	1301(1)	3131(22)	363(7)	1298(1)	3140(12)
383(2)	1307(4)	3154(16)	393(6)	1303(4)	3166(4)
400(9)	1389(14)	3168(7)	402(11)	1397(18)	3173(7)
416(5)	1393(5)	3169(8)	420(5)	1399(2)	3191(4)

Table S198. Harmonic vibrational frequencies (in cm^{-1}) and infrared intensities (in parentheses, in km/mol) for the $(Me_2C{=}N)_2Fe_2(CO)_4$ structure **2-4S-9.**

M06-L			B3PW91-D3		
22(1)	469(12)	1385(19)	11(2)	476(6)	1395(26)
31(2)	489(15)	1388(5)	27(1)	492(15)	1403(32)
44(2)	497(21)	1396(12)	44(3)	512(4)	1428(57)
58(0)	512(11)	1430(5)	56(0)	520(32)	1435(29)
67(1)	521(2)	1444(1)	63(2)	528(12)	1454(6)
81(2)	564(31)	1447(4)	80(1)	564(8)	1462(3)
88(1)	580(11)	1464(13)	94(1)	581(29)	1467(44)
95(1)	585(27)	1472(34)	101(0)	598(27)	1476(12)
102(2)	610(18)	1484(0)	102(2)	608(7)	1506(14)
113(2)	662(92)	1516(9)	115(2)	632(9)	1513(25)
130(2)	693(9)	1544(89)	121(1)	678(35)	1526(14)
144(1)	704(17)	1584(2)	139(0)	701(10)	1600(12)
150(3)	719(5)	1979(809)	150(1)	727(19)	1986(1055)
160(1)	829(6)	2013(1315)	162(0)	827(6)	2035(1849)
180(1)	880(18)	2019(2636)	183(5)	877(4)	2042(928)
194(1)	945(3)	2060(786)	195(1)	930(2)	2082(969)
197(1)	948(1)	2642(58)	211(1)	946(0)	2594(9)
239(0)	971(1)	2690(7)	232(1)	973(2)	2718(5)
275(1)	998(3)	2905(13)	271(2)	997(4)	2811(8)
315(3)	1020(5)	2997(21)	311(2)	1028(9)	3042(13)
331(1)	1043(1)	3022(35)	329(3)	1048(11)	3050(5)
386(6)	1064(28)	3069(41)	384(6)	1068(0)	3077(9)
414(2)	1067(10)	3087(28)	399(3)	1075(83)	3126(4)
417(12)	1166(12)	3114(9)	414(16)	1188(51)	3129(4)
423(18)	1266(17)	3117(8)	414(4)	1260(14)	3130(6)
445(32)	1294(18)	3145(23)	441(1)	1285(7)	3156(17)
449(3)	1361(33)	3149(10)	448(2)	1341(12)	3162(2)
457(10)	1374(47)	3162(19)	458(2)	1388(5)	3181(8)

Table S199. Harmonic vibrational frequencies (in cm^{-1}) and infrared intensities (in parentheses, in km/mol) for the $(Me_2C{=}N)_2Cr_2(CO)_6$ structure **2-6S-1.**

M06-L			B3PW91-D3		
17(2)	434(16)	1374(8)	13(2)	451(1)	1382(15)
30(2)	443(0)	1379(27)	33(2)	460(10)	1384(36)
32(1)	443(12)	1383(14)	37(1)	467(2)	1389(21)
62(0)	452(16)	1383(1)	65(0)	468(12)	1391(5)
64(3)	462(0)	1436(60)	65(4)	473(1)	1444(75)
67(0)	469(15)	1440(9)	70(0)	479(24)	1449(7)
69(2)	471(8)	1443(16)	77(1)	489(5)	1459(17)
77(0)	479(1)	1450(17)	82(0)	504(16)	1462(12)
86(1)	501(26)	1452(8)	90(1)	519(29)	1465(5)
88(1)	508(10)	1460(18)	91(1)	531(12)	1476(1)
91(1)	521(0)	1465(1)	100(1)	541(0)	1479(23)
98(2)	532(32)	1474(4)	108(1)	552(63)	1482(6)
100(1)	557(68)	1614(153)	111(0)	574(97)	1632(201)
109(0)	598(25)	1683(21)	117(0)	617(29)	1709(20)
111(0)	607(26)	1981(32)	120(1)	621(36)	2002(37)
127(0)	621(11)	1990(1299)	133(0)	638(5)	2010(1465)
142(0)	623(5)	1996(1267)	137(0)	641(15)	2018(1304)
161(0)	655(3)	1999(1085)	178(0)	668(7)	2021(1105)
169(0)	670(4)	2033(2666)	183(1)	695(5)	2049(2799)
177(0)	675(110)	2065(14)	194(1)	698(150)	2086(13)
192(1)	863(0)	3035(2)	210(1)	861(2)	3052(1)
198(1)	868(26)	3035(9)	215(0)	871(22)	3055(3)
208(3)	899(4)	3037(101)	217(1)	901(5)	3056(5)
278(0)	934(2)	3037(11)	288(0)	938(2)	3059(28)
281(1)	944(3)	3118(7)	291(0)	948(2)	3128(10)
299(32)	956(1)	3118(20)	303(56)	959(1)	3129(6)
306(0)	1077(0)	3120(12)	318(0)	1085(0)	3131(8)
356(3)	1078(0)	3122(14)	370(3)	1086(0)	3131(4)
391(7)	1084(22)	3172(7)	403(7)	1090(38)	3186(3)
399(13)	1087(19)	3173(22)	415(5)	1093(5)	3186(6)
427(27)	1245(2)	3173(8)	440(41)	1240(3)	3187(6)
428(3)	1256(2)	3174(4)	446(5)	1257(3)	3187(2)

Table S200. Harmonic vibrational frequencies (in cm^{-1}) and infrared intensities (in parentheses, in km/mol) for the $(Me_2C{=}N)_2Cr_2(CO)_6$ structure **2-6T-2.**

M06-L			B3PW91-D3		
13(2)	451(1)	1382(15)	30(1)	420(27)	1387(0)
33(2)	460(10)	1384(36)	41(0)	428(3)	1388(69)
37(1)	467(2)	1389(21)	41(1)	429(12)	1389(9)
65(0)	468(12)	1391(5)	52(0)	450(11)	1390(4)
65(4)	473(1)	1444(75)	58(1)	456(6)	1456(20)
70(0)	479(24)	1449(7)	62(1)	456(16)	1457(10)
77(1)	489(5)	1459(17)	62(0)	468(43)	1461(0)
82(0)	504(16)	1462(12)	74(2)	478(24)	1461(19)
90(1)	519(29)	1465(5)	74(1)	486(5)	1472(4)
91(1)	531(12)	1476(1)	76(1)	497(4)	1472(3)
100(1)	541(0)	1479(23)	82(0)	512(0)	1480(2)
108(1)	552(63)	1482(6)	88(1)	518(2)	1481(28)
111(0)	574(97)	1632(201)	93(1)	539(20)	1730(114)
117(0)	617(29)	1709(20)	100(0)	549(32)	1739(42)
120(1)	621(36)	2002(37)	121(1)	563(48)	1955(367)
133(0)	638(5)	2010(1465)	126(0)	602(5)	2020(894)
137(0)	641(15)	2018(1304)	144(7)	608(8)	2023(3539)
178(0)	668(7)	2021(1105)	147(2)	634(1)	2047(910)
183(1)	695(5)	2049(2799)	149(0)	647(106)	2059(1478)
194(1)	698(150)	2086(13)	164(11)	672(23)	2113(589)
210(1)	861(2)	3052(1)	175(0)	853(4)	3049(0)
215(0)	871(22)	3055(3)	214(5)	857(3)	3049(9)
217(1)	901(5)	3056(5)	242(0)	927(11)	3053(39)
288(0)	938(2)	3059(28)	274(0)	928(2)	3053(18)
291(0)	948(2)	3128(10)	281(38)	943(0)	3122(0)
303(56)	959(1)	3129(6)	285(2)	943(1)	3122(10)
318(0)	1085(0)	3131(8)	297(23)	1084(0)	3126(14)
370(3)	1086(0)	3131(4)	301(12)	1085(1)	3126(8)
403(7)	1090(38)	3186(3)	367(3)	1090(26)	3168(5)
415(5)	1093(5)	3186(6)	376(119)	1094(17)	3168(15)
440(41)	1240(3)	3187(6)	406(9)	1236(0)	3174(17)
446(5)	1257(3)	3187(2)	413(0)	1237(2)	3174(6)

Table S201. Harmonic vibrational frequencies (in cm^{-1}) and infrared intensities (in parentheses, in km/mol) for the $(Me_2C{=}N)_2Cr_2(CO)_6$ structure **2-6T-3.**

M06-L			B3PW91-D3		
21(1)	413(2)	1378(13)	22(1)	429(2)	1385(26)
31(1)	416(4)	1378(4)	29(1)	429(3)	1386(5)
41(3)	430(20)	1382(57)	39(2)	440(22)	1387(62)
49(2)	432(9)	1382(0)	46(1)	445(3)	1387(1)
52(0)	435(1)	1439(66)	52(0)	455(10)	1450(87)
56(0)	445(8)	1440(3)	63(0)	465(18)	1451(12)
60(0)	458(4)	1445(9)	63(0)	468(14)	1456(16)
63(2)	461(2)	1445(17)	68(0)	477(1)	1456(6)
64(1)	473(10)	1451(23)	72(2)	495(6)	1469(7)
75(0)	479(3)	1452(4)	78(0)	496(1)	1469(4)
81(1)	490(2)	1470(5)	81(1)	506(4)	1481(5)
85(1)	496(4)	1470(8)	86(1)	508(8)	1481(14)
89(1)	528(0)	1653(425)	91(1)	544(5)	1675(495)
96(1)	529(9)	1666(57)	97(1)	547(12)	1689(61)
98(0)	564(4)	1956(199)	101(0)	581(8)	1978(265)
114(0)	575(17)	1963(1939)	108(0)	592(28)	1985(1892)
116(0)	596(116)	2003(324)	109(0)	598(144)	2028(366)
136(0)	618(0)	2005(1415)	146(0)	619(0)	2029(1549)
149(0)	660(15)	2048(2016)	155(0)	677(11)	2078(1591)
154(0)	671(3)	2073(246)	160(0)	687(3)	2099(317)
171(0)	851(4)	3033(0)	167(0)	847(2)	3051(4)
183(0)	857(0)	3033(24)	183(0)	852(0)	3051(1)
206(0)	904(9)	3037(67)	205(0)	910(11)	3055(21)
257(4)	908(0)	3037(12)	257(3)	913(0)	3055(3)
273(12)	941(0)	3118(17)	282(8)	942(0)	3126(15)
276(2)	941(1)	3118(3)	282(0)	942(1)	3126(4)
289(3)	1081(1)	3122(1)	300(5)	1086(0)	3129(2)
305(34)	1081(0)	3122(36)	311(45)	1086(3)	3129(14)
369(5)	1083(2)	3164(10)	373(3)	1086(0)	3178(9)
370(9)	1086(1)	3164(25)	378(4)	1090(1)	3178(15)
400(1)	1246(8)	3170(23)	419(1)	1242(14)	3185(12)
400(3)	1247(1)	3170(0)	420(1)	1243(1)	3185(0)

Table S202. Harmonic vibrational frequencies (in cm^{-1}) and infrared intensities (in parentheses, in km/mol) for the $(Me_2C=N)_2Cr_2(CO)_6$ structure **2-6S-4.**

M06-L			B3PW91-D3		
25(2)	434(19)	1374(8)	13(2)	451(1)	1382(15)
32(2)	443(0)	1379(27)	33(2)	460(10)	1384(36)
33(1)	443(12)	1383(14)	37(1)	467(2)	1389(21)
63(0)	453(15)	1383(1)	65(0)	468(12)	1391(5)
65(3)	463(0)	1435(74)	65(4)	473(1)	1444(75)
66(0)	470(14)	1439(5)	70(0)	479(24)	1449(7)
71(2)	473(9)	1449(15)	77(1)	489(5)	1459(17)
78(0)	480(1)	1452(8)	82(0)	504(16)	1462(12)
86(2)	501(26)	1460(2)	90(1)	519(29)	1465(5)
88(1)	510(9)	1463(3)	91(1)	531(12)	1476(1)
92(0)	521(0)	1471(3)	100(1)	541(0)	1479(23)
93(2)	532(32)	1475(22)	108(1)	552(63)	1482(6)
97(1)	558(67)	1615(154)	111(0)	574(97)	1632(201)
110(0)	598(25)	1684(20)	117(0)	617(29)	1709(20)
110(0)	608(26)	1981(24)	120(1)	621(36)	2002(37)
129(0)	621(11)	1990(1409)	133(0)	638(5)	2010(1465)
141(0)	624(6)	1995(1134)	137(0)	641(15)	2018(1304)
173(0)	655(4)	1999(1095)	178(0)	668(7)	2021(1105)
184(0)	670(4)	2032(2688)	183(1)	695(5)	2049(2799)
190(1)	675(110)	2065(12)	194(1)	698(150)	2086(13)
193(3)	863(3)	3035(9)	210(1)	861(2)	3052(1)
205(1)	872(24)	3035(2)	215(0)	871(22)	3055(3)
223(1)	900(5)	3037(101)	217(1)	901(5)	3056(5)
281(0)	933(2)	3037(11)	288(0)	938(2)	3059(28)
285(0)	948(3)	3118(7)	291(0)	948(2)	3128(10)
297(32)	956(1)	3118(20)	303(56)	959(1)	3129(6)
312(0)	1077(0)	3120(12)	318(0)	1085(0)	3131(8)
361(2)	1081(0)	3122(13)	370(3)	1086(0)	3131(4)
391(8)	1086(32)	3173(7)	403(7)	1090(38)	3186(3)
399(13)	1088(7)	3173(15)	415(5)	1093(5)	3186(6)
427(25)	1244(2)	3175(8)	440(41)	1240(3)	3187(6)
431(2)	1258(2)	3177(11)	446(5)	1257(3)	3187(2)

Table S203. Harmonic vibrational frequencies (in cm^{-1}) and infrared intensities (in parentheses, in km/mol) for the $(Me_2C{=}N)_2Cr_2(CO)_6$ structure **2-6S-5.**

M06-L			B3PW91-D3		
32(1)	448(0)	1389(7)	35(1)	454(3)	1399(18)
35(1)	458(8)	1390(5)	41(1)	456(0)	1399(11)
43(4)	459(2)	1403(7)	46(1)	467(0)	1404(5)
51(0)	463(5)	1406(59)	48(4)	469(2)	1406(66)
56(0)	464(6)	1441(14)	62(0)	482(7)	1451(21)
71(0)	480(7)	1445(5)	66(2)	484(12)	1456(6)
71(1)	490(11)	1450(6)	77(0)	501(17)	1459(13)
75(1)	491(5)	1455(1)	80(1)	505(6)	1464(2)
78(1)	505(2)	1457(14)	85(0)	526(3)	1469(9)
79(3)	518(1)	1458(16)	86(3)	535(3)	1472(11)
92(0)	525(2)	1470(1)	98(0)	537(1)	1480(4)
97(5)	564(7)	1471(24)	103(0)	571(9)	1482(29)
98(0)	616(12)	1660(34)	105(1)	627(8)	1678(43)
105(0)	621(9)	1675(0)	109(0)	631(12)	1697(2)
106(1)	634(5)	1873(13)	110(1)	641(11)	1905(1)
115(0)	636(3)	1885(1020)	117(4)	649(1)	1921(1201)
130(0)	703(2)	1971(1042)	130(0)	707(33)	1987(1238)
138(0)	704(29)	1981(843)	145(1)	714(4)	1994(866)
140(0)	762(2)	2016(1695)	146(0)	760(1)	2033(1592)
148(0)	825(2)	2056(1053)	152(0)	828(1)	2073(1074)
173(0)	863(5)	3027(33)	173(0)	859(3)	3052(1)
177(2)	945(4)	3027(2)	175(1)	948(3)	3052(7)
186(0)	948(3)	3035(53)	177(0)	952(3)	3060(16)
235(0)	955(2)	3038(10)	239(0)	956(2)	3060(4)
247(1)	964(1)	3122(8)	249(1)	962(1)	3131(3)
349(0)	981(1)	3123(1)	350(0)	991(1)	3131(1)
367(4)	1076(16)	3124(21)	369(5)	1078(19)	3133(8)
377(1)	1082(2)	3124(1)	381(2)	1088(0)	3134(2)
386(49)	1083(1)	3134(7)	389(41)	1090(1)	3152(11)
409(0)	1130(41)	3134(35)	410(1)	1144(43)	3152(3)
427(0)	1300(0)	3167(14)	433(0)	1299(0)	3183(5)
436(0)	1301(4)	3167(1)	445(1)	1301(4)	3183(0)

Table S204. Harmonic vibrational frequencies (in cm^{-1}) and infrared intensities (in parentheses, in km/mol) for the $(Me_2C{=}N)_2Cr_2(CO)_6$ structure **2-6S-6.**

M06-L			B3PW91-D3		
17(2)	458(11)	1348(15)	35(1)	454(3)	1399(18)
28(1)	463(15)	1367(53)	41(1)	456(0)	1399(11)
38(1)	468(4)	1390(10)	46(1)	467(0)	1404(5)
60(1)	473(3)	1407(28)	48(4)	469(2)	1406(66)
67(0)	480(7)	1420(34)	62(0)	482(7)	1451(21)
72(0)	490(8)	1442(17)	66(2)	484(12)	1456(6)
77(0)	510(18)	1451(5)	77(0)	501(17)	1459(13)
80(1)	515(10)	1454(6)	80(1)	505(6)	1464(2)
87(1)	520(12)	1457(25)	85(0)	526(3)	1469(9)
91(2)	530(22)	1460(34)	86(3)	535(3)	1472(11)
92(1)	540(8)	1473(22)	98(0)	537(1)	1480(4)
97(2)	564(4)	1524(55)	103(0)	571(9)	1482(29)
106(3)	610(35)	1621(10)	105(1)	627(8)	1678(43)
122(0)	632(19)	1663(31)	109(0)	631(12)	1697(2)
138(0)	637(5)	1951(780)	110(1)	641(11)	1905(1)
161(2)	642(19)	1964(699)	117(4)	649(1)	1921(1201)
164(0)	655(13)	1987(1028)	130(0)	707(33)	1987(1238)
173(0)	682(71)	2000(817)	145(1)	714(4)	1994(866)
190(2)	709(34)	2021(2416)	146(0)	760(1)	2033(1592)
224(2)	838(4)	2060(793)	152(0)	828(1)	2073(1074)
225(2)	891(4)	2593(3)	173(0)	859(3)	3052(1)
291(4)	924(4)	2842(44)	175(1)	948(3)	3052(7)
302(4)	960(5)	2913(7)	177(0)	952(3)	3060(16)
334(3)	976(4)	3038(26)	239(0)	956(2)	3060(4)
353(5)	994(12)	3040(15)	249(1)	962(1)	3131(3)
368(11)	1003(22)	3084(27)	350(0)	991(1)	3131(1)
402(16)	1042(1)	3118(11)	369(5)	1078(19)	3133(8)
415(0)	1070(14)	3132(10)	381(2)	1088(0)	3134(2)
426(12)	1096(18)	3158(7)	389(41)	1090(1)	3152(11)
429(0)	1111(18)	3168(9)	410(1)	1144(43)	3152(3)
441(15)	1286(22)	3178(1)	433(0)	1299(0)	3183(5)
452(2)	1305(9)	3179(7)	445(1)	1301(4)	3183(0)

Table S205. Harmonic vibrational frequencies (in cm^{-1}) and infrared intensities (in parentheses, in km/mol) for the $(Me_2C{=}N)_2Cr_2(CO)_6$ structure **2-6T-7.**

M06-L			B3PW91-D3		
25(1)	409(20)	1389(7)	-147(13)	422(3)	1398(16)
36(0)	418(2)	1390(8)	39(1)	443(12)	1398(14)
38(1)	429(10)	1403(7)	47(1)	448(15)	1404(5)
48(0)	440(5)	1406(66)	51(1)	451(7)	1406(78)
57(1)	446(5)	1440(17)	60(0)	454(3)	1451(22)
59(0)	456(20)	1446(6)	62(0)	469(2)	1458(10)
69(0)	460(2)	1451(25)	72(0)	469(43)	1459(37)
72(2)	460(2)	1452(0)	75(1)	470(3)	1461(0)
74(2)	470(1)	1454(14)	80(0)	483(1)	1468(16)
80(1)	491(4)	1456(12)	85(3)	514(8)	1468(7)
81(0)	508(2)	1468(3)	94(1)	521(4)	1479(8)
91(0)	543(63)	1470(22)	100(0)	554(81)	1482(24)
95(0)	555(5)	1657(30)	106(0)	568(2)	1673(35)
112(0)	558(12)	1670(0)	107(1)	576(27)	1690(0)
115(1)	562(0)	1829(838)	111(1)	584(5)	1843(1081)
118(0)	574(15)	1832(31)	114(0)	595(15)	1869(21)
136(0)	646(53)	1985(2721)	129(2)	648(58)	1998(4241)
137(0)	657(2)	1996(1401)	141(1)	667(4)	2006(433)
155(1)	760(6)	2000(597)	150(0)	757(6)	2013(623)
156(0)	828(2)	2065(1150)	161(0)	830(2)	2082(1190)
189(1)	864(6)	3029(37)	171(2)	857(4)	3052(10)
192(1)	949(2)	3029(8)	182(0)	951(2)	3052(3)
221(0)	949(5)	3036(64)	182(1)	952(2)	3059(22)
235(0)	954(0)	3038(12)	213(0)	957(1)	3059(4)
247(1)	964(0)	3121(12)	238(1)	963(0)	3130(6)
283(3)	983(2)	3122(0)	249(2)	992(3)	3130(4)
355(0)	1076(15)	3123(16)	352(0)	1078(23)	3133(7)
372(3)	1082(2)	3123(10)	373(2)	1088(0)	3133(3)
380(1)	1083(7)	3139(7)	383(0)	1089(1)	3153(2)
382(0)	1131(42)	3139(22)	389(1)	1142(41)	3153(6)
396(42)	1301(0)	3170(14)	407(26)	1298(0)	3183(5)
405(2)	1302(3)	3171(1)	418(11)	1300(3)	3184(0)

Table S206. Harmonic vibrational frequencies (in cm^{-1}) and infrared intensities (in parentheses, in km/mol) for the $(Me_2C{=}N)_2Cr_2(CO)_6$ structure **2-6S-8.**

M06-L			B3PW91-D3		
22(1)	449(3)	1370(17)	25(3)	467(4)	1343(31)
29(4)	460(17)	1381(26)	33(1)	477(14)	1358(46)
42(3)	474(10)	1395(51)	41(1)	479(22)	1400(36)
45(0)	478(8)	1398(2)	65(1)	481(3)	1411(10)
66(2)	484(5)	1420(7)	70(0)	493(6)	1427(49)
74(1)	505(11)	1427(58)	77(0)	502(4)	1455(21)
79(0)	515(6)	1439(17)	81(0)	520(24)	1456(21)
84(1)	517(15)	1444(1)	85(1)	527(11)	1462(7)
85(2)	525(11)	1458(13)	92(1)	536(14)	1465(6)
88(1)	538(4)	1467(29)	98(2)	543(23)	1466(29)
93(2)	570(5)	1499(8)	98(1)	551(9)	1482(21)
96(5)	602(6)	1525(12)	102(2)	570(7)	1552(59)
99(0)	616(25)	1582(32)	109(3)	627(38)	1597(23)
113(2)	640(13)	1620(4)	129(0)	643(20)	1702(22)
131(8)	645(5)	1936(876)	148(0)	651(8)	1962(907)
138(2)	651(17)	1955(1019)	165(1)	654(20)	1977(831)
151(4)	689(35)	1977(1061)	168(2)	667(29)	2011(1040)
166(2)	707(12)	1990(802)	173(0)	696(85)	2022(900)
173(2)	718(13)	2026(1784)	193(2)	725(31)	2039(2069)
187(2)	837(8)	2051(755)	216(1)	837(5)	2078(912)
195(3)	876(0)	2721(24)	226(5)	889(3)	2483(6)
223(1)	932(1)	2874(9)	293(2)	924(2)	2763(37)
293(2)	940(1)	2954(14)	306(5)	969(3)	2930(3)
321(3)	956(2)	3025(12)	336(7)	977(2)	3056(6)
338(5)	984(6)	3035(6)	357(3)	997(10)	3063(3)
343(3)	1004(9)	3084(22)	377(11)	1013(15)	3107(7)
383(8)	1053(3)	3118(6)	397(9)	1043(1)	3123(4)
409(2)	1061(61)	3123(5)	419(2)	1076(13)	3145(5)
412(0)	1067(10)	3125(4)	423(9)	1094(42)	3163(1)
425(1)	1119(18)	3160(12)	433(2)	1122(15)	3182(3)
436(4)	1283(7)	3164(4)	447(17)	1280(20)	3193(0)
438(12)	1298(3)	3184(9)	461(1)	1299(19)	3196(2)

Complete Gaussian 16 reference (Reference 15)

Cite this work as:

Gaussian 16, Revision C.01,
M. J. Frisch, G. W. Trucks, H. B. Schlegel, G. E. Scuseria,
M. A. Robb, J. R. Cheeseman, G. Scalmani, V. Barone,
G. A. Petersson, H. Nakatsuji, X. Li, M. Caricato, A. V. Marenich,
J. Bloino, B. G. Janesko, R. Gomperts, B. Mennucci, H. P. Hratchian,
J. V. Ortiz, A. F. Izmaylov, J. L. Sonnenberg, D. Williams-Young,
F. Ding, F. Lipparini, F. Egidi, J. Goings, B. Peng, A. Petrone,
T. Henderson, D. Ranasinghe, V. G. Zakrzewski, J. Gao, N. Rega,
G. Zheng, W. Liang, M. Hada, M. Ehara, K. Toyota, R. Fukuda,
J. Hasegawa, M. Ishida, T. Nakajima, Y. Honda, O. Kitao, H. Nakai,
T. Vreven, K. Throssell, J. A. Montgomery, Jr., J. E. Peralta,
F. Ogliaro, M. J. Bearpark, J. J. Heyd, E. N. Brothers, K. N. Kudin,
V. N. Staroverov, T. A. Keith, R. Kobayashi, J. Normand,
K. Raghavachari, A. P. Rendell, J. C. Burant, S. S. Iyengar,
J. Tomasi, M. Cossi, J. M. Millam, M. Klene, C. Adamo, R. Cammi,
J. W. Ochterski, R. L. Martin, K. Morokuma, O. Farkas,
J. B. Foresman, and D. J. Fox, Gaussian, Inc., Wallingford CT, 2019.
